# Reducing the information gap on Loricarioidei (Siluriformes) mitochondrial genomics

**DOI:** 10.1186/s12864-017-3709-3

**Published:** 2017-05-04

**Authors:** Daniel Andrade Moreira, Paulo Andreas Buckup, Carolina Furtado, Adalberto Luis Val, Renata Schama, Thiago Estevam Parente

**Affiliations:** 1 Laboratório de Toxicologia Ambiental, Escola Nacional de Saúde Pública (ENSP), Fundação Oswaldo Cruz (FIOCRUZ), Av. Brasil, 4036, Rio de Janeiro, Brasil; 2 Laboratório de Biologia Computacional e Sistemas, Instituto Oswaldo Cruz (IOC), Fundação Oswaldo Cruz (FIOCRUZ), Av. Brasil, 4365, Rio de Janeiro, Brasil; 3 Departamento de Vertebrados, Museu Nacional, Universidade Federal do Rio de Janeiro (UFRJ), Quinta da Boa Vista, Rio de Janeiro, RJ Brasil; 4 Unidade de Genômica, Instituto Nacional do Câncer (INCA), Rua André Cavalcanti, 37, Rio de Janeiro, Brasil; 5 Laboratório de Ecofisiologia e Evolução Molecular, Instituto Nacional de Pesquisas da Amazônia (INPA), Av. André Araújo, 2936, Manaus, Brasil; 6 Laboratório de Genética Molecular de Microrganismos, Instituto Oswaldo Cruz (IOC), Fundação Oswaldo Cruz (FIOCRUZ), Av. Brasil, 4365, Rio de Janeiro, Brasil

**Keywords:** Catfishes, Teleostei, Loricariidae, Biodiversity, Evolution, Neotropical Region, Next-Generation Sequencing

## Abstract

**Background:**

The genetic diversity of Neotropical fish fauna is underrepresented in public databases. This distortion is evident for the order Siluriformes, in which the suborders Siluroidei and Loricarioidei share equivalent proportion of species, although far less is known about the genetics of the latter clade, endemic to the Neotropical Region. Recently, this information gap was evident in a study about the structural diversity of fish mitochondrial genomes, and hampered a precise chronological resolution of Siluriformes. It has also prevented molecular ecology investigations about these catfishes, their interactions with the environment, responses to anthropogenic changes and potential uses.

**Results:**

Using high-throughput sequencing, we provide the nearly complete mitochondrial genomes for 26 Loricariidae and one Callichthyidae species. Structural features were highly conserved. A notable exception was identified in the monophyletic clade comprising species of the *Hemiancistrus*, Hypostomini and *Peckoltia*-clades, a ~60 nucleotide-long deletion encompassing the seven nucleotides at the 3′ end of the Conserved Sequence Block (CSB) D of the control region. The expression of mitochondrial genes followed the usual punctuation pattern. Heteroplasmic sites were identified in most species. The retrieved phylogeny strongly corroborates the currently accepted tree, although bringing to debate the relationship between *Schizolecis guntheri* and *Pareiorhaphis garbei*, and highlighting the low genetic variability within the *Peckoltia*-clade, an eco-morphologically diverse and taxonomically problematic group.

**Conclusions:**

Herein we have launched the use of high-throughput mitochondrial genomics in the studies of the Loricarioidei species. The new genomic resources reduce the information gap on the molecular diversity of Neotropical fish fauna, impacting the capacity to investigate a variety of aspects of the molecular ecology and evolution of these fishes. Additionally, the species showing the partial CSB-D are candidate models to study the replication and transcription of vertebrate mitochondrial genome.

**Electronic supplementary material:**

The online version of this article (doi:10.1186/s12864-017-3709-3) contains supplementary material, which is available to authorized users.

## Background

Representing around 5% of vertebrate biodiversity [1], catfishes (Siluriformes) are classified in three suborders: Diplomystoidei, with seven valid species distributed in Andean areas of Argentina and Chile; Siluroidei, summing 2250 species distributed worldwide; and Loricarioidei, endemic to the Neotropical Region with 1538 valid species [1–3]. As of March 2017, while one nuclear genome and 102 complete mitochondrial genomes from Siluroidei species were deposited in GenBank, only four Loricarioidei species had their mitochondrial genome sequence publicly released (Table 1). This disproportion is even greater for general nucleotide sequences; for which the number of Siluroidei entries is almost 100 times the amount for Loricarioidei (Table 1).

**Table 1:** Disproportion of genetic information among Siluriformes’ suborders. Different types of entries in NCBI database, as well as the number of available, valid and new species described in the last 10 years for Siluriformes according to the Catalog of Fishes database

		Siluriformes		
Database	Parameter	Diplomystoidei	Loricarioidei	Siluroidei
NCBI	Nucleotide	547	8599	855963
	Nucleotide EST	-	-	498206
	Nucleotide GSS	-	-	63406
	Protein	360	6082	76138
	Structure	-	-	7
	Genome	1	4	103
	Popset	25	162	546
	GEO Datasets	-	-	118
	UniGene	-	-	204837
	PubMed Central	17	321	5513
	Gene	13	76	30421
	SRA Experiments	-	9	212
	Probe	-	-	5615
	Assembly	-	-	1
	Bio Project	-	5	86
	Bio Sample	-	9	304
	Clone DB	-	-	12
	PubChem BioAssay	-	-	1
	Taxonomy	13	1433	1766
CAL - Catalog of Fishes	Available	12	1801	3481
	Valid	7	1538	2250
	2008–2017	1	334	284

NCBI: https://www.ncbi.nlm.nih.gov/Taxonomy/Browser/wwwtax.cgi?id=7995 and the Catalog of Fishes: http://researcharchive.calacademy.org/research/ichthyology/catalog/SpeciesByFamily.asp, both accessed on March 24, 2017

This information gap on the genetic diversity of Loricarioidei fishes is evident in studies of Siluriformes [3, 4] and Otophysi [5, 6] evolution. Recently, in a time-calibrated mitogenome phylogeny of catfishes, the poor taxonomic representation of the Loricarioidei suborder was hypothesized as the most probable cause for the paraphyletic arrangement of the Callichthyidae and Loricariidae families [1]. According to those authors, although this arrangement “*does not represent a credible topology*”, it “*may have implications for the chronology of the basal siluriform nodes*” [1], and therefore, may impact studies on the historical biogeography of catfish dispersal throughout the globe.

The underrepresentation of Loricarioidei genetic information deposited in public databases has also prevented a variety of studies about these species, from molecular ecology, biogeography and phylogeny to potential uses in aquaculture or as sentinels of environmental pollution. For instance, species delimitation and phylogenetic analysis of highly speciose genera such as *Hypostomus*, *Peckoltia* and *Corydoras* could benefit from a larger volume of primary genetic resources. Access to new gene sequences would stimulate work on the biogeography and speciation processes of Loricarioidei fishes with wide distribution (e.g.: *Hoplosternum littorale*, *Callichthys callichthys*). Likewise, the lack of genetic data is a key limitation preventing studies about the molecular ecology of those Neotropical fishes.

Herein, we focused on the Loricariidae, the most species-rich family in the Loricarioidei and the fifth most species-rich family among all vertebrates [2, 7]. The nearly complete mitochondrial genome sequences from 26 species of Loricariidae and one of Callichthyidae were generated, and their structural features were analyzed. Additionally, the sequences of the 13 protein-coding genes and the two ribosomal RNAs were used to test the currently accepted phylogenetic relationships among Loricariidae subfamilies. These resources complement our recent efforts [8–12], augmenting from four to 36 the number of nearly complete mitochondrial genomes available for Loricarioidei fishes.

## Results

### The mitochondrial genomes of Loricariidae

The mitochondrial genomes from 27 species, representing 21 Loricariidae genera and *Corydoras schwartzi* (Callichthyidae), were sequenced almost to their full length. Detailed information regarding each mitogenome is available in Table 2 and Additional files 1, 2 and 3.

**Table 2:** Geographical coordinates of sampled species and their field and voucher catalog numbers. Vouchers were deposited in the Ichthyological collection of the National Museum belonging to the Federal University of Rio de Janeiro (MNRJ). Quasi-complete mitochondrial genomes were deposited in GenBank and their accession numbers are provided, along with the percentage coverage in comparison to NC015747 for Loricariidae or NC004698 for *Corydoras*

Species	Field no.	Location	Catalog no.	Accession no.	Coverage	Reference
*Hemipsilichthys nimius*	TP189	23°12′35.2″S 44°47′40.7″W (RJ)	MNRJ43650	KT239011	95.50%	This study
*Rineloricaria* cf. *lanceolata*	sp16.3	Aquarium specimen (PA)	MNRJ43638	KX087182	97.80%	This study
*Rineloricaria* sp.	TP144	22°31′06,3″S 42°53′55,5″W (RJ)	MNRJ42544	KX087183	95.40%	This study
*Loricariichthys platymetopon*	TP179	3°10′50.9″S 59°54′09.3″W (AM)	MNRJ43627	KT239018	95.50%	This study
*Loricariichthys castaneus*	TP029	21°13′08.7″S 41°18′37.7″W (RJ)	MNRJ41545	KT239015	92.30%	This study
*Loricaria cataphracta*	TP181	3°10′50.9″S 59°54′09.3″W (AM)	MNRJ43629	KX087174	98.30%	This study
*Otocinclus* cf. *hoppei*	sp10.7	Aquarium specimen (PA)	MNRJ43634	KX087176	99.00%	This study
*Hypoptopoma incognitum*	TP171	3°09′36.0″S 59°55′12.0″W (AM)	MNRJ43421	KT033767	100%	Moreira et al. (2016b) [10]
*Parotocinclus maculicauda*	TP011	22°36′01.6″S 43°05′30.1″W (RJ)	MNRJ41523	KX087179	94.90%	This study
*Hisonotus thayeri*	TP128	21°32′14.6″S 42°06′54.8″W (RJ)	MNRJ42481	KX087173	96.00%	This study
*Kronichthys heylandi*	8505	23°12′35.2″S 44°47′40.7″W (RJ)	MNRJ42082	KT239014	99.00%	This study
Neoplecostomini gen. n.	TP065	20°01′35.3″S 40°36′33.3″W (ES)	MNRJ41921	KX087172	95.50%	This study
*Neoplecostomus microps*	TP088	22°20′01.7″S 44°32′34.3″W (RJ)	MNRJ41752	KX087175	96.40%	This study
*Pareiorhaphis garbei*	TP009	22°32′03.4″S 43°02′18.7″W (RJ)	MNRJ41511	KX087178	96.80%	This study
*Schizolecis guntheri*	TP006	22°32′03.4″S 43°02′18.7″W (RJ)	MNRJ41510	KT239017	91.20%	This study
*Ancistrus* sp. 1	13.3	Aquarium specimen (PA)	MNRJ42890	KP960569	99.20%	Moreira et al. (2015) [8]
*Ancistrus* sp. 2	13.11	Aquarium specimen (PA)	MNRJ42890	KP960567	94.70%	Moreira et al. (2015) [8]
*Ancistrus multispinis*	TP003	22°32′03.4″S 43°02′18.7″W (RJ)	MNRJ41509	KT239006	96.30%	This study
*Dekeyseria amazonica*	TP165	3°09′36.0″S 59°55′12.0″W (AM)	MNRJ43618	KX087168	98.80%	This study
*Baryancistrus xanthellus*	sp11.19	Aquarium specimen (PA)	Missing	KX087167	99.10%	This study
*Pterygoplichthys* sp.	sp2	Aquarium specimen (RJ)	MNRJ43652	KX087181	96.80%	This study
*Pterygoplichthys pardalis*	TP154	3°09′36.0″S 59°55′12.0″W (AM)	MNRJ43607	KT239016	97.10%	This study
*Pterygoplichthys disjunctivus*		Not informed	Not informed	NC015747	100%	Nakatani et al. (2011) [6]
*Hypostomus* sp.	sp12.6	Aquarium specimen (PA)	MNRJ43635	KX087171	95.40%	This study
*Hypostomus* cf. *plecostomus*	TP164	3°09′36.0″S 59°55′12.0″W (AM)	MNRJ43617	KT239012	98.50%	This study
*Hypostomus affinis*	TP147	22°48′42.6″S 43°37′42.8″W (RJ)	MNRJ43256	KT239013	93.30%	This study
*Aphanotolurus emarginatus*	TP184	3°10′50.9″S 59°54′09.3″W (AM)	MNRJ43631	KT239019	96.90%	This study
*Peckoltia furcata*	sp15.2	Aquarium specimen (PA)	MNRJ43637	KX087180	96.30%	This study
*Ancistomus snethlageae*	sp17.2	Aquarium specimen (PA)	MNRJ43639	KX087166	98.90%	This study
*Panaqolus* sp.	sp4	Aquarium specimen (RJ)	MNRJ43654	KX087177	99.10%	This study
*Corydoras nattereri*	TP021	22°36′01.6″S 43°05′30.1″W (RJ)	MNRJ41520	KT239009	100%	Moreira et al. (2016a) [9]
*Corydoras schwartzi*	TP177	Aquarium specimen (AM)	MNRJ43625	KT239007	98.10%	This study
*Corydoras rabauti*		Not informed	Not informed	NC004698	100%	Saitoh et al. (2003) [16]

### Coverage, annotation and depth

**Fig. 1:**
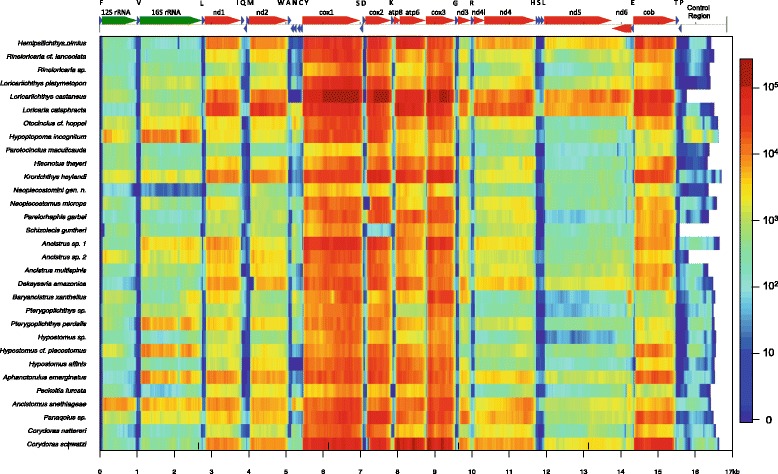
Annotation and sequencing depth of the 27 newly sequenced mitochondrial genomes. Annotation features are shown on the top of this figure as a linear representation generated by the MITOS WebServer [34]. In the upper panel, protein-coding genes are colored in *red*, ribosomal genes in *green*, and tRNA in *blue*. Each tRNA is identified with the one-letter code for the corresponding amino acid. The sequencing depth of each position of the mitochondrial genomes is indicated below the general annotation. In the lower panel, color gradient corresponds to the number of reads supporting a given nucleotide, according to the legend at the right of the figure. Illumina sequencing reads were aligned to the correspondent mitochondrial genome, using Bowtie 1.0 and counted on the Integrative Genome Viewer (IGV) [35]. Representation of read counts as a color-gradient was generated using R. For comparative purposes, the recently released mitogenomes of *H. incognitum*, *Ancistrus* sp. 1, *Ancistrus* sp.2 and *C. nattereri* were included

The new sequences covered from 91.2 to 99.2% the complete *Pterygoplichthys disjunctivus* mitogenome (Table 2), which was chosen as reference as it was one of the four complete mitogenomes of a Loricarioidei species previously available on public databases, not sequenced by our group. The few nucleotide-long gaps verified in the assembled mitochondrial genomes were located at transfer RNA (tRNA) genes. Despite these few gaps, the analysis of the sequenced mitogenomes, along with those available on GenBank, made it possible to infer the mitochondrial gene composition and order for all species, which was found to be identical to the usual pattern for vertebrates (Fig. 1).

Sequences of the two ribosomal RNA (12S and 16S rRNA) and the 13 protein-coding genes were obtained to their full length in most species. Most genes are encoded on the heavy strand, whereas NADH dehydrogenase subunit 6 (nad6) and eight tRNA are found on the light strand (Fig. 1). The majority of the 22 tRNA were completely sequenced in all species, except for *Hypostomus affinis* (Additional file 3). The complete sequence of the mitochondrial termination factor (mTERF) binding site was obtained for 15 species, while partial sequence was obtained for 10 species. The mTERF is located inside the tRNA-Leu2, justifying the highest frequency of partial sequences verified for this tRNA. Nucleotide frequencies among species were similar (Additional file 1). Among the protein-coding genes, cytochrome c oxidase subunit 1 (cox1) had the highest percentage of invariable amino acids (90%) and ATPase subunit 8 (atp8), the lowest (45%).

The origin of L-strand replication (O_L_) was sequenced, except for *Loricariichthys castaneus* and for *Hypostomus affinis*, and was conserved in most species. The mitochondrial control region (CR) was partially sequenced in all species, except *Schizolecis guntheri* in which CR was not sequenced (Fig. 1, Additional file 4). The sequence length of the CR varied from zero in *S. guntheri* to 1083 in *K. heylandi*, with a mean length of 792 nucleotides. The three CR domains (I, II and III) were found. The termination associated sequence (TAS) in the Domain I was enriched with adenine (A) and thymine (T) nucleotides, and indels were found among the species. Three conserved sequence blocks (CSB-F, CSB-E and CSB-D) were identified in Domain II.

**Fig. 2:**
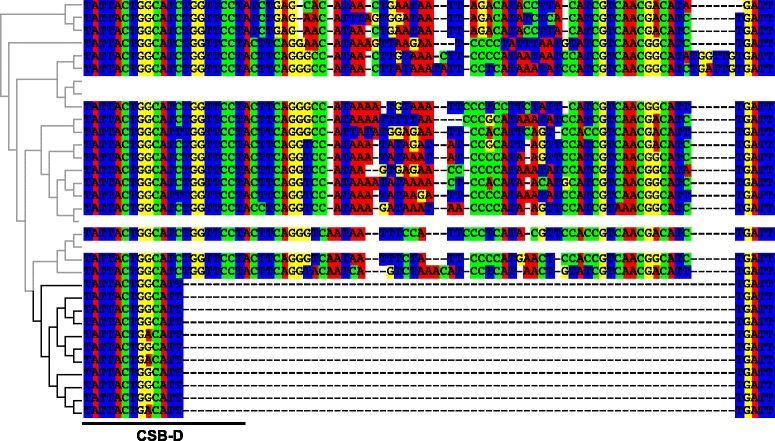
Long nucleotide deletion at the mitochondrial control region in species belonging to the Hypostomini, *Hemiancistrus* and *Peckoltia*-clades. Insertion/deletion mutations (indels) are shown as hyphen (−). The position of the Conserved Sequence Block D (CSB-D) is indicated at the left bottom. Phylogenetic relationships among sequences are shown on the left. Branches of the clade displaying the deletion are shown in *black*, while other are in *gray*. The names of each taxon are shown in Fig. 3

CSB-F was the most conserved among Loricarioidei species (Additional file 4). CSB-E was conserved among 16 species, but diverged in species of the *Hemiancistrus*, Hypostomini and *Peckoltia*-clades. While the 3′ end of CSB-E is rich in AT in the *Peckoltia*-clade and in A in the *Hemiancistrus* and Hypostomini clades, it is rich in G in the other investigated species (Additional file 4). A deletion of ~60 nucleotides, starting with the seven nucleotides at the 3′ end of CSB-D and spanning over other conserved blocks, follows the same phylogenetic trend (Fig. 2). This deletion is supported by >50 reads in each of the species sequenced herein and by the previously deposited sequence of *Pterygoplichthys disjunctivus* (NC_015747.1). At the same region, a six nucleotide-long insertion was noted in both species of *Rineloricaria* (Fig. 2). In most species, the T-homopolymer region was also found, which was followed by an AT-rich segment that characterizes the third domain. CSB-1, CSB-2 and CSB-3 were fully sequenced and highly conserved among the analyzed genomes.

Sequencing depth is a direct reflection of mitochondrial transcript expression levels. Minimum median sequencing depth was 329 found for *Parotocinclus maculicauda*, while the maximum median depth was 10,105 for *Loricaria cataphracta* (Fig. 1, Additional file 1). The variation among species is regarded as minimal differences in the amount of sample submitted to high-throughput sequencing rather than biological. On the other hand, the variation among regions within a single mitogenome is biologically significant, follows the classical mitochondrial RNA punctuation pattern and indicates the activity of a post-transcriptional gene expression mechanism. Genes coding for the three mitochondrial subunits of cytochrome c oxidase were sequenced at the greatest depth, while short intergenic and tRNA sequences showed the lowest depth (Fig. 1). Heteroplasmic positions were found in all species, except one (Additional file 5). The number of heteroplasmies varied from zero in Neoplecostomini gen. n. (TP065) to 21 in *Loricariichthys castaneus*, with median and average frequencies of 8.0 and 8.9, respectively.

### Annotation features

Detailed annotation features of the 13 protein-coding genes of each species are summarized in Additional file 2. The protein-coding genes cytochrome c oxidase subunits 2 and 3 (cox2 and cox3), NADH dehydrogenase subunits 2, 3, 4, 4l and 5 (nad2, nad3, nad4, nad4l, and nad5) were found to be highly conserved, both in total length and nucleotide composition, among species of Loricariidae and Callichthyidae. Cox2, cox3, nad2, nad3, nad4 genes were terminated with an incomplete stop codon (T--), while a complete stop codon (TAA) was found in nad4l and nad5 of all species, except for *Hemipsilichthys nimius*’ nad5 which is terminated by a TAG. In *Kronichthys heylandi* nad5 a GTG aligned to the ATG start codon that is found in the other species. However, the codon immediately before this GTG is an ATG. The other six protein-coding mitochondrial genes showed taxon-specific features that are addressed below.

The cytochrome b (cob) gene has 1138 bp and uses an incomplete stop codon (T--) in most species. However, a premature stop codon was found in all Loricariinae species, as well as in the four Ancistrinii clade species. This premature stop codon is produced by the change from TGA to either TAA or TAG and aligns with the penultimate codon of the other sequences. Within the context of the phylogenetic tree (see below) the presence of this premature stop codon is most parsimoniously interpreted as independent synapomorphies supporting the monophyly of the Loricariinae and Ancistrini. This finding is supported by other cob sequences from Loricariinae and Ancistrinii species available at GenBank, and can be interpreted as an example of convergent evolution.

The Loricariidae species possess a 1551 bp long cox1 gene, as opposed to Callichthyidae in which this gene consists of 1560 bp. The Loricariidae and Callichthyidae species sequenced herein use GTG as cox1 start codon. All Loricariidae species use TAA as a stop codon for this gene, except for two species, *Kronichthys heylandi* and *Schizolecis guntheri*, which use an incomplete stop codon (TA-). This exception cannot be confirmed in the literature as the nine cox1 genes sequences publicly available at GenBank and the Barcode of Life Data (BOLD) Systems for *K. heylandi* and the 12 for *S. guntheri* do not include the start or stop codons. Callichthyidae species were found to use AGG stop codon for cox1 termination.

The nad1 gene of the Callichthyidae species and of *Hemipsilichthys nimius* lack an amino acid at the sixth amino acid position, when aligned to the gene sequence of the remaining species. Another amino acid-long gap, at the 257th amino acid position, was found to be exclusive of Loricariinae representatives. In the case of *Loricaria cataphracta*, however, this gap consists of two amino acids. Among the Hypostominae, *Ancistrus sp. 1* and *Ancistrus sp. 2* show an additional amino acid-long gap at the penultimate position. These might be phylogenetically informative characters for further studies of Loricariinae and Callichthyidae.

The nad6 is a small protein-coding gene encoded by 522 bp in the studied Loricariidae, except for the five species of Loricariinae and *Schizolecis guntheri*. In these six exceptions, there is an amino acid-long gap at the 116th position. This gap is shared with the three *Corydoras* species, which also present another gap at the position 142nd.

The gene atp8 is coded by 168 bp in all studied species, except for four of the five Loricariinae species. In the two *Rineloricaria* species and in the two *Loricarichthys* species, an amino acid-long gap was found at the 40th position. As for the ATPase subunit 6 (atp6) gene, *Hemipsilichthys nimius* was the only species with GTG, instead of ATG, as the start codon. All Loricariidae species have the incomplete stop codon TA- to terminate the atp6 gene, while species of Callichthyidae have the complete stop codon TAA. *Corydoras* spp. (Callichthyidae) share an apomorphic insertion between the atp6 and cox3 gene. In the newly sequenced *C. schwartzi* mitogenome, this insertion has 17 nucleotides.

### Inferred phylogeny of Loricariidae subfamilies

**Fig. 3:**
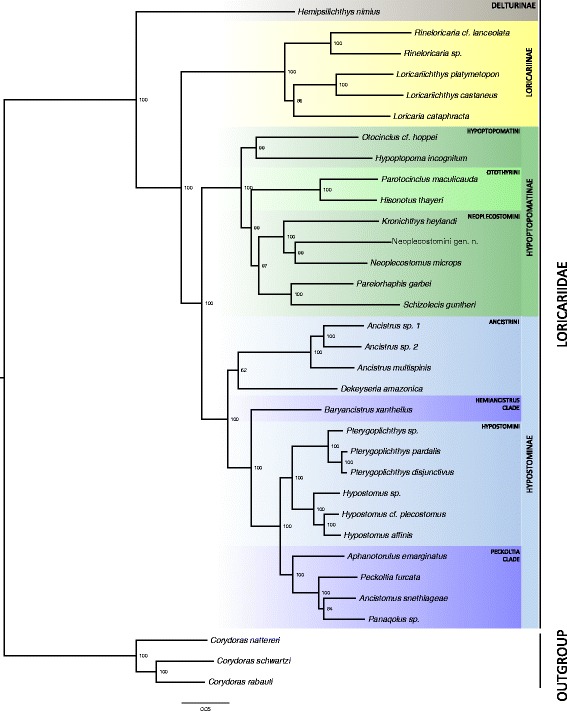
Maximum likelihood phylogeny of Loricariidae subfamilies. The two ribosomal RNA and 13 protein-coding genes (comprising 14116 nucleotides) were aligned using MUSCLE. Bootstrap support values are shown on each node and are based on 1000 replicates. Callichthyidae species were used to root the tree. Loricariidae subfamilies are highlighted in *gray* (Delturinae), *yellow* (Loricariinae), *green* (Hypoptopomatinae) and *blue* (Hypostominae). The scale bar represents the nucleotide substitution rate, using the GTR + GAMMA + I model

Sequences from the two ribosomal RNA (12S and 16SrRNA) and the 13 protein-coding mitochondrial genes were concatenated generating a super-alignment of 14116 nucleotides, for each of the 30 Loricariidae and three *Corydoras* species, producing a fully resolved maximum likelihood (ML) phylogenetic tree (Fig. 3, Final ML Optimization Likelihood: −144076.08), with branches showing high statistical support. The phylogenetic tree was rooted using sequences from *Corydoras schwartzi* and two additional *Corydoras* species obtained from GenBank (Table 2). *Hemipsilichthys nimius* (Delturinae) appears as the sister taxon of the other Loricariidae sub-families, except for Lithogeninae (not sampled in this study). The remaining loricariids form three large clusters, corresponding to the subfamily Loricariinae and a monophyletic group comprised by two clades: the subfamily Hypostominae and a clade formed by Neoplecostominae surrounded by Hypoptopomatinae.

Notably, the retrieved phylogeny strongly supports the position of Otothyrini, an Hypoptopomatinae tribe, closer to Neoplecostominae species, rather than to Hypoptopomatini species, the other traditional Hypoptopomatinae tribe. Thus, Hypoptopomatinae is considered monophyletic but, instead of two, it is composed by three tribes, Hypoptopomatini, Otothyrini and Neoplescostomini. *Schizolecis guntheri* is currently classified as a member of Otothyrini, but appeared clustered closer to *Pareiorhaphis garbei* and other members of Neoplecostomini than to the other Otothyrini.

It has been shown that for some lineages the phylogenetic tree topology retrieved using data from as few as three mitochondrial genes is the same as the topology retrieved using a concatenated alignment from the 13 protein-coding and the two rRNA genes [13]. In order to evaluate this in Neotropical catfishes, phylogenetic trees were generated using the three longest mitochondrial genes, which also harbor the majority of informative sites (cox1, nad4 and nad5) and the three mitochondrial genes with the highest performance (nad2, nad4 and nad5), as identified by Havird & Santos [13]. Those trees did not recover the same topology as encountered in the tree recovered using information on the 15 mitochondrial genes (Additional file 6). The position of *Loricaria cataphracta* was distinct in the tree based on the three genes with the highest performance, and the position of *Dekeyseria amazonica* was different in the tree based on the three longest genes (Fig. 3, Additional file 6).

### Pairwise nucleotide identity (PNI)

**Fig. 4:**
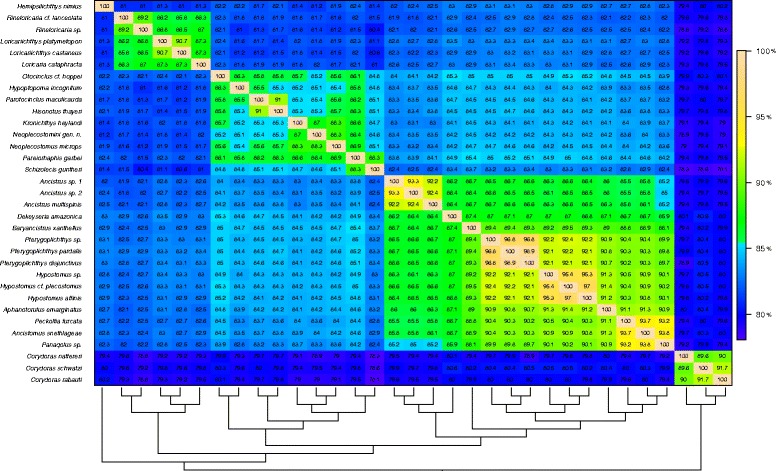
Pairwise nucleotide identity among sequenced mitochondrial genomes. Species are ordered according to the retrieved phylogeny on both axes. Species names are shown on the *left side*, and their phylogenetic relationships are depicted at the *bottom*. The color gradient represents the percentage of nucleotide identity according to the legend at the *right* of the figure

Nucleotide identities between pairs of concatenated mitochondrial genes were calculated for every combination of the 33 taxa, arranged according to their phylogenetic relationships and colored as a heat-map for visualization (Fig. 4). Four islands displaying higher pairwise nucleotide identities than their surroundings were identified. Not surprisingly, each of these islands is equivalent to major monophyletic clades. Pairwise nucleotide identities (PNI) among congeneric species displayed a higher degree of similarity, ranging from 89.2% between the two *Rineloricaria* to 95.4% between *Hypostomus* sp. and *H.* cf*. plecostomus*. PNI inside each island were most often above 85%, whereas nucleotide identity outside the islands but contained in the Loricariidae realm was most often below 85%, ranging down to 80%. Outside the Loricariidae realm, nucleotide identity between any Loricariidae species and any *Corydoras* ranged from 78 to 80%.

## Discussion

### Mitochondrial genome structure

The structure of the newly sequenced mitochondrial genomes follows the general pattern described for ostariophysian fish [14]. Among the identified differences, a ~60 nucleotide-long deletion at the control region shared by *Baryancistrus xanthellus* and species of Hypostomini and *Peckoltia*-clade highlights. In accordance with findings based on 248 ray-finned fish [14], cox1 was the most conserved protein-coding gene in the mitochondrial genomes of Loricarioidei species, while atp8 was the most variable. Corroborating Satoh et al. [14], the mitochondrial termination factor (mTERF) binding site found in the species used in this study was identical to the human sequence, implying functional conservation. The mTERF binding site is located inside the tRNA-Leu2 gene, which was the most frequent tRNA gene with partial sequence, corroborating the functional conservation.

The order of each gene in the mitogenome was inferred as the same as in other ostariophysian fish [14], which is the typical for metazoans [15]. An intriguing difference is the disruption of the classical head-to-tail junction between atp6 and cox3 among *Corydoras* species. A 17 nucleotide-long insertion was initially noted by Saitoh et al. [16] at the mitogenome of *C. rabauti*, but this trait was considered phylogenetically uninformative as it was not shared by any other species evaluated in that study [16]. Subsequently, Moreira et al. [9], observed a homologous 21 nucleotide-long insertion in the mitogenome of *C. nattereri* and hypothesized this apomorphic trait as relevant for the phylogeny of this speciose genus. The 17 nucleotide-long insertion found in *C. schwartzi* corroborates and extends that hypothesis. Although having the same length as in *C. rabauti*, these two 17 nucleotide-long insertions differ by six nucleotide substitutions (one transition and five transversions).

The mitochondrial control region is composed by three domains (I, II and III) containing several conserved sequence blocks (CSB), each showing a taxon-specific distribution in vertebrates [17–20]. Recently, it has been demonstrated that CSB-D and CSB-1 are present in 250 fish species, although their functions are not yet clear [14]. While the newly generated mitochondrial genomes confirmed the presence of CSB-1 within the Loricarioidei, more than one-third of the CSB-D was deleted in species of the *Hemiancistrus*, Hypostomini and *Peckoltia*-clades. A complete CSB-D was found in the other species, including the ones belonging to the subfamily Ancistrini, which is closely related to those clades where the partial CSB-D was identified. There are four monophyletic clades closer to the *Hemiancistrus*, Hypostomini and *Peckoltia*-clades than to Ancistrini [7], suggesting that this partial CSB-D deletion is a synapomorphy for a subset of Hypostominae species. It will be elucidative to test the occurrence of this deletion in species belonging to these four clades. Likewise, comparative studies using species in which CSB-D is partially deleted and their closest relatives with complete CSB-D might provide insights to the role played by this conserved sequence block in replication and transcription of the genome.

Among the 250 fish species studied by Satoh et al. [14], three Siluriformes species were used; two representatives of Siluroidei and *Corydoras rabauti*, a Loricarioidei representative. The sequence of *Pterygoplichthys disjunctivus* (NC_015747.1) corroborates this deletion in the CSB-D, and although it was available since 2011, it was not included in the study of Satoh et al. [14]. Here, the mitochondrial genome of *P. disjunctivus* is used to confirm our findings, as it was obtained using a combination of long and short PCR from DNA and sequenced using the Sanger method [6]. Additionally, this deletion is supported by the recently released mitochondrial genomes of *Hypancistrus zebra* (*Peckoltia*-clade; KX611143.1) [11] and *Pterygoplichthys anisitsi* (Hypostomini; KT239003, KT239004, KT239005) [12].

### Mitochondrial gene expression

The variation found in the expression of mitochondrial genes is in accordance with the punctuation pattern, in which the polycistronic RNA is cleaved at the tRNAs genes [21, 22]. This pattern has been observed for other Loricarioidei species [8–11], and suggests the fish mitochondria are transcribed as a polycistronic RNA which is latter edited to monocistronic molecules and to two bicistronic RNAs, coding for nad4/nad4L and atp8/atp6.

The high sequencing depth also allowed for the detection of heteroplasmic sites in all species, except in Neoplecostomini gen. n. (TP065), the species with the lowest count of mapped reads. On the other hand, *Loricariichthys castaneus* was the species with the highest counts of both heteroplasmic sites and mapped reads (Additional file 5). Recently, we reported similar frequencies of heteroplasmic sites found in the mitogenome of other fish species [8, 10, 11]. Mitochondrial heteroplasmies are associated with several health disorders in humans [23–25] and with insecticide resistance in insects [26]. However, the consequences of heteroplasmies on fish physiology, if any, are unknown.

### Phylogenetic analysis

Our phylogenetic analysis gives strong statistical support for most of the recently published Loricariidae phylogenies, including branches with poor bootstrap values [7, 27, 28]. Specifically, our data corroborate previous studies on the positioning of Neoplecostomini as a tribe embedded within the subfamily Hypoptopomatinae. The mitochondrial genomic data also supports the positioning of Neoplecostomini closer to Otothyrini, with Hypoptopomatini as the sister taxon of Neoplecostomini and Otothyrini, as proposed by Roxo et al. [27].

*Schizolecis guntheri* was found to be a member of Neoplecostomini rather than Otothyrini, its current classification based on morphological traits and limited molecular evidence [27, 28]. Indeed, a sister-group relationship between *S. guntheri* and *Pareiorhaphis garbei* was previously recovered by Cramer et al. [28], using a matrix of 4678 nucleotides from partial sequences of one mitochondrial (cox1) and three nuclear genes (recombination activating genes 1 and 2, and F-Reticulon 4). The *S. guntheri* and *P. garbei* specimens used by Cramer et al. [28] were sampled from the same population as in the present study. This finding is corroborated to some extend by the molecular identification of our specimens using the BOLD Systems (Additional file 7). According to the BOLD algorithm, our specimen of *P. garbei* appears in a small cluster composed by a few other *P. garbei* and unidentified *Neoplecostomus* species, in a sister relationship to a larger *S. guntheri* clade, while our *S. guntheri* appears embedded among other *S. guntheri* specimens from other regions in Brazil.

Of note, the pairwise nucleotide identity (PNI) variation encountered among three genera of the *Peckoltia*-clade (*Peckoltia*, *Ancistomus* and *Panaqolus*) is below the range of variation encountered within each of the two genera in its sister clade (Hypostomini), but above the variation between these genera (*Hypostomus* and *Pterygoplichthys*). This is also valid if we include the recently described mitochondrial genome of *Hypancistrus zebra* [11], another member of the *Peckoltia*-clade. However, the morphological diversity within the *Peckoltia*-clade exceeds by far the variation found among Hypostomini. Although the Hypostomini and *Peckoltia*-clade are both species-rich, most Hypostomini species are grouped into two large genera (*Hypostomus* and *Pterygoplichthys*) while the *Peckoltia*-clade is the most genus-rich Loricariidae tribe [7] and also harbors the worst taxonomic problems within this family [29].

The low divergence found among the mitochondrial genomes from the four genera (*H. zebra* included) belonging to the *Peckoltia*-clade is a piece of information that shall assist in the explanation and resolution of these long standing taxonomic problems. In addition, an intriguing question emerges from our results: How is the tremendous eco-morphological diversity characteristic of the Peckoltia-clade achieved with such a low genetic variation?

## Conclusions

Herein we have launched the use of high-throughput mitochondrial genomics in the studies of the Loricarioidei species, advancing the knowledge on the genetic diversity of Neotropical fish fauna. Nearly complete mitochondrial genomes were sequenced for 27 species, representing 21 Loricariidae and one Callichthyidae genera. These new resources greatly reduce the underrepresentation of Loricarioidei among the Siluriformes mitochondrial genomes deposited in GenBank and other public databases. However, the proportion of genetic data available for Siluroidei still outpaces that of Loricarioidei, especially with regard to nuclear genes.

## Methods

### Taxonomic sampling and RNA extraction

Twenty-seven species from 21 Loricariidae genera (representing six subfamilies) and one Callichthyidae genus, were sampled in the Amazon basin, and in coastal basins of southeastern Brazil, and acquired at local ornamental fish suppliers in the cities of Rio de Janeiro (Rio de Janeiro State) and Belém (Pará State), in Brazil (Table 2). Samples from the Amazon basin were collected near Manaus (Amazonas State); samples from coastal streams were collected in the states of Rio de Janeiro and Espírito Santo. Specimens were initially identified by experienced taxonomists; identifications were then confirmed by similarity searches using the cox1 Folmer region from each specimen against the BOLD Systems database (Additional file 7). Additionally, the complete and nearly complete mitochondrial genomes from Loricarioidei species available on GenBank were downloaded and used for comparative and phylogenetic purposes. GenBank accession numbers of all mitogenomes are displayed in Table 2. All voucher specimens were deposited at a permanent ichthyological collection (Museu Nacional, Universidade Federal do Rio de Janeiro - MNRJ) (Table 2). Fish sampling and handling were authorized by the appropriate Brazilian Government agency (Instituto Chico Mendes de Conservação da Biodiversidade, ICMBio), under the license number 21006-4.

Immediately after sampling, fish were killed, and liver dissected and preserved in RNA Later solution (Thermo Fisher Scientific) at 4 °C until arrival at the laboratory, where samples were stored at −20 °C. Total RNA was extracted from the liver tissue following the phenol-choloroform method, according to manufacture’s instructions (TRIzol Reagent, Thermo Fisher Scientific). After extraction, total RNA was initially quantified using a BioDrop DUO (BioDrop) spectrophotometer, and quality and quantity were further evaluated using the Bioanalyzer RNA 6000 nano kit (Agilent). Only RNA preparations with RNA Integrity Number (RIN) above 6.0 were used for the cDNA library preparation.

### Library preparation and sequencing

Complementary DNA (cDNA) libraries were prepared using the TruSeq RNA Sample Kit v.2 (Illumina), strictly following the manufacturer’s recommendations. The fragmentation time in the thermocycler was adjusted to 45 s to ensure that most fragment lengths were in the range of 300 to 500 base pairs. All libraries were accessed for quality using the Bioanalyzer DNA 1000 kit (Agilent). Library quantification was performed using the Library Quantification Universal Kit for Illumina with Revised Primers-SYBR (Kapa Biosystems) for real time PCR for each library, and once more for each pool of up to nine libraries, individually marked with specific barcodes, destined to clusterization in Illumina HiSeq2500 sequencing lanes. Library pools were clustered using the TrueSeq PE Cluster Kit v3 for cBot (Illumina) into different lanes. Paired-end sequencing of 100 bp were performed on a HiSeq2500 using the TrueSeq SBS Kit v.3 (Illumina). Raw data were demultiplexed using the BCL2FASTQ software (Illumina). Reads were trimmed for adaptors with Trimmomatic [30] and their quality was evaluated using FastQC (Babraham Bioinformatics). Reads with Phred score equal to or higher than 30 were used for *de novo* assembly of transcriptomes using Trinity’s default parameters [31, 32].

### Mitogenome assembly and annotation

The mitochondrial genomes were assembled using the mitochondrial transcripts retrieved from the liver transcriptome of each fish, following the approach described by Moreira et al. [8]. Briefly, searches using the BLASTn algorithm against the mitogenomes from the closest related species available were performed to capture mitochondrial transcripts among the entire transcriptome. The retrieved mitochondrial transcripts were aligned to the complete mitogenome available from the closest related species (*Pterygoplichthys disjunctivus* NC015747, *Hypoptopoma incognitum* NC028072 or *Ancistrus* sp. KP960567). The assembled mitochondrial genomes were manually edited for removal of poorly aligned bases at both ends. In most cases, some gaps remained in intergenic and tRNA regions due to the punctuation pattern of the mitochondrial transcript expression. These gaps were filled with Ns.

The assembled mitochondrial genomes were annotated using the web-based services MitoFish and MITOS [33, 34], and manually curated for inconsistencies. To estimate the sequencing depth of each base, Bowtie v. 1.0.0 was used to align Illumina reads onto the assembled mitogenomes. Aligned reads were viewed using IGV, Tablet, PysamStats (v. 0.84) and BAMStats (v. 1.25) [35–37]. Heteroplasmic sites were identified using three conditions: first, different nucleotides were sequenced in the same position; second, that position must have more than 100 supporting reads; and third, the frequency of the second most frequent base must be higher than 10%. Using these criteria, every polymorphic nucleotide is supported by at least 10 reads.

### Phylogenetic analysis

The sequences for the two rRNA and for the 13 protein-coding genes were recovered in the mitochondrial genomes from all species. The sequences of the 15 mitochondrial genes were aligned using the built-in MUSCLE algorithm from SeaView [38, 39]. Protein-coding gene sequences were aligned by their amino acid residues, and phylogenetic analyses were performed using the nucleotide information. Alignments of each gene were concatenated to create a single super-alignment consisting of 14,116 nucleotides. Pairwise nucleotide identity was calculated using SeaView and tabulated in R (R Core Team [40]).

jModelTest2 (v. 2.1.6) available at the CIPRES Science Gateway were used to select the best-fit model of nucleotide substitution under the Bayesian information criterion (BIC) [41–43]. The ML analysis was performed using RAxML (v. 8.2.4) available at CIPRES under the GTR + GAMMA + I model for all sites [44]. Branch support was evaluated with 1000 bootstrap replicates.

## Additional files

Additional file 1: Summary data about mitochondrial genome sequences produced in the study. Mitogenomes were assembled using transcriptomic data. The number of transcripts used to assemble each mitogenome is shown, as well as their lengths without gaps. The 100 bp paired end Illumina Hi-Seq2500 reads were mapped against the assembled mitochondrial genome. The number of total reads mapped, as well as the average and median sequencing depth, which estimates the number each nucleotide was sequenced, are also provided. The nucleotide usage for each mitochondrial genome is shown. (PDF 59 kb)

Additional file 2: Summary data about mitochondrial genome sequences produced in the study. Total length for each mitochondrial gene is given. For the protein coding genes, the used start and stop codons are shown. The complete 12S and 16S ribosomal RNA are highlighted in bold. (PDF 104 kb)

Additional file 3: Completeness of transfer RNAs sequencing. It is shown whether each of the 22 tRNA coded in the mitochondrial genome of 31 Loricarioidei species was sequenced to its complete length (complete), partially sequenced (partial) or not sequenced (not seq.). (PDF 65 kb)

Additional file 4: The mitochondrial control region highlighting the Conserved Sequence Blocks (CSB). Insertion/deletion mutations (indels) are shown as hyphens (−). Background is colored according to the nucleotide at each position, blue for T, red for A, yellow for G and green for C, or in white for indels. The positions of the CSB are delimited at the right bottom of the alignment. (PDF 376 kb)

Additional file 5: Heteroplasmic sites identified on the mitochondrial genomes of Loricarioidei catfishes. This spreadsheet file is sub-divided in 32 independent sheets; one containing the legend, another with a summary and one for each of the 31 Loricarioidei species, identified by their field numbers as detailed on Table 2 in the main text. The sheets for each species show every heteroplasmic position identified, the total number of supporting reads, as well as, for each nucleotide, the absolute count and its proportion. On the Summary sheet, each column contains all the heteroplasmic positions found for a single species (identified on the column head). The first column on the left shows the gene where this position is located. Genes coding for ribosomal RNAs are colored in green, while genes coding for transfer RNAs are colored in blue and protein coding genes are colored in orange. Lines represent homologous positions among the mitochondrial genomes from the different species. Three conditions were used to characterize a heteroplasmic site: first, different nucleotides were sequenced in the same position; second, that position must have more than 100 supporting reads; and third, the frequency of the second most frequent base must be higher than 10%. On the Summary sheet, the positions with more than 1000 supporting reads are highlighted in red. (XLSX 63 kb)

Additional file 6: Phylogenetic tree retrieved using the three longest genes (A) and the three genes with the best performance (B). The three longest genes, cox1, nad4 and nad5, also harbored most informative characters. A - The position of *Dekeyseria amazonica*, highlighted in a red box, changed in relation to the tree using the 15 mitochondrial genes. The three genes with best performance as identified by Havird & Santos [13] were nad2, nad4 and nad5. B - The position of *Loricaria cataphracta*, highlighted in a red box, changed in relation to the tree using the 15 mitochondrial genes. Genes were aligned with built-in MUSCLE using SeaView and used for phylogenetic tree reconstruction using RAxML under the GTR + GAMMA + I model and 1000 bootstrap replica. (PDF 62 kb)

Additional file 7: Confirmation of species identification using cox1 barcode sequence and the BOLD Systems. The Folmer region of Cytochrome c oxidase subunit 1 of each species was used as queries for similarity searches against the BOLD database. The resulting output is presented as a phylogenetic tree generated online. The query species are shown in red and indicated by a red arrow. For each tree, the species name, as well as its field and voucher numbers are shown above the arrow. (PDF 765 kb)

## Abbreviations

12S rRNA and 16S rRNA: 12S and 16S ribosomal RNA
AM: State of Amazonas
atp6 and atp8: ATPase subunit 6 and 8
BOLD: Barcode of Life Data
cDNA: Complementary DNA
cob: Cytochrome b
cox1–3: Cytochrome c oxidase subunits 1,2 and 3
CR: Mitochondrial control region
ES: State of Espírito Santo
ICMBio: Instituto Chico Mendes de Conservação da Biodiversidade
ML: Maximum likelihood
MNRJ: Museu Nacional, Universidade Federal do Rio de Janeiro
mTERF: Mitochondrial termination factor
nad1–6 and 4l: NADH dehydrogenase subunit 1–6 and 4l
O_L_: Origin of L-strand replication
PA: State of Pará
PNI: Pairwise nucleotide identity
RIN: RNA Integrity Number
RJ: State of Rio de Janeiro
TAS: Termination associated sequence
tRNA: Transfer RNA
