# Supplementary material for: Reducing the information gap on Loricarioidei (Siluriformes) mitochondrial genomics
Source: BMC Genomics. 2017 May 4;18:345. doi: 10.1186/s12864-017-3709-3 (PMC5418769; doi:10.1186/s12864-017-3709-3)
Supplement: Supplementary file 1 — Summary data about mitochondrial genome sequences produced in the study. Mitogenomes were assembled using transcriptomic data. The number of transcripts used to assemble each mitogenome is shown, as well as their lengths without gaps. The 100 bp paired end Illumina Hi-Seq2500 reads were mapped against the assembled mitochondrial genome. The number of total reads mapped, as well as the average and median sequencing depth, which estimates the number each nucleotide was sequenced, are also provided. The nucleotide usage for each mitochondrial genome is shown. (PDF 59 kb) [file 12864_2017_3709_MOESM1_ESM.pdf]

Additional file 1: Summary data about mitochondrial genome sequences produced in the study. Mitogenomes were assembled using transcriptomic data. The number of transcripts used to assemble each mitogenome is shown, as well as their lengths without gaps. The 100bp paired end Illumina Hi-Seq2500 reads were mapped against the assembled mitochondrial genome. The number of total reads mapped, as well as the average and median sequencing depth, which estimates the number each nucleotide was sequenced, are also provided. The nucleotide usage for each mitochondrial genome is shown.

Species	Number of transcripts used to assemble	Length without gaps	Reads mapped	Average sequencing depth	Median sequencing depth	Nucleotide frequencies					
						%A	%C	%G	%T	GC%	AT%
Hemipsilichthys nimius	11	15,784	1,869,528	10,923	4,418	29.7%	28.9%	15.5%	25.9%	44.4%	55.6%
Rineloricaria cf. *lanceolata*	8	16,154	906,705	5,438	1,495	31.8%	28.4%	14.8%	25.1%	43.2%	56.9%
Rineloricaria sp.	13	15,754	395,202	2,378	638	31.8%	27.0%	14.5%	26.6%	41.5%	58.4%
Loricariichthys platymetopon	7	15,778	1,245,347	7,460	1,671	31.2%	27.5%	15.0%	26.3%	42.5%	57.5%
Loricariichthys castaneus	10	15,254	4,921,826	28,772	9,518	31.4%	27.1%	14.6%	26.9%	41.7%	58.3%
Loricaria cataphracta	4	16,232	2,873,462	17,351	10,105	31.9%	26.9%	14.6%	26.5%	41.5%	58.4%
Otocinclus cf. *hoppei*	7	16,352	852,574	5,087	2,192	30.3%	29.3%	15.7%	24.7%	45.0%	55.0%
Hypoptopoma incognitum	5	16,630	895,334	5,293	1,715	29.3%	31.1%	16.3%	23.3%	47.4%	52.6%
Parotocinclus maculicauda	9	15,677	194,552	1,155	329	29.3%	30.3%	16.6%	23.8%	46.9%	53.1%
Hisonotus thayeri	9	15,868	673,875	4,012	915	29.8%	29.5%	16.2%	24.5%	45.7%	54.3%
Kronichthys heylandi	10	16,350	1,900,795	11,196	1,906	30.9%	29.2%	15.3%	24.6%	44.5%	55.5%
Neoplecostomini gen. n.	6	15,781	184,540	1,098	382	30.8%	28.5%	15.7%	25.0%	44.2%	55.8%
Neoplecostomus microps	7	15,923	618,371	3,676	861	30.6%	29.0%	15.6%	24.8%	44.6%	55.4%
Pareiorhaphis garbei	6	15,998	712,614	4,209	751	30.4%	29.9%	15.6%	24.1%	45.5%	54.5%
Schizolecis guntheri	9	15,061	350,081	2,076	555	29.0%	31.3%	16.5%	23.2%	47.8%	52.2%
Ancistrus sp. 1	13	16,396	1,408,010	8,351	2,572	30.9%	28.0%	15.1%	26.0%	43.1%	56.9%
Ancistrus sp. 2	7	15,644	711,472	4,245	1,545	30.2%	28.3%	15.7%	25.8%	44.0%	56.0%
Ancistrus multispinis	9	15,907	620,344	3,701	1,391	30.3%	27.9%	15.5%	26.3%	43.4%	56.6%
Dekeyseria amazonica	5	16,330	895,226	5,416	2,567	31.3%	28.1%	15.1%	25.6%	43.2%	56.9%
Baryancistrus xanthellus	9	16,368	575,810	3,459	554	31.2%	27.4%	15.0%	26.4%	42.4%	57.6%
Pterygoplichthys sp.	11	15,993	373,444	2,254	614	31.5%	26.9%	14.6%	26.9%	41.5%	58.4%
Pterygoplichthys pardalis	10	16,043	569,103	3,397	1,554	31.7%	27.1%	14.6%	26.7%	41.7%	58.4%
Pterygoplichthys disjunctivus	GenBank	16,521				31.8%	26.6%	14.6%	26.9%	41.2%	58.7%
Hypostomus sp.	10	15,769	314,982	1,895	338	31.5%	26.6%	14.4%	27.5%	41.0%	59.0%
Hypostomus cf. *plecostomus*	8	16,267	652,284	3,881	1,513	31.4%	26.9%	14.7%	27.0%	41.6%	58.4%
Hypostomus affinis	13	15,407	674,628	4,018	480	31.3%	26.7%	14.5%	27.5%	41.2%	58.8%
Aphanotorulus emarginatus	9	16,010	1,183,075	7,060	3,791	31.4%	26.6%	14.7%	27.2%	41.3%	58.6%
Peckoltia furcata	9	15,902	317,782	1,876	437	31.9%	26.7%	14.4%	27.0%	41.1%	58.9%
Ancistomus snethlageae	5	16,333	1,118,439	6,731	4,537	32.1%	26.3%	14.3%	27.3%	40.6%	59.4%
Panaqolus sp.	10	16,371	1,316,451	7,733	2,810	32.0%	26.6%	14.3%	27.2%	40.9%	59.2%
Corydoras nattereri	11	16,557	583,549	3,470	928	32.3%	27.0%	15.1%	25.6%	42.1%	57.9%
Corydoras schwartzi	7	16,517	3,134,364	18,237	4,988	32.9%	26.9%	14.5%	25.8%	41.4%	58.7%
Corydoras rabauti	GenBank	16,831				32.6%	26.6%	14.8%	26.0%	41.4%	58.6%
