# Supplementary material for: Reducing the information gap on Loricarioidei (Siluriformes) mitochondrial genomics
Source: BMC Genomics. 2017 May 4;18:345. doi: 10.1186/s12864-017-3709-3 (PMC5418769; doi:10.1186/s12864-017-3709-3)
Supplement: Supplementary file 2 — Summary data about mitochondrial genome sequences produced in the study. Total length for each mitochondrial gene is given. For the protein coding genes, the used start and stop codons are shown. The complete 12S and 16S ribosomal RNA are highlighted in bold. (PDF 104 kb) [file 12864_2017_3709_MOESM2_ESM.pdf]

Additional file 2: Summary data about mitochondrial genome sequences produced in the study. Total length for each mitochondrial gene is given. For the protein coding genes, the used start and stop codons are shown. The complete 12S and 16S ribosomal RNA are highlighted in bold.

Species	12S rRNA	16S rRNA	nad1			nad2			cox1			cox2			atp8			atp6		
Hemipsilichthys nimius	**950**	1661	972	ATG	TAG	1045	ATG	T--	1551	GTG	TAA	691	ATG	T--	168	ATG	TAA	683	GTG	TA-
Rineloricaria cf. *lanceolata*	940	1660	972	ATG	TAA	1045	ATG	T--	1551	GTG	TAA	691	ATG	T--	165	ATG	TAA	683	ATG	TA-
Rineloricaria sp.	945	**1665**	972	ATG	TAA	1045	ATG	T--	1551	GTG	TAA	691	ATG	T--	165	ATG	TAA	683	ATG	TA-
Loricariichthys platymetopon	**948**	**1665**	972	ATG	TAG	1045	ATG	T--	1551	GTG	TAA	691	ATG	T--	165	ATG	TAA	683	ATG	TA-
Loricariichthys castaneus	**948**	**1669**	972	ATG	TAA	1045	ATG	T--	1551	GTG	TAA	691	ATG	T--	165	ATG	TAA	683	ATG	TA-
Loricaria cataphracta	927	1655	969	ATG	TAA	1045	ATG	T--	1551	GTG	TAA	691	ATG	T--	168	ATG	TAA	683	ATG	TA-
Otocinclus cf. *hoppei*	**952**	**1675**	975	ATG	TAA	1045	ATG	T--	1551	GTG	TAA	691	ATG	T--	168	ATG	TAA	683	ATG	TA-
Hypoptopoma incognitum	**957**	**1677**	975	ATG	TAG	1045	ATG	T--	1551	GTG	TAA	691	ATG	T--	168	ATG	TAA	683	ATG	TA-
Parotocinclus maculicauda	919	1664	975	ATG	TAA	1045	ATG	T--	1551	GTG	TAA	691	ATG	T--	168	ATG	TAA	683	ATG	TA-
Hisonotus thayeri	931	**1679**	975	ATG	TAA	1045	ATG	T--	1550	GTG	TAA	691	ATG	T--	168	ATG	TAA	683	ATG	TA-
Kronichthys heylandi	937	**1672**	975	ATG	TAA	1045	ATG	T--	1551	GTG	TA	691	ATG	T--	168	ATG	TAA	683	ATG	TA-
Neoplecostomini gen. n.	845	1648	957	-	TAG	1045	ATG	T--	1551	GTG	TAA	691	ATG	T--	168	ATG	TAA	683	ATG	TA-
Neoplecostomus microps	943	**1673**	975	ATG	TAA	1033	-	T--	1551	GTG	TAA	604	-	T--	168	ATG	TAA	683	ATG	TA-
Pareiorhaphis garbei	933	**1680**	975	ATG	TAG	1045	ATG	T--	1551	GTG	TAA	691	ATG	T--	168	ATG	TAA	683	ATG	TA-
Schizolecis guntheri	897	1636	975	ATG	TAA	1045	ATG	T--	1550	GTG	TA	691	ATG	T--	168	ATG	TAA	683	ATG	TA-
Ancistrus sp. 1	**950**	**1672**	972	ATG	TAA	1045	ATG	T--	1551	GTG	TAA	691	ATG	T--	168	ATG	TAA	683	ATG	TA-
Ancistrus sp. 2	939	**1672**	972	ATG	TAA	1045	ATG	T--	1551	GTG	TAA	691	ATG	T--	168	ATG	TAA	683	ATG	TA-
Ancistrus multispinis	924	**1670**	975	ATG	TAA	1045	ATG	T--	1551	GTG	TAA	691	ATG	T--	168	ATG	TAA	683	ATG	TA-
Dekeyseria amazonica	**950**	**1677**	975	ATG	TAA	937	-	T--	1551	GTG	TAA	691	ATG	T--	168	ATG	TAA	683	ATG	TA-
Baryancistrus xanthellus	951	1663	975	ATG	TAA	1045	ATG	T--	1551	GTG	TAA	691	ATG	T--	168	ATG	TAA	683	ATG	TA-
Pterygoplichthys sp.	**954**	**1676**	975	ATG	TAA	1045	ATG	T--	1551	GTG	TAA	691	ATG	T--	168	ATG	TAA	683	ATG	TA-
Pterygoplichthys pardalis	920	1669	975	ATG	TAA	1045	ATG	T--	1551	GTG	TAA	691	ATG	T--	168	ATG	TAA	683	ATG	TA-
Pterygoplichthys disjunctivus	**955**	**1676**	975	ATG	TAA	1045	ATG	T--	1551	GTG	TAA	691	ATG	T--	168	ATG	TAA	683	ATG	TA-
Hypostomus sp.	934	1651	975	ATG	TAA	1045	ATG	T--	1551	GTG	TAA	691	ATG	T--	168	ATG	TAA	683	ATG	TA-
Hypostomus cf. *plecostomus*	**954**	**1678**	975	ATG	TAA	1045	ATG	T--	1551	GTG	TAA	691	ATG	T--	168	ATG	TAA	683	ATG	TA-
Hypostomus affinis	922	1661	975	ATG	TAA	1045	ATG	T--	1551	GTG	TAA	691	ATG	T--	168	ATG	TAA	683	ATG	TA-
Aphanotorulus emarginatus	914	**1678**	975	ATG	TAA	1045	ATG	T--	1551	GTG	TAA	691	ATG	T--	168	ATG	TAA	683	ATG	TA-
Peckoltia furcata	922	1669	975	ATG	TAA	1045	ATG	T--	1551	GTG	TAA	641	ATG	-	168	ATG	TAA	683	ATG	TA-
Ancistomus snethlageae	937	**1674**	975	ATG	TAA	1045	ATG	T--	1551	GTG	TAA	691	ATG	T--	168	ATG	TAA	683	ATG	TA-
Panaqolus sp.	941	**1675**	975	ATG	TAA	1045	ATG	T--	1551	GTG	TAA	691	ATG	T--	168	ATG	TAA	683	ATG	TA-
Corydoras nattereri	**945**	**1669**	972	ATG	TAG	1045	ATG	T--	1560	GTG	AGG	691	ATG	T--	168	ATG	TAA	684	ATG	TAA
Corydoras schwartzi	**946**	**1672**	972	ATG	TAG	1045	ATG	T--	1560	GTG	AGG	682	-	T--	168	ATG	TAA	684	ATG	TAA
Corydoras rabauti	**946**	**1669**	972	ATG	TAG	1045	ATG	T--	1560	GTG	AGG	691	ATG	T--	168	ATG	TAA	684	ATG	TAA

Species		cox3			nad3			nad4l			nad4			nad5			nad6			cyt b	
Hemipsilichthys nimius	784	ATG	T--	349	ATG	T--	297	ATG	TAA	1381	ATG	T--	1827	ATG	TAG	522	ATG	TAA	1138	ATG	T--
Rineloricaria cf. *lanceolata*	784	ATG	T--	349	ATG	T--	297	ATG	TAA	1381	ATG	T--	1827	ATG	TAA	519	ATG	TAG	1134	ATG	TAG
Rineloricaria sp.	784	ATG	T--	349	ATG	T--	297	ATG	TAA	1381	ATG	T--	1827	ATG	TAA	519	ATG	TAG	1134	ATG	TAA
Loricariichthys platymetopon	784	ATG	T--	349	ATG	T--	297	ATG	TAA	1381	ATG	T--	1827	ATG	TAA	519	ATG	TAA	1134	ATG	TAA
Loricariichthys castaneus	784	ATG	T--	349	ATG	T--	297	ATG	TAA	1381	ATG	T--	1827	ATG	TAA	519	ATG	TAA	1134	ATG	TAA
Loricaria cataphracta	784	ATG	T--	349	ATG	T--	297	ATG	TAA	1381	ATG	T--	1827	ATG	TAA	519	ATG	TAA	1134	ATG	TAA
Otocinclus cf. *hoppei*	784	ATG	T--	349	ATG	T--	297	ATG	TAA	1381	ATG	T--	1827	ATG	TAA	522	ATG	TAG	1138	ATG	T--
Hypoptopoma incognitum	784	ATG	T--	349	ATG	T--	297	ATG	TAA	1381	ATG	T--	1827	ATG	TAA	522	ATG	TAG	1138	ATG	T--
Parotocinclus maculicauda	784	ATG	T--	334	-	T--	297	ATG	TAA	1381	ATG	T--	1827	ATG	TAA	522	ATG	TAG	1138	ATG	T--
Hisonotus thayeri	784	ATG	T--	349	ATG	T--	297	ATG	TAA	1381	ATG	T--	1827	ATG	TAA	522	ATG	TAG	1138	ATG	T--
Kronichthys heylandi	784	ATG	T--	349	ATG	T--	297	ATG	TAA	1381	ATG	T--	1827	GTG	TAA	522	ATG	TAG	1138	ATG	T--
Neoplecostomini gen. n.	784	ATG	T--	346	ATG	T--	297	ATG	TAA	1381	ATG	T--	1827	ATG	TAA	522	ATG	TAG	1138	ATG	T--
Neoplecostomus microps	784	ATG	T--	340	-	T--	297	ATG	TAA	1379	ATG	-	1827	ATG	TAA	522	ATG	TAG	1138	ATG	T--
Pareiorhaphis garbei	772	-	T--	349	ATG	T--	297	ATG	TAA	1381	ATG	T--	1827	ATG	TAA	522	ATG	TAG	1138	ATG	T--
Schizolecis guntheri	784	ATG	T--	349	ATG	T--	297	ATG	TAA	1381	ATG	T--	1824	ATG	TAA	519	ATG	TAG	1138	ATG	T--
Ancistrus sp. 1	772	-	T--	349	ATG	T--	297	ATG	TAA	1381	ATG	T--	1827	ATG	TAA	522	ATG	TAA	1134	ATG	TAA
Ancistrus sp. 2	784	ATG	T--	349	ATG	T--	297	ATG	TAA	1381	ATG	T--	1827	ATG	TAA	522	ATG	TAA	1134	ATG	TAA
Ancistrus multispinis	778	-	T--	349	ATG	T--	297	ATG	TAA	1381	ATG	T--	1827	ATG	TAA	522	ATG	TAA	1134	ATG	TAA
Dekeyseria amazonica	784	ATG	T--	349	ATG	T--	297	ATG	TAA	1381	ATG	T--	1827	ATG	TAA	522	ATG	TAG	1134	ATG	TAA
Baryancistrus xanthellus	784	ATG	T--	349	ATG	T--	297	ATG	TAA	1381	ATG	T--	1827	ATG	TAA	522	ATG	TAG	1138	ATG	T--
Pterygoplichthys sp.	775	-	T--	349	ATG	T--	297	ATG	TAA	1381	ATG	T--	1815	-	TAA	522	ATG	TAG	1132	-	T--
Pterygoplichthys pardalis	784	ATG	T--	349	ATG	T--	297	ATG	TAA	1381	ATG	T--	1827	ATG	TAA	522	ATG	TAG	1135	-	T--
Pterygoplichthys disjunctivus	784	ATG	T--	349	ATG	T--	297	ATG	TAA	1381	ATG	T--	1827	ATG	TAA	522	ATG	TAG	1138	ATG	T--
Hypostomus sp.	784	ATG	T--	349	ATG	T--	291	-	TAA	1381	ATG	T--	1812	-	TAA	522	ATG	TAG	1107	ATG	-
Hypostomus cf. *plecostomus*	784	ATG	T--	349	ATG	T--	297	ATG	TAA	1381	ATG	T--	1827	ATG	TAA	522	ATG	TAG	1136	ATG	-
Hypostomus affinis	772	-	T--	349	ATG	T--	297	ATG	TAA	1381	ATG	T--	1827	ATG	TAA	522	ATG	TAG	1094	-	-
Aphanotorulus emarginatus	784	ATG	T--	349	ATG	T--	297	ATG	TAA	1381	ATG	T--	1827	ATG	TAA	522	ATG	TAG	1138	ATG	T--
Peckoltia furcata	784	ATG	T--	349	ATG	T--	297	ATG	TAA	1381	ATG	T--	1827	ATG	TAA	522	ATG	TAA	1138	ATG	T--
Ancistomus snethlageae	784	ATG	T--	349	ATG	T--	297	ATG	TAA	1381	ATG	T--	1827	ATG	TAA	522	ATG	TAG	1138	ATG	T--
Panaqolus sp.	784	ATG	T--	349	ATG	T--	297	ATG	TAA	1381	ATG	T--	1827	ATG	TAA	522	ATG	TAG	1138	ATG	T--
Corydoras nattereri	784	ATG	T--	349	ATG	T--	297	ATG	TAA	1381	ATG	T--	1827	ATG	TAA	516	ATG	TAA	1138	ATG	T--
Corydoras schwartzi	784	ATG	T--	349	ATG	T--	297	ATG	TAA	1381	ATG	T--	1827	ATG	TAA	516	ATG	TAG	1138	ATG	T--
Corydoras rabauti	784	ATG	T--	349	ATG	T--	297	ATG	TAA	1381	ATG	T--	1827	ATG	TAA	516	ATG	TAA	1138	ATG	T--
