# Supplementary material for: Reducing the information gap on Loricarioidei (Siluriformes) mitochondrial genomics
Source: BMC Genomics. 2017 May 4;18:345. doi: 10.1186/s12864-017-3709-3 (PMC5418769; doi:10.1186/s12864-017-3709-3)
Supplement: Supplementary file 3 — Completeness of transfer RNAs sequencing. It is shown whether each of the 22 tRNA coded in the mitochondrial genome of 31 Loricarioidei species was sequenced to its complete length (complete), partially sequenced (partial) or not sequenced (not seq.). (PDF 65 kb) [file 12864_2017_3709_MOESM3_ESM.pdf]

Additional file 3: Completeness of transfer RNAs sequencing. It is shown whether each of the 22 tRNA coded in to mitochondrial genome of 31 Loricarioidei species was sequenced to its complete length (complete), partially sequenced (partial) or not sequenced (not seq.).

**Species**	**tRNA-Phe**	**tRNA-Val**	**tRNA-Leu2**	**tRNA-Ile**
Hemipsilichthys nimius	partial	complete	partial	complete
Rineloricaria cf. *lanceolata*	not seq.	complete	partial	complete
Rineloricaria sp.	not seq.	complete	partial	complete
Loricariichthys platymetopon	partial	complete	complete	complete
Loricariichthys castaneus	partial	partial	complete	complete
Loricaria cataphracta	partial	not seq.	complete	complete
Otocinclus cf. *hoppei*	complete	complete	partial	not seq.
Hypoptopoma incognitum	complete	complete	complete	complete
Parotocinclus maculicauda	not seq.	not seq.	partial	complete
Hisonotus thayeri	not seq.	not seq.	partial	complete
Kronichthys heylandi	complete	partial	complete	complete
Neoplecostomini gen. n.	not seq.	not seq.	not seq.	complete
Neoplecostomus microps	not seq.	complete	complete	complete
Pareiorhaphis garbei	complete	not seq.	complete	complete
Schizolecis guntheri	not seq.	partial	partial	complete
Ancistrus sp. 1	partial	complete	partial	complete
Ancistrus sp. 2	not seq.	complete	complete	complete
Ancistrus multispinis	not seq.	partial	complete	not seq.
Dekeyseria amazonica	partial	complete	complete	complete
Baryancistrus xanthellus	complete	not seq.	partial	complete
Pterygoplichthys sp.	complete	complete	partial	complete
Pterygoplichthys pardalis	complete	not seq.	partial	complete
Hypostomus sp.	complete	not seq.	partial	not seq.
Hypostomus cf. *plecostomus*	complete	partial	partial	complete
Hypostomus affinis	not seq.	not seq.	partial	not seq.
Aphanotorulus emarginatus	partial	not seq.	partial	not seq.
Peckoltia furcata	partial	not seq.	complete	not seq.
Ancistomus snethlageae	not seq.	complete	complete	complete
Panaqolus sp.	not seq.	complete	complete	complete
Corydoras nattereri	complete	complete	complete	complete
Corydoras schwartzi	partial	partial	complete	complete
**complete**	10	14	15	25
**partial**	9	6	15	0
**not sequenced**	12	11	1	6

tRNA-Gln	tRNA-Met	tRNA-Trp	tRNA-Ala	tRNA-Asn	tRNA-Cys	tRNA-Tyr
complete	complete	complete	complete	complete	complete	complete
complete	complete	not seq.	partial	complete	complete	complete
complete	complete	complete	complete	complete	complete	complete
complete	complete	complete	complete	complete	complete	complete
complete	complete	not seq.	not seq.	not seq.	not seq.	partial
complete	complete	complete	complete	complete	complete	complete
partial	complete	complete	complete	complete	complete	complete
complete	complete	complete	complete	complete	complete	complete
partial	not seq.	complete	complete	complete	complete	complete
complete	complete	complete	complete	complete	complete	complete
partial	partial	not seq.	complete	complete	complete	complete
complete	complete	complete	complete	complete	complete	complete
complete	partial	complete	complete	complete	complete	complete
complete	complete	complete	complete	complete	complete	complete
complete	complete	complete	complete	complete	complete	complete
complete	complete	complete	complete	complete	complete	complete
complete	complete	complete	complete	complete	complete	complete
partial	complete	complete	complete	complete	complete	complete
complete	complete	complete	complete	complete	complete	complete
complete	complete	complete	complete	complete	complete	complete
complete	complete	partial	complete	complete	complete	complete
partial	partial	complete	complete	complete	complete	complete
partial	complete	complete	complete	complete	complete	complete
complete	complete	complete	complete	complete	complete	complete
not seq.	not seq.	not seq.	not seq.	not seq.	not seq.	not seq.
not seq.	complete	complete	complete	complete	complete	complete
partial	complete	complete	complete	complete	complete	complete
complete	complete	complete	complete	complete	complete	complete
complete	complete	partial	partial	complete	complete	complete
complete	complete	complete	complete	complete	complete	complete
complete	complete	complete	complete	complete	complete	complete
22	26	25	27	29	29	29
7	3	2	2	0	0	1
2	2	4	2	2	2	1

tRNA-Ser2	tRNA-Asp	tRNA-Lys	tRNA-Gly	tRNA-Arg	tRNA-His	tRNA-Ser1
complete	partial	complete	complete	complete	not seq.	not seq.
complete	not seq.	complete	complete	complete	complete	complete
complete	not seq.	complete	complete	complete	not seq.	partial
complete	complete	complete	complete	complete	complete	complete
complete	not seq.	complete	complete	complete	complete	complete
complete	complete	complete	complete	complete	complete	complete
complete	complete	complete	complete	complete	complete	complete
complete	complete	complete	complete	complete	complete	complete
complete	partial	complete	not seq.	complete	not seq.	not seq.
complete	complete	complete	complete	complete	complete	complete
complete	complete	complete	partial	complete	not seq.	not seq.
complete	complete	complete	complete	complete	complete	complete
complete	not seq.	complete	not seq.	complete	not seq.	not seq.
complete	complete	complete	complete	complete	not seq.	not seq.
complete	partial	complete	complete	complete	not seq.	not seq.
complete	complete	complete	complete	complete	not seq.	partial
complete	complete	complete	complete	complete	complete	complete
complete	complete	complete	partial	complete	complete	complete
complete	complete	complete	complete	complete	complete	complete
complete	complete	complete	complete	complete	partial	complete
complete	complete	complete	partial	partial	not seq.	not seq.
complete	complete	complete	complete	complete	complete	complete
complete	complete	partial	partial	partial	not seq.	not seq.
complete	complete	complete	complete	complete	complete	complete
complete	complete	complete	complete	complete	complete	complete
complete	complete	complete	complete	complete	complete	complete
complete	complete	partial	complete	complete	partial	complete
complete	complete	complete	complete	complete	complete	complete
complete	complete	complete	complete	complete	complete	complete
complete	complete	complete	complete	complete	complete	complete
complete	not seq.	complete	complete	complete	complete	partial
31	23	29	25	29	19	20
0	3	2	4	2	2	3
0	5	0	2	0	10	8

tRNA-Leu1	tRNA-Glu	tRNA-Thr	tRNA-Pro	complete	partial	not sequenced
partial	complete	not seq.	not seq.	14	4	4
complete	complete	complete	complete	17	2	3
complete	complete	not seq.	not seq.	15	2	5
complete	complete	complete	partial	20	2	0
complete	complete	complete	complete	14	3	5
complete	complete	complete	partial	19	2	1
complete	complete	not seq.	not seq.	17	2	3
complete	complete	complete	complete	22	0	0
partial	complete	complete	complete	12	4	6
complete	complete	not seq.	not seq.	17	1	4
partial	complete	complete	complete	14	5	3
complete	complete	complete	complete	19	0	3
partial	complete	not seq.	partial	13	3	6
partial	complete	complete	complete	18	1	3
not seq.	complete	not seq.	not seq.	13	3	6
complete	complete	complete	complete	18	3	1
complete	complete	complete	complete	21	0	1
complete	complete	partial	complete	16	4	2
complete	complete	complete	complete	21	1	0
complete	complete	partial	complete	18	3	1
not seq.	complete	not seq.	not seq.	13	4	5
complete	partial	not seq.	not seq.	15	4	3
not seq.	complete	not seq.	not seq.	10	5	7
complete	complete	not seq.	not seq.	18	2	2
complete	partial	not seq.	not seq.	8	2	12
complete	complete	not seq.	not seq.	15	2	5
complete	complete	not seq.	partial	14	5	3
complete	complete	complete	complete	21	0	1
complete	complete	complete	complete	19	2	1
complete	complete	complete	complete	22	0	0
complete	complete	complete	complete	18	3	1
23	29	16	16	**complete**		
5	2	2	4		**partial**	
3	0	13	11			**not sequenced**
