# Supplementary material for: Reducing the information gap on Loricarioidei (Siluriformes) mitochondrial genomics
Source: BMC Genomics. 2017 May 4;18:345. doi: 10.1186/s12864-017-3709-3 (PMC5418769; doi:10.1186/s12864-017-3709-3)
Supplement: Supplementary file 4 — The mitochondrial control region highlighting the Conserved Sequence Blocks (CSB). Insertion/deletion mutations (indels) are shown as hyphens (−). Background is colored according to the nucleotide at each position, blue for T, red for A, yellow for G and green for C, or in white for indels. The positions of the CSB are delimited at the right bottom of the alignment. (PDF 376 kb) [file 12864_2017_3709_MOESM4_ESM.pdf]

Additional file 4: The mitochondrial control region highlighting the Conserved Sequence Blocks (CSB). Insertion/deletion mutations (indels) are shown as hyphens (-). Background is colored according to the nucleotide at each position, blue for T, red for A, yellow for G and green for C, or in white for indels. The positions of the Conserved Sequence Blocks (CSB) are delimited at the right bottom of the alignment.

```
                                1
Corydoras schwartzi             CGATATACAGTATTACATGT AATAACACAACTAATGTTTC AACACCAAGTACAGCACGCA CAACATATGTTTAATACCAT
Corydoras nattereri             -------------------- -------------------- ---TTGTATATAGTACTGCC CAACACATATACAACACATA
Hemipsilichthys nimius          -------------------- -------------------- -------------------- --------------------
Rineloricaria cf. lanceolata    -------------------- -------------------- -------------------- --------------------
Rineloricaria sp.               -------------------- -------------------- -------------------- --------------------
Loricariichthys platymetopon    -------------------- -------------------- -------------------- --------------------
Loricariichthys castaneus       -------------------- -------------------- -------------------- --------------------
Loricaria cataphracta           -------------------- -------------------- -------------------- --------------------
Otocinclus cf. hoppei           -------------------- -------------------- -------------------- --------------------
Hypoptopoma incognitum          -------------------- -------------------- -------------------- --------------------
Parotocinclus maculicauda       -------------------- -------------------- -------------------- --------------------
Hisonotus thayeri               -------------------- -------------------- -------------------- --------------------
Kronichthys heylandi            -------------------- -------------------- -------------------- --------------------
Neoplecostomini gen. n.         -------------------- -------------------- -------------------- --------------------
Neoplecostomus microps          -------------------- -------------------- -------------------- --------------------
Pareiorhaphis garbei            -------------------- --------------AGTGCC TATATATAGCCCACCTATAG TACAATACATATATTACATT
Schizolecis guntheri            -------------------- -------------------- -------------------- --------------------
Ancistrus sp. 1                 ---CAGCACACACGTATACA CCACAAGTGCAATACATACA CCATATTATAGTATAACACA TGACATATGTACACTACGTA
Ancistrus sp. 2                 -------------------- -------------------- -------------------- --------------------
Ancistrus multispinis           -------------------- -------------------- -------------------- --------------------
Dekeyseria amazonica            -------------------- -------------------- -------------------- --------------------
Baryancistrus xanthellus        -------------------- -------AACATGTATATAG TACATTATGGTGTTAATACA TATTATGTATATAGTACATT
Pterygoplichthys sp.            -------------------- -------------------- -------------------- --------------------
Pterygoplichthys pardalis       -------------------- -------------------- -------------------- --------------------
Hypostomus sp.                  -------------------- -------------------- -------------------- --------------------
Hypostomus cf. plecostomus      -------------------- -------------------- -------------------- --------------------
Hypostomus affinis              -------------------- -------------------- -------------------- -------------CTAACTC
Aphanotorulus emarginatus       -------------------- -------------------- -------------------- --------------------
Peckoltia furcata               -------------------- -------------------- -------------------- --------------------
Ancistomus snethlageae          -------------------- -------------------- -------------------- --------------------
Panaqolus sp.                   -------------------- -------AGCATGTATATAT TACATTATGGTGTTAATACA TATTATGTATATATTACATT
P.disjunctivus_NC015747         -------------------- -----------------TAT TACATTATGGTATTAATACA TATTATGTATATATTACATT
```

```
                                 81
Corydoras schwartzi              AAATAGGTACTAAACACGTA CGCGTGATATTGTATGCATG TATGTAC-TAAGTACAT-AA TATGTATAATAT-----TAC
Corydoras nattereri              TGTCAATAATT--------- -------------------- TACATAC-CTTGTACATGTA CATATGTATTAAGTACATAC
Hemipsilichthys nimius           -------------------- -------------------- -------------------- --------------------
Rineloricaria cf. lanceolata     -------------------- -------------------- CACATATGGTATAATACATA CCTATGTACTAC-----CTC
Rineloricaria sp.                -------------------- -------------------- -------------------- ------------------AT
Loricariichthys platymetopon     -------------------- -------------------- -------------------- --------------------
Loricariichthys castaneus        -------------------- -------------------- -------------------- CT---GCAACAA-----TAT
Loricaria cataphracta            -------------------- -------------------- -------------------- -------------------C
Otocinclus cf. hoppei            -------------------- -------------------- -ATGTAT-ATAATACAT-AT TATGTATAATTA-----TAC
Hypoptopoma incognitum           ---TGAGTAGTACATAT--- -------------------- TATGTAT-ATAGTATAT-TA T-------------------
Parotocinclus maculicauda        -------------------- ---------AACCGTACGCC CCATAAT-ATAGTACATATA TGCATGGGG--------TAC
Hisonotus thayeri                -------------------- -------------------- -------------------- --------ATAA-----TGC
Kronichthys heylandi             -------------------- -------------------- -------ATCATCACTCATG GGTACACACCACAAGAGTAC
Neoplecostomini gen. n.          -------------------- -------------------- -------------------- --------ACAA-----TGC
Neoplecostomus microps           -------------------- -------------------- --------ATAAT------- -----------------TAC
Pareiorhaphis garbei             ATGGTACTAATACATATATT ACATTATGGTACT------- AATACAT-ATATTACAT-TA TG---GTACTAA-----TAC
Schizolecis guntheri             -------------------- -------------------- -------------------- --------------------
Ancistrus sp. 1                  ATGGTGTTAATACATAA--- -------------------- TATGTAT-ATATTACAT-AA TG---GTGTTAA-----TAC
Ancistrus sp. 2                  -------------------- -------------------- -------------------- ---------CAA-----GGC
Ancistrus multispinis            --------------CGA--- -------------------- GATGCAC-ATATTACAG-TA TG---GTGTTAA-----TGC
Dekeyseria amazonica             ----------------C--- -------------------- AACGTCA-ACACTTCAC-TA TG---GTGTTAA-----TGC
Baryancistrus xanthellus         ATGGTGTTAATACATAT--- -------------------- TATGTAT-ATAGTACAT-TA TG---GTGTTAA-----TAC
Pterygoplichthys sp.             ----------TACATAT--- -------------------- TATGTAT-ATATTACAT-TA TG---GTATTAA-----TAC
Pterygoplichthys pardalis        -------------------- -------------------- -------------------- --------------------
Hypostomus sp.                   ----------TACATAT--- -------------------- TATGTAT-ATATTACAT-TA TG---GTATTAA-----TAC
Hypostomus cf. plecostomus       -------------------- -------------------- -------------------- --------------------
Hypostomus affinis               CCACCCCTAACTCCCA---- -------------------- AAGCTAGGATTCTAAACTAA ACTATTTTCTGA--------
Aphanotorulus emarginatus        ----TTCTAACTCCCAC--- -------------------- CCCTAAC-TCCCAAAGC-TA GG---ATTCTAA-----ATT
Peckoltia furcata                -------------------- -------------------- -------------------- -----------------GTC
Ancistomus snethlageae           ---------------AT--- -------------------- CATTATA-ATATTACAT-TA TG---GTATTAA-----TAC
Panaqolus sp.                    ATGGTGTTAATACATAT--- -------------------- TATGTAT-ATATTACAT-TA TG---GTGTTAA-----TAC
P.disjunctivus_NC015747          ATGGTATTAATACATAT--- -------------------- TATGTAT-ATATTACAT-TA TG---GTATTAA-----TAC
```

```
                                  161
Corydoras schwartzi               ATATAT---------ATATG TATTAA---------GTACA TTATATGTATAA-------T ATTACATATATATATGTATT
Corydoras nattereri               ATATATGTACTAAGTACATA CATGTA---------TAATA TTACATACATATATGTACCA AATACATATA----------
Hemipsilichthys nimius            -------------------- -------------------- -------------------- --------------------
Rineloricaria cf. lanceolata      ATACATTATGATATAGTACA TATATATGCATTATCTTACA TTATG---ATAT-------- AGTACATATA----------
Rineloricaria sp.                 ATA----------------- -------------------- -------------------- --------------------
Loricariichthys platymetopon      -------------------- -------------------- -------------------- --------------------
Loricariichthys castaneus         ATA----------------- -------------------- ----G---GGTA-------T AGTATATA------------
Loricaria cataphracta             ATA----------------- ---ATA---------TTACA TTATG---GTAT-------- AGTACATAT-----------
Otocinclus cf. hoppei             ATA---------------TA TGTATA-------------- -------------------T AATACATAT-----------
Hypoptopoma incognitum            -------------------G TATATA---------GTACA TAATG---GTAT-------- AGTACATAC-----------
Parotocinclus maculicauda         ATA------------TTATG CATATA---------GTAC- --------ATATATGCATGG GGTACATAT-----------
Hisonotus thayeri                 ATG----------------- -------------------- -------------------G GGTACATAT-----------
Kronichthys heylandi              ATATGTATGACACATAAATG TATATA---------CTACA TTAT---------------T GGTACATACATA--------
Neoplecostomini gen. n.           ACC----------------- -------------------- -----------T-------G AGCACATAG-----------
Neoplecostomus microps            ACA----------------- CATGTA---------TTACA CTATG---GTGT-------- AATTCATAT-----------
Pareiorhaphis garbei              ATATATTACATTATGGTATG TATATA---------TTACA TTATG---GTAC-------T AATACATAT-----------
Schizolecis guntheri              -------------------- -------------------- -------------------- --------------------
Ancistrus sp. 1                   ATA------------ATATG TATATA---------TTACA TAATG---GTGT-------T AATACATAA-----------
Ancistrus sp. 2                   ACA----------------- ----------------CGCA CTAGG---ACAT-------- AATACATAA-----------
Ancistrus multispinis             ATA------------ATGTG TATATA---------TTACA TTATG---GTAT-------T AATACATAA-----------
Dekeyseria amazonica              ATA--TT------------- -GTGTA---------TTATA TTATG---ATAT-------T AATACATAT-----------
Baryancistrus xanthellus          ATA------------TTATG TATATA---------GTACA TTATG---GTGT-------T AATACATAT-----------
Pterygoplichthys sp.              ATA------------TTATG TATATA---------TTACA TTATG---GTAT-------T AATACATAT-----------
Pterygoplichthys pardalis         -------------------- -------------------- -------------------- --TACATAT-----------
Hypostomus sp.                    ATA------------TTATG TATATA---------TTACA TTATG---GTAT-------T AATACATAT-----------
Hypostomus cf. plecostomus        -------------------- -------------------- -------------------- --TACATAT-----------
Hypostomus affinis                ------------------TG TATATA---------TTACA TTATG---GTAT-------T AATACATAT-----------
Aphanotorulus emarginatus         AAA--CTATTTTCTGAGCAA TGTACA---------TTACA CTATG---GTAT-------T AATGCATAT-----------
Peckoltia furcata                 GTA----------------- TGTACA---------TTACA CTATG---GTGT-------T AATGCATAT-----------
Ancistomus snethlageae            ATA------------TTATG TATATA---------TTACA TTATG---GTAT-------T AATACATAT-----------
Panaqolus sp.                     ATA------------TTATG TATATA---------TTACA TTATG---GTGT-------T AATACATAT-----------
P.disjunctivus_NC015747           ATA------------TTATG TATATA---------TTACA TTATG---GTAT-------T AATACATAT-----------
```

```
                                 241
Corydoras schwartzi              AAGTACATTATATGTATAAT ATTACATATATA-GTATAAT ATTACATATATATATGTGCT AAAT----ACATAA-T-ATG
Corydoras nattereri              ----------CATGTATAAT ATTACATATATATGTA---- ----------------TT-- AAAT----ACATAA-T-ATG
Hemipsilichthys nimius           -------------------- -------------------- -------------------- --------------------
Rineloricaria cf. lanceolata     ----------TATGCATTAT CTTACAT-TATG-ATA---- ----------------T--- -AGT----ACATATAC-ATA
Rineloricaria sp.                ----------TATGTATTAT CTTACATTAATTATTGGTCC TCAAATAATTAACCAATT-- -AATCAGGACATAA-A-TTG
Loricariichthys platymetopon     -------------------- -------------------- -------------------- --------------------
Loricariichthys castaneus        ----------TATGCACTAT ATTACAT-TATG-GTA---- ----------------T--- -AGT----AC----------
Loricaria cataphracta            ----------TATGCATAAT ATTACAT-TATA-ATG---- ----------------CTTT AAAT----AATTAA-T-ATG
Otocinclus cf. hoppei            ----------TATGCATAAT TATACATATATGCATG---- ----------------T--- -AAT----ACATATATGGTG
Hypoptopoma incognitum           ----------TATGTAT-AT -------------------- -------------------- -AGT----ACATAA-T-ATG
Parotocinclus maculicauda        ----------TATGCAT-AT AGTACATATATGCATG---- ------------------G- -GGT----ACATAT-T-ATG
Hisonotus thayeri                ----------TATGCAT-AT AGTACAT-TATG-GTA---- ----------------TA-- -TCC----CCACAT-C-ATG
Kronichthys heylandi             ----------TATGTAC-GT AATGCACGTATGTAT----- ----------------TT-- -AAT----ACATAT-T-ATG
Neoplecostomini gen. n.          ----------TATGTAC--- -------------------- -----------------T-- -AGT----ACATAT-T-ATG
Neoplecostomus microps           ----------TATGCGT--- GGTACACGTATGTAT----- ----------------TT-- -AAT----ACATAT-T-ATG
Pareiorhaphis garbei             ----------TATGTAT-AT ATTACAT-TATG-GTA---- ----------------CT-- -AAT----ACATAT-T-ATG
Schizolecis guntheri             -------------------- -------------------- -------------------- --------------------
Ancistrus sp. 1                  ----------TATGTAT-AT ATTACAT-AATG-GTG---- ----------------TT-- -AAT----ACATAA-T-ATG
Ancistrus sp. 2                  ----------TATGCAT--T AGTACAT-TATG-GTG---- ----------------TT-- -AAT----ACATAA-T-ATG
Ancistrus multispinis            ----------TATGCAT-AT ATTACAT-TATG-GTG---- ----------------TT-- -AAT----ACATAA-T-ATG
Dekeyseria amazonica             ----------TATGTAT-AT ATTACAT-TATG-GTA---- ----------------TT-- -AAT----ACATAC-T-ATG
Baryancistrus xanthellus         ----------TATGTAT-AT AGTACAT-TATG-GTG---- ----------------TT-- -AAT----ACATAT-T-ATG
Pterygoplichthys sp.             ----------TATGTAT-AT ATTACAT-TATG-GTA---- ----------------TT-- -AAT----ACATAT-T-ATG
Pterygoplichthys pardalis        ----------TATGTAT-AT ATTACAT-TATG-GTA---- ----------------TT-- -AAT----ACATAT-T-ATG
Hypostomus sp.                   ----------TATGTAT-AT ATTACAT-TATG-GTA---- ----------------TT-- -AAT----ACATAT-T-ATG
Hypostomus cf. plecostomus       ----------TATGTAT-AT ATTACAT-TATG-GTA---- ----------------TT-- -AAT----ACATAT-T-ATG
Hypostomus affinis               ----------TATGTAT-AT ATTACAT-TATG-GTG---- ----------------TT-- -AAT----ACATAC-T-ATG
Aphanotorulus emarginatus        ----------TATGTAT-AT ATTACAT-TATG-GTG---- ----------------TT-- -AAT----ACATAC-T-ATG
Peckoltia furcata                ----------TATGTAT--- ---------ATG-GTG---- ----------------T--- -AGT----ACATAT-T-ATG
Ancistomus snethlageae           ----------TATGTAT-AT ATTACAT-TATG-GTA---- ----------------TT-- -AAT----ACATAT-T-ATG
Panaqolus sp.                    ----------TATGTAT-AT ATTACAT-TATG-GTG---- ----------------TT-- -AAT----ACATAT-T-ATG
P.disjunctivus_NC015747          ----------TATGTAT-AT ATTACAT-TATG-GTA---- ----------------TT-- -AAT----ACATAT-T-ATG
```

```
                                321
Corydoras schwartzi             TATAATATTGCATACATTTA TATTACCCATTCATAATGTA AGAGTAATTAGAATAAATTC ATCAAGACATACATTTATAT
Corydoras nattereri             TATAATATTACATACATATA TTTTACCCATACATTATGTA CCAGTAACGAATATAAACTC ATCAAAACACAAAATTATAT
Hemipsilichthys nimius          -------------------- -------------------- -------------------- --------------------
Rineloricaria cf. lanceolata    CAT-ATATTACATAATTTCA TGGTCC-------------- -------------------- -------------------T
Rineloricaria sp.               AAG-ATAAAACAATAAATTA A------------------- -------------------- --------------------
Loricariichthys platymetopon    -------------------- -------------------- -------------------- --------------------
Loricariichthys castaneus       -------------------- -------------------- -------------------- --------------------
Loricaria cataphracta           AAT----------------- -------------------- -------------------- -----------------TGG
Otocinclus cf. hoppei           TAT----CCCCATTAATAGA T------------------- -------------------- -------------------A
Hypoptopoma incognitum          TAT-TTAATGCATTATGATA TATC---------------- -------------------- -------CCCATGACCTGCT
Parotocinclus maculicauda       CAT-ATAGTACATTATGGTA TATCCCCATTTCACGA---- -------------------- -------------------T
Hisonotus thayeri               A------------------- -------------------- -------------------- -------------------T
Kronichthys heylandi            TAT-ATAGTACAT-ATGATA TATCCCCATCTCCTGA---- -------------------- -------------------T
Neoplecostomini gen. n.         TA------------------ -------------------- -------------------- -------------------C
Neoplecostomus microps          TAT-ATAGTACAT-ATGATA TATCCCCATCTCCTGA---- -------------------- -------------------A
Pareiorhaphis garbei            TAT-ATATTACATTATGGTA TATCCTCATTTCAAGG---- -------------------- -------------------T
Schizolecis guntheri            -------------------- -------------------- -------------------- --------------------
Ancistrus sp. 1                 TAT-ATACCCCATATTACTG T------------------- -------------------- -------------------T
Ancistrus sp. 2                 TAT-ATAGTACATTA----- -------------------- -------------------- --------------------
Ancistrus multispinis           TAT-AATCCCCATATTACTG T------------------- -------------------- -------------------T
Dekeyseria amazonica            TAT-AATCCCCATAAAATTA T------------------- -------------------- -------------------A
Baryancistrus xanthellus        TAT-ATAGTACATTAATTTA T------------------- -------------------- -------------------C
Pterygoplichthys sp.            TAT-ATATTACATCCTCCTA T------------------- -------------------- -------------------T
Pterygoplichthys pardalis       TAT-ATATTACATCTCTCTA T------------------- -------------------- -------------------T
Hypostomus sp.                  TAT-ATATTACATCTCCTTA T------------------- -------------------- -------------------T
Hypostomus cf. plecostomus      TAT-ATATTACATCTCCCTA T------------------- -------------------- -------------------T
Hypostomus affinis              TAT-ATATTGCATCACCTTA T------------------- -------------------- -------------------T
Aphanotorulus emarginatus       TAT-ATATTGCATCACTCTA T------------------- -------------------- -------------------T
Peckoltia furcata               TAT-ATATTACATTACTCGG T------------------- -------------------- -------------------T
Ancistomus snethlageae          TAT-ATATTACATTACTCGG T------------------- -------------------- -------------------T
Panaqolus sp.                   TAT-ATATTACATTACTCGA T------------------- -------------------- -------------------T
P.disjunctivus_NC015747         TAT-ATATTACATCTCTCTA T------------------- -------------------- -------------------T
```

```
                                401
Corydoras schwartzi             TCTAGAAATAATTTCTTTAG ACCCGGGAAATAGATTTATC CCCATAACTGCCCTCAAACA TTTTCTATGAAGTATA-ACT
Corydoras nattereri             TCTACAAATAATTTCTTTAA AGCTGGGAAATAGATTCATC CCCATAACTGCCTTCAACCA TTTTCTATGAAGCTTA-ACT
Hemipsilichthys nimius          -------------------- -------------------- -------------------- --------------------
Rineloricaria cf. lanceolata    CAATAATTTAATTAATTAAT TAAAACTAAATTGAAAATAC -------------AACAATA TATTAATTTAACCATCTATT
Rineloricaria sp.               -------------------- -------------------- -------------TTCAACC -----------------ATT
Loricariichthys platymetopon    -------------------- -------------------- -------------------- --------------------
Loricariichthys castaneus       -------------------- -------------------- -------------------- --------------------
Loricaria cataphracta           TCAAGACA------------ -------------------- -------------TTAAATT TAACTTACCAATAA---ATT
Otocinclus cf. hoppei           TAAAGGC-TATAACCTTGA- -------------------- -------------CTCACCT ACTTCTATTCATATCC-AAA
Hypoptopoma incognitum          TTTAATGTTAAATTTTTAA- -------------------- -------------TTCAAC- TATTTTATTGAACACA-GTT
Parotocinclus maculicauda       TTAATTGCTAAACCCTCGA- -------------------- -------------CTCAAC- TATCTTGTTCAATCGT-GTT
Hisonotus thayeri               TTAATTGTTAAACCCTAGA- -------------------- -------------CTCAGC- TATCTTGTTCAATAGT-ATT
Kronichthys heylandi            TTAATTGTTAAACCCTTGA- -------------------- -------------TTCAAC- TAACTTGTTCGGTAAATATC
Neoplecostomini gen. n.         TATATGCATAAT-------- -------------------- -------------------- --------------------
Neoplecostomus microps          TTAGTCGTTAAACCCTTAA- -------------------- -------------CTCAAC- TAACTTGTTCGATAAATGTT
Pareiorhaphis garbei            TTAATTATTAAACCCTTGA- -------------------- -------------TTCAAC- TATCTTATTCAATAAATACC
Schizolecis guntheri            -------------------- -------------------- -------------------- --------------------
Ancistrus sp. 1                 TAAAGGCAT--TCCCTTGA- -------------------- -------------TTCAACT TATT--ATTAAGGTCA-ACA
Ancistrus sp. 2                 -------------------- -------------------- -------------------- --------------------
Ancistrus multispinis           TAAAGGCAT--TCCCTTGA- -------------------- -------------TTCAACT TATT--ATTAAGGTCA-ACA
Dekeyseria amazonica            TAATGGCATATCTTTATGA- -------------------- -------------TTTAAAG -----------------ATT
Baryancistrus xanthellus        TAAAGGCATAATTTCTTGA- -------------------- -------------GTTGACA TATCTAGTTAAATAGA-ATT
Pterygoplichthys sp.            TAAAGGCATAATCTCTTGA- -------------------- -------------CTCAACA TATCTAGTTGTTCCCCAATT
Pterygoplichthys pardalis       TAAAGGCATAATCTCTTGA- -------------------- -------------CTCAACA TACCTAGTTATTTACC-ATT
Hypostomus sp.                  TAAAGGCATAATCTCTTGA- -------------------- -------------CTCAACA TACCTAGTTAAGAACA-ATT
Hypostomus cf. plecostomus      TAAAGGCATAATCTCTTGA- -------------------- -------------CTCAACA TACCTAGTTAAGCTCA-ATT
Hypostomus affinis              TAAAGGCATAATCTCTTGA- -------------------- -------------CTCAACA TACCTAGTTAAGAACA-ATT
Aphanotorulus emarginatus       TAAAGGCATAATCTCTTGA- -------------------- -------------CTCAACA TACCTAGTTAAAAACA-ATT
Peckoltia furcata               TAAAGGCATAATCTCTTGA- -------------------- -------------CTCAACA TATCTAGTTAAGAACA-ATT
Ancistomus snethlageae          TAAAGGCATAGTCTCTTGA- -------------------- -------------CTCAACA TATCTAGTTAAGTACA-ATT
Panaqolus sp.                   TAAAGGCATAATCTCTTGA- -------------------- -------------CTCAACA TACCTAGTTAAGAACA-ATT
P.disjunctivus_NC015747         TAAAGGCATAATCTCTTGA- -------------------- -------------CTCAACA TACCTAGTTATTTACC-ATT
```

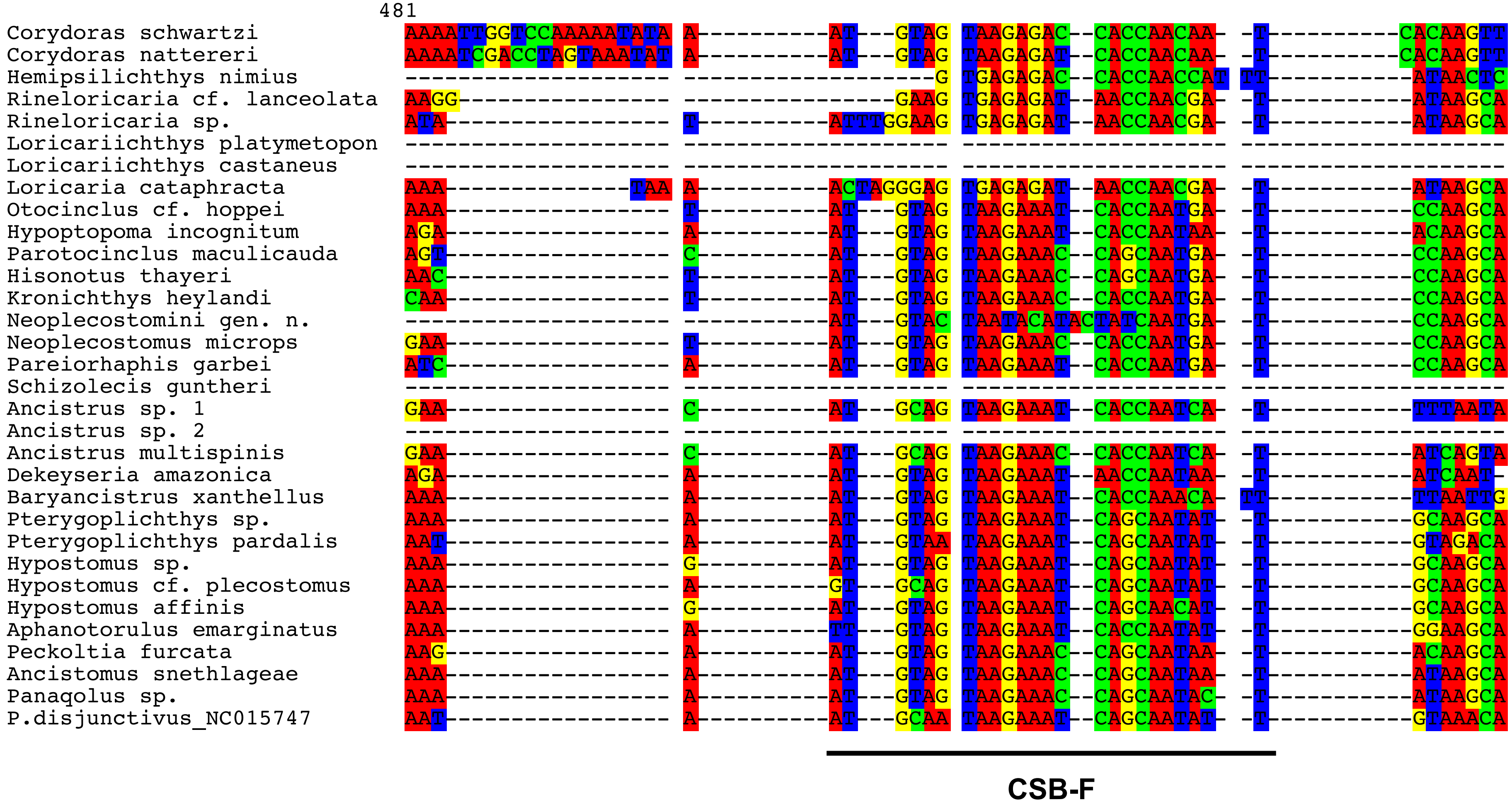
481
Corydoras schwartzi
Corydoras nattereri
Hemipsilichthys nimius
Rineloricaria cf. lanceolata
Rineloricaria sp.
Loricariichthys platymetopon
Loricariichthys castaneus
Loricaria cataphracta
Otocinclus cf. hoppei
Hypoptopoma incognitum
Parotocinclus maculicauda
Hisonotus thayeri
Kronichthys heylandi
Neoplecostomini gen. n.
Neoplecostomus microps
Pareiorhaphis garbei
Schizolecis guntheri
Ancistrus sp. 1
Ancistrus sp. 2
Ancistrus multispinis
Dekeyseria amazonica
Baryancistrus xanthellus
Pterygoplichthys sp.
Pterygoplichthys pardalis
Hypostomus sp.
Hypostomus cf. plecostomus
Hypostomus affinis
Aphanotorulus emarginatus
Peckoltia furcata
Ancistomus snethlageae
Panaqolus sp.
P.disjunctivus_NC015747
CSB-F

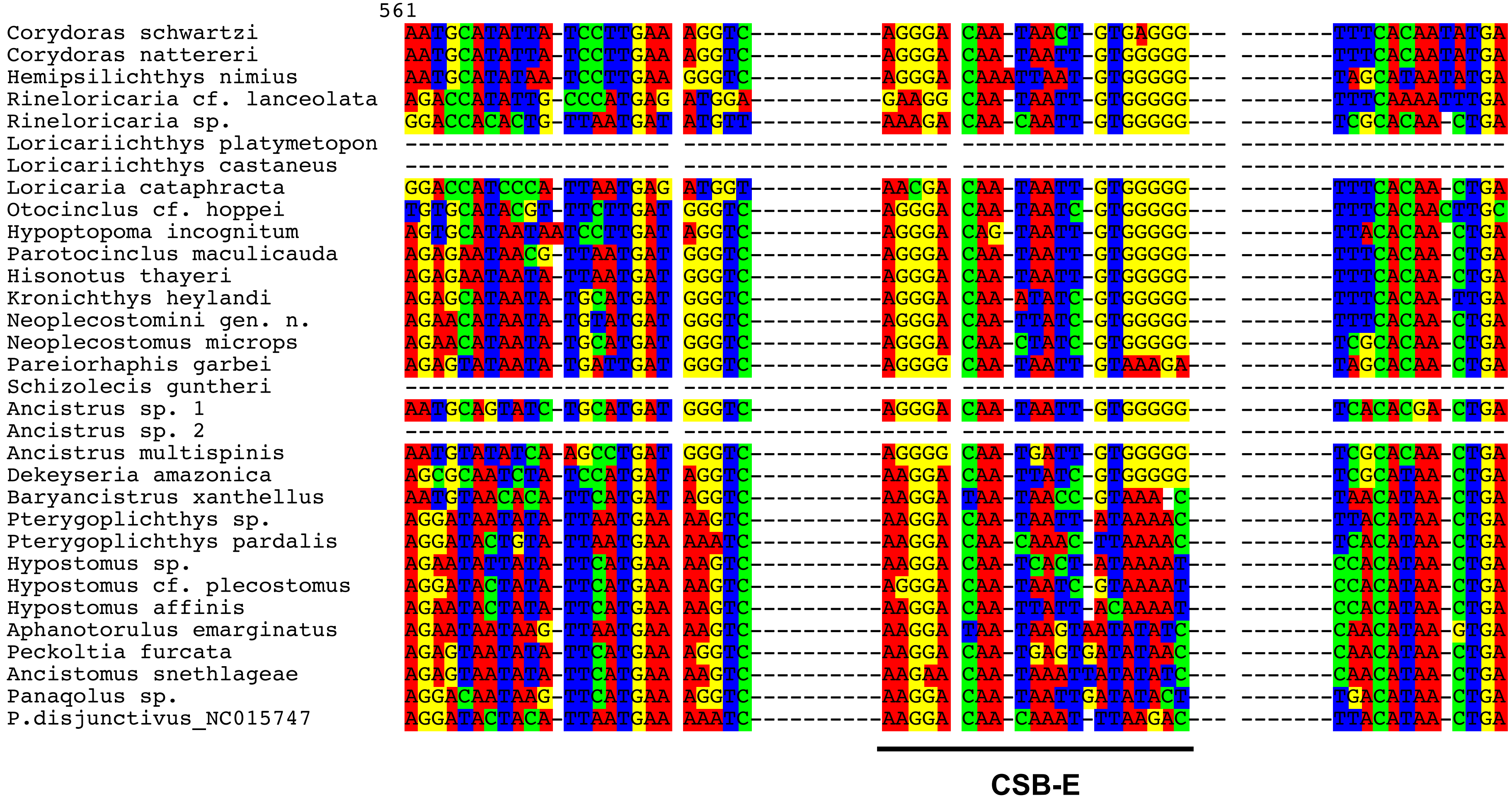
561
Corydoras schwartzi AATGCATATTA-TCCTTGAA AGGTC----------AGGGA CAA-TAACT-GTGAGGG--- -------TTTCACAATATGA
Corydoras nattereri AATGCATATTA-TCCTTGAA AGGTC----------AGGGA CAA-TAATT-GTGGGGG--- -------TTTCACAATATGA
Hemipsilichthys nimius AATGCATATAA-TCCTTGAA GGGTC----------AGGGA CAAATTAAT-GTGGGGG--- -------TAGCATAATATGA
Rineloricaria cf. lanceolata AGACCATATTG-CCCATGAG ATGGA----------GAAGG CAA-TAATT-GTGGGGG--- -------TTTCAAAATTTGA
Rineloricaria sp. GGACCACACTG-TTAATGAT ATGTT----------AAAGA CAA-CAATT-GTGGGGG--- -------TCGCACAA-CTGA
Loricariichthys platymetopon -------------------- -------------------- -------------------- --------------------
Loricariichthys castaneus -------------------- -------------------- -------------------- --------------------
Loricaria cataphracta GGACCATCCCA-TTAATGAG ATGGT----------AACGA CAA-TAATT-GTGGGGG--- -------TTTCACAA-CTGA
Otocinclus cf. hoppei TGTGCATACGT-TTCTTGAT GGGTC----------AGGGA CAA-TAATC-GTGGGGG--- -------TTTCACAACTTGC
Hypoptopoma incognitum AGTGCATAATAATCCTTGAT AGGTC----------AGGGA CAG-TAATT-GTGGGGG--- -------TTACACAA-CTGA
Parotocinclus maculicauda AGAGAATAACG-TTAATGAT GGGTC----------AGGGA CAA-TAATT-GTGGGGG--- -------TTTCACAA-CTGA
Hisonotus thayeri AGAGAATAATA-TTAATGAT GGGTC----------AGGGA CAA-TAATT-GTGGGGG--- -------TTTCACAA-CTGA
Kronichthys heylandi AGAGCATAATA-TGCATGAT GGGTC----------AGGGA CAA-ATATC-GTGGGGG--- -------TTTCACAA-TTGA
Neoplecostomini gen. n. AGAACATAATA-TGTATGAT GGGTC----------AGGGA CAA-TTATC-GTGGGGG--- -------TTTCACAA-CTGA
Neoplecostomus microps AGAACATAATA-TGCATGAT GGGTC----------AGGGA CAA-CTATC-GTGGGGG--- -------TCGCACAA-CTGA
Pareiorhaphis garbei AGAGTATAATA-TGATTGAT GGGTC----------AGGGG CAA-TAATT-GTAAAGA--- -------TAGCACAA-CTGA
Schizolecis guntheri -------------------- -------------------- -------------------- --------------------
Ancistrus sp. 1 AATGCAGTATC-TGCATGAT GGGTC----------AGGGA CAA-TAATT-GTGGGGG--- -------TCACACGA-CTGA
Ancistrus sp. 2 -------------------- -------------------- -------------------- --------------------
Ancistrus multispinis AATGTATATCA-AGCCTGAT GGGTC----------AGGGG CAA-TGATT-GTGGGGG--- -------TCGCACAA-CTGA
Dekeyseria amazonica AGCGCAATCTA-TCCATGAT AGGTC----------AAGGA CAA-TTATC-GTGGGGG--- -------TCGCATAA-CTGA
Baryancistrus xanthellus AATGTAACACA-TTCATGAT AGGTC----------AAGGA TAA-TAACC-GTAAA-C--- -------TAACATAA-CTGA
Pterygoplichthys sp. AGGATAATATA-TTAATGAA AAGTC----------AAGGA CAA-TAATT-ATAAAAC--- -------TTACATAA-CTGA
Pterygoplichthys pardalis AGGATACTGTA-TTAATGAA AAATC----------AAGGA CAA-CAAAC-TTAAAAC--- -------TCACATAA-CTGA
Hypostomus sp. AGAATATTATA-TTCATGAA AAGTC----------AAGGA CAA-TCACT-ATAAAAT--- -------CCACATAA-CTGA
Hypostomus cf. plecostomus AGGATACTATA-TTCATGAA AAGTC----------AGGGA CAA-TAATC-GTAAAAT--- -------CCACATAA-CTGA
Hypostomus affinis AGAATACTATA-TTCATGAA AAGTC----------AAGGA CAA-TTATT-ACAAAAT--- -------CCACATAA-CTGA
Aphanotorulus emarginatus AGAATAATAAG-TTAATGAA AAGTC----------AAGGA TAA-TAAGTAATATATC--- -------CAACATAA-GTGA
Peckoltia furcata AGAGTAATATA-TTCATGAA AGGTC----------AAGGA CAA-TGAGTGATATAAC--- -------CAACATAA-CTGA
Ancistomus snethlageae AGAGTAATATA-TTCATGAA AAGTC----------AAGAA CAA-TAAATTATATATC--- -------CAACATAA-CTGA
Panaqolus sp. AGGACAATAAG-TTCATGAA AGGTC----------AAGGA CAA-TAATTGATATACT--- -------TGACATAA-CTGA
P.disjunctivus_NC015747 AGGATACTACA-TTAATGAA AAATC----------AAGGA CAA-CAAAT-TTAAGAC--- -------TTACATAA-CTGA
CSB-E

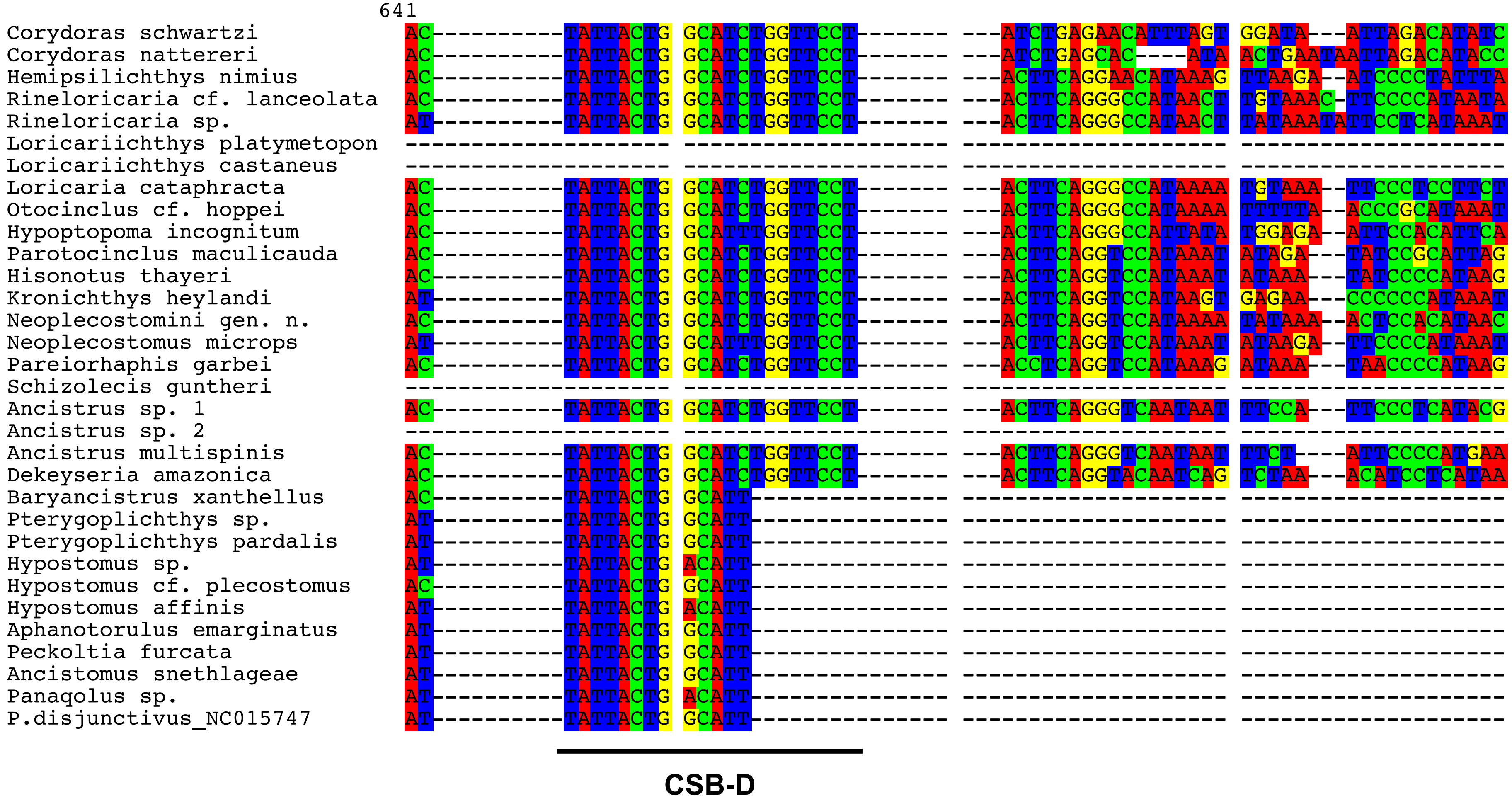
641
Corydoras schwartzi
Corydoras nattereri
Hemipsilichthys nimius
Rineloricaria cf. lanceolata
Rineloricaria sp.
Loricariichthys platymetopon
Loricariichthys castaneus
Loricaria cataphracta
Otocinclus cf. hoppei
Hypoptopoma incognitum
Parotocinclus maculicauda
Hisonotus thayeri
Kronichthys heylandi
Neoplecostomini gen. n.
Neoplecostomus microps
Pareiorhaphis garbei
Schizolecis guntheri
Ancistrus sp. 1
Ancistrus sp. 2
Ancistrus multispinis
Dekeyseria amazonica
Baryancistrus xanthellus
Pterygoplichthys sp.
Pterygoplichthys pardalis
Hypostomus sp.
Hypostomus cf. plecostomus
Hypostomus affinis
Aphanotorulus emarginatus
Peckoltia furcata
Ancistomus snethlageae
Panaqolus sp.
P.disjunctivus_NC015747
CSB-D

```
                                721
Corydoras schwartzi             TCACATCGTCAACGACATC- -----TGATTATTGGTGTAG ---TATAAAATAGCACAAAC CCACCATGCCGA-GCATTCT
Corydoras nattereri             TTACATCGTCAACGACATA- ------GATTATTGGTGGGG ---TTCAAAATAGCACAAAC CCACCATGCCGA-GCATTCT
Hemipsilichthys nimius          ATGTATCGTCAACGGCATC- -----TGATTAATGGTGTAG -TCCATTTCATATCAT-GAC CCACCATGCCAAGGCATTCT
Rineloricaria cf. lanceolata    ATCCATCGTCAACGGCATAT GGTTGTGATTAATGGTGTTA ACCTAATAAAT-CCAT-GAC CCACCATGCCAAGGCGTTCT
Rineloricaria sp.               ATCCATCGTCAACGGCATCT GATTGTGATTG-TGGTGGAG GACTGATAGT--CCAT-GAC CCACCATGCCAAGGCGTTCT
Loricariichthys platymetopon    -------------------- -------------------- -------------------- --------------------
Loricariichthys castaneus       -------------------- -------------------- -------------------- --------------------
Loricaria cataphracta           ATTCATCGTCAACGGCATT- -----TGATTAATGGTGTTA -ACTTGTAAA-ATCAT-AAC CCAACATGCCAAGGCATTCT
Otocinclus cf. hoppei           ATCCATCGTCAACGACATC- -----TGATTAATGGTGTAG -ACCTGTTGATATAAT-AAC CCACCATGCCAAGGCATTCT
Hypoptopoma incognitum          GTCCACCGTCAACGACATT- -----TGATTATTGGTGTAG -TCTCGAGTATATCAT-GAC CCACCATGCCAAGGCATTCT
Parotocinclus maculicauda       TTCCATCGTCAACGACATC- -----TGATTATTGGTGTAG -TCTCGAGTATATCAT-AAC CCAACATGCCAAGGCGTTCT
Hisonotus thayeri               TTCCATCGTCAACGGCATC- -----TGATTATTGGTGTAG -TCTCGAGTATATCAT-GAC CCACCATGCCAAGGCGTTCT
Kronichthys heylandi            ATCCATCGTCAACGGCATA- -----TGATTATTGGTGTAG -TCTCGAGTATATCAT-GAC CCACCATGCCAAGGCGTTCT
Neoplecostomini gen. n.         ATGCATCGTCAACGGCATC- -----TGATTAATGGTGTGA -CCCAAATTATATCAT-GAC TCACCATGCCAAGGCATTCT
Neoplecostomus microps          ATCCATCGTCAACGGCATT- -----TGATTATTGGTGTAG -TCTCGATTATATAAT-AAC CCCCCATGCCAAGGCGTTCT
Pareiorhaphis garbei            TTCCATCGTAAACGGCATC- -----TGATTATTGGTGTAA -CCCTATAAATATCAT-GAC CCCCCATGCCAAGGCGTTCA
Schizolecis guntheri            -------------------- -------------------- -------------------- --------------------
Ancistrus sp. 1                 TTCCACCGTCAACGACATC- -----TGATTATTGGTGTAA -CCCTGATAAT-CCAT-GAC TCACCATGCCAAGGCGTTCA
Ancistrus sp. 2                 -------------------- ------------TGGTGTTA -------------------- --------------------
Ancistrus multispinis           CTCCACCGTCAACGGCATC- -----TGATTATTGGTGTTA CCCTTAATAAT-CCAT-GAC TCACCATGCCAAGGCGTTCA
Dekeyseria amazonica            CTGTATCGTCAACGACATT- -----TGATTATTGGTGTTA ACTT--GAAATAGCACAAAC TCACCATG-CAAAGCATTCT
Baryancistrus xanthellus        -------------------- -----TGATTATTGGTGTCA ACCTTAAAAA--TCAT-AAC CCAACATGCCAAGGCATTCT
Pterygoplichthys sp.            -------------------- -----TGATTATTGGTTTAA -CCCTGTAAATATCAT-GAC TCACCATGCCAAGGCATTCT
Pterygoplichthys pardalis       -------------------- -----TGATTATTGGTTTCA -TCCTATAAATATAAT-GAC TCACCATGCCAAGGCATTCT
Hypostomus sp.                  -------------------- -----TGATTATTGATTTAA GTCCTGTAAATATCAT-AAC TCACCATGCCAAGGCATTCT
Hypostomus cf. plecostomus      -------------------- -----TGATTATTGCTTTAA GTCCTGTAAATATCAT-GAC TCACCATGCCAAGGCATTCT
Hypostomus affinis              -------------------- -----TGATTATTGATTTAA GTCCTGTAAATATCAT-GAC TCACCATGCCAAGGCATTCT
Aphanotorulus emarginatus       -------------------- -----TGATTATTGGTGTTG ACCTTATTAA-ATCAT-AAC TCACCATGCCAAGGCATTCT
Peckoltia furcata               -------------------- -----TGATTATTGGTGTAA ACCTTATCAA-ACCAT-GAC CCCCCATGCCAAGGCATTCT
Ancistomus snethlageae          -------------------- -----TGATTATTGGTGTAT ACCTTATTAA-ATCAT-AAC TCACCATGCCAAGGCATTCT
Panaqolus sp.                   -------------------- -----TGATTATTGGTGTAA ACCTTATTAA-ATCAT-GAC TCACCATGCCAAGGCATTCT
P.disjunctivus_NC015747         -------------------- -----TGATTATTGGTTTTA -TCCTGTAAATATAAT-GAC TCACCATGCCAAGGCATTCT
```

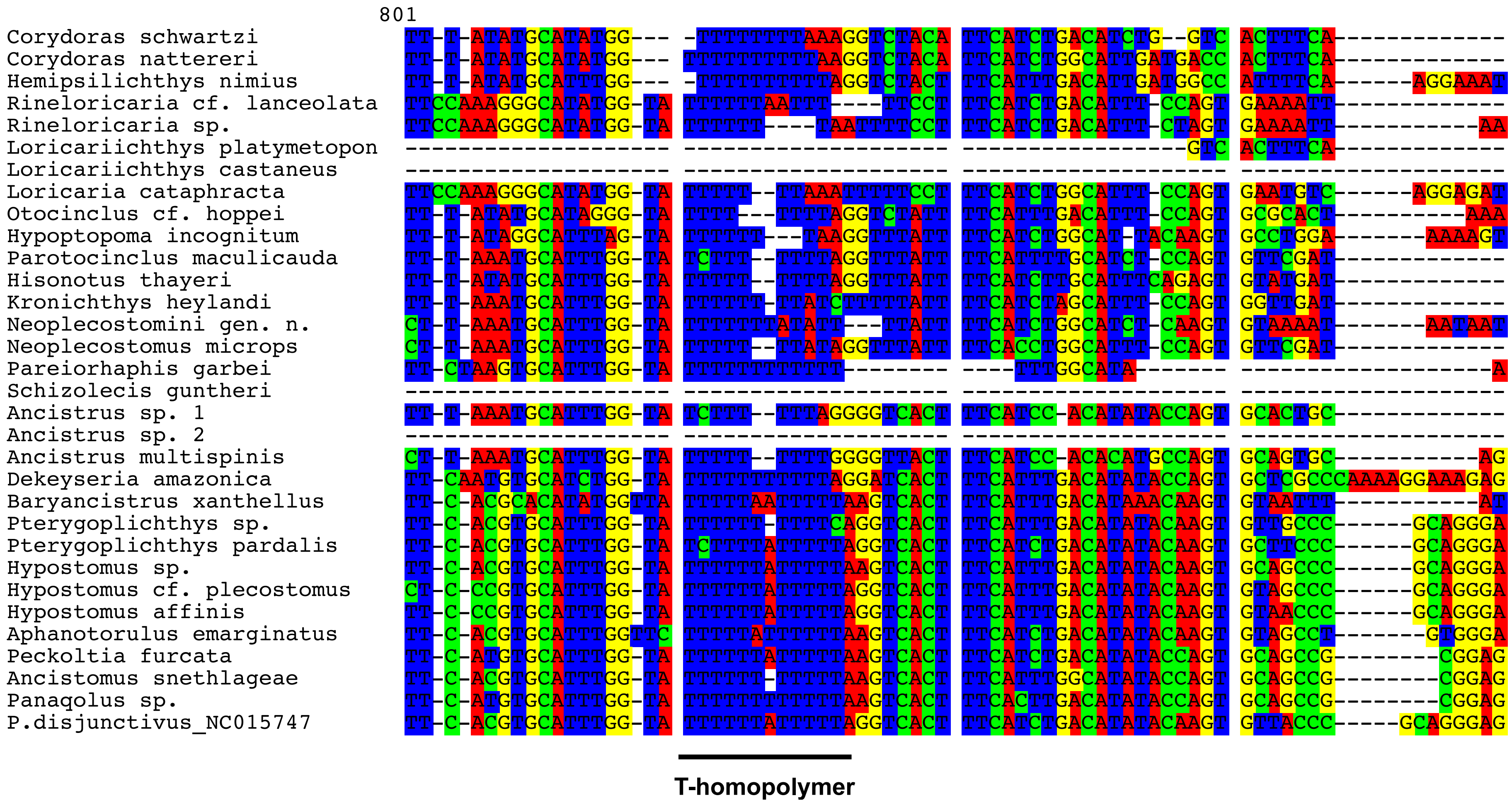
801
Corydoras schwartzi
Corydoras nattereri
Hemipsilichthys nimius
Rineloricaria cf. lanceolata
Rineloricaria sp.
Loricariichthys platymetopon
Loricariichthys castaneus
Loricaria cataphracta
Otocinclus cf. hoppei
Hypoptopoma incognitum
Parotocinclus maculicauda
Hisonotus thayeri
Kronichthys heylandi
Neoplecostomini gen. n.
Neoplecostomus microps
Pareiorhaphis garbei
Schizolecis guntheri
Ancistrus sp. 1
Ancistrus sp. 2
Ancistrus multispinis
Dekeyseria amazonica
Baryancistrus xanthellus
Pterygoplichthys sp.
Pterygoplichthys pardalis
Hypostomus sp.
Hypostomus cf. plecostomus
Hypostomus affinis
Aphanotorulus emarginatus
Peckoltia furcata
Ancistomus snethlageae
Panaqolus sp.
P.disjunctivus_NC015747
T-homopolymer

```
                               881
Corydoras schwartzi            -AGAATTAACAGACAGGGTC GTAC-ATTTAGTAACA---- TCATGAGTAAATATTCTG-A ATTATTT-AATGACATAATT
Corydoras nattereri            -AGAGCTAACAGACAAGGTG GTACTATTTAGTAATA---- TCATAAGTAAATATTCTG-A ATTATTT-AATGACATATTC
Hemipsilichthys nimius         AGTAATTA-------AGGTG G--T-ATTAATCCATT-TTG CTTTAACCATTATTCATCCA AAAAATTCAAGAGTATCAAA
Rineloricaria cf. lanceolata   AAGAAATATGGTTTAAGGTT GAAC-ATTTTCCAAGC---- --ATCCGCACTATTAATGTC ATGGTTT-CATGACATAACC
Rineloricaria sp.              GAAATTTGGTTTA--AGGTT GAAC-ATTTTCCAAGC---- --ATCCGCTCTAATAATGTA ATGGTTTAAATGACATATTT
Loricariichthys platymetopon   -AGAATTAACAGACAGGGTC GTAC-ATTTAGTAATA---- TCATGAGTAAATATTCTG-A ATTATTT-AATGACATAATT
Loricariichthys castaneus      -------------------- -------------------- -------------------- --------------------
Loricaria cataphracta          AGTATTTA-------AGGTA GAAC-ATTTTCCAAGCATGG GTATA-------TAAATGTA ATGATTT-AATGACATGACA
Otocinclus cf. hoppei          AAGAAATAGA-----AGGTT GAAC-ATTTTTAGATTATTA TCTCA--AACAGTAAATGTA ATGATTT-AATGACATATAC
Hypoptopoma incognitum         TCCAATTA-------GGGTG GTCC-AATTCTGGATC-TTG GTGTAAGCAATGTTAATGTA ATGATTC-AATGACATACAG
Parotocinclus maculicauda      AATAACCAAATTTA-AGGTG GAAC-GCAAATATAAC-TGC CCAGC--AAATGATAATGTA TTGATTT-AATGACATATAT
Hisonotus thayeri              AAGAACCAAATTTA-AGGTG GAAC-ATAAATTTACT---- GCCCGGCAGAAGATAATGTA TTGATTT-AATGACATATAC
Kronichthys heylandi           AATGATTATAATTA-AGGTG GAAC-ATATTTAATCT-TAC TTAAA--TAATGTTAATGTA GTGATTT-AATGACATTTAC
Neoplecostomini gen. n.        TATAATTA-------AGGTG GGAC-ATAATAAACAT-TCA CCCAAG-TAATATGAATGTA ATGATTT-AATGACATATAT
Neoplecostomus microps         AATAATTATATTTA-AGGTT GGAC-ATTTTTAATCT-GGC TTCAA--TAATATAAATGTA ATTATCT-AATGACATTTAC
Pareiorhaphis garbei           TGTATATATAATGTATAGAA GCACCCAGCCATACAGCACT ATTCTACCATCTAAATATAT ATATA-T-AAACGCACGACT
Schizolecis guntheri           -------------------- -------------------- -------------------- --------------------
Ancistrus sp. 1                AAGTACTGAAAATCAAGGTA GTTC-TTATATTAAAG-TTA ATGCG--TCCATGAAATGTA AAGATTT-AAAGACATAATC
Ancistrus sp. 2                -------------------- -------------------- -------------------- --------------------
Ancistrus multispinis          AGTACTAAAAAATA-AGGTA GTTC-TAAAATTAGTA-TTA ATAAG--TCCATTTAATGTG AAGATTT-AAAGACATAATA
Dekeyseria amazonica           GACAATTGAATTA--AGGTG G--T-ACTAATAAGTA-TTG TTAAAGTTACAGATAATGAA AAGATTT-AATGACATATTA
Baryancistrus xanthellus       AATAATTAATA----AGGTA GA---ATATAATAGTCATTC ATGAA-TTACATAACATGAA AAGATTT-AAAGACATA-TC
Pterygoplichthys sp.           GATATTTTAAATA--AGGTG GAAT-AATAAATAGTT-GGG TTAAA--TACAGTAAATGTA AAGATTT-AAAGACATAATC
Pterygoplichthys pardalis      GATATTTAAAATA--GGGTG GAAT-AATAAATAACT-GGG TTAAA--TACAGTAAATGTA AAGATTT-AAAGACATAATC
Hypostomus sp.                 AACATATAATAATACAGGTG GAAC-ATAAATTAACT-TAA TTAAA--TACAGATTATGTA AAGATTT-AAGGACATAATA
Hypostomus cf. plecostomus     GACATTTAAAATT--AGGTG GAAC-ATAAGTTAAAT-TAA TTAAA--TACAGTTTATGTG AAGATTT-AAAGACATAATA
Hypostomus affinis             GACATTTAAAATT--AGGTG GAAC-ATAAGTTGTTT-TAG TTAAA--TACAGTTTATGTA AAGATTT-AAGGACATAATA
Aphanotorulus emarginatus      GATATTTCTATATA-AGGTG GAAC-ATGAATTAATA-TTA TTAAG--TACAGTTTATGTA GAGATTT-AAAGACATAATC
Peckoltia furcata              GACATTTATATTTA-AGGTG GAAT-ATGGATTAAAT-TAG TTAAA--TACATTTCATGCA AAGATTT-AAGGACATAATT
Ancistomus snethlageae         GACATTTATATTTA-AGGTG GAAC-ATAAATTAAAT-TAG TTAAA--TACATACTATGTA GAGATTT-AAAGACATAATC
Panaqolus sp.                  GACAA-TAATATATAGGGTG GAAT-ATAAATTAAAC-TGA TTAAG--CACATTTTATGTA ATGATTT-AAGGACATAATA
P.disjunctivus_NC015747        AATATTTGAGATA--AGGTG GAAT-AATAAATAACT-GGA TTAAA--TACAGTGAATGTA AAGATTT-AAAGACATAATC
```

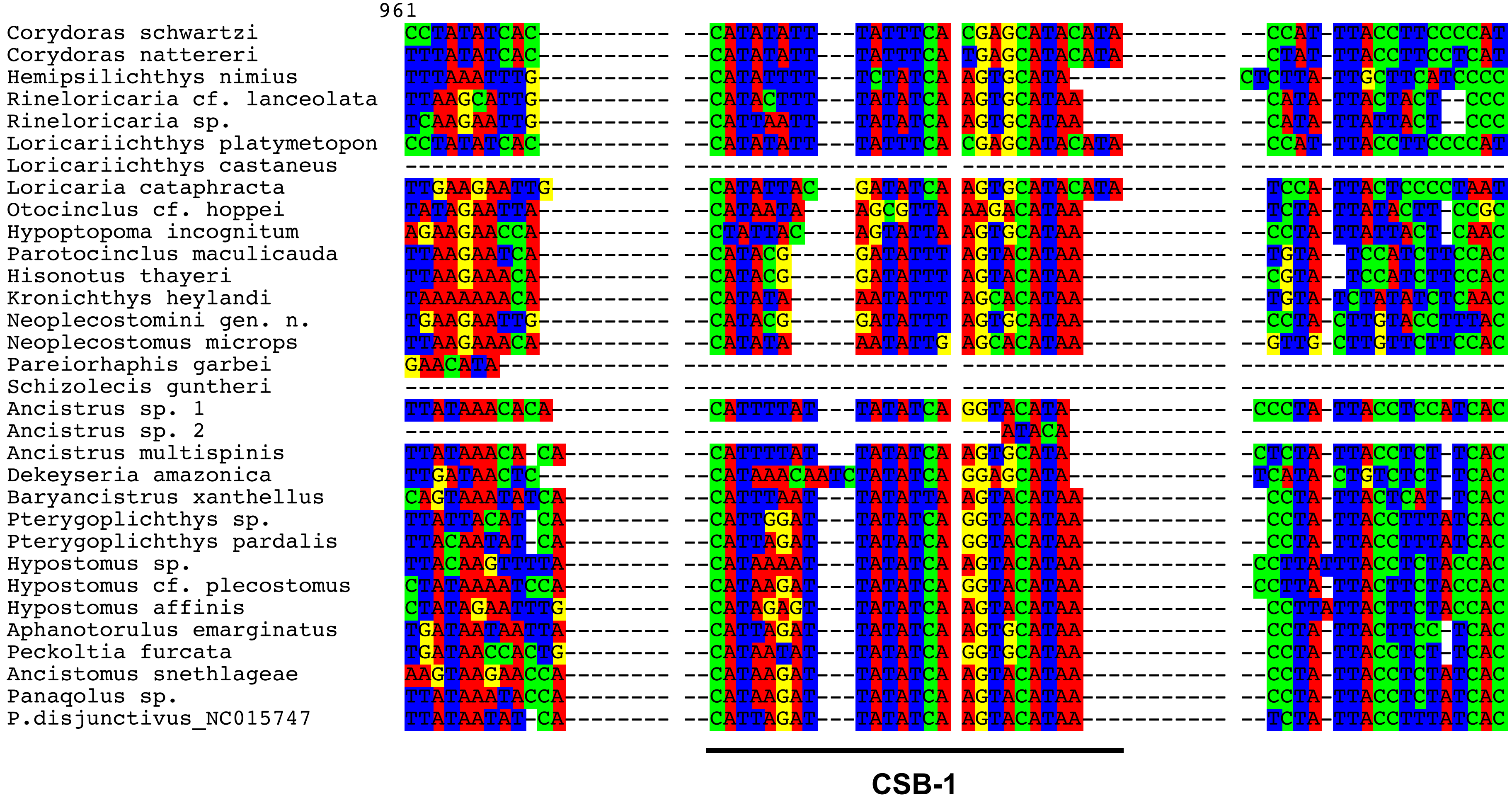
961
Corydoras schwartzi
Corydoras nattereri
Hemipsilichthys nimius
Rineloricaria cf. lanceolata
Rineloricaria sp.
Loricariichthys platymetopon
Loricariichthys castaneus
Loricaria cataphracta
Otocinclus cf. hoppei
Hypoptopoma incognitum
Parotocinclus maculicauda
Hisonotus thayeri
Kronichthys heylandi
Neoplecostomini gen. n.
Neoplecostomus microps
Pareiorhaphis garbei
Schizolecis guntheri
Ancistrus sp. 1
Ancistrus sp. 2
Ancistrus multispinis
Dekeyseria amazonica
Baryancistrus xanthellus
Pterygoplichthys sp.
Pterygoplichthys pardalis
Hypostomus sp.
Hypostomus cf. plecostomus
Hypostomus affinis
Aphanotorulus emarginatus
Peckoltia furcata
Ancistomus snethlageae
Panaqolus sp.
P.disjunctivus_NC015747
CSB-1

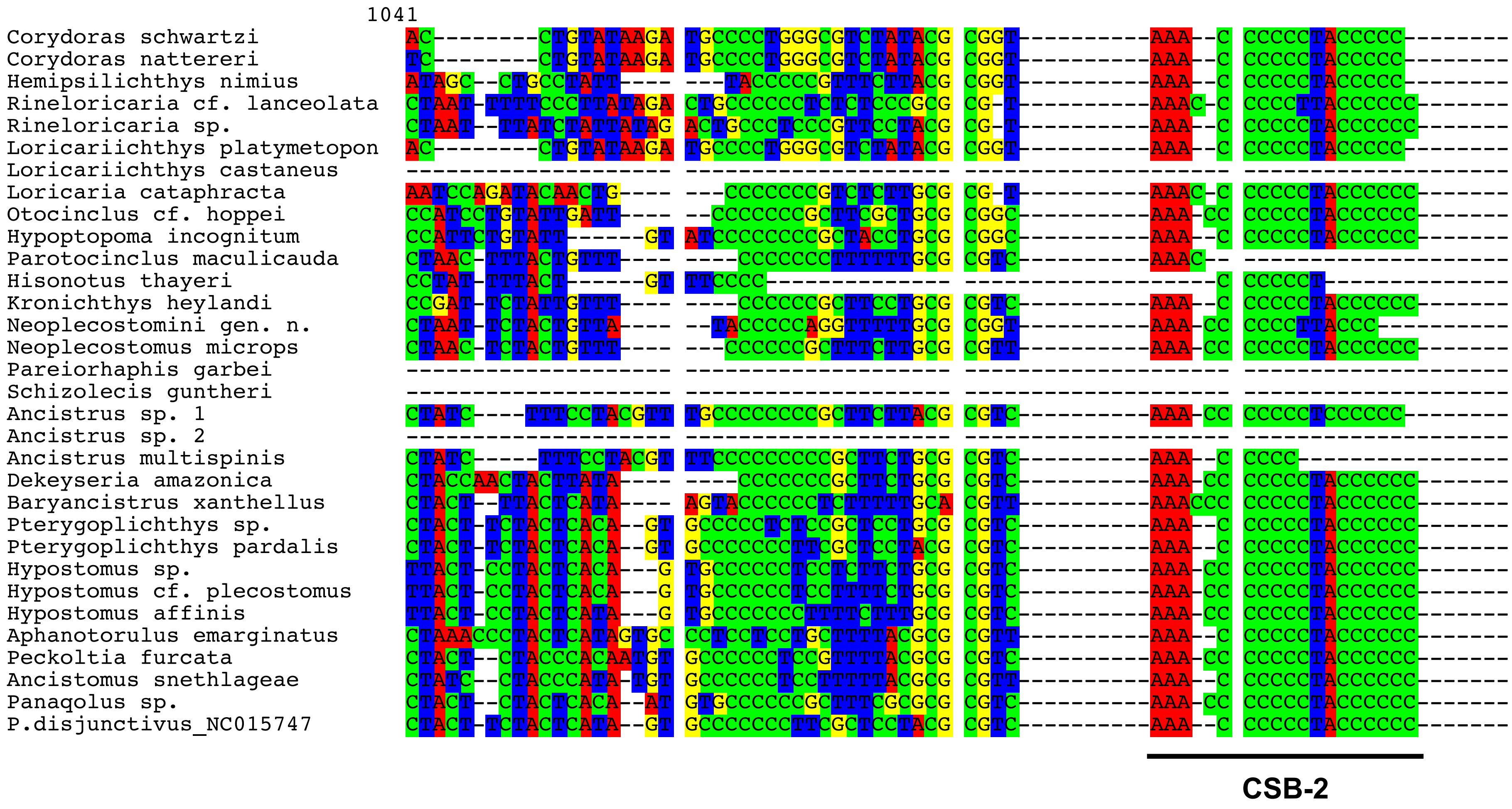
1041
Corydoras schwartzi
Corydoras nattereri
Hemipsilichthys nimius
Rineloricaria cf. lanceolata
Rineloricaria sp.
Loricariichthys platymetopon
Loricariichthys castaneus
Loricaria cataphracta
Otocinclus cf. hoppei
Hypoptopoma incognitum
Parotocinclus maculicauda
Hisonotus thayeri
Kronichthys heylandi
Neoplecostomini gen. n.
Neoplecostomus microps
Pareiorhaphis garbei
Schizolecis guntheri
Ancistrus sp. 1
Ancistrus sp. 2
Ancistrus multispinis
Dekeyseria amazonica
Baryancistrus xanthellus
Pterygoplichthys sp.
Pterygoplichthys pardalis
Hypostomus sp.
Hypostomus cf. plecostomus
Hypostomus affinis
Aphanotorulus emarginatus
Peckoltia furcata
Ancistomus snethlageae
Panaqolus sp.
P.disjunctivus_NC015747
CSB-2

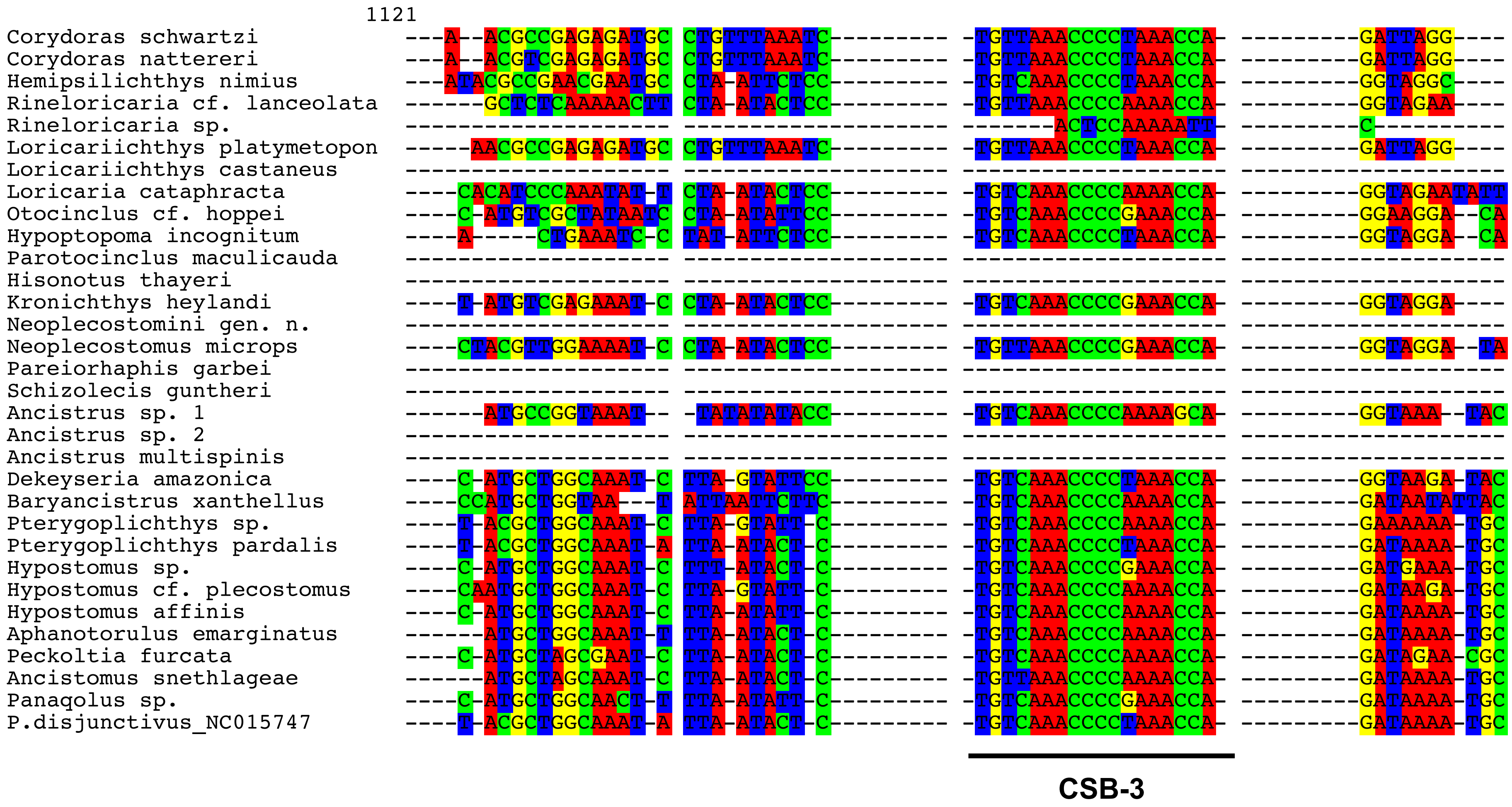
1121
Corydoras schwartzi
Corydoras nattereri
Hemipsilichthys nimius
Rineloricaria cf. lanceolata
Rineloricaria sp.
Loricariichthys platymetopon
Loricariichthys castaneus
Loricaria cataphracta
Otocinclus cf. hoppei
Hypoptopoma incognitum
Parotocinclus maculicauda
Hisonotus thayeri
Kronichthys heylandi
Neoplecostomini gen. n.
Neoplecostomus microps
Pareiorhaphis garbei
Schizolecis guntheri
Ancistrus sp. 1
Ancistrus sp. 2
Ancistrus multispinis
Dekeyseria amazonica
Baryancistrus xanthellus
Pterygoplichthys sp.
Pterygoplichthys pardalis
Hypostomus sp.
Hypostomus cf. plecostomus
Hypostomus affinis
Aphanotorulus emarginatus
Peckoltia furcata
Ancistomus snethlageae
Panaqolus sp.
P.disjunctivus_NC015747
CSB-3

```
                                1201
Corydoras schwartzi             -------------------- -------------------- ---------CTCGATC---- ------GGCGACAA----GC
Corydoras nattereri             -------------------- -------------------- ---------CTCGACC---- ------GACGACAA----GC
Hemipsilichthys nimius          -------------------- -------------------- -------------------- ------------------CC
Rineloricaria cf. lanceolata    -------------------- --TTTTTCGAT--------A GCTCATGTCACCACCC---- ------ACTCAC--------
Rineloricaria sp.               -------------------- -------------------- -------------------- --------------------
Loricariichthys platymetopon    -------------------- -------------------- ---------CTCGATC---- ------GGCGACAA----GC
Loricariichthys castaneus       -------------------- -------------------- -------------------- --------------------
Loricaria cataphracta           CAGTGATGTAAAACCA---- -------------------A GCCCACACATTACATT---- ------ATACATTATACATT
Otocinclus cf. hoppei           CAACAT-GCGTAGTACTCAA AATATGATAAAGCCCGGACC ACCTAAACACACAACCCACA CAATGTAGGGACATCCCCAT
Hypoptopoma incognitum          CAGCGGGTAACCCATTAAAA CACACAACCCA--------A ACATGTACATTATTTC---- ------TCTCCCCC----AC
Parotocinclus maculicauda       -------------------- -------------------- -------------------- --------------------
Hisonotus thayeri               -------------------- -------------------- -------------------- --------------------
Kronichthys heylandi            ---CACAACATATAAAACAC ATAAGTACAAA--------C AGTTAACCTAAAACAC---- ------AAATAAAC----CC
Neoplecostomini gen. n.         -------------------- -------------------- -------------------- --------------------
Neoplecostomus microps          CAACATATAAAACACCTAAA ACAACAAAAATGAGCCTAAA AAATACAAAAAACCCC---- ------GGACATACAACACT
Pareiorhaphis garbei            -------------------- -------------------- -------------------- --------------------
Schizolecis guntheri            --
Ancistrus sp. 1                 CAACATACAATACAC--GTA ATTCCTAAAAT--------T ATTTGC----CGACCC---- ------AGACATAT----AT
Ancistrus sp. 2                 -------------------- -------------------- -------------------- --------------------
Ancistrus multispinis           -------------------- -------------------- -------------------- --------------------
Dekeyseria amazonica            CAACAT-------------- ------AAAAT--------A ATATACACACCGACCC---- ------AGACATAT----AT
Baryancistrus xanthellus        CAACATAAAACATTTAATTC TTCACCAAAAT--------A ACTTACACATCAACCC---- ------AGATATAC----CT
Pterygoplichthys sp.            CATCATAATAAATTACATAA ATTACCAAATT--------A ACTTGCATA-CAACCC---- ------AGACATAT----CT
Pterygoplichthys pardalis       CACCATAATAAATTATATAA ACTACCAAACT--------A ACTTGCACA-CAACCC---- ------AGACATAC----CT
Hypostomus sp.                  CAACAC----AATTATATAA CCTTTCAAAAT--------A ATTTGTACA-CAACCC---- ------AGACATAC----CC
Hypostomus cf. plecostomus      CAACACAATAAATTATATAA CCTTTCAAAAT--------A ACTTGTACA-CAACCC---- ------AGACATAC----CC
Hypostomus affinis              CAACACAGTAAATTATATAA CCTTTCAAAAT--------A ACTTGTACA-CAACCC---- ------AGACATAC----CC
Aphanotorulus emarginatus       CAACATTGAATCATATAATT TCTCTCAAAAT--------A ACTTACACATCGACCC---- ------AGACATAC----CT
Peckoltia furcata               CAACACTGAATTTTATAACC ACTATCAAAAT--------A ACTTATACATCAACCC---- ------AGACATAT----CT
Ancistomus snethlageae          CAACACTGAATTATATAACC ACTATTAAGAT--------A ACCTACACATCAACCC---- ------AGACATAT----CT
Panaqolus sp.                   CAACACTGAATTGTACAATC ACTATCAAAAT--------A ATTTACACATCGACCC---- ------AGACATAC----CT
P.disjunctivus_NC015747         CACCATAATAAATTATATAA ACTACCAAACT--------A ACTTGCACA-CAACCC---- ------AGACATAC----CT
```

```
                                 1281
Corydoras schwartzi              AACGAGTATA---------- -------------------- --ATGTGTCAC--------- --------------------
Corydoras nattereri              AACGAGTATA---------- -------------------- --ATGTGTCAC--------- --------------------
Hemipsilichthys nimius           GATCAGCGTG---------- -------------------- --AT---------------- --------------------
Rineloricaria cf. lanceolata     ------TACA---------- -------------------- --GTATATTGC--------- --------------------
Rineloricaria sp.                -------------------- -------------------- -------------------- --------------------
Loricariichthys platymetopon     AACGAG-------------- -------------------- -------------------- --------------------
Loricariichthys castaneus        -------------------- -------------------- -------------------- --------------------
Loricaria cataphracta            ATACAATATA---------- -------------------- --TAATATTACATTATACAT TATATAA-------------
Otocinclus cf. hoppei            AATATATTTT---------- -------------------- --ATACCCCACTACTATATA TATATAGTCATATATAGTTA
Hypoptopoma incognitum           AAACAACCCC---------- -------------------- --ACAAACCCAACCACGTAA CACATCAGTAT---------
Parotocinclus maculicauda        -------------------- -------------------- -------------------- --------------------
Hisonotus thayeri                -------------------- -------------------- -------------------- --------------------
Kronichthys heylandi             CGGACGTACA---------- -------------------- --CGCCATTAAACATGCTAG AATAAGTACAAAGACACCTA
Neoplecostomini gen. n.          -------------------- -------------------- -------------------- --------------------
Neoplecostomus microps           ACCAAGCATATTAAAGTAGG CACATGGACACACCGCACCA TTATATAGTATTTTATACCA TAAATAT-------------
Pareiorhaphis garbei             -------------------- -------------------- -------------------
Schizolecis guntheri
Ancistrus sp. 1                  AATAAATTTA---------- -------------------- --AT-TACCACATCAGACGG CAAAACT-------------
Ancistrus sp. 2                  -------------------- -------------------- -------------------- --------------------
Ancistrus multispinis            -------------------- -------------------- -------------------- --------------------
Dekeyseria amazonica             AGTATATTCA---------- -------------------- --ATCCACCTCTCCAAACGA CACCTGT--AG---------
Baryancistrus xanthellus         AATAAATATA---------- -------------------- --ACTTACCGCATCAAACGG CATATAT-------------
Pterygoplichthys sp.             AAT-AACACA---------- -------------------- --AAACATTACACCAAACGG TATATAT-------------
Pterygoplichthys pardalis        AAT-AACACA---------- -------------------- --AAACATTTCACCAAACGG CATATAT-------------
Hypostomus sp.                   AAT-AATACA---------- -------------------- --AAACATTACCTCAAACGG CATATAT-------------
Hypostomus cf. plecostomus       AAT-AATATA---------- -------------------- --AAATATCACCTCAAACGG CACATAT-------------
Hypostomus affinis               AATAAATATA---------- -------------------- --AAATATTACCTCAAACGG TACATAT-------------
Aphanotorulus emarginatus        AAT-AGTATA---------- -------------------- --AAACACTATACTACACAG TATAAAT--AA---------
Peckoltia furcata                AAT-AATATA---------- -------------------- --AAATATTAC--------- --------------------
Ancistomus snethlageae           AAT-AACATA---------- -------------------- --AAATATTATG-------- --------------------
Panaqolus sp.                    AAT-AACATA---------- -------------------- --AAAAATTCTA-------- --------------------
P.disjunctivus_NC015747          AAT-AACACA---------- -------------------- --AAACATTTCACCAAACGG CATATAT-------------
```

```
                              1361
Corydoras schwartzi           -------------------- -------------------- -------------------- --------------------
Corydoras nattereri           -------------------- -------------------- -------------------- --------------------
Hemipsilichthys nimius        -------------------- -------------------- -------------------- --------------------
Rineloricaria cf. lanceolata  -------------------- -------------------- -------------------- --------------------
Rineloricaria sp.             -------------------- -------------------- -------------------- --------------------
Loricariichthys platymetopon  -------------------- -------------------- -------------------- --------------------
Loricariichthys castaneus     -------------------- -------------------- -------------------- --------------------
Loricaria cataphracta         -------------------- -------------------- -------------------- --------------------
Otocinclus cf. hoppei         TATAGTTATATAGTTATATA GTTATATAGTTATATAGTTA TATAGTTATATAGATTTATT TATATAGTGTATATATTTAT
Hypoptopoma incognitum        -------------------- ------------GCTATATA TACGTGTTATATAGTGTGTT ATATAGTGTGTTATATAGTG
Parotocinclus maculicauda     -------------------- -------------------- -------------------- --------------------
Hisonotus thayeri             -------------------- -------------------- -------------------- --------------------
Kronichthys heylandi          CACATCATGGTATTATATAT AGTACTGTATTATATATAGT ACTGTATTATATATAGTACT GTATTATATATAGTACTGTA
Neoplecostomini gen. n.       -------------------- -------------------- -------------------- --------------------
Neoplecostomus microps        -------------------- -------------------- -------------------- --------GGTAATATAGGG
Pareiorhaphis garbei
Schizolecis guntheri
Ancistrus sp. 1               -------------------- -------------------- -------------------- --------------------
Ancistrus sp. 2               -------------------- -------------------- -------------------- --------------------
Ancistrus multispinis         -------------------- -------------------- -------------------- --------------------
Dekeyseria amazonica          -------------------- -------------------- -------------------- --------------------
Baryancistrus xanthellus      -------------------- -------------------- ---------TAGCATATATT TATTTACACTAATTATATAA
Pterygoplichthys sp.          -------------------- -------------------- -------------------- --------------------
Pterygoplichthys pardalis     -------------------- -------------------- -------------------- --------------------
Hypostomus sp.                -------------------- -------------------- -------------------- --------------------
Hypostomus cf. plecostomus    -------------------- -------------------- -------------------- --------------------
Hypostomus affinis            -------------------- -------------------- -------------------- --------------------
Aphanotorulus emarginatus     -------------------- -------------------- -------------------- --------------------
Peckoltia furcata             -------------------- -------------------- -------------------- --------------------
Ancistomus snethlageae        -------------------- -------------------- -------------------- --------------------
Panaqolus sp.                 -------------------- -------------------- -------------------- --------------------
P.disjunctivus_NC015747       -------------------- -------------------- -------------------- ------------------TG
```

```
                               1441
Corydoras schwartzi            -TATATAT------------ ---TGTTGCAT----ATACA CTATATA------------- --------------------
Corydoras nattereri            -TATATAT------------ ---TGTTGCAT----ATACA CTATATA------------- --------------------
Hemipsilichthys nimius         -------------------- -------------------- -------------------- --------------------
Rineloricaria cf. lanceolata   -------------------- ----------------TACA GTATA--------------- --------------------
Rineloricaria sp.              -------------------- -------------------- -------------------- --------------------
Loricariichthys platymetopon   -------------------- -------------------- -------------------- --------------------
Loricariichthys castaneus      -------------------- -------------------- -------------------- --------------------
Loricaria cataphracta          -------------------- ---TATTACATT---ATACA TTATATAATATTACAT---- ------TATAC---------
Otocinclus cf. hoppei          TTATATAGTG-TATATATTT ATTTATATAGTGTATATATT TATTTATATAGTGTATATAT AATGCATACATGTATATATA
Hypoptopoma incognitum         TGTTATATAG-TGTGTTATA TAGTGTGTTAT----ATAGT GTGTTATATAGTGTGT---- ------TATAT---AATGCT
Parotocinclus maculicauda      -------------------- -------------------- -------------------- --------------------
Hisonotus thayeri              -------------------- -------------------- -------------------- --------------------
Kronichthys heylandi           TTATATATAG-TACTGTATT AACTGTATTATAGTACTGTA TTATAGTACTGTATTA---- ------TAGTACTGTATTAT
Neoplecostomini gen. n.        -------------------- -------------------- -------------------- --------------------
Neoplecostomus microps         CTATAGAA-T-AGTGCAACA CAATACAATACTAGAATATA GTATATTATATTATAT---- ------TATAT---TATATT
Pareiorhaphis garbei
Schizolecis guntheri
Ancistrus sp. 1                -AGCCTACTC-TTTTTTCCC CCATCAATTTA----TTTTT ATATATTATATTATAT---- ------ATTATATATATATA
Ancistrus sp. 2                -------------------- -------------------- -------------------- --------------------
Ancistrus multispinis          -------------------- -------------------- -------------------- --------------------
Dekeyseria amazonica           -TCTATAT-T-TATAGATTT ATTTAGTTTAT----TTATA TATTATATTATATTAT---- ------ATTAT---ATTATT
Baryancistrus xanthellus       TTATATAT------------ --ATATATTATATATATATA ATATATATAATTAT------ --------TATATTAAAATT
Pterygoplichthys sp.           -------TGG-TATGTGTCT TTTTATATCATACCAAATTT ATATATA------------- ------TATATATATATATA
Pterygoplichthys pardalis      -------------------- ---TAGTATAT----ATCTT TTATATTGCACCAATT---- -------------ATATATA
Hypostomus sp.                 TAGTATATAT---------- ---TATGTCAC----TTATA TTATATTATATTATAT---- --------------TATATT
Hypostomus cf. plecostomus     TCGCATATAGATCCACACCA ATATATATTAA----TTATA TTATATTATATTATAT---- --------------TATATT
Hypostomus affinis             -----------TCTCAATCT CCCTACTACAA--------- -------------------- --------------TACTTA
Aphanotorulus emarginatus      -TATATAT-T-TACCTACAT TATTATATTAT-----TATA TATTATTATATATTAT---- ------TATAT---TATTAT
Peckoltia furcata              ----ATATGC-CCCTTATAT ATATACTATAT----ATATA CTATATA------------- ------TATAC---TATATA
Ancistomus snethlageae         -------------------- ---TATGTCCC----TTATA TTATATTATATTATAT---- ------TATAT---TATATT
Panaqolus sp.                  -------------------- ---TGCACCCC----TTATA TTATATTATATTATAT---- ------TATAT---TATATT
P.disjunctivus_NC015747        GTATATATCT-TT------- ---TATATTGC----ACCAA TTATATATATATATAT---- -------ATATATATATATA
```

```
                                1521
Corydoras schwartzi            -------------------- -------------------- -------------------- --------------------
Corydoras nattereri            -------------------- -------------------- -------------------- --------------------
Hemipsilichthys nimius         -------------------- -------------------- -------------------- --------------------
Rineloricaria cf. lanceolata   -------------------- -------------------- -------------------- --------------------
Rineloricaria sp.              -------------------- -------------------- -------------------- --------------------
Loricariichthys platymetopon   -------------------- -------------------- -------------------- --------------------
Loricariichthys castaneus      -------------------- -------------------- -------------------- --------------------
Loricaria cataphracta          ---ATTATATAATATTACAT TATACATT------------ -------------------- --------------------
Otocinclus cf. hoppei          CATAATGTATTATAGTGTTA CATATA-------------- -------------------- --------------------
Hypoptopoma incognitum         AGTGTTATATAGCACAATAT ATATACATATCACACAGTAC ACTGTACCACCTGACCCTAA CATAAACTATTTATTTTACA
Parotocinclus maculicauda      -------------------- -------------------- -------------------- --------------------
Hisonotus thayeri              -------------------- -------------------- -------------------- --------------------
Kronichthys heylandi           AGTACTGTATTATGTTACAC ACGATCCTGTAATGCTGTAC TACTGTAATACACCCCAACA CATTGTAGGATATTATAGTA
Neoplecostomini gen. n.        -------------------- -------------------- -------------------- --------------------
Neoplecostomus microps         A-TATTATATTATATTATAT TATATTATATTATATTAT-- -------------------- --------------------
Pareiorhaphis garbei
Schizolecis guntheri
Ancistrus sp. 1                GATTTTATATAATAAAAAAA -------------------- -------------------- --------------------
Ancistrus sp. 2                -------------------- -------------------- -------------------- --------------------
Ancistrus multispinis          -------------------- -------------------- -------------------- --------------------
Dekeyseria amazonica           A-TATTATATTATA------ -------------------- -------------------- --------------------
Baryancistrus xanthellus       AATTATACATTACACCTGAT TCTACAT------------- -------------------- --------------------
Pterygoplichthys sp.           TAAAATACAT---------- -------------------- -------------------- --------------------
Pterygoplichthys pardalis      TATATAAAATCACAC----- -------------------- -------------------- --------------------
Hypostomus sp.                 A-TATTATATTATATTATAT TA------------------ -------------------- --------------------
Hypostomus cf. plecostomus     A-TATTATATTATATTATAT TATATTATATTATATTATAT TATATTATATTATATTATTC CACAC---------------
Hypostomus affinis             TATATTATAT---------- -------------------- -------------------- --------------------
Aphanotorulus emarginatus      A-TTAT-TATATTATTATAT TATTATATTA---------- -------------------- --------------------
Peckoltia furcata              TATACTATATATATACT--- -------------------- -------------------- --------------------
Ancistomus snethlageae         A-TATTATATTATATTATAT TATATTA------------- -------------------- --------------------
Panaqolus sp.                  A-TATTATATTA-------- -------------------- -------------------- --------------------
P.disjunctivus_NC015747        TATATTATAATATAATATAT TACAAAATCACAC------- -------------------- --------------------
```

```
                                1601
Corydoras schwartzi            -------------------- -------------------- -----
Corydoras nattereri            -------------------- -------------------- -----
Hemipsilichthys nimius         -------------------- -------------------- -----
Rineloricaria cf. lanceolata   -------------------- -------------------- -----
Rineloricaria sp.              -------------------- -------------------- -----
Loricariichthys platymetopon   -------------------- -------------------- -----
Loricariichthys castaneus      -------------------- -------------------- -----
Loricaria cataphracta          -------------------- -------------------- -----
Otocinclus cf. hoppei          -------------------- -------------------- -----
Hypoptopoma incognitum         TACACAATAATACAACACAC CCAG---------------- -----
Parotocinclus maculicauda      -------------------- -------------------- -----
Hisonotus thayeri              -------------------- -------------------- -----
Kronichthys heylandi           TATCCTTATATATTCTATTA TGATCATTATATATATATAT AACAT
Neoplecostomini gen. n.        -------------------- -------------------- -----
Neoplecostomus microps         -------------------- -------------------- -----
Pareiorhaphis garbei
Schizolecis guntheri
Ancistrus sp. 1                -------------------- -------------------- -----
Ancistrus sp. 2                -------------------- -------------------- -----
Ancistrus multispinis          -------------------- -------------------- -----
Dekeyseria amazonica           -------------------- -------------------- -----
Baryancistrus xanthellus       -------------------- -------------------- -----
Pterygoplichthys sp.           -------------------- -------------------- -----
Pterygoplichthys pardalis      -------------------- -------------------- -----
Hypostomus sp.                 -------------------- -------------------- -----
Hypostomus cf. plecostomus     -------------------- -------------------- -----
Hypostomus affinis             -------------------- -------------------- -----
Aphanotorulus emarginatus      -------------------- -------------------- -----
Peckoltia furcata              -------------------- -------------------- -----
Ancistomus snethlageae         -------------------- -------------------- -----
Panaqolus sp.                  -------------------- -------------------- -----
P.disjunctivus_NC015747        -------------------- -------------------- -----
```
