# Supplementary material for: Reducing the information gap on Loricarioidei (Siluriformes) mitochondrial genomics
Source: BMC Genomics. 2017 May 4;18:345. doi: 10.1186/s12864-017-3709-3 (PMC5418769; doi:10.1186/s12864-017-3709-3)
Supplement: Supplementary file 6 — Phylogenetic tree retrieved using the three longest genes (A) and the three genes with the best performance (B). The three longest genes, cox1, nad4 and nad5, also harbored most informative characters. A - The position of Dekeyseria amazonica, highlighted in a red box, changed in relation to the tree using the 15 mitochondrial genes. The three genes with best performance as identified by Havird & Santos [13] were nad2, nad4 and nad5. B - The position of Loricaria cataphracta, highlighted in a red box, changed in relation to the tree using the 15 mitochondrial genes. Genes were aligned with built-in MUSCLE using SeaView and used for phylogenetic tree reconstruction using RAxML under the GTR + GAMMA + I model and 1000 bootstrap replica. (PDF 62 kb) [file 12864_2017_3709_MOESM6_ESM.pdf]

Additional file 6: Phylogenetic tree retrieved using the three longest genes (A) and the three genes with the best performance (B). The three longest genes, cox1, nad4 and nad5, also harbored most informative characters. A - The position of *Dekeyseria amazonica*, highlighted in a red box, changed in relation to the tree using the 15 mitochondrial genes. The three genes with best performance as identified by Havird & Santos 2014 were nad2, nad4 and nad5. B - The position of *Loricaria cataphracta*, highlighted in a red box, changed in relation to the tree using the 15 mitochondrial genes. Genes were aligned with built-in MUSCLE using SeaView and used for phylogenetic tree reconstruction using RAxML under the GTR+GAMMA+I model and 1000 bootstrap replica.

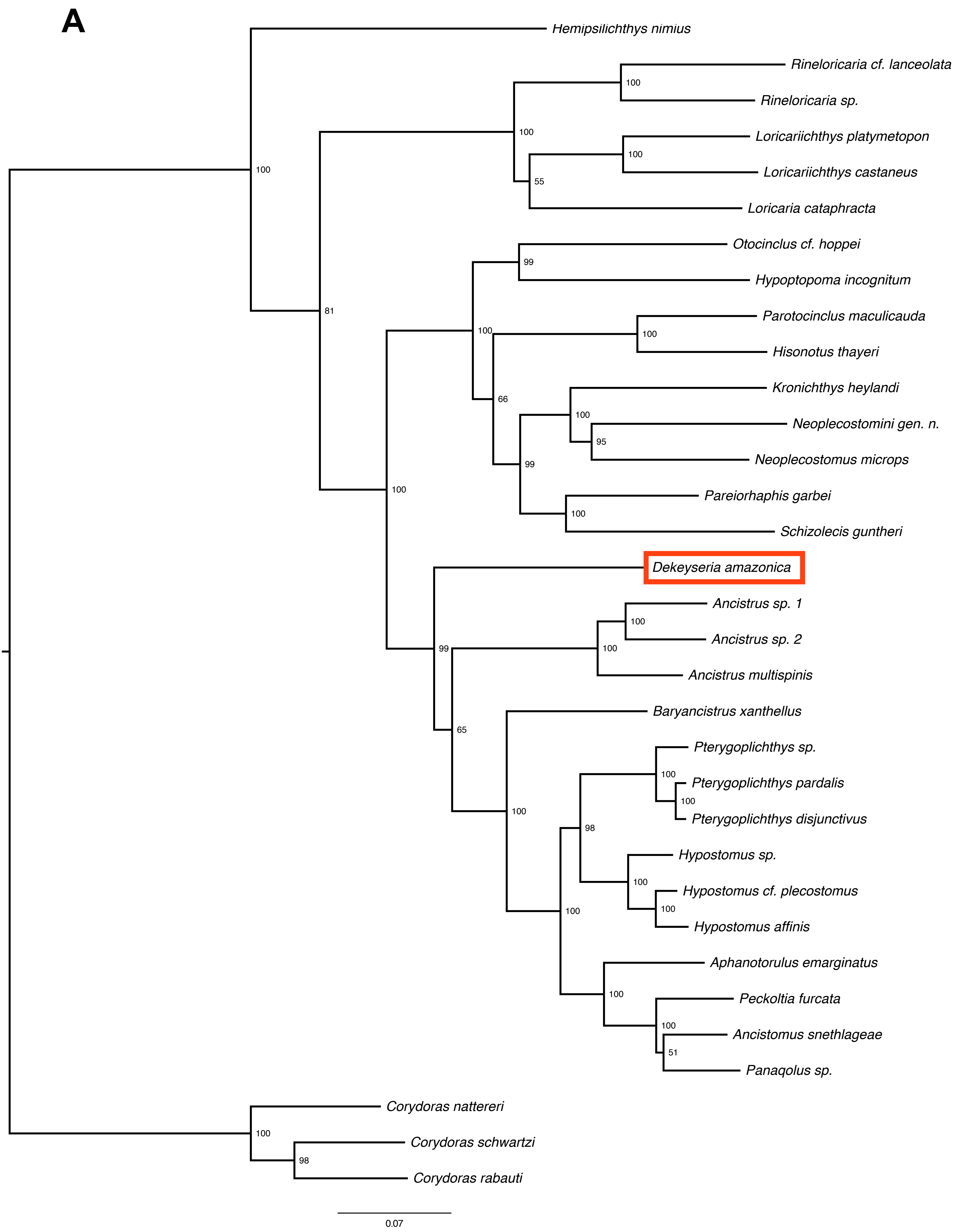

A
Hemipsilichthys nimius
Rineloricaria cf. lanceolata
Rineloricaria sp.
Loricariichthys platymetopon
Loricariichthys castaneus
Loricaria cataphracta
Otocinclus cf. hoppei
Hypoptopoma incognitum
Parotocinclus maculicauda
Hisonotus thayeri
Kronichthys heylandi
Neoplecostomini gen. n.
Neoplecostomus microps
Pareiorhaphis garbei
Schizolecis guntheri
Dekeyseria amazonica
Ancistrus sp. 1
Ancistrus sp. 2
Ancistrus multispinis
Baryancistrus xanthellus
Pterygoplichthys sp.
Pterygoplichthys pardalis
Pterygoplichthys disjunctivus
Hypostomus sp.
Hypostomus cf. plecostomus
Hypostomus affinis
Aphanotorulus emarginatus
Peckoltia furcata
Ancistomus snethlageae
Panaqolus sp.
Corydoras nattereri
Corydoras schwartzi
Corydoras rabauti
0.07

B

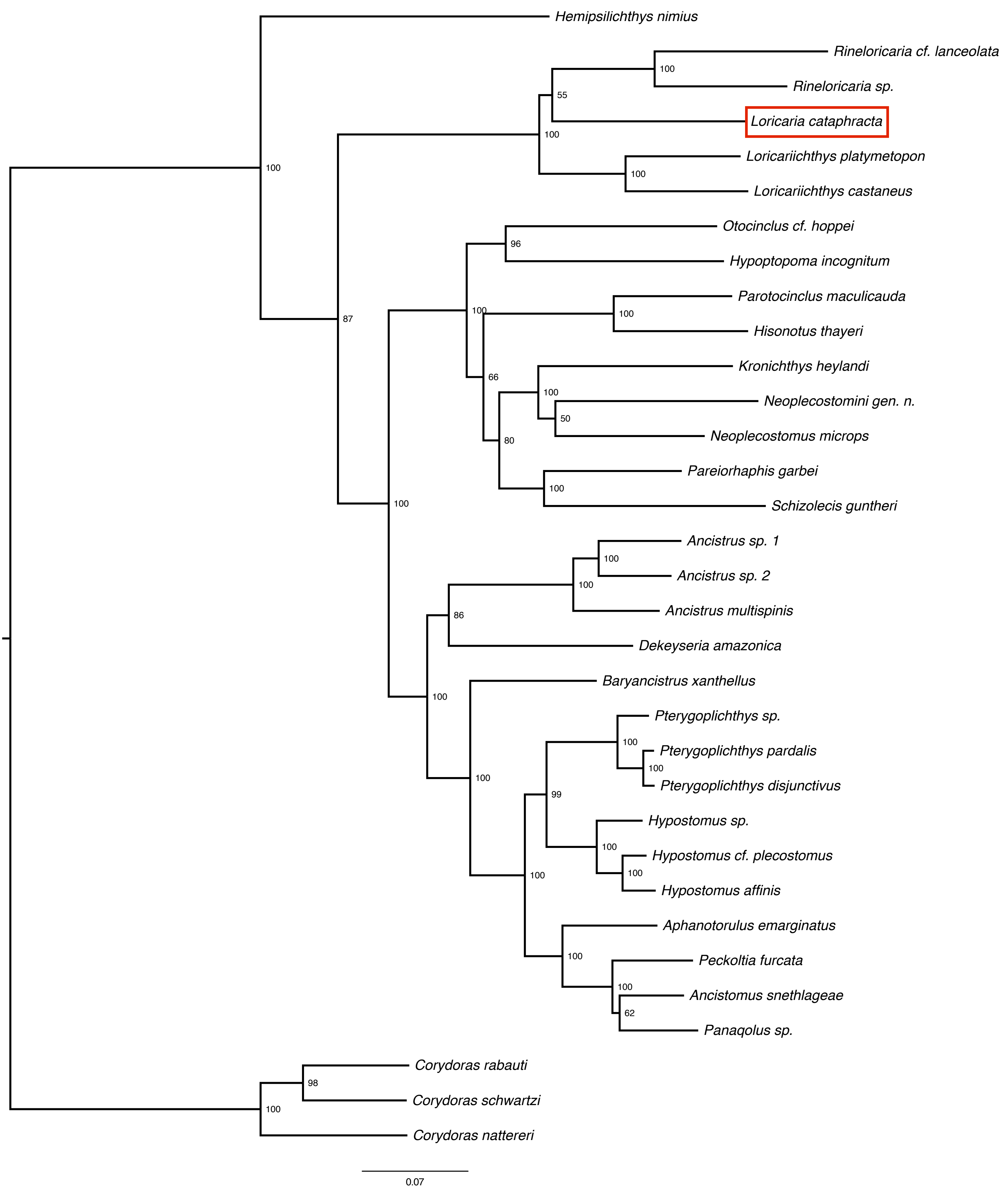
Hemipsilichthys nimius
Rineloricaria cf. lanceolata
Rineloricaria sp.
Loricaria cataphracta
Loricariichthys platymetopon
Loricariichthys castaneus
Otocinclus cf. hoppei
Hypoptopoma incognitum
Parotocinclus maculicauda
Hisonotus thayeri
Kronichthys heylandi
Neoplecostomini gen. n.
Neoplecostomus microps
Pareiorhaphis garbei
Schizolecis guntheri
Ancistrus sp. 1
Ancistrus sp. 2
Ancistrus multispinis
Dekeyseria amazonica
Baryancistrus xanthellus
Pterygoplichthys sp.
Pterygoplichthys pardalis
Pterygoplichthys disjunctivus
Hypostomus sp.
Hypostomus cf. plecostomus
Hypostomus affinis
Aphanotorulus emarginatus
Peckoltia furcata
Ancistomus snethlageae
Panaqolus sp.
Corydoras rabauti
Corydoras schwartzi
Corydoras nattereri
100
55
100
100
100
96
100
100
87
66
100
50
80
100
100
100
100
86
100
100
100
100
99
100
100
100
100
100
62
98
100
0.07
