# Supplementary material for: Reducing the information gap on Loricarioidei (Siluriformes) mitochondrial genomics
Source: BMC Genomics. 2017 May 4;18:345. doi: 10.1186/s12864-017-3709-3 (PMC5418769; doi:10.1186/s12864-017-3709-3)
Supplement: Supplementary file 7 — Confirmation of species identification using cox1 barcode sequence and the BOLD Systems. The Folmer region of Cytochrome c oxidase subunit 1 of each species was used as queries for similarity searches against the BOLD database. The resulting output is presented as a phylogenetic tree generated online. The query species are shown in red and indicated by a red arrow. For each tree, the species name, as well as its field and voucher numbers are shown above the arrow. (PDF 765 kb) [file 12864_2017_3709_MOESM7_ESM.pdf]

Additional file 7: Confirmation of species identification using cox1 barcode sequence and the BOLD Systems. The Folmer region of Cytochrome c oxidase subunit 1 of each species was used as queries for similarity searches against the BOLD database. The result output was visualized as a phylogenetic tree generated online. The query species are shown in red and indicated by a red arrow. For each tree, the species name, as well as its field and voucher numbers are shown close above the arrow.

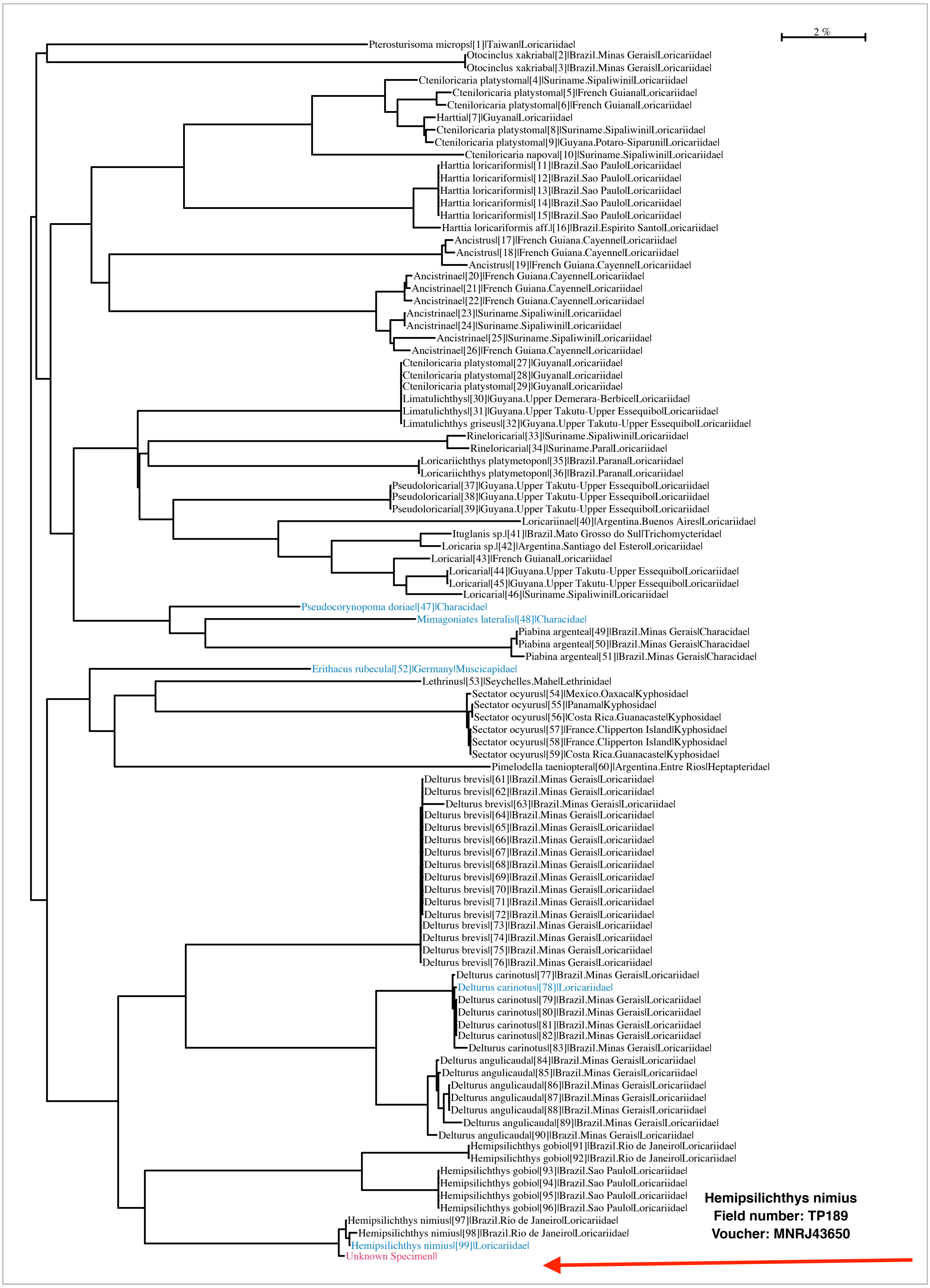
2 %
Pterosturisoma microps|[1]|Taiwan|Loricariidae|
Otocinclus xakriaba|[2]|Brazil.Minas Gerais|Loricariidae|
Otocinclus xakriaba|[3]|Brazil.Minas Gerais|Loricariidae|
Ctenilorcaria platystoma|[4]|Suriname.Sipaliwini|Loricariidae|
Ctenilorcaria platystoma|[5]|French Guiana|Loricariidae|
Ctenilorcaria platystoma|[6]|French Guiana|Loricariidae|
Harttia|[7]|Guyana|Loricariidae|
Ctenilorcaria platystoma|[8]|Suriname.Sipaliwini|Loricariidae|
Ctenilorcaria platystoma|[9]|Guyana.Potaro-Siparuni|Loricariidae|
Ctenilorcaria napova|[10]|Suriname.Sipaliwini|Loricariidae|
Harttia loricariformis|[11]|Brazil.Sao Paulo|Loricariidae|
Harttia loricariformis|[12]|Brazil.Sao Paulo|Loricariidae|
Harttia loricariformis|[13]|Brazil.Sao Paulo|Loricariidae|
Harttia loricariformis|[14]|Brazil.Sao Paulo|Loricariidae|
Harttia loricariformis|[15]|Brazil.Sao Paulo|Loricariidae|
Harttia loricariformis aff.|[16]|Brazil.Espirito Santo|Loricariidae|
Ancistrus|[17]|French Guiana.Cayenne|Loricariidae|
Ancistrus|[18]|French Guiana.Cayenne|Loricariidae|
Ancistrus|[19]|French Guiana.Cayenne|Loricariidae|
Ancistrinae|[20]|French Guiana.Cayenne|Loricariidae|
Ancistrinae|[21]|French Guiana.Cayenne|Loricariidae|
Ancistrinae|[22]|French Guiana.Cayenne|Loricariidae|
Ancistrinae|[23]|Suriname.Sipaliwini|Loricariidae|
Ancistrinae|[24]|Suriname.Sipaliwini|Loricariidae|
Ancistrinae|[25]|Suriname.Sipaliwini|Loricariidae|
Ancistrinae|[26]|French Guiana.Cayenne|Loricariidae|
Ctenilorcaria platystoma|[27]|Guyana|Loricariidae|
Ctenilorcaria platystoma|[28]|Guyana|Loricariidae|
Ctenilorcaria platystoma|[29]|Guyana|Loricariidae|
Limatulichthys|[30]|Guyana.Upper Demerara-Berbice|Loricariidae|
Limatulichthys|[31]|Guyana.Upper Takutu-Upper Essequibo|Loricariidae|
Limatulichthys griseus|[32]|Guyana.Upper Takutu-Upper Essequibo|Loricariidae|
Rineloricaria|[33]|Suriname.Sipaliwini|Loricariidae|
Rineloricaria|[34]|Suriname.Para|Loricariidae|
Loricariichthys platymetopon|[35]|Brazil.Parana|Loricariidae|
Loricariichthys platymetopon|[36]|Brazil.Parana|Loricariidae|
Pseudoloricaria|[37]|Guyana.Upper Takutu-Upper Essequibo|Loricariidae|
Pseudoloricaria|[38]|Guyana.Upper Takutu-Upper Essequibo|Loricariidae|
Pseudoloricaria|[39]|Guyana.Upper Takutu-Upper Essequibo|Loricariidae|
Loricariinae|[40]|Argentina.Buenos Aires|Loricariidae|
Ituglanis sp.|[41]|Brazil.Mato Grosso do Sul|Trichomycteridae|
Loricaria sp.|[42]|Argentina.Santiago del Estero|Loricariidae|
Loricaria|[43]|French Guiana|Loricariidae|
Loricaria|[44]|Guyana.Upper Takutu-Upper Essequibo|Loricariidae|
Loricaria|[45]|Guyana.Upper Takutu-Upper Essequibo|Loricariidae|
Loricaria|[46]|Suriname.Sipaliwini|Loricariidae|
Pseudocorynopoma doriae|[47]|Characidae|
Mimagoniates lateralis|[48]|Characidae|
Piabina argentea|[49]|Brazil.Minas Gerais|Characidae|
Piabina argentea|[50]|Brazil.Minas Gerais|Characidae|
Piabina argentea|[51]|Brazil.Minas Gerais|Characidae|
Erithacus rubecula|[52]|Germany|Muscicapidae|
Lethrinus|[53]|Seychelles.Mahe|Lethrinidae|
Sectator ocyurus|[54]|Mexico.Oaxaca|Kyphosidae|
Sectator ocyurus|[55]|Panama|Kyphosidae|
Sectator ocyurus|[56]|Costa Rica.Guanacaste|Kyphosidae|
Sectator ocyurus|[57]|France.Clipperton Island|Kyphosidae|
Sectator ocyurus|[58]|France.Clipperton Island|Kyphosidae|
Sectator ocyurus|[59]|Costa Rica.Guanacaste|Kyphosidae|
Pimelodella taeniopteral|[60]|Argentina.Entre Rios|Heptapteridae|
Delturus brevis|[61]|Brazil.Minas Gerais|Loricariidae|
Delturus brevis|[62]|Brazil.Minas Gerais|Loricariidae|
Delturus brevis|[63]|Brazil.Minas Gerais|Loricariidae|
Delturus brevis|[64]|Brazil.Minas Gerais|Loricariidae|
Delturus brevis|[65]|Brazil.Minas Gerais|Loricariidae|
Delturus brevis|[66]|Brazil.Minas Gerais|Loricariidae|
Delturus brevis|[67]|Brazil.Minas Gerais|Loricariidae|
Delturus brevis|[68]|Brazil.Minas Gerais|Loricariidae|
Delturus brevis|[69]|Brazil.Minas Gerais|Loricariidae|
Delturus brevis|[70]|Brazil.Minas Gerais|Loricariidae|
Delturus brevis|[71]|Brazil.Minas Gerais|Loricariidae|
Delturus brevis|[72]|Brazil.Minas Gerais|Loricariidae|
Delturus brevis|[73]|Brazil.Minas Gerais|Loricariidae|
Delturus brevis|[74]|Brazil.Minas Gerais|Loricariidae|
Delturus brevis|[75]|Brazil.Minas Gerais|Loricariidae|
Delturus brevis|[76]|Brazil.Minas Gerais|Loricariidae|
Delturus carinotus|[77]|Brazil.Minas Gerais|Loricariidae|
Delturus carinotus|[78]|Loricariidae|
Delturus carinotus|[79]|Brazil.Minas Gerais|Loricariidae|
Delturus carinotus|[80]|Brazil.Minas Gerais|Loricariidae|
Delturus carinotus|[81]|Brazil.Minas Gerais|Loricariidae|
Delturus carinotus|[82]|Brazil.Minas Gerais|Loricariidae|
Delturus carinotus|[83]|Brazil.Minas Gerais|Loricariidae|
Delturus angulicauda|[84]|Brazil.Minas Gerais|Loricariidae|
Delturus angulicauda|[85]|Brazil.Minas Gerais|Loricariidae|
Delturus angulicauda|[86]|Brazil.Minas Gerais|Loricariidae|
Delturus angulicauda|[87]|Brazil.Minas Gerais|Loricariidae|
Delturus angulicauda|[88]|Brazil.Minas Gerais|Loricariidae|
Delturus angulicauda|[89]|Brazil.Minas Gerais|Loricariidae|
Delturus angulicauda|[90]|Brazil.Minas Gerais|Loricariidae|
Hemipsilichthys gobio|[91]|Brazil.Rio de Janeiro|Loricariidae|
Hemipsilichthys gobio|[92]|Brazil.Rio de Janeiro|Loricariidae|
Hemipsilichthys gobio|[93]|Brazil.Sao Paulo|Loricariidae|
Hemipsilichthys gobio|[94]|Brazil.Sao Paulo|Loricariidae|
Hemipsilichthys gobio|[95]|Brazil.Sao Paulo|Loricariidae|
Hemipsilichthys gobio|[96]|Brazil.Sao Paulo|Loricariidae|
Hemipsilichthys nimius|[97]|Brazil.Rio de Janeiro|Loricariidae|
Hemipsilichthys nimius|[98]|Brazil.Rio de Janeiro|Loricariidae|
Hemipsilichthys nimius|[99]|Loricariidae|
Unknown Specimen||
Hemipsilichthys nimius
Field number: TP189
Voucher: MNRJ43650

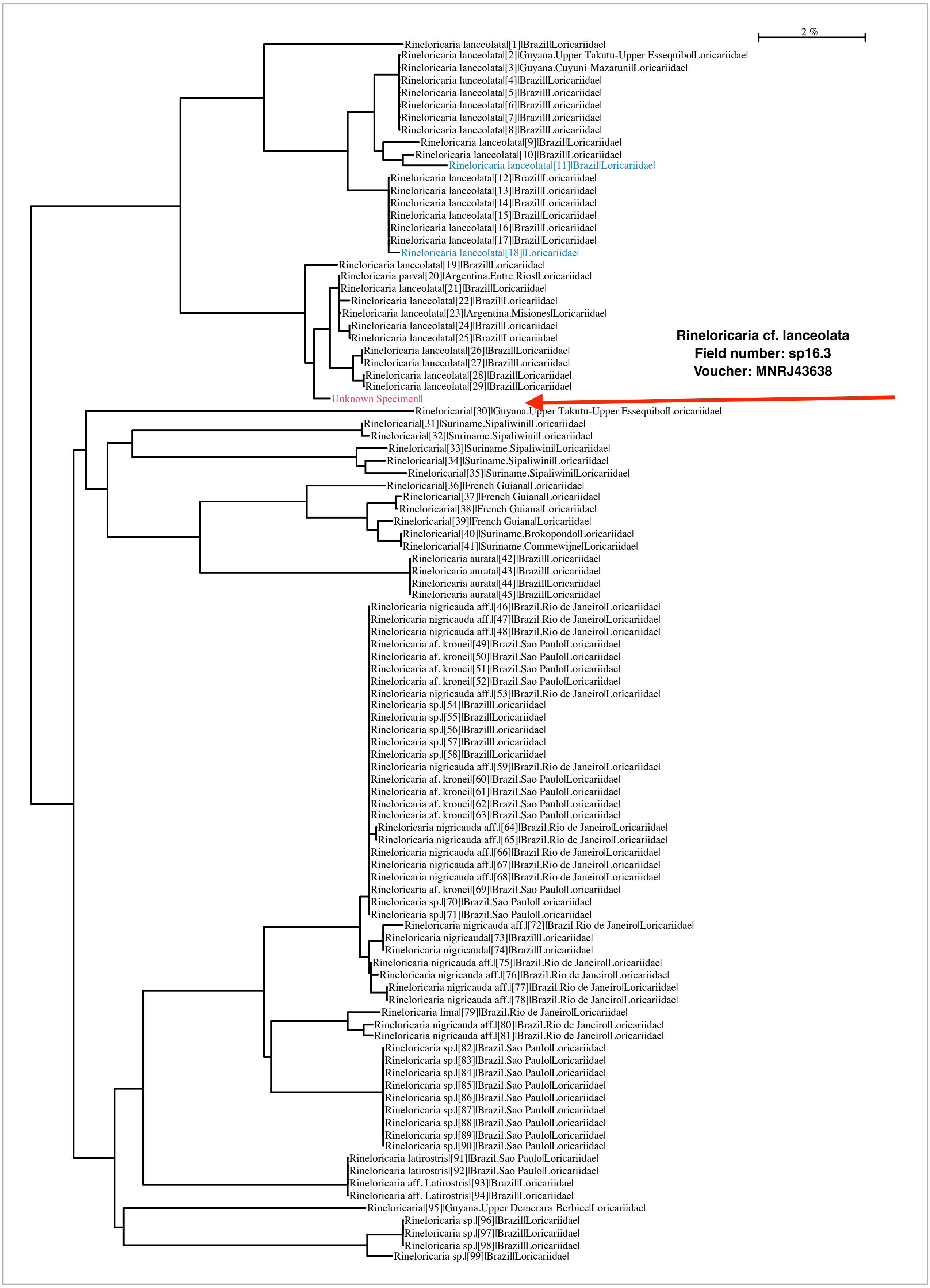
2 %
Rineloricaria lanceolata|[1]|Brazil|Loricariidae|
Rineloricaria lanceolata|[2]|Guyana.Upper Takutu-Upper Essequibo|Loricariidae|
Rineloricaria lanceolata|[3]|Guyana.Cuyuni-Mazaruni|Loricariidae|
Rineloricaria lanceolata|[4]|Brazil|Loricariidae|
Rineloricaria lanceolata|[5]|Brazil|Loricariidae|
Rineloricaria lanceolata|[6]|Brazil|Loricariidae|
Rineloricaria lanceolata|[7]|Brazil|Loricariidae|
Rineloricaria lanceolata|[8]|Brazil|Loricariidae|
Rineloricaria lanceolata|[9]|Brazil|Loricariidae|
Rineloricaria lanceolata|[10]|Brazil|Loricariidae|
Rineloricaria lanceolata|[11]|Brazil|Loricariidae|
Rineloricaria lanceolata|[12]|Brazil|Loricariidae|
Rineloricaria lanceolata|[13]|Brazil|Loricariidae|
Rineloricaria lanceolata|[14]|Brazil|Loricariidae|
Rineloricaria lanceolata|[15]|Brazil|Loricariidae|
Rineloricaria lanceolata|[16]|Brazil|Loricariidae|
Rineloricaria lanceolata|[17]|Brazil|Loricariidae|
Rineloricaria lanceolata|[18]|Loricariidae|
Rineloricaria lanceolata|[19]|Brazil|Loricariidae|
Rineloricaria parva|[20]|Argentina.Entre Rios|Loricariidae|
Rineloricaria lanceolata|[21]|Brazil|Loricariidae|
Rineloricaria lanceolata|[22]|Brazil|Loricariidae|
Rineloricaria lanceolata|[23]|Argentina.Misiones|Loricariidae|
Rineloricaria lanceolata|[24]|Brazil|Loricariidae|
Rineloricaria lanceolata|[25]|Brazil|Loricariidae|
Rineloricaria lanceolata|[26]|Brazil|Loricariidae|
Rineloricaria lanceolata|[27]|Brazil|Loricariidae|
Rineloricaria lanceolata|[28]|Brazil|Loricariidae|
Rineloricaria lanceolata|[29]|Brazil|Loricariidae|
Unknown Specimen||
Rinelocaria cf. lanceolata
Field number: sp16.3
Voucher: MNRJ43638
Rineloricaria|[30]|Guyana.Upper Takutu-Upper Essequibo|Loricariidae|
Rineloricaria|[31]|Suriname.Sipaliwini|Loricariidae|
Rineloricaria|[32]|Suriname.Sipaliwini|Loricariidae|
Rineloricaria|[33]|Suriname.Sipaliwini|Loricariidae|
Rineloricaria|[34]|Suriname.Sipaliwini|Loricariidae|
Rineloricaria|[35]|Suriname.Sipaliwini|Loricariidae|
Rineloricaria|[36]|French Guiana|Loricariidae|
Rineloricaria|[37]|French Guiana|Loricariidae|
Rineloricaria|[38]|French Guiana|Loricariidae|
Rineloricaria|[39]|French Guiana|Loricariidae|
Rineloricaria|[40]|Suriname.Brokopondo|Loricariidae|
Rineloricaria|[41]|Suriname.Commewijne|Loricariidae|
Rineloricaria aurata|[42]|Brazil|Loricariidae|
Rineloricaria aurata|[43]|Brazil|Loricariidae|
Rineloricaria aurata|[44]|Brazil|Loricariidae|
Rineloricaria aurata|[45]|Brazil|Loricariidae|
Rineloricaria nigricauda aff.|[46]|Brazil.Rio de Janeiro|Loricariidae|
Rineloricaria nigricauda aff.|[47]|Brazil.Rio de Janeiro|Loricariidae|
Rineloricaria nigricauda aff.|[48]|Brazil.Rio de Janeiro|Loricariidae|
Rineloricaria af. kronei|[49]|Brazil.Sao Paulo|Loricariidae|
Rineloricaria af. kronei|[50]|Brazil.Sao Paulo|Loricariidae|
Rineloricaria af. kronei|[51]|Brazil.Sao Paulo|Loricariidae|
Rineloricaria af. kronei|[52]|Brazil.Sao Paulo|Loricariidae|
Rineloricaria nigricauda aff.|[53]|Brazil.Rio de Janeiro|Loricariidae|
Rineloricaria sp.|[54]|Brazil|Loricariidae|
Rineloricaria sp.|[55]|Brazil|Loricariidae|
Rineloricaria sp.|[56]|Brazil|Loricariidae|
Rineloricaria sp.|[57]|Brazil|Loricariidae|
Rineloricaria sp.|[58]|Brazil|Loricariidae|
Rineloricaria nigricauda aff.|[59]|Brazil.Rio de Janeiro|Loricariidae|
Rineloricaria af. kronei|[60]|Brazil.Sao Paulo|Loricariidae|
Rineloricaria af. kronei|[61]|Brazil.Sao Paulo|Loricariidae|
Rineloricaria af. kronei|[62]|Brazil.Sao Paulo|Loricariidae|
Rineloricaria af. kronei|[63]|Brazil.Sao Paulo|Loricariidae|
Rineloricaria nigricauda aff.|[64]|Brazil.Rio de Janeiro|Loricariidae|
Rineloricaria nigricauda aff.|[65]|Brazil.Rio de Janeiro|Loricariidae|
Rineloricaria nigricauda aff.|[66]|Brazil.Rio de Janeiro|Loricariidae|
Rineloricaria nigricauda aff.|[67]|Brazil.Rio de Janeiro|Loricariidae|
Rineloricaria nigricauda aff.|[68]|Brazil.Rio de Janeiro|Loricariidae|
Rineloricaria af. kronei|[69]|Brazil.Sao Paulo|Loricariidae|
Rineloricaria sp.|[70]|Brazil.Sao Paulo|Loricariidae|
Rineloricaria sp.|[71]|Brazil.Sao Paulo|Loricariidae|
Rineloricaria nigricauda aff.|[72]|Brazil.Rio de Janeiro|Loricariidae|
Rineloricaria nigricauda|[73]|Brazil|Loricariidae|
Rineloricaria nigricauda|[74]|Brazil|Loricariidae|
Rineloricaria nigricauda aff.|[75]|Brazil.Rio de Janeiro|Loricariidae|
Rineloricaria nigricauda aff.|[76]|Brazil.Rio de Janeiro|Loricariidae|
Rineloricaria nigricauda aff.|[77]|Brazil.Rio de Janeiro|Loricariidae|
Rineloricaria nigricauda aff.|[78]|Brazil.Rio de Janeiro|Loricariidae|
Rineloricaria lima|[79]|Brazil.Rio de Janeiro|Loricariidae|
Rineloricaria nigricauda aff.|[80]|Brazil.Rio de Janeiro|Loricariidae|
Rineloricaria nigricauda aff.|[81]|Brazil.Rio de Janeiro|Loricariidae|
Rineloricaria sp.|[82]|Brazil.Sao Paulo|Loricariidae|
Rineloricaria sp.|[83]|Brazil.Sao Paulo|Loricariidae|
Rineloricaria sp.|[84]|Brazil.Sao Paulo|Loricariidae|
Rineloricaria sp.|[85]|Brazil.Sao Paulo|Loricariidae|
Rineloricaria sp.|[86]|Brazil.Sao Paulo|Loricariidae|
Rineloricaria sp.|[87]|Brazil.Sao Paulo|Loricariidae|
Rineloricaria sp.|[88]|Brazil.Sao Paulo|Loricariidae|
Rineloricaria sp.|[89]|Brazil.Sao Paulo|Loricariidae|
Rineloricaria sp.|[90]|Brazil.Sao Paulo|Loricariidae|
Rineloricaria latirostris|[91]|Brazil.Sao Paulo|Loricariidae|
Rineloricaria latirostris|[92]|Brazil.Sao Paulo|Loricariidae|
Rineloricaria aff. Latirostris|[93]|Brazil|Loricariidae|
Rineloricaria aff. Latirostris|[94]|Brazil|Loricariidae|
Rineloricaria|[95]|Guyana.Upper Demerara-Berbice|Loricariidae|
Rineloricaria sp.|[96]|Brazil|Loricariidae|
Rineloricaria sp.|[97]|Brazil|Loricariidae|
Rineloricaria sp.|[98]|Brazil|Loricariidae|
Rineloricaria sp.|[99]|Brazil|Loricariidae|

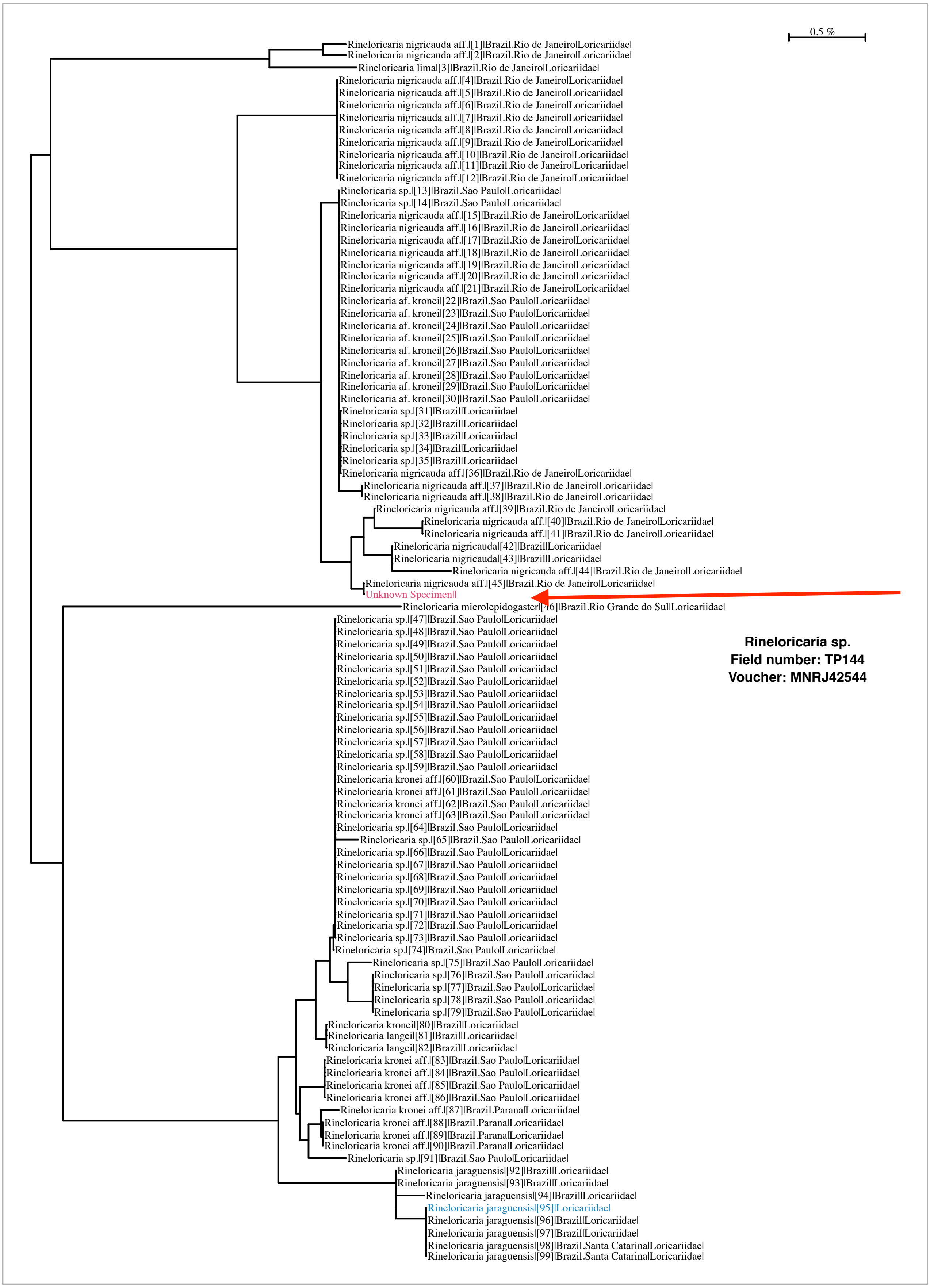
0.5 %
Rineloricaria nigricauda aff.|[1]|Brazil.Rio de Janeiro|Loricariidae|
Rineloricaria nigricauda aff.|[2]|Brazil.Rio de Janeiro|Loricariidae|
Rineloricaria lima|[3]|Brazil.Rio de Janeiro|Loricariidae|
Rineloricaria nigricauda aff.|[4]|Brazil.Rio de Janeiro|Loricariidae|
Rineloricaria nigricauda aff.|[5]|Brazil.Rio de Janeiro|Loricariidae|
Rineloricaria nigricauda aff.|[6]|Brazil.Rio de Janeiro|Loricariidae|
Rineloricaria nigricauda aff.|[7]|Brazil.Rio de Janeiro|Loricariidae|
Rineloricaria nigricauda aff.|[8]|Brazil.Rio de Janeiro|Loricariidae|
Rineloricaria nigricauda aff.|[9]|Brazil.Rio de Janeiro|Loricariidae|
Rineloricaria nigricauda aff.|[10]|Brazil.Rio de Janeiro|Loricariidae|
Rineloricaria nigricauda aff.|[11]|Brazil.Rio de Janeiro|Loricariidae|
Rineloricaria nigricauda aff.|[12]|Brazil.Rio de Janeiro|Loricariidae|
Rineloricaria sp.|[13]|Brazil.Sao Paulo|Loricariidae|
Rineloricaria sp.|[14]|Brazil.Sao Paulo|Loricariidae|
Rineloricaria nigricauda aff.|[15]|Brazil.Rio de Janeiro|Loricariidae|
Rineloricaria nigricauda aff.|[16]|Brazil.Rio de Janeiro|Loricariidae|
Rineloricaria nigricauda aff.|[17]|Brazil.Rio de Janeiro|Loricariidae|
Rineloricaria nigricauda aff.|[18]|Brazil.Rio de Janeiro|Loricariidae|
Rineloricaria nigricauda aff.|[19]|Brazil.Rio de Janeiro|Loricariidae|
Rineloricaria nigricauda aff.|[20]|Brazil.Rio de Janeiro|Loricariidae|
Rineloricaria nigricauda aff.|[21]|Brazil.Rio de Janeiro|Loricariidae|
Rineloricaria af. kronei|[22]|Brazil.Sao Paulo|Loricariidae|
Rineloricaria af. kronei|[23]|Brazil.Sao Paulo|Loricariidae|
Rineloricaria af. kronei|[24]|Brazil.Sao Paulo|Loricariidae|
Rineloricaria af. kronei|[25]|Brazil.Sao Paulo|Loricariidae|
Rineloricaria af. kronei|[26]|Brazil.Sao Paulo|Loricariidae|
Rineloricaria af. kronei|[27]|Brazil.Sao Paulo|Loricariidae|
Rineloricaria af. kronei|[28]|Brazil.Sao Paulo|Loricariidae|
Rineloricaria af. kronei|[29]|Brazil.Sao Paulo|Loricariidae|
Rineloricaria af. kronei|[30]|Brazil.Sao Paulo|Loricariidae|
Rineloricaria sp.|[31]|Brazil|Loricariidae|
Rineloricaria sp.|[32]|Brazil|Loricariidae|
Rineloricaria sp.|[33]|Brazil|Loricariidae|
Rineloricaria sp.|[34]|Brazil|Loricariidae|
Rineloricaria sp.|[35]|Brazil|Loricariidae|
Rineloricaria nigricauda aff.|[36]|Brazil.Rio de Janeiro|Loricariidae|
Rineloricaria nigricauda aff.|[37]|Brazil.Rio de Janeiro|Loricariidae|
Rineloricaria nigricauda aff.|[38]|Brazil.Rio de Janeiro|Loricariidae|
Rineloricaria nigricauda aff.|[39]|Brazil.Rio de Janeiro|Loricariidae|
Rineloricaria nigricauda aff.|[40]|Brazil.Rio de Janeiro|Loricariidae|
Rineloricaria nigricauda aff.|[41]|Brazil.Rio de Janeiro|Loricariidae|
Rineloricaria nigricauda|[42]|Brazil|Loricariidae|
Rineloricaria nigricauda|[43]|Brazil|Loricariidae|
Rineloricaria nigricauda aff.|[44]|Brazil.Rio de Janeiro|Loricariidae|
Rineloricaria nigricauda aff.|[45]|Brazil.Rio de Janeiro|Loricariidae|
Unknown Specimen||
Rineloricaria microlepidogaster|[46]|Brazil.Rio Grande do Sul|Loricariidae|
Rineloricaria sp.|[47]|Brazil.Sao Paulo|Loricariidae|
Rineloricaria sp.|[48]|Brazil.Sao Paulo|Loricariidae|
Rineloricaria sp.|[49]|Brazil.Sao Paulo|Loricariidae|
Rineloricaria sp.|[50]|Brazil.Sao Paulo|Loricariidae|
Rineloricaria sp.|[51]|Brazil.Sao Paulo|Loricariidae|
Rineloricaria sp.|[52]|Brazil.Sao Paulo|Loricariidae|
Rineloricaria sp.|[53]|Brazil.Sao Paulo|Loricariidae|
Rineloricaria sp.|[54]|Brazil.Sao Paulo|Loricariidae|
Rineloricaria sp.|[55]|Brazil.Sao Paulo|Loricariidae|
Rineloricaria sp.|[56]|Brazil.Sao Paulo|Loricariidae|
Rineloricaria sp.|[57]|Brazil.Sao Paulo|Loricariidae|
Rineloricaria sp.|[58]|Brazil.Sao Paulo|Loricariidae|
Rineloricaria sp.|[59]|Brazil.Sao Paulo|Loricariidae|
Rineloricaria kronei aff.|[60]|Brazil.Sao Paulo|Loricariidae|
Rineloricaria kronei aff.|[61]|Brazil.Sao Paulo|Loricariidae|
Rineloricaria kronei aff.|[62]|Brazil.Sao Paulo|Loricariidae|
Rineloricaria kronei aff.|[63]|Brazil.Sao Paulo|Loricariidae|
Rineloricaria sp.|[64]|Brazil.Sao Paulo|Loricariidae|
Rineloricaria sp.|[65]|Brazil.Sao Paulo|Loricariidae|
Rineloricaria sp.|[66]|Brazil.Sao Paulo|Loricariidae|
Rineloricaria sp.|[67]|Brazil.Sao Paulo|Loricariidae|
Rineloricaria sp.|[68]|Brazil.Sao Paulo|Loricariidae|
Rineloricaria sp.|[69]|Brazil.Sao Paulo|Loricariidae|
Rineloricaria sp.|[70]|Brazil.Sao Paulo|Loricariidae|
Rineloricaria sp.|[71]|Brazil.Sao Paulo|Loricariidae|
Rineloricaria sp.|[72]|Brazil.Sao Paulo|Loricariidae|
Rineloricaria sp.|[73]|Brazil.Sao Paulo|Loricariidae|
Rineloricaria sp.|[74]|Brazil.Sao Paulo|Loricariidae|
Rineloricaria sp.|[75]|Brazil.Sao Paulo|Loricariidae|
Rineloricaria sp.|[76]|Brazil.Sao Paulo|Loricariidae|
Rineloricaria sp.|[77]|Brazil.Sao Paulo|Loricariidae|
Rineloricaria sp.|[78]|Brazil.Sao Paulo|Loricariidae|
Rineloricaria sp.|[79]|Brazil.Sao Paulo|Loricariidae|
Rineloricaria kronei|[80]|Brazil|Loricariidae|
Rineloricaria langei|[81]|Brazil|Loricariidae|
Rineloricaria langei|[82]|Brazil|Loricariidae|
Rineloricaria kronei aff.|[83]|Brazil.Sao Paulo|Loricariidae|
Rineloricaria kronei aff.|[84]|Brazil.Sao Paulo|Loricariidae|
Rineloricaria kronei aff.|[85]|Brazil.Sao Paulo|Loricariidae|
Rineloricaria kronei aff.|[86]|Brazil.Sao Paulo|Loricariidae|
Rineloricaria kronei aff.|[87]|Brazil.Parana|Loricariidae|
Rineloricaria kronei aff.|[88]|Brazil.Parana|Loricariidae|
Rineloricaria kronei aff.|[89]|Brazil.Parana|Loricariidae|
Rineloricaria kronei aff.|[90]|Brazil.Parana|Loricariidae|
Rineloricaria sp.|[91]|Brazil.Sao Paulo|Loricariidae|
Rineloricaria jaraguensis|[92]|Brazil|Loricariidae|
Rineloricaria jaraguensis|[93]|Brazil|Loricariidae|
Rineloricaria jaraguensis|[94]|Brazil|Loricariidae|
Rineloricaria jaraguensis|[95]||Loricariidae|
Rineloricaria jaraguensis|[96]|Brazil|Loricariidae|
Rineloricaria jaraguensis|[97]|Brazil|Loricariidae|
Rineloricaria jaraguensis|[98]|Brazil.Santa Catarina|Loricariidae|
Rineloricaria jaraguensis|[99]|Brazil.Santa Catarina|Loricariidae|
Rineloricaria sp.
Field number: TP144
Voucher: MNRJ42544

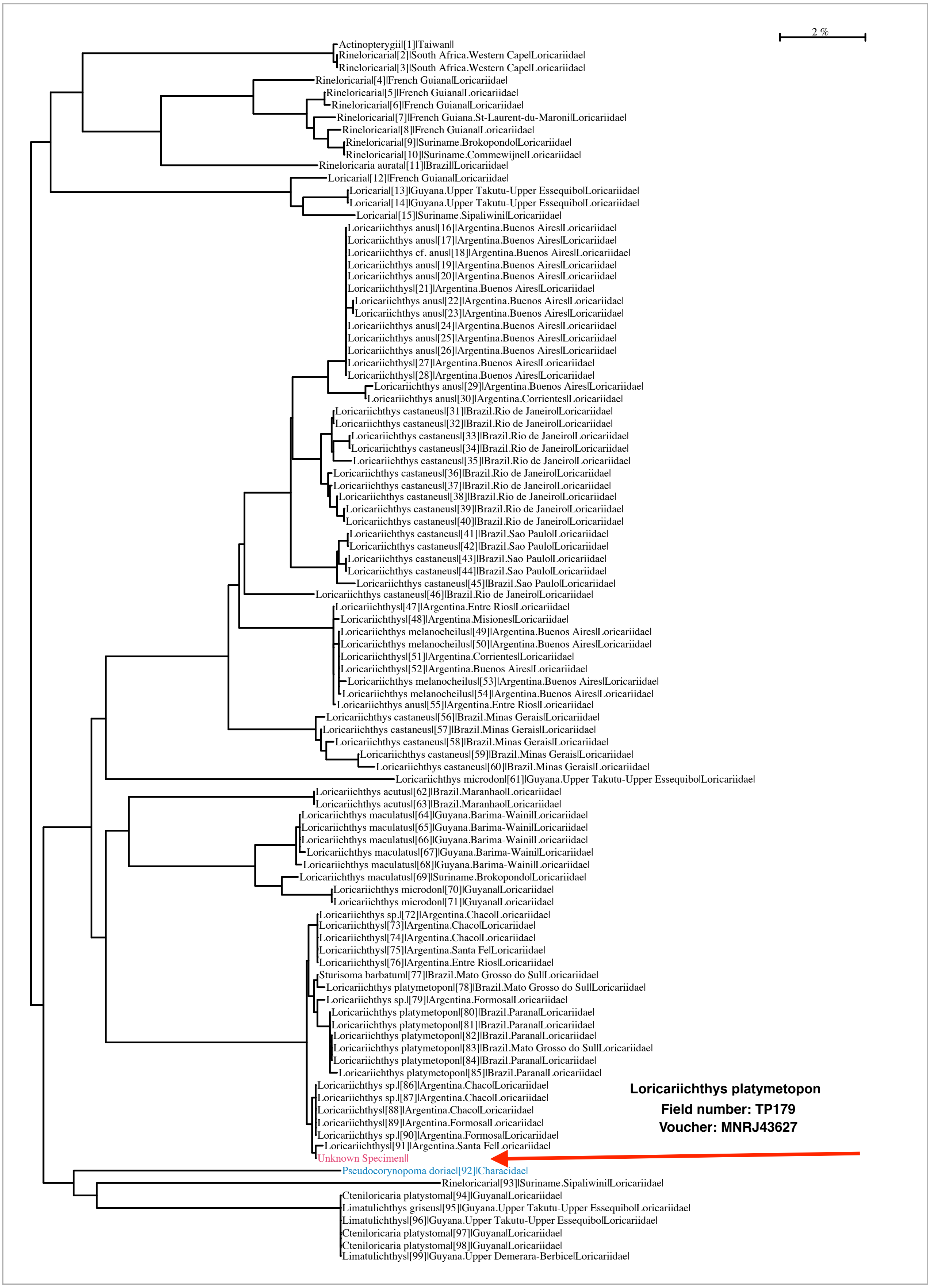
2 %
Actinopterygii|[1]|Taiwan|
Rineloricaria|[2]|South Africa.Western Cape|Loricariidae|
Rineloricaria|[3]|South Africa.Western Cape|Loricariidae|
Rineloricaria|[4]|French Guiana|Loricariidae|
Rineloricaria|[5]|French Guiana|Loricariidae|
Rineloricaria|[6]|French Guiana|Loricariidae|
Rineloricaria|[7]|French Guiana.St-Laurent-du-Maroni|Loricariidae|
Rineloricaria|[8]|French Guiana|Loricariidae|
Rineloricaria|[9]|Suriname.Brokopondo|Loricariidae|
Rineloricaria|[10]|Suriname.Commewijne|Loricariidae|
Rineloricaria aurata|[11]|Brazil|Loricariidae|
Loricaria|[12]|French Guiana|Loricariidae|
Loricaria|[13]|Guyana.Upper Takutu-Upper Essequibo|Loricariidae|
Loricaria|[14]|Guyana.Upper Takutu-Upper Essequibo|Loricariidae|
Loricaria|[15]|Suriname.Sipaliwini|Loricariidae|
Loricariichthys anus|[16]|Argentina.Buenos Aires|Loricariidae|
Loricariichthys anus|[17]|Argentina.Buenos Aires|Loricariidae|
Loricariichthys cf. anus|[18]|Argentina.Buenos Aires|Loricariidae|
Loricariichthys anus|[19]|Argentina.Buenos Aires|Loricariidae|
Loricariichthys anus|[20]|Argentina.Buenos Aires|Loricariidae|
Loricariichthys|[21]|Argentina.Buenos Aires|Loricariidae|
Loricariichthys anus|[22]|Argentina.Buenos Aires|Loricariidae|
Loricariichthys anus|[23]|Argentina.Buenos Aires|Loricariidae|
Loricariichthys anus|[24]|Argentina.Buenos Aires|Loricariidae|
Loricariichthys anus|[25]|Argentina.Buenos Aires|Loricariidae|
Loricariichthys anus|[26]|Argentina.Buenos Aires|Loricariidae|
Loricariichthys|[27]|Argentina.Buenos Aires|Loricariidae|
Loricariichthys|[28]|Argentina.Buenos Aires|Loricariidae|
Loricariichthys anus|[29]|Argentina.Buenos Aires|Loricariidae|
Loricariichthys anus|[30]|Argentina.Corrientes|Loricariidae|
Loricariichthys castaneus|[31]|Brazil.Rio de Janeiro|Loricariidae|
Loricariichthys castaneus|[32]|Brazil.Rio de Janeiro|Loricariidae|
Loricariichthys castaneus|[33]|Brazil.Rio de Janeiro|Loricariidae|
Loricariichthys castaneus|[34]|Brazil.Rio de Janeiro|Loricariidae|
Loricariichthys castaneus|[35]|Brazil.Rio de Janeiro|Loricariidae|
Loricariichthys castaneus|[36]|Brazil.Rio de Janeiro|Loricariidae|
Loricariichthys castaneus|[37]|Brazil.Rio de Janeiro|Loricariidae|
Loricariichthys castaneus|[38]|Brazil.Rio de Janeiro|Loricariidae|
Loricariichthys castaneus|[39]|Brazil.Rio de Janeiro|Loricariidae|
Loricariichthys castaneus|[40]|Brazil.Rio de Janeiro|Loricariidae|
Loricariichthys castaneus|[41]|Brazil.Sao Paulo|Loricariidae|
Loricariichthys castaneus|[42]|Brazil.Sao Paulo|Loricariidae|
Loricariichthys castaneus|[43]|Brazil.Sao Paulo|Loricariidae|
Loricariichthys castaneus|[44]|Brazil.Sao Paulo|Loricariidae|
Loricariichthys castaneus|[45]|Brazil.Sao Paulo|Loricariidae|
Loricariichthys castaneus|[46]|Brazil.Rio de Janeiro|Loricariidae|
Loricariichthys|[47]|Argentina.Entre Rios|Loricariidae|
Loricariichthys|[48]|Argentina.Misiones|Loricariidae|
Loricariichthys melanocheilus|[49]|Argentina.Buenos Aires|Loricariidae|
Loricariichthys melanocheilus|[50]|Argentina.Buenos Aires|Loricariidae|
Loricariichthys|[51]|Argentina.Corrientes|Loricariidae|
Loricariichthys|[52]|Argentina.Buenos Aires|Loricariidae|
Loricariichthys melanocheilus|[53]|Argentina.Buenos Aires|Loricariidae|
Loricariichthys melanocheilus|[54]|Argentina.Buenos Aires|Loricariidae|
Loricariichthys anus|[55]|Argentina.Entre Rios|Loricariidae|
Loricariichthys castaneus|[56]|Brazil.Minas Gerais|Loricariidae|
Loricariichthys castaneus|[57]|Brazil.Minas Gerais|Loricariidae|
Loricariichthys castaneus|[58]|Brazil.Minas Gerais|Loricariidae|
Loricariichthys castaneus|[59]|Brazil.Minas Gerais|Loricariidae|
Loricariichthys castaneus|[60]|Brazil.Minas Gerais|Loricariidae|
Loricariichthys microdon|[61]|Guyana.Upper Takutu-Upper Essequibo|Loricariidae|
Loricariichthys acutus|[62]|Brazil.Maranhao|Loricariidae|
Loricariichthys acutus|[63]|Brazil.Maranhao|Loricariidae|
Loricariichthys maculatus|[64]|Guyana.Barima-Waini|Loricariidae|
Loricariichthys maculatus|[65]|Guyana.Barima-Waini|Loricariidae|
Loricariichthys maculatus|[66]|Guyana.Barima-Waini|Loricariidae|
Loricariichthys maculatus|[67]|Guyana.Barima-Waini|Loricariidae|
Loricariichthys maculatus|[68]|Guyana.Barima-Waini|Loricariidae|
Loricariichthys maculatus|[69]|Suriname.Brokopondo|Loricariidae|
Loricariichthys microdon|[70]|Guyana|Loricariidae|
Loricariichthys microdon|[71]|Guyana|Loricariidae|
Loricariichthys sp.|[72]|Argentina.Chaco|Loricariidae|
Loricariichthys|[73]|Argentina.Chaco|Loricariidae|
Loricariichthys|[74]|Argentina.Chaco|Loricariidae|
Loricariichthys|[75]|Argentina.Santa Fe|Loricariidae|
Loricariichthys|[76]|Argentina.Entre Rios|Loricariidae|
Sturisoma barbatum|[77]|Brazil.Mato Grosso do Sul|Loricariidae|
Loricariichthys platymetopon|[78]|Brazil.Mato Grosso do Sul|Loricariidae|
Loricariichthys sp.|[79]|Argentina.Formosa|Loricariidae|
Loricariichthys platymetopon|[80]|Brazil.Parana|Loricariidae|
Loricariichthys platymetopon|[81]|Brazil.Parana|Loricariidae|
Loricariichthys platymetopon|[82]|Brazil.Parana|Loricariidae|
Loricariichthys platymetopon|[83]|Brazil.Mato Grosso do Sul|Loricariidae|
Loricariichthys platymetopon|[84]|Brazil.Parana|Loricariidae|
Loricariichthys platymetopon|[85]|Brazil.Parana|Loricariidae|
Loricariichthys sp.|[86]|Argentina.Chaco|Loricariidae|
Loricariichthys sp.|[87]|Argentina.Chaco|Loricariidae|
Loricariichthys|[88]|Argentina.Chaco|Loricariidae|
Loricariichthys|[89]|Argentina.Formosa|Loricariidae|
Loricariichthys sp.|[90]|Argentina.Formosa|Loricariidae|
Loricariichthys|[91]|Argentina.Santa Fe|Loricariidae|
Unknown Specimen||
Pseudocorynopoma doriae|[92]|Characidae|
Rineloricaria|[93]|Suriname.Sipaliwini|Loricariidae|
Cteniloricaria platystoma|[94]|Guyana|Loricariidae|
Limatulichthys griseus|[95]|Guyana.Upper Takutu-Upper Essequibo|Loricariidae|
Limatulichthys|[96]|Guyana.Upper Takutu-Upper Essequibo|Loricariidae|
Ctenilocaria platystoma|[97]|Guyana|Loricariidae|
Ctenilocaria platystoma|[98]|Guyana|Loricariidae|
Limatulichthys|[99]|Guyana.Upper Demerara-Berbice|Loricariidae|
Loricariichthys platymetopon
Field number: TP179
Voucher: MNRJ43627

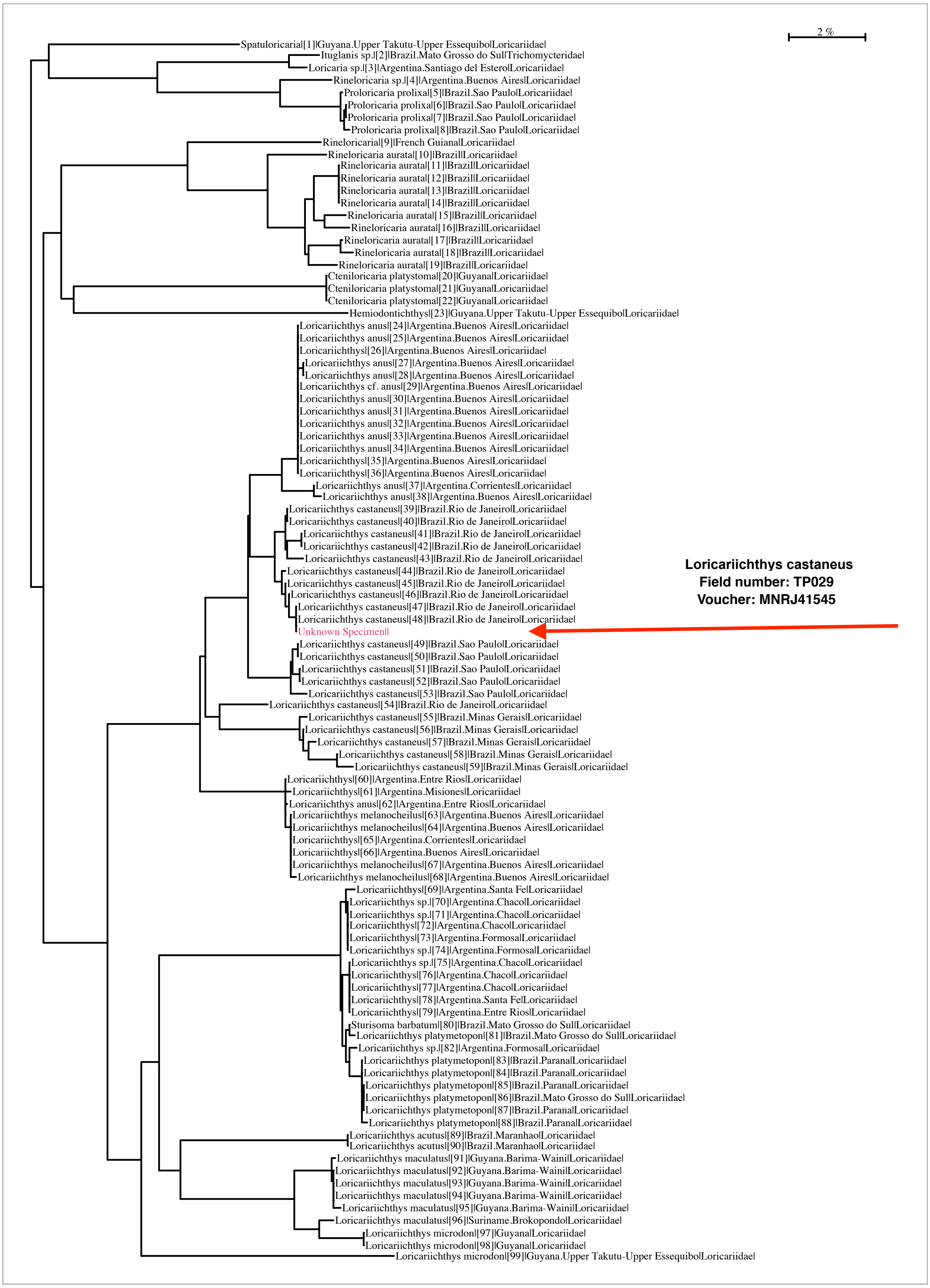
2 %
Spatuloricaria|[1]|Guyana.Upper Takutu-Upper Essequibo|Loricariidae|
Ituglanis sp.|[2]|Brazil.Mato Grosso do Sul|Trichomycteridae|
Loricaria sp.|[3]|Argentina.Santiago del Estero|Loricariidae|
Rineloricaria sp.|[4]|Argentina.Buenos Aires|Loricariidae|
Proloricaria prolixa|[5]|Brazil.Sao Paulo|Loricariidae|
Proloricaria prolixa|[6]|Brazil.Sao Paulo|Loricariidae|
Proloricaria prolixa|[7]|Brazil.Sao Paulo|Loricariidae|
Proloricaria prolixa|[8]|Brazil.Sao Paulo|Loricariidae|
Rineloricaria|[9]|French Guiana|Loricariidae|
Rineloricaria aurata|[10]|Brazil|Loricariidae|
Rineloricaria aurata|[11]|Brazil|Loricariidae|
Rineloricaria aurata|[12]|Brazil|Loricariidae|
Rineloricaria aurata|[13]|Brazil|Loricariidae|
Rineloricaria aurata|[14]|Brazil|Loricariidae|
Rineloricaria aurata|[15]|Brazil|Loricariidae|
Rineloricaria aurata|[16]|Brazil|Loricariidae|
Rineloricaria aurata|[17]|Brazil|Loricariidae|
Rineloricaria aurata|[18]|Brazil|Loricariidae|
Rineloricaria aurata|[19]|Brazil|Loricariidae|
Ctenilocaria platystoma|[20]|Guyana|Loricariidae|
Ctenilocaria platystoma|[21]|Guyana|Loricariidae|
Ctenilocaria platystoma|[22]|Guyana|Loricariidae|
Hemiodontichthys|[23]|Guyana.Upper Takutu-Upper Essequibo|Loricariidae|
Loricariichthys anus|[24]|Argentina.Buenos Aires|Loricariidae|
Loricariichthys anus|[25]|Argentina.Buenos Aires|Loricariidae|
Loricariichthys|[26]|Argentina.Buenos Aires|Loricariidae|
Loricariichthys anus|[27]|Argentina.Buenos Aires|Loricariidae|
Loricariichthys anus|[28]|Argentina.Buenos Aires|Loricariidae|
Loricariichthys cf. anus|[29]|Argentina.Buenos Aires|Loricariidae|
Loricariichthys anus|[30]|Argentina.Buenos Aires|Loricariidae|
Loricariichthys anus|[31]|Argentina.Buenos Aires|Loricariidae|
Loricariichthys anus|[32]|Argentina.Buenos Aires|Loricariidae|
Loricariichthys anus|[33]|Argentina.Buenos Aires|Loricariidae|
Loricariichthys anus|[34]|Argentina.Buenos Aires|Loricariidae|
Loricariichthys|[35]|Argentina.Buenos Aires|Loricariidae|
Loricariichthys|[36]|Argentina.Buenos Aires|Loricariidae|
Loricariichthys anus|[37]|Argentina.Corrientes|Loricariidae|
Loricariichthys anus|[38]|Argentina.Buenos Aires|Loricariidae|
Loricariichthys castaneus|[39]|Brazil.Rio de Janeiro|Loricariidae|
Loricariichthys castaneus|[40]|Brazil.Rio de Janeiro|Loricariidae|
Loricariichthys castaneus|[41]|Brazil.Rio de Janeiro|Loricariidae|
Loricariichthys castaneus|[42]|Brazil.Rio de Janeiro|Loricariidae|
Loricariichthys castaneus|[43]|Brazil.Rio de Janeiro|Loricariidae|
Loricariichthys castaneus|[44]|Brazil.Rio de Janeiro|Loricariidae|
Loricariichthys castaneus|[45]|Brazil.Rio de Janeiro|Loricariidae|
Loricariichthys castaneus|[46]|Brazil.Rio de Janeiro|Loricariidae|
Loricariichthys castaneus|[47]|Brazil.Rio de Janeiro|Loricariidae|
Loricariichthys castaneus|[48]|Brazil.Rio de Janeiro|Loricariidae|
Unknown Specimen||
Loricariichthys castaneus
Field number: TP029
Voucher: MNRJ41545
Loricariichthys castaneus|[49]|Brazil.Sao Paulo|Loricariidae|
Loricariichthys castaneus|[50]|Brazil.Sao Paulo|Loricariidae|
Loricariichthys castaneus|[51]|Brazil.Sao Paulo|Loricariidae|
Loricariichthys castaneus|[52]|Brazil.Sao Paulo|Loricariidae|
Loricariichthys castaneus|[53]|Brazil.Sao Paulo|Loricariidae|
Loricariichthys castaneus|[54]|Brazil.Rio de Janeiro|Loricariidae|
Loricariichthys castaneus|[55]|Brazil.Minas Gerais|Loricariidae|
Loricariichthys castaneus|[56]|Brazil.Minas Gerais|Loricariidae|
Loricariichthys castaneus|[57]|Brazil.Minas Gerais|Loricariidae|
Loricariichthys castaneus|[58]|Brazil.Minas Gerais|Loricariidae|
Loricariichthys castaneus|[59]|Brazil.Minas Gerais|Loricariidae|
Loricariichthys|[60]|Argentina.Entre Rios|Loricariidae|
Loricariichthys|[61]|Argentina.Misiones|Loricariidae|
Loricariichthys anus|[62]|Argentina.Entre Rios|Loricariidae|
Loricariichthys melanocheilus|[63]|Argentina.Buenos Aires|Loricariidae|
Loricariichthys melanocheilus|[64]|Argentina.Buenos Aires|Loricariidae|
Loricariichthys|[65]|Argentina.Corrientes|Loricariidae|
Loricariichthys|[66]|Argentina.Buenos Aires|Loricariidae|
Loricariichthys melanocheilus|[67]|Argentina.Buenos Aires|Loricariidae|
Loricariichthys melanocheilus|[68]|Argentina.Buenos Aires|Loricariidae|
Loricariichthys|[69]|Argentina.Santa Fe|Loricariidae|
Loricariichthys sp.|[70]|Argentina.Chaco|Loricariidae|
Loricariichthys sp.|[71]|Argentina.Chaco|Loricariidae|
Loricariichthys|[72]|Argentina.Chaco|Loricariidae|
Loricariichthys|[73]|Argentina.Formosa|Loricariidae|
Loricariichthys sp.|[74]|Argentina.Formosa|Loricariidae|
Loricariichthys sp.|[75]|Argentina.Chaco|Loricariidae|
Loricariichthys|[76]|Argentina.Chaco|Loricariidae|
Loricariichthys|[77]|Argentina.Chaco|Loricariidae|
Loricariichthys|[78]|Argentina.Santa Fe|Loricariidae|
Loricariichthys|[79]|Argentina.Entre Rios|Loricariidae|
Sturisoma barbatum|[80]|Brazil.Mato Grosso do Sul|Loricariidae|
Loricariichthys platymetopon|[81]|Brazil.Mato Grosso do Sul|Loricariidae|
Loricariichthys sp.|[82]|Argentina.Formosa|Loricariidae|
Loricariichthys platymetopon|[83]|Brazil.Parana|Loricariidae|
Loricariichthys platymetopon|[84]|Brazil.Parana|Loricariidae|
Loricariichthys platymetopon|[85]|Brazil.Parana|Loricariidae|
Loricariichthys platymetopon|[86]|Brazil.Mato Grosso do Sul|Loricariidae|
Loricariichthys platymetopon|[87]|Brazil.Parana|Loricariidae|
Loricariichthys platymetopon|[88]|Brazil.Parana|Loricariidae|
Loricariichthys acutus|[89]|Brazil.Maranhao|Loricariidae|
Loricariichthys acutus|[90]|Brazil.Maranhao|Loricariidae|
Loricariichthys maculatus|[91]|Guyana.Barima-Waini|Loricariidae|
Loricariichthys maculatus|[92]|Guyana.Barima-Waini|Loricariidae|
Loricariichthys maculatus|[93]|Guyana.Barima-Waini|Loricariidae|
Loricariichthys maculatus|[94]|Guyana.Barima-Waini|Loricariidae|
Loricariichthys maculatus|[95]|Guyana.Barima-Waini|Loricariidae|
Loricariichthys maculatus|[96]|Suriname.Brokopondo|Loricariidae|
Loricariichthys microdon|[97]|Guyana|Loricariidae|
Loricariichthys microdon|[98]|Guyana|Loricariidae|
Loricariichthys microdon|[99]|Guyana.Upper Takutu-Upper Essequibo|Loricariidae|

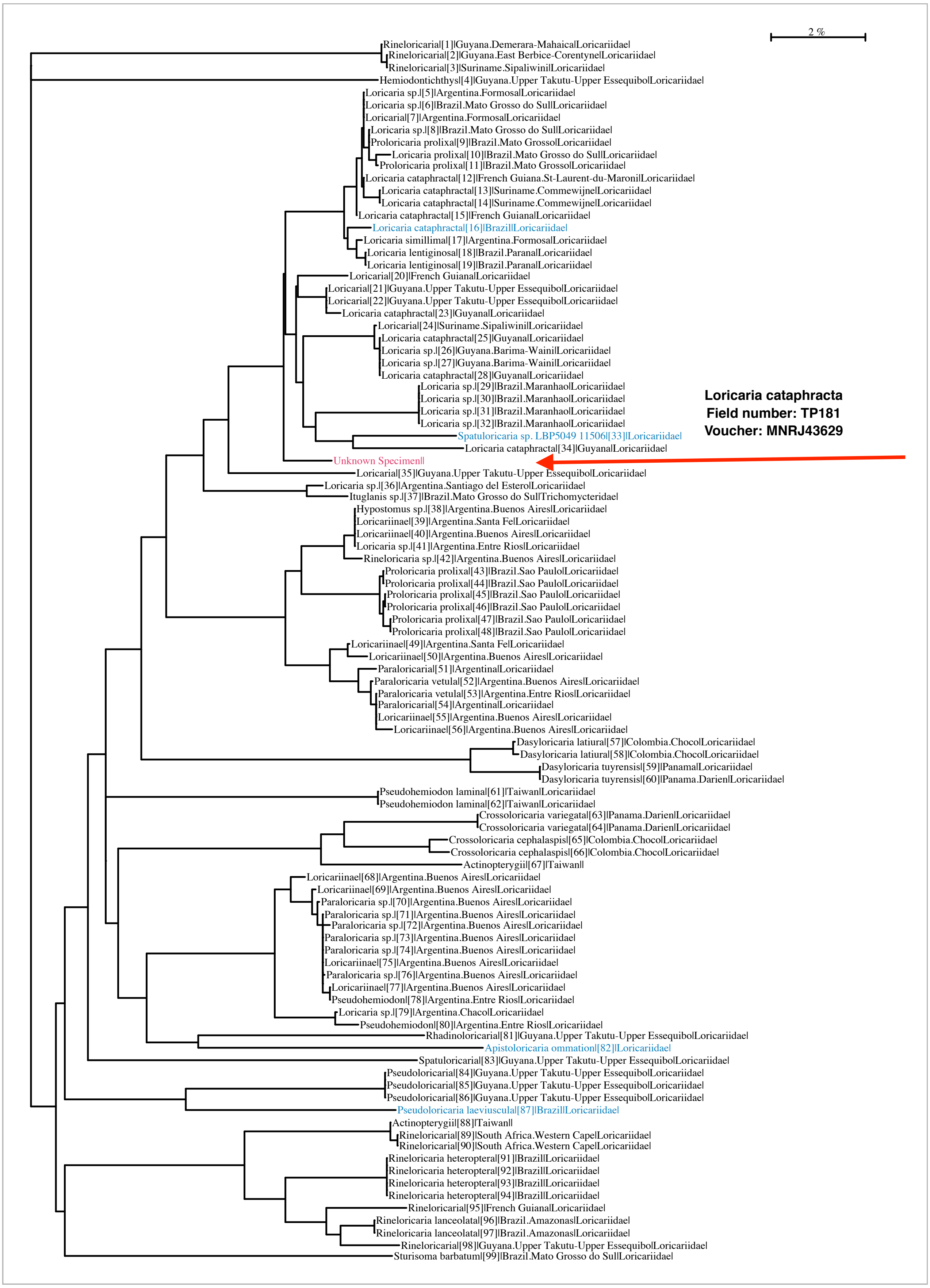
2 %
Rineloricaria|[1]|Guyana.Demerara-Mahaica|Loricariidae|
Rineloricaria|[2]|Guyana.East Berbice-Corentyne|Loricariidae|
Rineloricaria|[3]|Suriname.Sipaliwini|Loricariidae|
Hemiodontichthys|[4]|Guyana.Upper Takutu-Upper Essequibo|Loricariidae|
Loricaria sp.|[5]|Argentina.Formosa|Loricariidae|
Loricaria sp.|[6]|Brazil.Mato Grosso do Sul|Loricariidae|
Loricaria|[7]|Argentina.Formosa|Loricariidae|
Loricaria sp.|[8]|Brazil.Mato Grosso do Sul|Loricariidae|
Proloricaria prolixa|[9]|Brazil.Mato Grosso|Loricariidae|
Loricaria prolixa|[10]|Brazil.Mato Grosso do Sul|Loricariidae|
Proloricaria prolixa|[11]|Brazil.Mato Grosso|Loricariidae|
Loricaria cataphracta|[12]|French Guiana.St-Laurent-du-Maroni|Loricariidae|
Loricaria cataphracta|[13]|Suriname.Commewijne|Loricariidae|
Loricaria cataphracta|[14]|Suriname.Commewijne|Loricariidae|
Loricaria cataphracta|[15]|French Guiana|Loricariidae|
Loricaria cataphracta|[16]|Brazil|Loricariidae|
Loricaria simillima|[17]|Argentina.Formosa|Loricariidae|
Loricaria lentiginosa|[18]|Brazil.Parana|Loricariidae|
Loricaria lentiginosa|[19]|Brazil.Parana|Loricariidae|
Loricaria|[20]|French Guiana|Loricariidae|
Loricaria|[21]|Guyana.Upper Takutu-Upper Essequibo|Loricariidae|
Loricaria|[22]|Guyana.Upper Takutu-Upper Essequibo|Loricariidae|
Loricaria cataphracta|[23]|Guyana|Loricariidae|
Loricaria|[24]|Suriname.Sipaliwini|Loricariidae|
Loricaria cataphracta|[25]|Guyana|Loricariidae|
Loricaria sp.|[26]|Guyana.Barima-Waini|Loricariidae|
Loricaria sp.|[27]|Guyana.Barima-Waini|Loricariidae|
Loricaria cataphracta|[28]|Guyana|Loricariidae|
Loricaria sp.|[29]|Brazil.Maranhao|Loricariidae|
Loricaria sp.|[30]|Brazil.Maranhao|Loricariidae|
Loricaria sp.|[31]|Brazil.Maranhao|Loricariidae|
Loricaria sp.|[32]|Brazil.Maranhao|Loricariidae|
Spatuloricaria sp. LBP5049 11506|[33]|Loricariidae|
Loricaria cataphracta|[34]|Guyana|Loricariidae|
Unknown Specimen||
Loricaria|[35]|Guyana.Upper Takutu-Upper Essequibo|Loricariidae|
Loricaria sp.|[36]|Argentina.Santiago del Estero|Loricariidae|
Ituglanis sp.|[37]|Brazil.Mato Grosso do Sul|Trichomycteridae|
Hypostomus sp.|[38]|Argentina.Buenos Aires|Loricariidae|
Loricariinae|[39]|Argentina.Santa Fe|Loricariidae|
Loricariinae|[40]|Argentina.Buenos Aires|Loricariidae|
Loricaria sp.|[41]|Argentina.Entre Rios|Loricariidae|
Rineloricaria sp.|[42]|Argentina.Buenos Aires|Loricariidae|
Proloricaria prolixa|[43]|Brazil.Sao Paulo|Loricariidae|
Proloricaria prolixa|[44]|Brazil.Sao Paulo|Loricariidae|
Proloricaria prolixa|[45]|Brazil.Sao Paulo|Loricariidae|
Proloricaria prolixa|[46]|Brazil.Sao Paulo|Loricariidae|
Proloricaria prolixa|[47]|Brazil.Sao Paulo|Loricariidae|
Proloricaria prolixa|[48]|Brazil.Sao Paulo|Loricariidae|
Loricariinae|[49]|Argentina.Santa Fe|Loricariidae|
Loricariinae|[50]|Argentina.Buenos Aires|Loricariidae|
Paraloricaria|[51]|Argentina|Loricariidae|
Paraloricaria vetula|[52]|Argentina.Buenos Aires|Loricariidae|
Paraloricaria vetula|[53]|Argentina.Entre Rios|Loricariidae|
Paraloricaria|[54]|Argentina|Loricariidae|
Loricariinae|[55]|Argentina.Buenos Aires|Loricariidae|
Loricariinae|[56]|Argentina.Buenos Aires|Loricariidae|
Dasyloricaria latiura|[57]|Colombia.Choco|Loricariidae|
Dasyloricaria latiura|[58]|Colombia.Choco|Loricariidae|
Dasyloricaria tuyrensis|[59]|Panama|Loricariidae|
Dasyloricaria tuyrensis|[60]|Panama.Darien|Loricariidae|
Pseudohemiodon lamina|[61]|Taiwan|Loricariidae|
Pseudohemiodon lamina|[62]|Taiwan|Loricariidae|
Crossoloricaria variegata|[63]|Panama.Darien|Loricariidae|
Crossoloricaria variegata|[64]|Panama.Darien|Loricariidae|
Crossoloricaria cephalaspis|[65]|Colombia.Choco|Loricariidae|
Crossoloricaria cephalaspis|[66]|Colombia.Choco|Loricariidae|
Actinopterygii|[67]|Taiwan||
Loricariinae|[68]|Argentina.Buenos Aires|Loricariidae|
Loricariinae|[69]|Argentina.Buenos Aires|Loricariidae|
Paraloricaria sp.|[70]|Argentina.Buenos Aires|Loricariidae|
Paraloricaria sp.|[71]|Argentina.Buenos Aires|Loricariidae|
Paraloricaria sp.|[72]|Argentina.Buenos Aires|Loricariidae|
Paraloricaria sp.|[73]|Argentina.Buenos Aires|Loricariidae|
Paraloricaria sp.|[74]|Argentina.Buenos Aires|Loricariidae|
Loricariinae|[75]|Argentina.Buenos Aires|Loricariidae|
Paraloricaria sp.|[76]|Argentina.Buenos Aires|Loricariidae|
Loricariinae|[77]|Argentina.Buenos Aires|Loricariidae|
Pseudohemiodon|[78]|Argentina.Entre Rios|Loricariidae|
Loricaria sp.|[79]|Argentina.Chaco|Loricariidae|
Pseudohemiodon|[80]|Argentina.Entre Rios|Loricariidae|
Rhadinoloricaria|[81]|Guyana.Upper Takutu-Upper Essequibo|Loricariidae|
Apistoloricaria ommation|[82]|Loricariidae|
Spatuloricaria|[83]|Guyana.Upper Takutu-Upper Essequibo|Loricariidae|
Pseudoloricaria|[84]|Guyana.Upper Takutu-Upper Essequibo|Loricariidae|
Pseudoloricaria|[85]|Guyana.Upper Takutu-Upper Essequibo|Loricariidae|
Pseudoloricaria|[86]|Guyana.Upper Takutu-Upper Essequibo|Loricariidae|
Pseudoloricaria laeviuscula|[87]|Brazil|Loricariidae|
Actinopterygii|[88]|Taiwan||
Rineloricaria|[89]|South Africa.Western Cape|Loricariidae|
Rineloricaria|[90]|South Africa.Western Cape|Loricariidae|
Rineloricaria heteroptera|[91]|Brazil|Loricariidae|
Rineloricaria heteroptera|[92]|Brazil|Loricariidae|
Rineloricaria heteroptera|[93]|Brazil|Loricariidae|
Rineloricaria heteroptera|[94]|Brazil|Loricariidae|
Rineloricaria|[95]|French Guiana|Loricariidae|
Rineloricaria lanceolata|[96]|Brazil.Amazonas|Loricariidae|
Rineloricaria lanceolata|[97]|Brazil.Amazonas|Loricariidae|
Rineloricaria|[98]|Guyana.Upper Takutu-Upper Essequibo|Loricariidae|
Sturisoma barbatum|[99]|Brazil.Mato Grosso do Sul|Loricariidae|
Loricaria cataphracta
Field number: TP181
Voucher: MNRJ43629

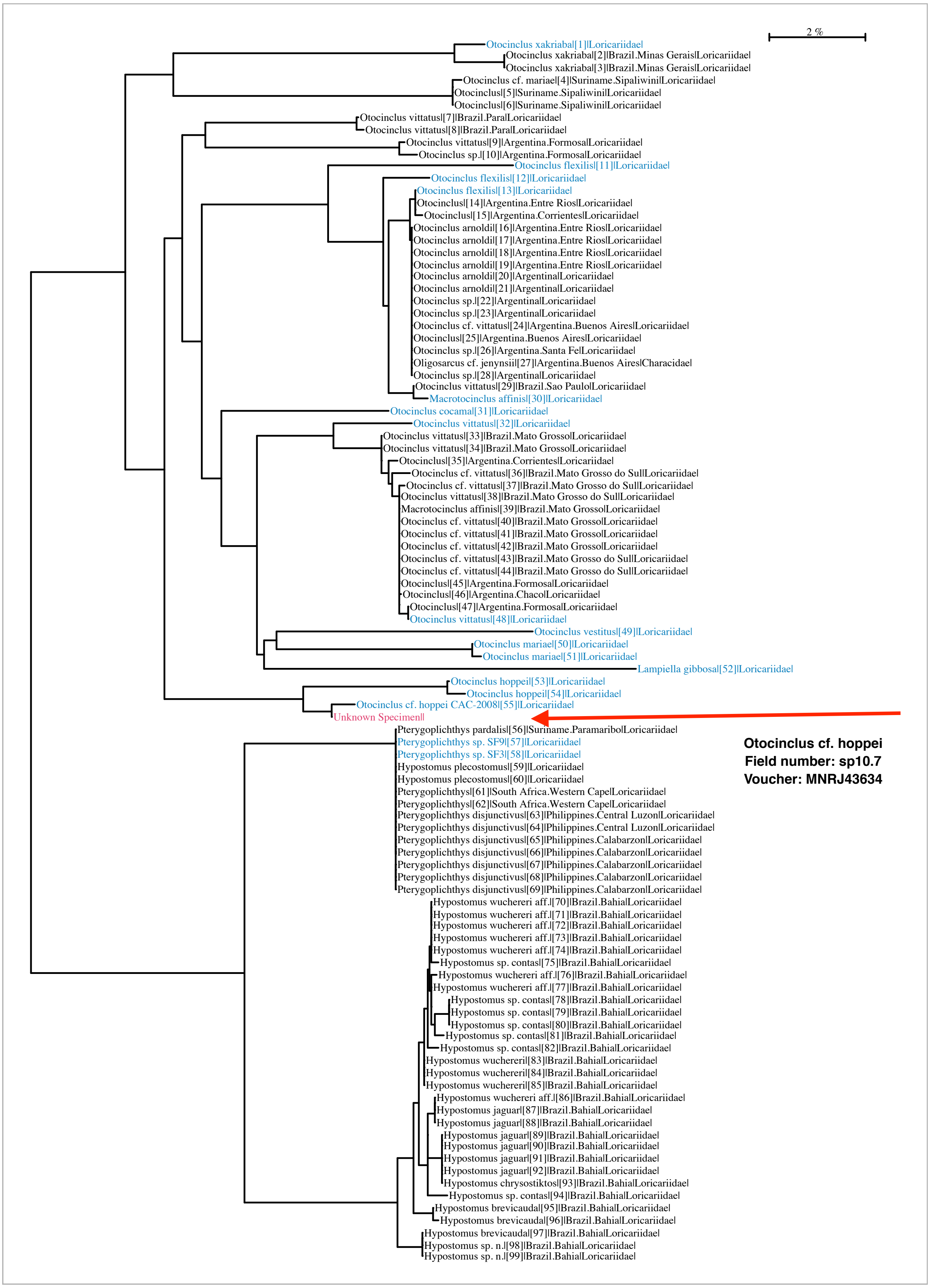
2 %
Otocinclus xakriaba|[1]|Loricariidae|
Otocinclus xakriaba|[2]|Brazil.Minas Gerais|Loricariidae|
Otocinclus xakriaba|[3]|Brazil.Minas Gerais|Loricariidae|
Otocinclus cf. mariae|[4]|Suriname.Sipaliwini|Loricariidae|
Otocinclus|[5]|Suriname.Sipaliwini|Loricariidae|
Otocinclus|[6]|Suriname.Sipaliwini|Loricariidae|
Otocinclus vittatus|[7]|Brazil.Para|Loricariidae|
Otocinclus vittatus|[8]|Brazil.Para|Loricariidae|
Otocinclus vittatus|[9]|Argentina.Formosa|Loricariidae|
Otocinclus sp.|[10]|Argentina.Formosa|Loricariidae|
Otocinclus flexilis|[11]|Loricariidae|
Otocinclus flexilis|[12]|Loricariidae|
Otocinclus flexilis|[13]|Loricariidae|
Otocinclus|[14]|Argentina.Entre Rios|Loricariidae|
Otocinclus|[15]|Argentina.Corrientes|Loricariidae|
Otocinclus arnoldi|[16]|Argentina.Entre Rios|Loricariidae|
Otocinclus arnoldi|[17]|Argentina.Entre Rios|Loricariidae|
Otocinclus arnoldi|[18]|Argentina.Entre Rios|Loricariidae|
Otocinclus arnoldi|[19]|Argentina.Entre Rios|Loricariidae|
Otocinclus arnoldi|[20]|Argentina|Loricariidae|
Otocinclus arnoldi|[21]|Argentina|Loricariidae|
Otocinclus sp.|[22]|Argentina|Loricariidae|
Otocinclus sp.|[23]|Argentina|Loricariidae|
Otocinclus cf. vittatus|[24]|Argentina.Buenos Aires|Loricariidae|
Otocinclus|[25]|Argentina.Buenos Aires|Loricariidae|
Otocinclus sp.|[26]|Argentina.Santa Fe|Loricariidae|
Oligosarcus cf. jenynsii|[27]|Argentina.Buenos Aires|Characidae|
Otocinclus sp.|[28]|Argentina|Loricariidae|
Otocinclus vittatus|[29]|Brazil.Sao Paulo|Loricariidae|
Macrotocinclus affinis|[30]|Loricariidae|
Otocinclus cocama|[31]|Loricariidae|
Otocinclus vittatus|[32]|Loricariidae|
Otocinclus vittatus|[33]|Brazil.Mato Grosso|Loricariidae|
Otocinclus vittatus|[34]|Brazil.Mato Grosso|Loricariidae|
Otocinclus|[35]|Argentina.Corrientes|Loricariidae|
Otocinclus cf. vittatus|[36]|Brazil.Mato Grosso do Sul|Loricariidae|
Otocinclus cf. vittatus|[37]|Brazil.Mato Grosso do Sul|Loricariidae|
Otocinclus vittatus|[38]|Brazil.Mato Grosso do Sul|Loricariidae|
Macrotocinclus affinis|[39]|Brazil.Mato Grosso|Loricariidae|
Otocinclus cf. vittatus|[40]|Brazil.Mato Grosso|Loricariidae|
Otocinclus cf. vittatus|[41]|Brazil.Mato Grosso|Loricariidae|
Otocinclus cf. vittatus|[42]|Brazil.Mato Grosso|Loricariidae|
Otocinclus cf. vittatus|[43]|Brazil.Mato Grosso do Sul|Loricariidae|
Otocinclus cf. vittatus|[44]|Brazil.Mato Grosso do Sul|Loricariidae|
Otocinclus|[45]|Argentina.Formosa|Loricariidae|
Otocinclus|[46]|Argentina.Chaco|Loricariidae|
Otocinclus|[47]|Argentina.Formosa|Loricariidae|
Otocinclus vittatus|[48]|Loricariidae|
Otocinclus vestitus|[49]|Loricariidae|
Otocinclus mariae|[50]|Loricariidae|
Otocinclus mariae|[51]|Loricariidae|
Lampiella gibbosa|[52]|Loricariidae|
Otocinclus hoppei|[53]|Loricariidae|
Otocinclus hoppei|[54]|Loricariidae|
Otocinclus cf. hoppei CAC-2008|[55]|Loricariidae|
Unknown Specimen||
Pterygoplichthys pardalis|[56]|Suriname.Paramaribo|Loricariidae|
Pterygoplichthys sp. SF9|[57]|Loricariidae|
Pterygoplichthys sp. SF3|[58]|Loricariidae|
Hypostomus plecostomus|[59]|Loricariidae|
Hypostomus plecostomus|[60]|Loricariidae|
Pterygoplichthys|[61]|South Africa.Western Cape|Loricariidae|
Pterygoplichthys|[62]|South Africa.Western Cape|Loricariidae|
Pterygoplichthys disjunctivus|[63]|Philippines.Central Luzon|Loricariidae|
Pterygoplichthys disjunctivus|[64]|Philippines.Central Luzon|Loricariidae|
Pterygoplichthys disjunctivus|[65]|Philippines.Calabarzon|Loricariidae|
Pterygoplichthys disjunctivus|[66]|Philippines.Calabarzon|Loricariidae|
Pterygoplichthys disjunctivus|[67]|Philippines.Calabarzon|Loricariidae|
Pterygoplichthys disjunctivus|[68]|Philippines.Calabarzon|Loricariidae|
Pterygoplichthys disjunctivus|[69]|Philippines.Calabarzon|Loricariidae|
Hypostomus wuchereri aff.|[70]|Brazil.Bahia|Loricariidae|
Hypostomus wuchereri aff.|[71]|Brazil.Bahia|Loricariidae|
Hypostomus wuchereri aff.|[72]|Brazil.Bahia|Loricariidae|
Hypostomus wuchereri aff.|[73]|Brazil.Bahia|Loricariidae|
Hypostomus wuchereri aff.|[74]|Brazil.Bahia|Loricariidae|
Hypostomus sp. contas|[75]|Brazil.Bahia|Loricariidae|
Hypostomus wuchereri aff.|[76]|Brazil.Bahia|Loricariidae|
Hypostomus wuchereri aff.|[77]|Brazil.Bahia|Loricariidae|
Hypostomus sp. contas|[78]|Brazil.Bahia|Loricariidae|
Hypostomus sp. contas|[79]|Brazil.Bahia|Loricariidae|
Hypostomus sp. contas|[80]|Brazil.Bahia|Loricariidae|
Hypostomus sp. contas|[81]|Brazil.Bahia|Loricariidae|
Hypostomus sp. contas|[82]|Brazil.Bahia|Loricariidae|
Hypostomus wuchereri|[83]|Brazil.Bahia|Loricariidae|
Hypostomus wuchereri|[84]|Brazil.Bahia|Loricariidae|
Hypostomus wuchereri|[85]|Brazil.Bahia|Loricariidae|
Hypostomus wuchereri aff.|[86]|Brazil.Bahia|Loricariidae|
Hypostomus jaguar|[87]|Brazil.Bahia|Loricariidae|
Hypostomus jaguar|[88]|Brazil.Bahia|Loricariidae|
Hypostomus jaguar|[89]|Brazil.Bahia|Loricariidae|
Hypostomus jaguar|[90]|Brazil.Bahia|Loricariidae|
Hypostomus jaguar|[91]|Brazil.Bahia|Loricariidae|
Hypostomus jaguar|[92]|Brazil.Bahia|Loricariidae|
Hypostomus chrysostiktos|[93]|Brazil.Bahia|Loricariidae|
Hypostomus sp. contas|[94]|Brazil.Bahia|Loricariidae|
Hypostomus brevicauda|[95]|Brazil.Bahia|Loricariidae|
Hypostomus brevicauda|[96]|Brazil.Bahia|Loricariidae|
Hypostomus brevicauda|[97]|Brazil.Bahia|Loricariidae|
Hypostomus sp. n.|[98]|Brazil.Bahia|Loricariidae|
Hypostomus sp. n.|[99]|Brazil.Bahia|Loricariidae|
Otocinclus cf. hoppei
Field number: sp10.7
Voucher: MNRJ43634

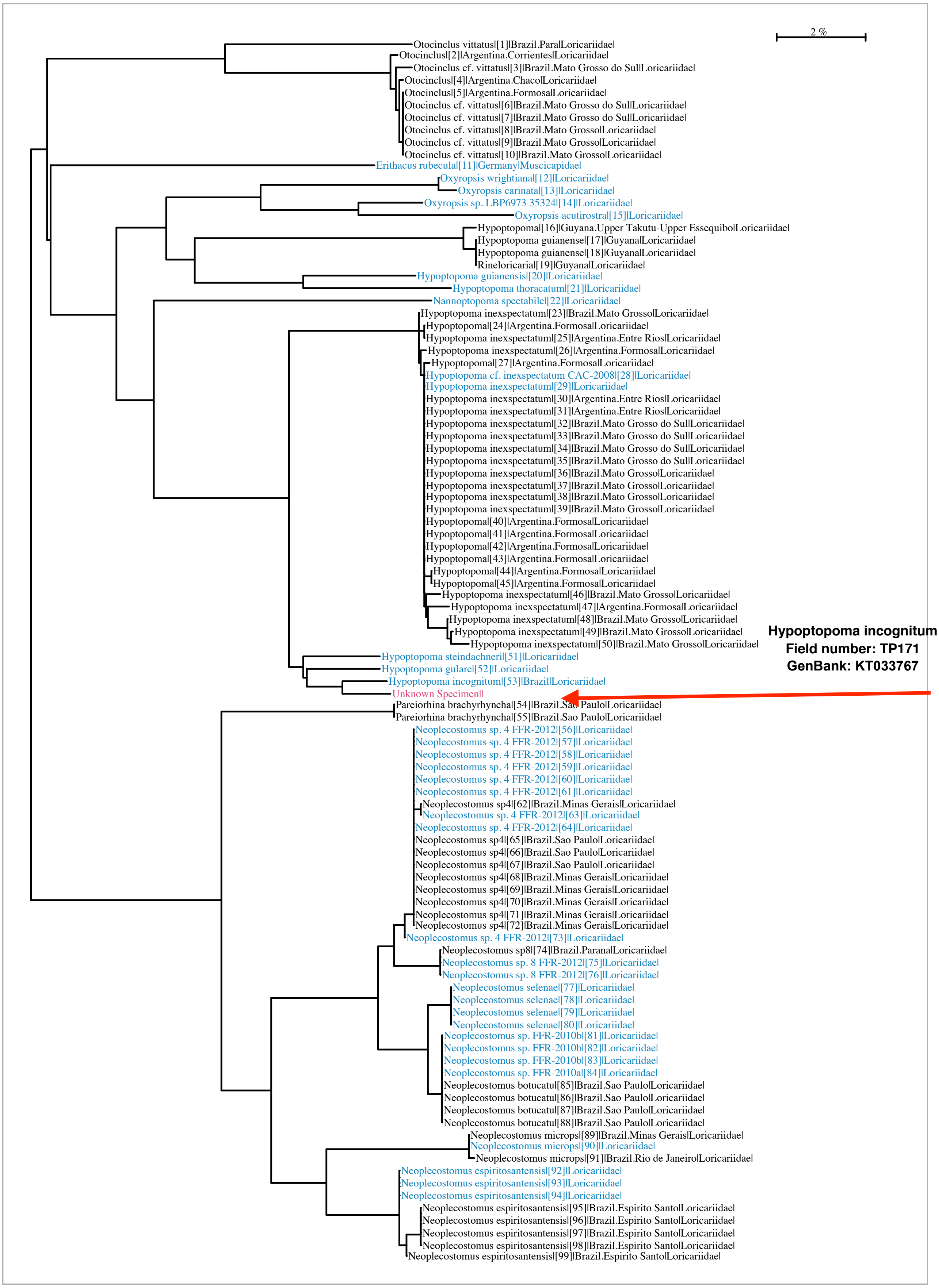
2 %
Otocinclus vittatus|[1]|Brazil.Para|Loricariidae|
Otocinclus|[2]|Argentina.Corrientes|Loricariidae|
Otocinclus cf. vittatus|[3]|Brazil.Mato Grosso do Sul|Loricariidae|
Otocinclus|[4]|Argentina.Chaco|Loricariidae|
Otocinclus|[5]|Argentina.Formosa|Loricariidae|
Otocinclus cf. vittatus|[6]|Brazil.Mato Grosso do Sul|Loricariidae|
Otocinclus cf. vittatus|[7]|Brazil.Mato Grosso do Sul|Loricariidae|
Otocinclus cf. vittatus|[8]|Brazil.Mato Grosso|Loricariidae|
Otocinclus cf. vittatus|[9]|Brazil.Mato Grosso|Loricariidae|
Otocinclus cf. vittatus|[10]|Brazil.Mato Grosso|Loricariidae|
Erithacus rubecula|[11]|Germany|Muscicapidae|
Oxyropsis wrightiana|[12]|Loricariidae|
Oxyropsis carinata|[13]|Loricariidae|
Oxyropsis sp. LBP6973 35324|[14]|Loricariidae|
Oxyropsis acutirostra|[15]|Loricariidae|
Hypoptopoma|[16]|Guyana.Upper Takutu-Upper Essequibo|Loricariidae|
Hypoptopoma guianense|[17]|Guyana|Loricariidae|
Hypoptopoma guianense|[18]|Guyana|Loricariidae|
Rineloricaria|[19]|Guyana|Loricariidae|
Hypoptopoma guianensis|[20]|Loricariidae|
Hypoptopoma thoracatum|[21]|Loricariidae|
Nannoptopoma spectabile|[22]|Loricariidae|
Hypoptopoma inexspectatum|[23]|Brazil.Mato Grosso|Loricariidae|
Hypoptopoma|[24]|Argentina.Formosa|Loricariidae|
Hypoptopoma inexspectatum|[25]|Argentina.Entre Rios|Loricariidae|
Hypoptopoma inexspectatum|[26]|Argentina.Formosa|Loricariidae|
Hypoptopoma|[27]|Argentina.Formosa|Loricariidae|
Hypoptopoma cf. inexspectatum CAC-2008|[28]|Loricariidae|
Hypoptopoma inexspectatum|[29]|Loricariidae|
Hypoptopoma inexspectatum|[30]|Argentina.Entre Rios|Loricariidae|
Hypoptopoma inexspectatum|[31]|Argentina.Entre Rios|Loricariidae|
Hypoptopoma inexspectatum|[32]|Brazil.Mato Grosso do Sul|Loricariidae|
Hypoptopoma inexspectatum|[33]|Brazil.Mato Grosso do Sul|Loricariidae|
Hypoptopoma inexspectatum|[34]|Brazil.Mato Grosso do Sul|Loricariidae|
Hypoptopoma inexspectatum|[35]|Brazil.Mato Grosso do Sul|Loricariidae|
Hypoptopoma inexspectatum|[36]|Brazil.Mato Grosso|Loricariidae|
Hypoptopoma inexspectatum|[37]|Brazil.Mato Grosso|Loricariidae|
Hypoptopoma inexspectatum|[38]|Brazil.Mato Grosso|Loricariidae|
Hypoptopoma inexspectatum|[39]|Brazil.Mato Grosso|Loricariidae|
Hypoptopoma|[40]|Argentina.Formosa|Loricariidae|
Hypoptopoma|[41]|Argentina.Formosa|Loricariidae|
Hypoptopoma|[42]|Argentina.Formosa|Loricariidae|
Hypoptopoma|[43]|Argentina.Formosa|Loricariidae|
Hypoptopoma|[44]|Argentina.Formosa|Loricariidae|
Hypoptopoma|[45]|Argentina.Formosa|Loricariidae|
Hypoptopoma inexspectatum|[46]|Brazil.Mato Grosso|Loricariidae|
Hypoptopoma inexspectatum|[47]|Argentina.Formosa|Loricariidae|
Hypoptopoma inexspectatum|[48]|Brazil.Mato Grosso|Loricariidae|
Hypoptopoma inexspectatum|[49]|Brazil.Mato Grosso|Loricariidae|
Hypoptopoma inexspectatum|[50]|Brazil.Mato Grosso|Loricariidae|
Hypoptopoma incognitum
Field number: TP171
GenBank: KT033767
Hypoptopoma steindachneri|[51]|Loricariidae|
Hypoptopoma gulare|[52]|Loricariidae|
Hypoptopoma incognitum|[53]|Brazil|Loricariidae|
Unknown Specimen|
Pareiorhina brachyrhyncha|[54]|Brazil.Sao Paulo|Loricariidae|
Pareiorhina brachyrhyncha|[55]|Brazil.Sao Paulo|Loricariidae|
Neoplecostomus sp. 4 FFR-2012|[56]|Loricariidae|
Neoplecostomus sp. 4 FFR-2012|[57]|Loricariidae|
Neoplecostomus sp. 4 FFR-2012|[58]|Loricariidae|
Neoplecostomus sp. 4 FFR-2012|[59]|Loricariidae|
Neoplecostomus sp. 4 FFR-2012|[60]|Loricariidae|
Neoplecostomus sp. 4 FFR-2012|[61]|Loricariidae|
Neoplecostomus sp4|[62]|Brazil.Minas Gerais|Loricariidae|
Neoplecostomus sp. 4 FFR-2012|[63]|Loricariidae|
Neoplecostomus sp. 4 FFR-2012|[64]|Loricariidae|
Neoplecostomus sp4|[65]|Brazil.Sao Paulo|Loricariidae|
Neoplecostomus sp4|[66]|Brazil.Sao Paulo|Loricariidae|
Neoplecostomus sp4|[67]|Brazil.Sao Paulo|Loricariidae|
Neoplecostomus sp4|[68]|Brazil.Minas Gerais|Loricariidae|
Neoplecostomus sp4|[69]|Brazil.Minas Gerais|Loricariidae|
Neoplecostomus sp4|[70]|Brazil.Minas Gerais|Loricariidae|
Neoplecostomus sp4|[71]|Brazil.Minas Gerais|Loricariidae|
Neoplecostomus sp4|[72]|Brazil.Minas Gerais|Loricariidae|
Neoplecostomus sp. 4 FFR-2012|[73]|Loricariidae|
Neoplecostomus sp8|[74]|Brazil.Parana|Loricariidae|
Neoplecostomus sp. 8 FFR-2012|[75]|Loricariidae|
Neoplecostomus sp. 8 FFR-2012|[76]|Loricariidae|
Neoplecostomus selenae|[77]|Loricariidae|
Neoplecostomus selenae|[78]|Loricariidae|
Neoplecostomus selenae|[79]|Loricariidae|
Neoplecostomus selenae|[80]|Loricariidae|
Neoplecostomus sp. FFR-2010b|[81]|Loricariidae|
Neoplecostomus sp. FFR-2010b|[82]|Loricariidae|
Neoplecostomus sp. FFR-2010b|[83]|Loricariidae|
Neoplecostomus sp. FFR-2010a|[84]|Loricariidae|
Neoplecostomus botucatu|[85]|Brazil.Sao Paulo|Loricariidae|
Neoplecostomus botucatu|[86]|Brazil.Sao Paulo|Loricariidae|
Neoplecostomus botucatu|[87]|Brazil.Sao Paulo|Loricariidae|
Neoplecostomus botucatu|[88]|Brazil.Sao Paulo|Loricariidae|
Neoplecostomus microps|[89]|Brazil.Minas Gerais|Loricariidae|
Neoplecostomus microps|[90]|Loricariidae|
Neoplecostomus microps|[91]|Brazil.Rio de Janeiro|Loricariidae|
Neoplecostomus espiritosantensis|[92]|Loricariidae|
Neoplecostomus espiritosantensis|[93]|Loricariidae|
Neoplecostomus espiritosantensis|[94]|Loricariidae|
Neoplecostomus espiritosantensis|[95]|Brazil.Espirito Santo|Loricariidae|
Neoplecostomus espiritosantensis|[96]|Brazil.Espirito Santo|Loricariidae|
Neoplecostomus espiritosantensis|[97]|Brazil.Espirito Santo|Loricariidae|
Neoplecostomus espiritosantensis|[98]|Brazil.Espirito Santo|Loricariidae|
Neoplecostomus espiritosantensis|[99]|Brazil.Espirito Santo|Loricariidae|

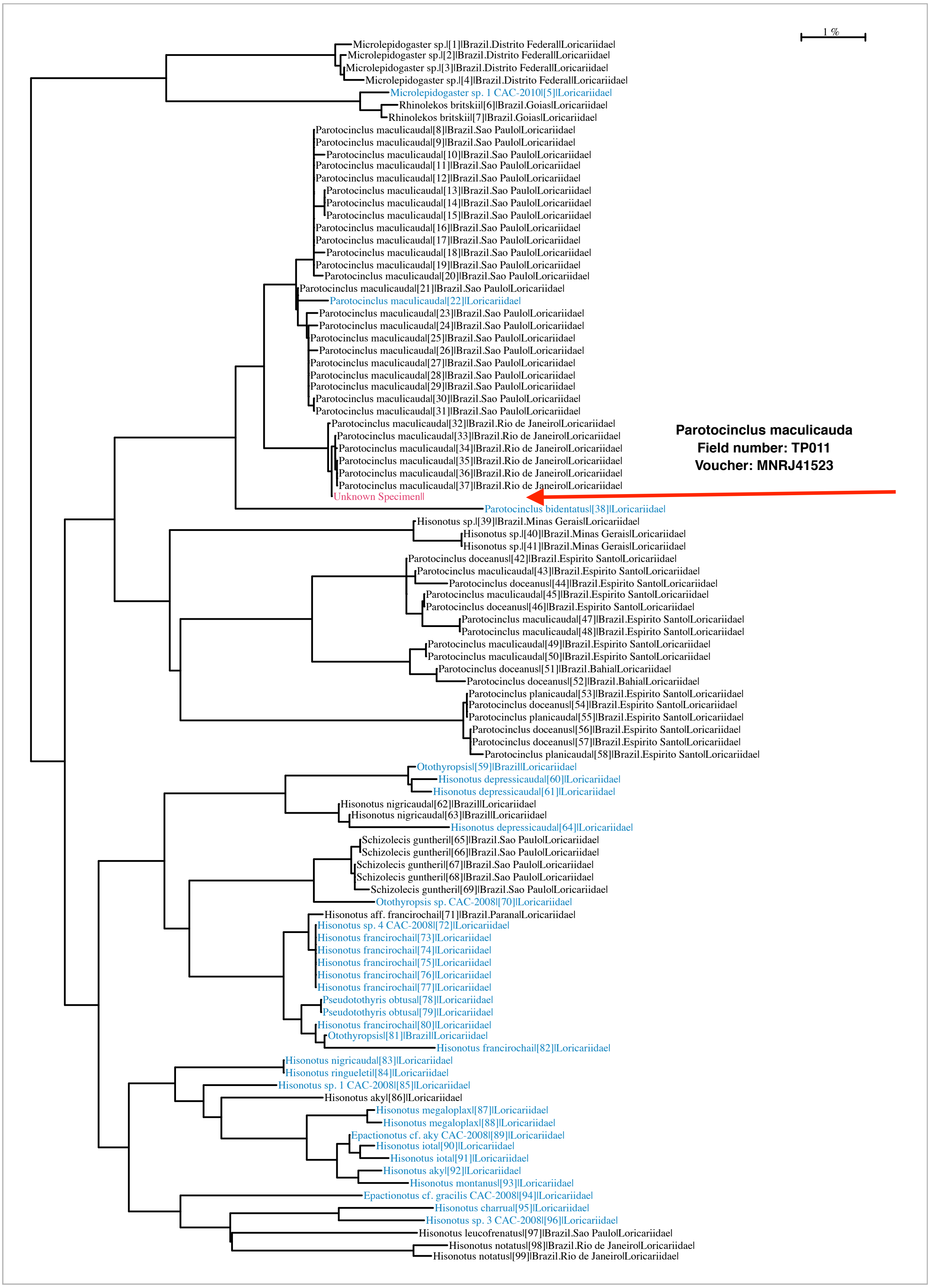
1 %
Microlepidogaster sp.|[1]|Brazil.Distrito Federal|Loricariidae|
Microlepidogaster sp.|[2]|Brazil.Distrito Federal|Loricariidae|
Microlepidogaster sp.|[3]|Brazil.Distrito Federal|Loricariidae|
Microlepidogaster sp.|[4]|Brazil.Distrito Federal|Loricariidae|
Microlepidogaster sp. 1 CAC-2010|[5]|Loricariidae|
Rhinolekos britskii|[6]|Brazil.Goias|Loricariidae|
Rhinolekos britskii|[7]|Brazil.Goias|Loricariidae|
Parotocinclus maculicauda|[8]|Brazil.Sao Paulo|Loricariidae|
Parotocinclus maculicauda|[9]|Brazil.Sao Paulo|Loricariidae|
Parotocinclus maculicauda|[10]|Brazil.Sao Paulo|Loricariidae|
Parotocinclus maculicauda|[11]|Brazil.Sao Paulo|Loricariidae|
Parotocinclus maculicauda|[12]|Brazil.Sao Paulo|Loricariidae|
Parotocinclus maculicauda|[13]|Brazil.Sao Paulo|Loricariidae|
Parotocinclus maculicauda|[14]|Brazil.Sao Paulo|Loricariidae|
Parotocinclus maculicauda|[15]|Brazil.Sao Paulo|Loricariidae|
Parotocinclus maculicauda|[16]|Brazil.Sao Paulo|Loricariidae|
Parotocinclus maculicauda|[17]|Brazil.Sao Paulo|Loricariidae|
Parotocinclus maculicauda|[18]|Brazil.Sao Paulo|Loricariidae|
Parotocinclus maculicauda|[19]|Brazil.Sao Paulo|Loricariidae|
Parotocinclus maculicauda|[20]|Brazil.Sao Paulo|Loricariidae|
Parotocinclus maculicauda|[21]|Brazil.Sao Paulo|Loricariidae|
Parotocinclus maculicauda|[22]|Loricariidae|
Parotocinclus maculicauda|[23]|Brazil.Sao Paulo|Loricariidae|
Parotocinclus maculicauda|[24]|Brazil.Sao Paulo|Loricariidae|
Parotocinclus maculicauda|[25]|Brazil.Sao Paulo|Loricariidae|
Parotocinclus maculicauda|[26]|Brazil.Sao Paulo|Loricariidae|
Parotocinclus maculicauda|[27]|Brazil.Sao Paulo|Loricariidae|
Parotocinclus maculicauda|[28]|Brazil.Sao Paulo|Loricariidae|
Parotocinclus maculicauda|[29]|Brazil.Sao Paulo|Loricariidae|
Parotocinclus maculicauda|[30]|Brazil.Sao Paulo|Loricariidae|
Parotocinclus maculicauda|[31]|Brazil.Sao Paulo|Loricariidae|
Parotocinclus maculicauda|[32]|Brazil.Rio de Janeiro|Loricariidae|
Parotocinclus maculicauda|[33]|Brazil.Rio de Janeiro|Loricariidae|
Parotocinclus maculicauda|[34]|Brazil.Rio de Janeiro|Loricariidae|
Parotocinclus maculicauda|[35]|Brazil.Rio de Janeiro|Loricariidae|
Parotocinclus maculicauda|[36]|Brazil.Rio de Janeiro|Loricariidae|
Parotocinclus maculicauda|[37]|Brazil.Rio de Janeiro|Loricariidae|
Unknown Specimen||
Parotocinclus maculicauda
Field number: TP011
Voucher: MNRJ41523
Parotocinclus bidentatus|[38]|Loricariidae|
Hisonotus sp.|[39]|Brazil.Minas Gerais|Loricariidae|
Hisonotus sp.|[40]|Brazil.Minas Gerais|Loricariidae|
Hisonotus sp.|[41]|Brazil.Minas Gerais|Loricariidae|
Parotocinclus doceanus|[42]|Brazil.Espirito Santo|Loricariidae|
Parotocinclus maculicauda|[43]|Brazil.Espirito Santo|Loricariidae|
Parotocinclus doceanus|[44]|Brazil.Espirito Santo|Loricariidae|
Parotocinclus maculicauda|[45]|Brazil.Espirito Santo|Loricariidae|
Parotocinclus doceanus|[46]|Brazil.Espirito Santo|Loricariidae|
Parotocinclus maculicauda|[47]|Brazil.Espirito Santo|Loricariidae|
Parotocinclus maculicauda|[48]|Brazil.Espirito Santo|Loricariidae|
Parotocinclus maculicauda|[49]|Brazil.Espirito Santo|Loricariidae|
Parotocinclus maculicauda|[50]|Brazil.Espirito Santo|Loricariidae|
Parotocinclus doceanus|[51]|Brazil.Bahia|Loricariidae|
Parotocinclus doceanus|[52]|Brazil.Bahia|Loricariidae|
Parotocinclus planicauda|[53]|Brazil.Espirito Santo|Loricariidae|
Parotocinclus doceanus|[54]|Brazil.Espirito Santo|Loricariidae|
Parotocinclus planicauda|[55]|Brazil.Espirito Santo|Loricariidae|
Parotocinclus doceanus|[56]|Brazil.Espirito Santo|Loricariidae|
Parotocinclus doceanus|[57]|Brazil.Espirito Santo|Loricariidae|
Parotocinclus planicauda|[58]|Brazil.Espirito Santo|Loricariidae|
Otothyropsis|[59]|Brazil|Loricariidae|
Hisonotus depressicauda|[60]|Loricariidae|
Hisonotus depressicauda|[61]|Loricariidae|
Hisonotus nigricauda|[62]|Brazil|Loricariidae|
Hisonotus nigricauda|[63]|Brazil|Loricariidae|
Hisonotus depressicauda|[64]|Loricariidae|
Schizolecis guntheri|[65]|Brazil.Sao Paulo|Loricariidae|
Schizolecis guntheri|[66]|Brazil.Sao Paulo|Loricariidae|
Schizolecis guntheri|[67]|Brazil.Sao Paulo|Loricariidae|
Schizolecis guntheri|[68]|Brazil.Sao Paulo|Loricariidae|
Schizolecis guntheri|[69]|Brazil.Sao Paulo|Loricariidae|
Otothyropsis sp. CAC-2008|[70]|Loricariidae|
Hisonotus aff. francirochai|[71]|Brazil.Parana|Loricariidae|
Hisonotus sp. 4 CAC-2008|[72]|Loricariidae|
Hisonotus francirochai|[73]|Loricariidae|
Hisonotus francirochai|[74]|Loricariidae|
Hisonotus francirochai|[75]|Loricariidae|
Hisonotus francirochai|[76]|Loricariidae|
Hisonotus francirochai|[77]|Loricariidae|
Pseudotothyris obtusa|[78]|Loricariidae|
Pseudotothyris obtusa|[79]|Loricariidae|
Hisonotus francirochai|[80]|Loricariidae|
Otothyropsis|[81]|Brazil|Loricariidae|
Hisonotus francirochai|[82]|Loricariidae|
Hisonotus nigricauda|[83]|Loricariidae|
Hisonotus ringueleti|[84]|Loricariidae|
Hisonotus sp. 1 CAC-2008|[85]|Loricariidae|
Hisonotus aky|[86]|Loricariidae|
Hisonotus megaloplax|[87]|Loricariidae|
Hisonotus megaloplax|[88]|Loricariidae|
Epactionotus cf. aky CAC-2008|[89]|Loricariidae|
Hisonotus iota|[90]|Loricariidae|
Hisonotus iota|[91]|Loricariidae|
Hisonotus aky|[92]|Loricariidae|
Hisonotus montanus|[93]|Loricariidae|
Epactionotus cf. gracilis CAC-2008|[94]|Loricariidae|
Hisonotus charrua|[95]|Loricariidae|
Hisonotus sp. 3 CAC-2008|[96]|Loricariidae|
Hisonotus leucofrenatus|[97]|Brazil.Sao Paulo|Loricariidae|
Hisonotus notatus|[98]|Brazil.Rio de Janeiro|Loricariidae|
Hisonotus notatus|[99]|Brazil.Rio de Janeiro|Loricariidae|

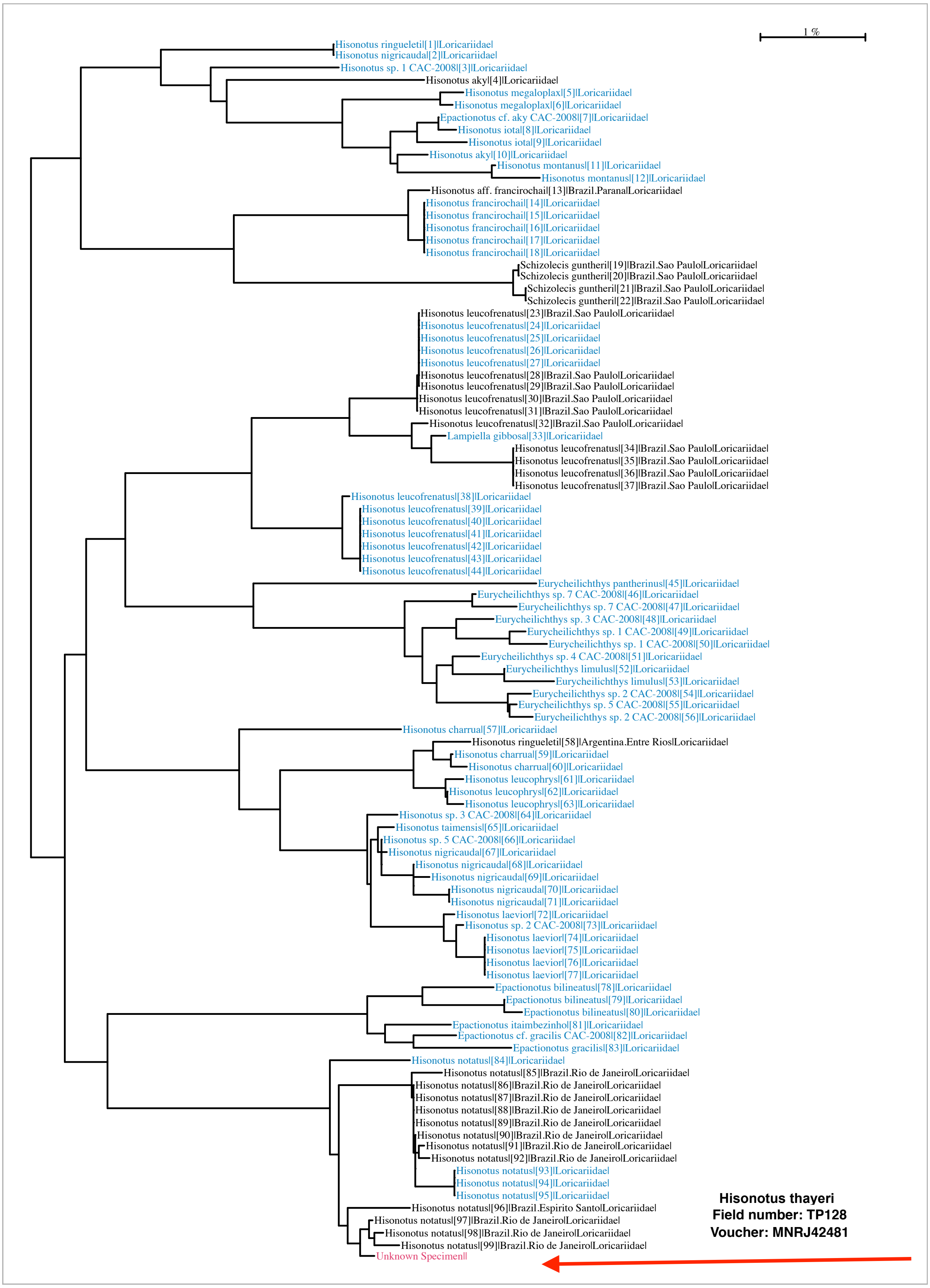
1 %
Hisonotus ringueleti|[1]||Loricariidae|
Hisonotus nigricauda|[2]||Loricariidae|
Hisonotus sp. 1 CAC-2008|[3]||Loricariidae|
Hisonotus aky|[4]||Loricariidae|
Hisonotus megaloplax|[5]||Loricariidae|
Hisonotus megaloplax|[6]||Loricariidae|
Epactionotus cf. aky CAC-2008|[7]||Loricariidae|
Hisonotus iota|[8]||Loricariidae|
Hisonotus iota|[9]||Loricariidae|
Hisonotus aky|[10]||Loricariidae|
Hisonotus montanus|[11]||Loricariidae|
Hisonotus montanus|[12]||Loricariidae|
Hisonotus aff. francirochai|[13]|Brazil.Parana|Loricariidae|
Hisonotus francirochai|[14]||Loricariidae|
Hisonotus francirochai|[15]||Loricariidae|
Hisonotus francirochai|[16]||Loricariidae|
Hisonotus francirochai|[17]||Loricariidae|
Hisonotus francirochai|[18]||Loricariidae|
Schizolecis guntheri|[19]|Brazil.Sao Paulo|Loricariidae|
Schizolecis guntheri|[20]|Brazil.Sao Paulo|Loricariidae|
Schizolecis guntheri|[21]|Brazil.Sao Paulo|Loricariidae|
Schizolecis guntheri|[22]|Brazil.Sao Paulo|Loricariidae|
Hisonotus leucofrenatus|[23]|Brazil.Sao Paulo|Loricariidae|
Hisonotus leucofrenatus|[24]||Loricariidae|
Hisonotus leucofrenatus|[25]||Loricariidae|
Hisonotus leucofrenatus|[26]||Loricariidae|
Hisonotus leucofrenatus|[27]||Loricariidae|
Hisonotus leucofrenatus|[28]|Brazil.Sao Paulo|Loricariidae|
Hisonotus leucofrenatus|[29]|Brazil.Sao Paulo|Loricariidae|
Hisonotus leucofrenatus|[30]|Brazil.Sao Paulo|Loricariidae|
Hisonotus leucofrenatus|[31]|Brazil.Sao Paulo|Loricariidae|
Hisonotus leucofrenatus|[32]|Brazil.Sao Paulo|Loricariidae|
Lampiella gibbosa|[33]||Loricariidae|
Hisonotus leucofrenatus|[34]|Brazil.Sao Paulo|Loricariidae|
Hisonotus leucofrenatus|[35]|Brazil.Sao Paulo|Loricariidae|
Hisonotus leucofrenatus|[36]|Brazil.Sao Paulo|Loricariidae|
Hisonotus leucofrenatus|[37]|Brazil.Sao Paulo|Loricariidae|
Hisonotus leucofrenatus|[38]||Loricariidae|
Hisonotus leucofrenatus|[39]||Loricariidae|
Hisonotus leucofrenatus|[40]||Loricariidae|
Hisonotus leucofrenatus|[41]||Loricariidae|
Hisonotus leucofrenatus|[42]||Loricariidae|
Hisonotus leucofrenatus|[43]||Loricariidae|
Hisonotus leucofrenatus|[44]||Loricariidae|
Eurycheilichthys pantherinus|[45]||Loricariidae|
Eurycheilichthys sp. 7 CAC-2008|[46]||Loricariidae|
Eurycheilichthys sp. 7 CAC-2008|[47]||Loricariidae|
Eurycheilichthys sp. 3 CAC-2008|[48]||Loricariidae|
Eurycheilichthys sp. 1 CAC-2008|[49]||Loricariidae|
Eurycheilichthys sp. 1 CAC-2008|[50]||Loricariidae|
Eurycheilichthys sp. 4 CAC-2008|[51]||Loricariidae|
Eurycheilichthys limulus|[52]||Loricariidae|
Eurycheilichthys limulus|[53]||Loricariidae|
Eurycheilichthys sp. 2 CAC-2008|[54]||Loricariidae|
Eurycheilichthys sp. 5 CAC-2008|[55]||Loricariidae|
Eurycheilichthys sp. 2 CAC-2008|[56]||Loricariidae|
Hisonotus charrua|[57]||Loricariidae|
Hisonotus ringueleti|[58]|Argentina.Entre Rios|Loricariidae|
Hisonotus charrua|[59]||Loricariidae|
Hisonotus charrua|[60]||Loricariidae|
Hisonotus leucophrys|[61]||Loricariidae|
Hisonotus leucophrys|[62]||Loricariidae|
Hisonotus leucophrys|[63]||Loricariidae|
Hisonotus sp. 3 CAC-2008|[64]||Loricariidae|
Hisonotus taimensis|[65]||Loricariidae|
Hisonotus sp. 5 CAC-2008|[66]||Loricariidae|
Hisonotus nigricauda|[67]||Loricariidae|
Hisonotus nigricauda|[68]||Loricariidae|
Hisonotus nigricauda|[69]||Loricariidae|
Hisonotus nigricauda|[70]||Loricariidae|
Hisonotus nigricauda|[71]||Loricariidae|
Hisonotus laevior|[72]||Loricariidae|
Hisonotus sp. 2 CAC-2008|[73]||Loricariidae|
Hisonotus laevior|[74]||Loricariidae|
Hisonotus laevior|[75]||Loricariidae|
Hisonotus laevior|[76]||Loricariidae|
Hisonotus laevior|[77]||Loricariidae|
Epactionotus bilineatus|[78]||Loricariidae|
Epactionotus bilineatus|[79]||Loricariidae|
Epactionotus bilineatus|[80]||Loricariidae|
Epactionotus itaimbezinho|[81]||Loricariidae|
Epactionotus cf. gracilis CAC-2008|[82]||Loricariidae|
Epactionotus gracilis|[83]||Loricariidae|
Hisonotus notatus|[84]||Loricariidae|
Hisonotus notatus|[85]|Brazil.Rio de Janeiro|Loricariidae|
Hisonotus notatus|[86]|Brazil.Rio de Janeiro|Loricariidae|
Hisonotus notatus|[87]|Brazil.Rio de Janeiro|Loricariidae|
Hisonotus notatus|[88]|Brazil.Rio de Janeiro|Loricariidae|
Hisonotus notatus|[89]|Brazil.Rio de Janeiro|Loricariidae|
Hisonotus notatus|[90]|Brazil.Rio de Janeiro|Loricariidae|
Hisonotus notatus|[91]|Brazil.Rio de Janeiro|Loricariidae|
Hisonotus notatus|[92]|Brazil.Rio de Janeiro|Loricariidae|
Hisonotus notatus|[93]||Loricariidae|
Hisonotus notatus|[94]||Loricariidae|
Hisonotus notatus|[95]||Loricariidae|
Hisonotus notatus|[96]|Brazil.Espirito Santo|Loricariidae|
Hisonotus notatus|[97]|Brazil.Rio de Janeiro|Loricariidae|
Hisonotus notatus|[98]|Brazil.Rio de Janeiro|Loricariidae|
Hisonotus notatus|[99]|Brazil.Rio de Janeiro|Loricariidae|
Unknown Specimen||
Hisonotus thayeri
Field number: TP128
Voucher: MNRJ42481

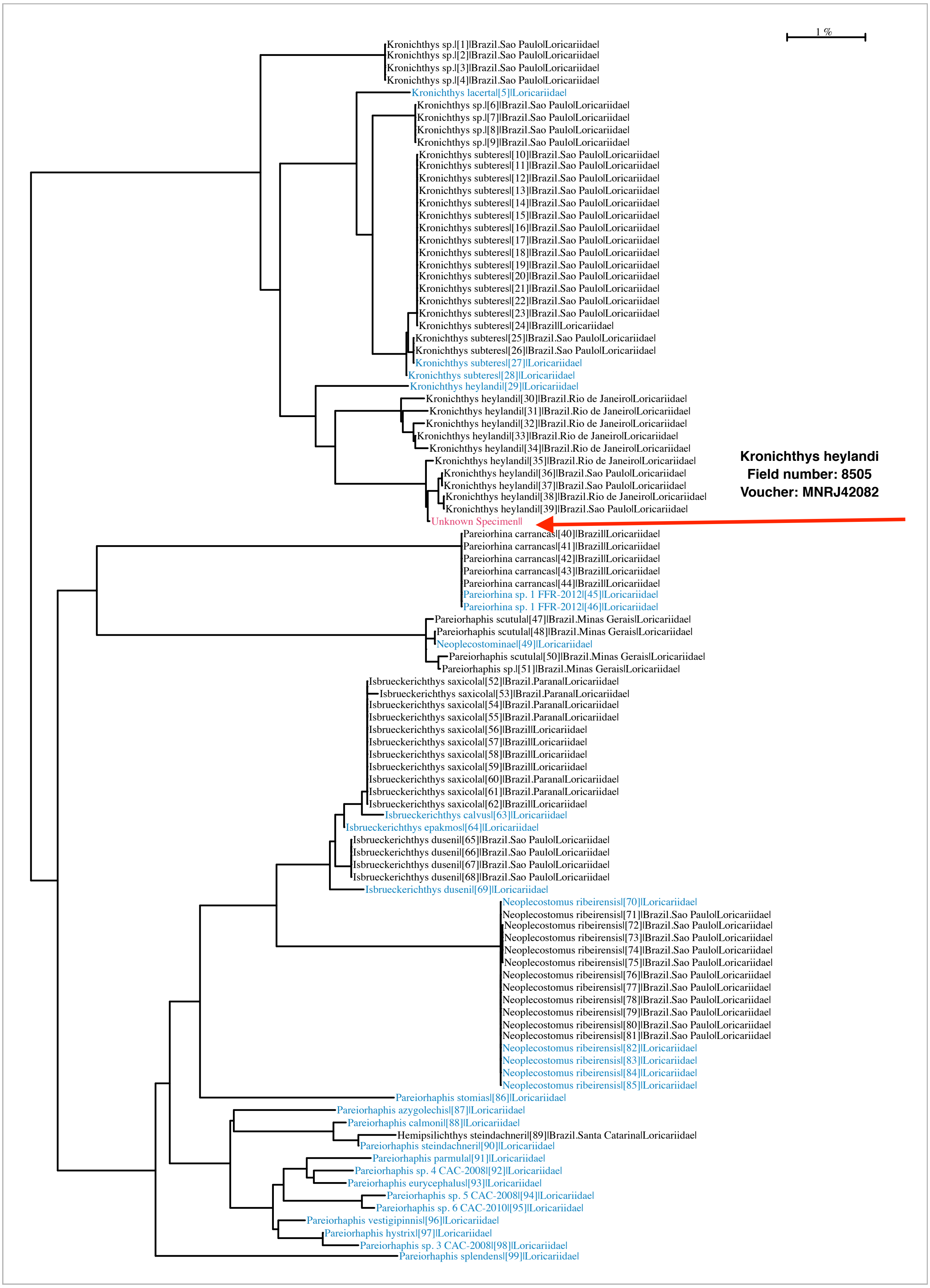
1 %
Kronichthys sp.|[1]|Brazil.Sao Paulo|Loricariidae|
Kronichthys sp.|[2]|Brazil.Sao Paulo|Loricariidae|
Kronichthys sp.|[3]|Brazil.Sao Paulo|Loricariidae|
Kronichthys sp.|[4]|Brazil.Sao Paulo|Loricariidae|
Kronichthys lacerta|[5]|Loricariidae|
Kronichthys sp.|[6]|Brazil.Sao Paulo|Loricariidae|
Kronichthys sp.|[7]|Brazil.Sao Paulo|Loricariidae|
Kronichthys sp.|[8]|Brazil.Sao Paulo|Loricariidae|
Kronichthys sp.|[9]|Brazil.Sao Paulo|Loricariidae|
Kronichthys subteres|[10]|Brazil.Sao Paulo|Loricariidae|
Kronichthys subteres|[11]|Brazil.Sao Paulo|Loricariidae|
Kronichthys subteres|[12]|Brazil.Sao Paulo|Loricariidae|
Kronichthys subteres|[13]|Brazil.Sao Paulo|Loricariidae|
Kronichthys subteres|[14]|Brazil.Sao Paulo|Loricariidae|
Kronichthys subteres|[15]|Brazil.Sao Paulo|Loricariidae|
Kronichthys subteres|[16]|Brazil.Sao Paulo|Loricariidae|
Kronichthys subteres|[17]|Brazil.Sao Paulo|Loricariidae|
Kronichthys subteres|[18]|Brazil.Sao Paulo|Loricariidae|
Kronichthys subteres|[19]|Brazil.Sao Paulo|Loricariidae|
Kronichthys subteres|[20]|Brazil.Sao Paulo|Loricariidae|
Kronichthys subteres|[21]|Brazil.Sao Paulo|Loricariidae|
Kronichthys subteres|[22]|Brazil.Sao Paulo|Loricariidae|
Kronichthys subteres|[23]|Brazil.Sao Paulo|Loricariidae|
Kronichthys subteres|[24]|Brazil|Loricariidae|
Kronichthys subteres|[25]|Brazil.Sao Paulo|Loricariidae|
Kronichthys subteres|[26]|Brazil.Sao Paulo|Loricariidae|
Kronichthys subteres|[27]|Loricariidae|
Kronichthys subteres|[28]|Loricariidae|
Kronichthys heylandi|[29]|Loricariidae|
Kronichthys heylandi|[30]|Brazil.Rio de Janeiro|Loricariidae|
Kronichthys heylandi|[31]|Brazil.Rio de Janeiro|Loricariidae|
Kronichthys heylandi|[32]|Brazil.Rio de Janeiro|Loricariidae|
Kronichthys heylandi|[33]|Brazil.Rio de Janeiro|Loricariidae|
Kronichthys heylandi|[34]|Brazil.Rio de Janeiro|Loricariidae|
Kronichthys heylandi|[35]|Brazil.Rio de Janeiro|Loricariidae|
Kronichthys heylandi|[36]|Brazil.Sao Paulo|Loricariidae|
Kronichthys heylandi|[37]|Brazil.Sao Paulo|Loricariidae|
Kronichthys heylandi|[38]|Brazil.Rio de Janeiro|Loricariidae|
Kronichthys heylandi|[39]|Brazil.Sao Paulo|Loricariidae|
Unknown Specimen||
Kronichthys heylandi
Field number: 8505
Voucher: MNRJ42082
Pareiorhina carrancas|[40]|Brazil|Loricariidae|
Pareiorhina carrancas|[41]|Brazil|Loricariidae|
Pareiorhina carrancas|[42]|Brazil|Loricariidae|
Pareiorhina carrancas|[43]|Brazil|Loricariidae|
Pareiorhina carrancas|[44]|Brazil|Loricariidae|
Pareiorhina sp. 1 FFR-2012|[45]|Loricariidae|
Pareiorhina sp. 1 FFR-2012|[46]|Loricariidae|
Pareiorhaphis scutula|[47]|Brazil.Minas Gerais|Loricariidae|
Pareiorhaphis scutula|[48]|Brazil.Minas Gerais|Loricariidae|
Neoplecostominae|[49]|Loricariidae|
Pareiorhaphis scutula|[50]|Brazil.Minas Gerais|Loricariidae|
Pareiorhaphis sp.|[51]|Brazil.Minas Gerais|Loricariidae|
Isbrueckerichthys saxicola|[52]|Brazil.Parana|Loricariidae|
Isbrueckerichthys saxicola|[53]|Brazil.Parana|Loricariidae|
Isbrueckerichthys saxicola|[54]|Brazil.Parana|Loricariidae|
Isbrueckerichthys saxicola|[55]|Brazil.Parana|Loricariidae|
Isbrueckerichthys saxicola|[56]|Brazil|Loricariidae|
Isbrueckerichthys saxicola|[57]|Brazil|Loricariidae|
Isbrueckerichthys saxicola|[58]|Brazil|Loricariidae|
Isbrueckerichthys saxicola|[59]|Brazil|Loricariidae|
Isbrueckerichthys saxicola|[60]|Brazil.Parana|Loricariidae|
Isbrueckerichthys saxicola|[61]|Brazil.Parana|Loricariidae|
Isbrueckerichthys saxicola|[62]|Brazil|Loricariidae|
Isbrueckerichthys calvus|[63]|Loricariidae|
Isbrueckerichthys epakmos|[64]|Loricariidae|
Isbrueckerichthys duseni|[65]|Brazil.Sao Paulo|Loricariidae|
Isbrueckerichthys duseni|[66]|Brazil.Sao Paulo|Loricariidae|
Isbrueckerichthys duseni|[67]|Brazil.Sao Paulo|Loricariidae|
Isbrueckerichthys duseni|[68]|Brazil.Sao Paulo|Loricariidae|
Isbrueckerichthys duseni|[69]|Loricariidae|
Neoplecostomus ribeirensis|[70]|Loricariidae|
Neoplecostomus ribeirensis|[71]|Brazil.Sao Paulo|Loricariidae|
Neoplecostomus ribeirensis|[72]|Brazil.Sao Paulo|Loricariidae|
Neoplecostomus ribeirensis|[73]|Brazil.Sao Paulo|Loricariidae|
Neoplecostomus ribeirensis|[74]|Brazil.Sao Paulo|Loricariidae|
Neoplecostomus ribeirensis|[75]|Brazil.Sao Paulo|Loricariidae|
Neoplecostomus ribeirensis|[76]|Brazil.Sao Paulo|Loricariidae|
Neoplecostomus ribeirensis|[77]|Brazil.Sao Paulo|Loricariidae|
Neoplecostomus ribeirensis|[78]|Brazil.Sao Paulo|Loricariidae|
Neoplecostomus ribeirensis|[79]|Brazil.Sao Paulo|Loricariidae|
Neoplecostomus ribeirensis|[80]|Brazil.Sao Paulo|Loricariidae|
Neoplecostomus ribeirensis|[81]|Brazil.Sao Paulo|Loricariidae|
Neoplecostomus ribeirensis|[82]|Loricariidae|
Neoplecostomus ribeirensis|[83]|Loricariidae|
Neoplecostomus ribeirensis|[84]|Loricariidae|
Neoplecostomus ribeirensis|[85]|Loricariidae|
Pareiorhaphis stomias|[86]|Loricariidae|
Pareiorhaphis azygolechis|[87]|Loricariidae|
Pareiorhaphis calmoni|[88]|Loricariidae|
Hemipsilichthys steindachneri|[89]|Brazil.Santa Catarina|Loricariidae|
Pareiorhaphis steindachneri|[90]|Loricariidae|
Pareiorhaphis parmula|[91]|Loricariidae|
Pareiorhaphis sp. 4 CAC-2008|[92]|Loricariidae|
Pareiorhaphis eurycephalus|[93]|Loricariidae|
Pareiorhaphis sp. 5 CAC-2008|[94]|Loricariidae|
Pareiorhaphis sp. 6 CAC-2010|[95]|Loricariidae|
Pareiorhaphis vestigipinnis|[96]|Loricariidae|
Pareiorhaphis hystrix|[97]|Loricariidae|
Pareiorhaphis sp. 3 CAC-2008|[98]|Loricariidae|
Pareiorhaphis splendens|[99]|Loricariidae|

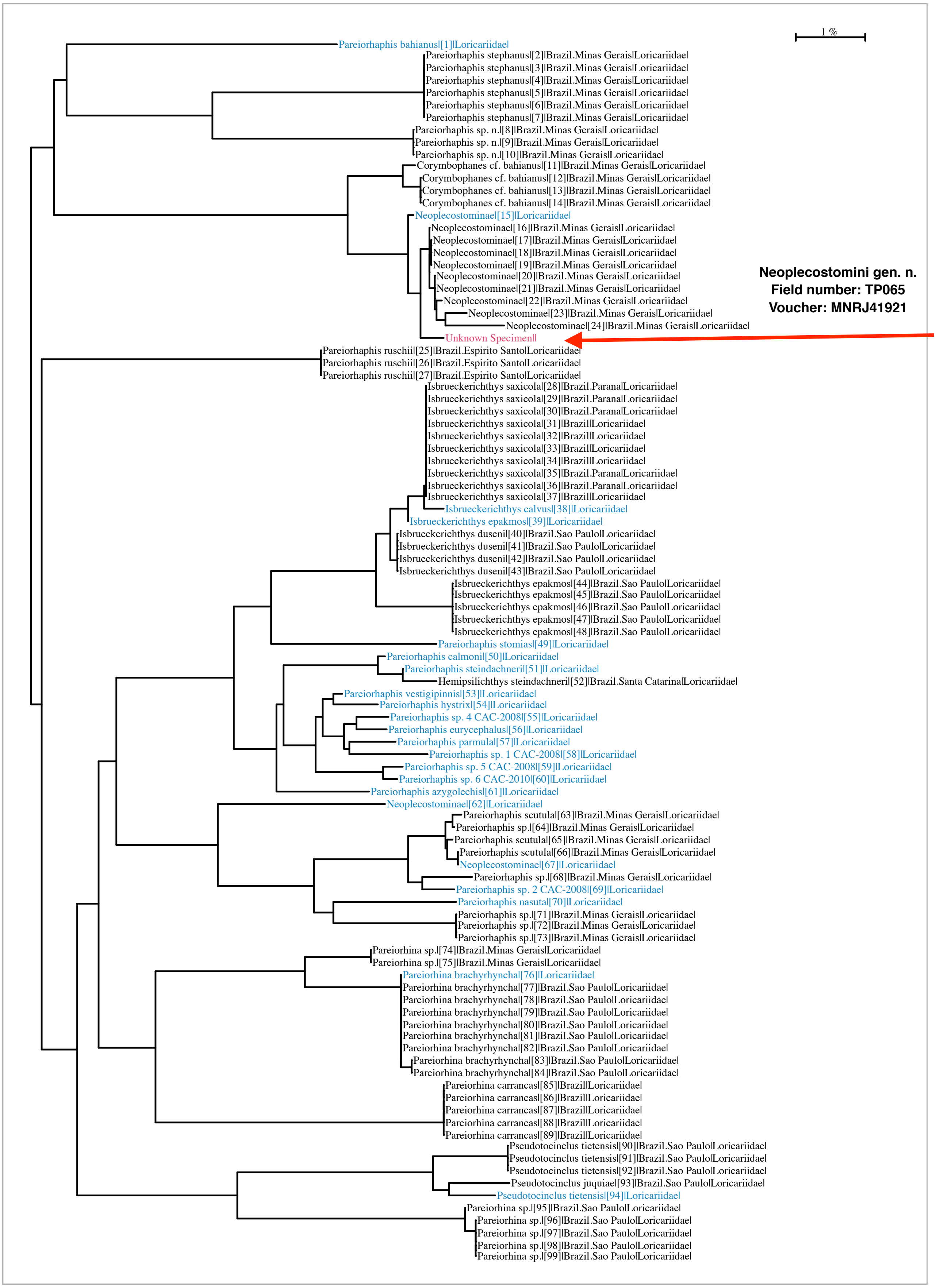
1 %
Pareiorhaphis bahianus|[1]|Loricariidae|
Pareiorhaphis stephanus|[2]|Brazil.Minas Gerais|Loricariidae|
Pareiorhaphis stephanus|[3]|Brazil.Minas Gerais|Loricariidae|
Pareiorhaphis stephanus|[4]|Brazil.Minas Gerais|Loricariidae|
Pareiorhaphis stephanus|[5]|Brazil.Minas Gerais|Loricariidae|
Pareiorhaphis stephanus|[6]|Brazil.Minas Gerais|Loricariidae|
Pareiorhaphis stephanus|[7]|Brazil.Minas Gerais|Loricariidae|
Pareiorhaphis sp. n.|[8]|Brazil.Minas Gerais|Loricariidae|
Pareiorhaphis sp. n.|[9]|Brazil.Minas Gerais|Loricariidae|
Pareiorhaphis sp. n.|[10]|Brazil.Minas Gerais|Loricariidae|
Corymbophanes cf. bahianus|[11]|Brazil.Minas Gerais|Loricariidae|
Corymbophanes cf. bahianus|[12]|Brazil.Minas Gerais|Loricariidae|
Corymbophanes cf. bahianus|[13]|Brazil.Minas Gerais|Loricariidae|
Corymbophanes cf. bahianus|[14]|Brazil.Minas Gerais|Loricariidae|
Neoplecostominae|[15]|Loricariidae|
Neoplecostominae|[16]|Brazil.Minas Gerais|Loricariidae|
Neoplecostominae|[17]|Brazil.Minas Gerais|Loricariidae|
Neoplecostominae|[18]|Brazil.Minas Gerais|Loricariidae|
Neoplecostominae|[19]|Brazil.Minas Gerais|Loricariidae|
Neoplecostominae|[20]|Brazil.Minas Gerais|Loricariidae|
Neoplecostominae|[21]|Brazil.Minas Gerais|Loricariidae|
Neoplecostominae|[22]|Brazil.Minas Gerais|Loricariidae|
Neoplecostominae|[23]|Brazil.Minas Gerais|Loricariidae|
Neoplecostominae|[24]|Brazil.Minas Gerais|Loricariidae|
Neoplecostomini gen. n.
Field number: TP065
Voucher: MNRJ41921
Unknown Specimen||
Pareiorhaphis ruschii|[25]|Brazil.Espirito Santo|Loricariidae|
Pareiorhaphis ruschii|[26]|Brazil.Espirito Santo|Loricariidae|
Pareiorhaphis ruschii|[27]|Brazil.Espirito Santo|Loricariidae|
Isbrueckerichthys saxicola|[28]|Brazil.Parana|Loricariidae|
Isbrueckerichthys saxicola|[29]|Brazil.Parana|Loricariidae|
Isbrueckerichthys saxicola|[30]|Brazil.Parana|Loricariidae|
Isbrueckerichthys saxicola|[31]|Brazil|Loricariidae|
Isbrueckerichthys saxicola|[32]|Brazil|Loricariidae|
Isbrueckerichthys saxicola|[33]|Brazil|Loricariidae|
Isbrueckerichthys saxicola|[34]|Brazil|Loricariidae|
Isbrueckerichthys saxicola|[35]|Brazil.Parana|Loricariidae|
Isbrueckerichthys saxicola|[36]|Brazil.Parana|Loricariidae|
Isbrueckerichthys saxicola|[37]|Brazil|Loricariidae|
Isbrueckerichthys calvus|[38]|Loricariidae|
Isbrueckerichthys epakmos|[39]|Loricariidae|
Isbrueckerichthys duseni|[40]|Brazil.Sao Paulo|Loricariidae|
Isbrueckerichthys duseni|[41]|Brazil.Sao Paulo|Loricariidae|
Isbrueckerichthys duseni|[42]|Brazil.Sao Paulo|Loricariidae|
Isbrueckerichthys duseni|[43]|Brazil.Sao Paulo|Loricariidae|
Isbrueckerichthys epakmos|[44]|Brazil.Sao Paulo|Loricariidae|
Isbrueckerichthys epakmos|[45]|Brazil.Sao Paulo|Loricariidae|
Isbrueckerichthys epakmos|[46]|Brazil.Sao Paulo|Loricariidae|
Isbrueckerichthys epakmos|[47]|Brazil.Sao Paulo|Loricariidae|
Isbrueckerichthys epakmos|[48]|Brazil.Sao Paulo|Loricariidae|
Pareiorhaphis stomias|[49]|Loricariidae|
Pareiorhaphis calmoni|[50]|Loricariidae|
Pareiorhaphis steindachneri|[51]|Loricariidae|
Hemipsilichthys steindachneri|[52]|Brazil.Santa Catarina|Loricariidae|
Pareiorhaphis vestigipinnis|[53]|Loricariidae|
Pareiorhaphis hystrix|[54]|Loricariidae|
Pareiorhaphis sp. 4 CAC-2008|[55]|Loricariidae|
Pareiorhaphis eurycephalus|[56]|Loricariidae|
Pareiorhaphis parmula|[57]|Loricariidae|
Pareiorhaphis sp. 1 CAC-2008|[58]|Loricariidae|
Pareiorhaphis sp. 5 CAC-2008|[59]|Loricariidae|
Pareiorhaphis sp. 6 CAC-2010|[60]|Loricariidae|
Pareiorhaphis azygolechis|[61]|Loricariidae|
Neoplecostominae|[62]|Loricariidae|
Pareiorhaphis scutula|[63]|Brazil.Minas Gerais|Loricariidae|
Pareiorhaphis sp.|[64]|Brazil.Minas Gerais|Loricariidae|
Pareiorhaphis scutula|[65]|Brazil.Minas Gerais|Loricariidae|
Pareiorhaphis scutula|[66]|Brazil.Minas Gerais|Loricariidae|
Neoplecostominae|[67]|Loricariidae|
Pareiorhaphis sp.|[68]|Brazil.Minas Gerais|Loricariidae|
Pareiorhaphis sp. 2 CAC-2008|[69]|Loricariidae|
Pareiorhaphis nasuta|[70]|Loricariidae|
Pareiorhaphis sp.|[71]|Brazil.Minas Gerais|Loricariidae|
Pareiorhaphis sp.|[72]|Brazil.Minas Gerais|Loricariidae|
Pareiorhaphis sp.|[73]|Brazil.Minas Gerais|Loricariidae|
Pareiorhina sp.|[74]|Brazil.Minas Gerais|Loricariidae|
Pareiorhina sp.|[75]|Brazil.Minas Gerais|Loricariidae|
Pareiorhina brachyrhyncha|[76]|Loricariidae|
Pareiorhina brachyrhyncha|[77]|Brazil.Sao Paulo|Loricariidae|
Pareiorhina brachyrhyncha|[78]|Brazil.Sao Paulo|Loricariidae|
Pareiorhina brachyrhyncha|[79]|Brazil.Sao Paulo|Loricariidae|
Pareiorhina brachyrhyncha|[80]|Brazil.Sao Paulo|Loricariidae|
Pareiorhina brachyrhyncha|[81]|Brazil.Sao Paulo|Loricariidae|
Pareiorhina brachyrhyncha|[82]|Brazil.Sao Paulo|Loricariidae|
Pareiorhina brachyrhyncha|[83]|Brazil.Sao Paulo|Loricariidae|
Pareiorhina brachyrhyncha|[84]|Brazil.Sao Paulo|Loricariidae|
Pareiorhina carrancas|[85]|Brazil|Loricariidae|
Pareiorhina carrancas|[86]|Brazil|Loricariidae|
Pareiorhina carrancas|[87]|Brazil|Loricariidae|
Pareiorhina carrancas|[88]|Brazil|Loricariidae|
Pareiorhina carrancas|[89]|Brazil|Loricariidae|
Pseudotocinclus tietensis|[90]|Brazil.Sao Paulo|Loricariidae|
Pseudotocinclus tietensis|[91]|Brazil.Sao Paulo|Loricariidae|
Pseudotocinclus tietensis|[92]|Brazil.Sao Paulo|Loricariidae|
Pseudotocinclus juquiae|[93]|Brazil.Sao Paulo|Loricariidae|
Pseudotocinclus tietensis|[94]|Loricariidae|
Pareiorhina sp.|[95]|Brazil.Sao Paulo|Loricariidae|
Pareiorhina sp.|[96]|Brazil.Sao Paulo|Loricariidae|
Pareiorhina sp.|[97]|Brazil.Sao Paulo|Loricariidae|
Pareiorhina sp.|[98]|Brazil.Sao Paulo|Loricariidae|
Pareiorhina sp.|[99]|Brazil.Sao Paulo|Loricariidae|

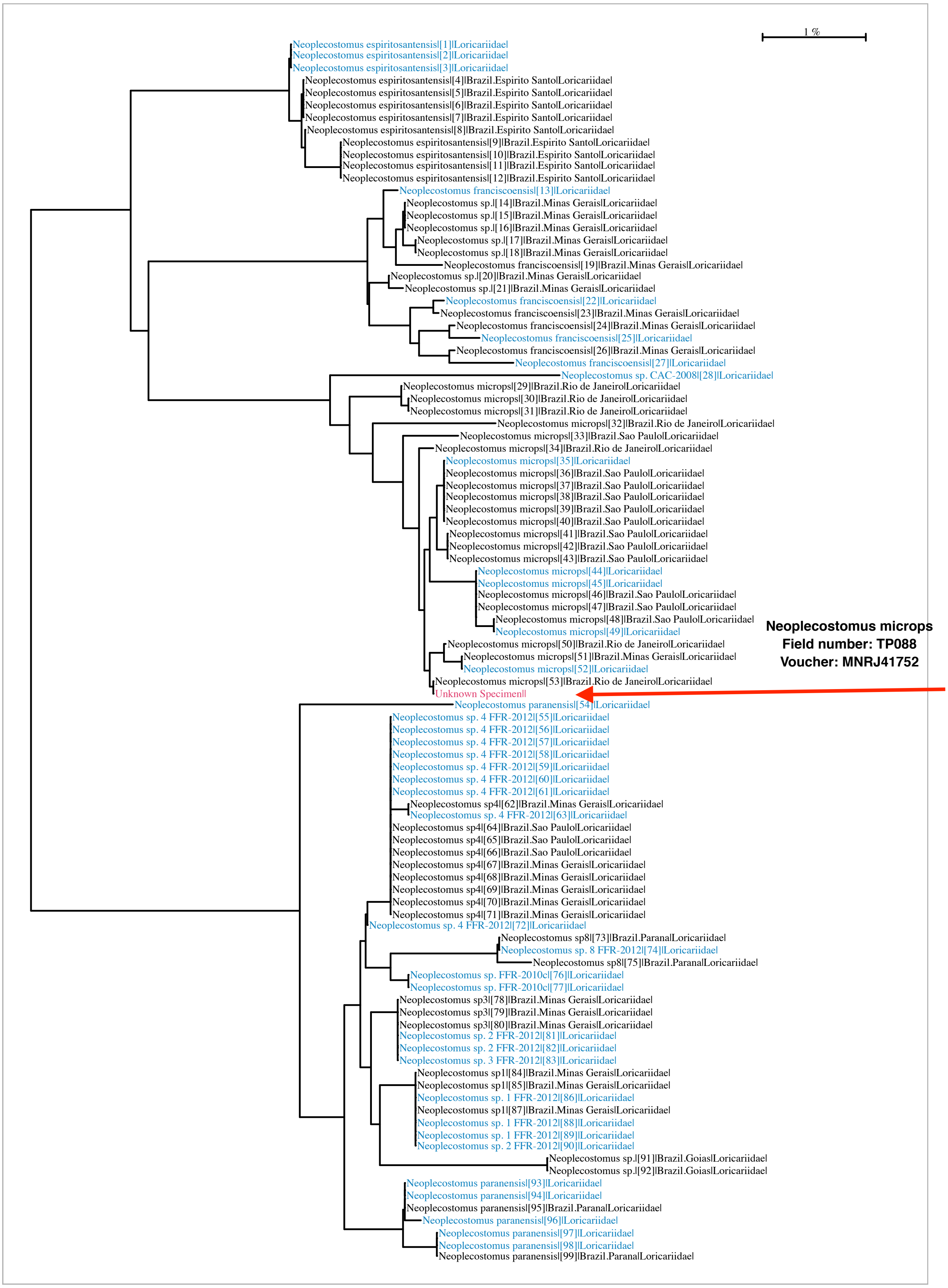
1 %
Neoplecostomus espiritosantensis|[1]|Loricariidae|
Neoplecostomus espiritosantensis|[2]|Loricariidae|
Neoplecostomus espiritosantensis|[3]|Loricariidae|
Neoplecostomus espiritosantensis|[4]|Brazil.Espirito Santo|Loricariidae|
Neoplecostomus espiritosantensis|[5]|Brazil.Espirito Santo|Loricariidae|
Neoplecostomus espiritosantensis|[6]|Brazil.Espirito Santo|Loricariidae|
Neoplecostomus espiritosantensis|[7]|Brazil.Espirito Santo|Loricariidae|
Neoplecostomus espiritosantensis|[8]|Brazil.Espirito Santo|Loricariidae|
Neoplecostomus espiritosantensis|[9]|Brazil.Espirito Santo|Loricariidae|
Neoplecostomus espiritosantensis|[10]|Brazil.Espirito Santo|Loricariidae|
Neoplecostomus espiritosantensis|[11]|Brazil.Espirito Santo|Loricariidae|
Neoplecostomus espiritosantensis|[12]|Brazil.Espirito Santo|Loricariidae|
Neoplecostomus franciscoensis|[13]|Loricariidae|
Neoplecostomus sp.|[14]|Brazil.Minas Gerais|Loricariidae|
Neoplecostomus sp.|[15]|Brazil.Minas Gerais|Loricariidae|
Neoplecostomus sp.|[16]|Brazil.Minas Gerais|Loricariidae|
Neoplecostomus sp.|[17]|Brazil.Minas Gerais|Loricariidae|
Neoplecostomus sp.|[18]|Brazil.Minas Gerais|Loricariidae|
Neoplecostomus franciscoensis|[19]|Brazil.Minas Gerais|Loricariidae|
Neoplecostomus sp.|[20]|Brazil.Minas Gerais|Loricariidae|
Neoplecostomus sp.|[21]|Brazil.Minas Gerais|Loricariidae|
Neoplecostomus franciscoensis|[22]|Loricariidae|
Neoplecostomus franciscoensis|[23]|Brazil.Minas Gerais|Loricariidae|
Neoplecostomus franciscoensis|[24]|Brazil.Minas Gerais|Loricariidae|
Neoplecostomus franciscoensis|[25]|Loricariidae|
Neoplecostomus franciscoensis|[26]|Brazil.Minas Gerais|Loricariidae|
Neoplecostomus franciscoensis|[27]|Loricariidae|
Neoplecostomus sp. CAC-2008|[28]|Loricariidae|
Neoplecostomus microps|[29]|Brazil.Rio de Janeiro|Loricariidae|
Neoplecostomus microps|[30]|Brazil.Rio de Janeiro|Loricariidae|
Neoplecostomus microps|[31]|Brazil.Rio de Janeiro|Loricariidae|
Neoplecostomus microps|[32]|Brazil.Rio de Janeiro|Loricariidae|
Neoplecostomus microps|[33]|Brazil.Sao Paulo|Loricariidae|
Neoplecostomus microps|[34]|Brazil.Rio de Janeiro|Loricariidae|
Neoplecostomus microps|[35]|Loricariidae|
Neoplecostomus microps|[36]|Brazil.Sao Paulo|Loricariidae|
Neoplecostomus microps|[37]|Brazil.Sao Paulo|Loricariidae|
Neoplecostomus microps|[38]|Brazil.Sao Paulo|Loricariidae|
Neoplecostomus microps|[39]|Brazil.Sao Paulo|Loricariidae|
Neoplecostomus microps|[40]|Brazil.Sao Paulo|Loricariidae|
Neoplecostomus microps|[41]|Brazil.Sao Paulo|Loricariidae|
Neoplecostomus microps|[42]|Brazil.Sao Paulo|Loricariidae|
Neoplecostomus microps|[43]|Brazil.Sao Paulo|Loricariidae|
Neoplecostomus microps|[44]|Loricariidae|
Neoplecostomus microps|[45]|Loricariidae|
Neoplecostomus microps|[46]|Brazil.Sao Paulo|Loricariidae|
Neoplecostomus microps|[47]|Brazil.Sao Paulo|Loricariidae|
Neoplecostomus microps|[48]|Brazil.Sao Paulo|Loricariidae|
Neoplecostomus microps|[49]|Loricariidae|
Neoplecostomus microps|[50]|Brazil.Rio de Janeiro|Loricariidae|
Neoplecostomus microps|[51]|Brazil.Minas Gerais|Loricariidae|
Neoplecostomus microps|[52]|Loricariidae|
Neoplecostomus microps|[53]|Brazil.Rio de Janeiro|Loricariidae|
Unknown Specimen||
Neoplecostomus paranensis|[54]|Loricariidae|
Neoplecostomus sp. 4 FFR-2012|[55]|Loricariidae|
Neoplecostomus sp. 4 FFR-2012|[56]|Loricariidae|
Neoplecostomus sp. 4 FFR-2012|[57]|Loricariidae|
Neoplecostomus sp. 4 FFR-2012|[58]|Loricariidae|
Neoplecostomus sp. 4 FFR-2012|[59]|Loricariidae|
Neoplecostomus sp. 4 FFR-2012|[60]|Loricariidae|
Neoplecostomus sp. 4 FFR-2012|[61]|Loricariidae|
Neoplecostomus sp4|[62]|Brazil.Minas Gerais|Loricariidae|
Neoplecostomus sp. 4 FFR-2012|[63]|Loricariidae|
Neoplecostomus sp4|[64]|Brazil.Sao Paulo|Loricariidae|
Neoplecostomus sp4|[65]|Brazil.Sao Paulo|Loricariidae|
Neoplecostomus sp4|[66]|Brazil.Sao Paulo|Loricariidae|
Neoplecostomus sp4|[67]|Brazil.Minas Gerais|Loricariidae|
Neoplecostomus sp4|[68]|Brazil.Minas Gerais|Loricariidae|
Neoplecostomus sp4|[69]|Brazil.Minas Gerais|Loricariidae|
Neoplecostomus sp4|[70]|Brazil.Minas Gerais|Loricariidae|
Neoplecostomus sp4|[71]|Brazil.Minas Gerais|Loricariidae|
Neoplecostomus sp. 4 FFR-2012|[72]|Loricariidae|
Neoplecostomus sp8|[73]|Brazil.Parana|Loricariidae|
Neoplecostomus sp. 8 FFR-2012|[74]|Loricariidae|
Neoplecostomus sp8|[75]|Brazil.Parana|Loricariidae|
Neoplecostomus sp. FFR-2010c|[76]|Loricariidae|
Neoplecostomus sp. FFR-2010c|[77]|Loricariidae|
Neoplecostomus sp3|[78]|Brazil.Minas Gerais|Loricariidae|
Neoplecostomus sp3|[79]|Brazil.Minas Gerais|Loricariidae|
Neoplecostomus sp3|[80]|Brazil.Minas Gerais|Loricariidae|
Neoplecostomus sp. 2 FFR-2012|[81]|Loricariidae|
Neoplecostomus sp. 2 FFR-2012|[82]|Loricariidae|
Neoplecostomus sp. 3 FFR-2012|[83]|Loricariidae|
Neoplecostomus sp1|[84]|Brazil.Minas Gerais|Loricariidae|
Neoplecostomus sp1|[85]|Brazil.Minas Gerais|Loricariidae|
Neoplecostomus sp. 1 FFR-2012|[86]|Loricariidae|
Neoplecostomus sp1|[87]|Brazil.Minas Gerais|Loricariidae|
Neoplecostomus sp. 1 FFR-2012|[88]|Loricariidae|
Neoplecostomus sp. 1 FFR-2012|[89]|Loricariidae|
Neoplecostomus sp. 2 FFR-2012|[90]|Loricariidae|
Neoplecostomus sp.|[91]|Brazil.Goias|Loricariidae|
Neoplecostomus sp.|[92]|Brazil.Goias|Loricariidae|
Neoplecostomus paranensis|[93]|Loricariidae|
Neoplecostomus paranensis|[94]|Loricariidae|
Neoplecostomus paranensis|[95]|Brazil.Parana|Loricariidae|
Neoplecostomus paranensis|[96]|Loricariidae|
Neoplecostomus paranensis|[97]|Loricariidae|
Neoplecostomus paranensis|[98]|Loricariidae|
Neoplecostomus paranensis|[99]|Brazil.Parana|Loricariidae|
Neoplecostomus microps
Field number: TP088
Voucher: MNRJ41752

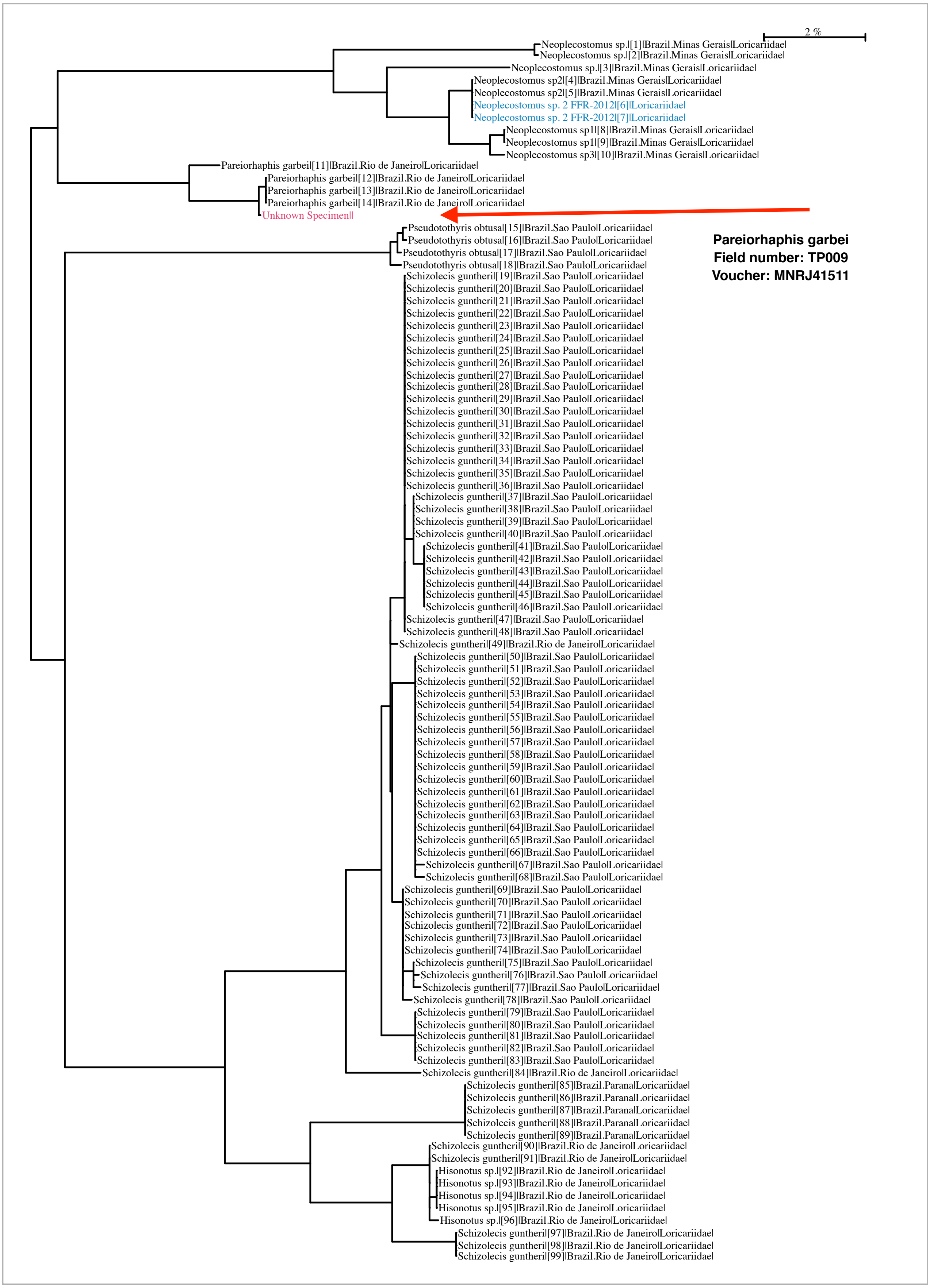
2 %
Neoplecostomus sp.|[1]|Brazil.Minas Gerais|Loricariidae|
Neoplecostomus sp.|[2]|Brazil.Minas Gerais|Loricariidae|
Neoplecostomus sp.|[3]|Brazil.Minas Gerais|Loricariidae|
Neoplecostomus sp2|[4]|Brazil.Minas Gerais|Loricariidae|
Neoplecostomus sp2|[5]|Brazil.Minas Gerais|Loricariidae|
Neoplecostomus sp. 2 FFR-2012|[6]|Loricariidae|
Neoplecostomus sp. 2 FFR-2012|[7]|Loricariidae|
Neoplecostomus sp1|[8]|Brazil.Minas Gerais|Loricariidae|
Neoplecostomus sp1|[9]|Brazil.Minas Gerais|Loricariidae|
Neoplecostomus sp3|[10]|Brazil.Minas Gerais|Loricariidae|
Pareiorhaphis garbei|[11]|Brazil.Rio de Janeiro|Loricariidae|
Pareiorhaphis garbei|[12]|Brazil.Rio de Janeiro|Loricariidae|
Pareiorhaphis garbei|[13]|Brazil.Rio de Janeiro|Loricariidae|
Pareiorhaphis garbei|[14]|Brazil.Rio de Janeiro|Loricariidae|
Unknown Specimen||
Pareiorhaphis garbei
Field number: TP009
Voucher: MNRJ41511
Pseudotothyris obtusa|[15]|Brazil.Sao Paulo|Loricariidae|
Pseudotothyris obtusa|[16]|Brazil.Sao Paulo|Loricariidae|
Pseudotothyris obtusa|[17]|Brazil.Sao Paulo|Loricariidae|
Pseudotothyris obtusa|[18]|Brazil.Sao Paulo|Loricariidae|
Schizolecis guntheri|[19]|Brazil.Sao Paulo|Loricariidae|
Schizolecis guntheri|[20]|Brazil.Sao Paulo|Loricariidae|
Schizolecis guntheri|[21]|Brazil.Sao Paulo|Loricariidae|
Schizolecis guntheri|[22]|Brazil.Sao Paulo|Loricariidae|
Schizolecis guntheri|[23]|Brazil.Sao Paulo|Loricariidae|
Schizolecis guntheri|[24]|Brazil.Sao Paulo|Loricariidae|
Schizolecis guntheri|[25]|Brazil.Sao Paulo|Loricariidae|
Schizolecis guntheri|[26]|Brazil.Sao Paulo|Loricariidae|
Schizolecis guntheri|[27]|Brazil.Sao Paulo|Loricariidae|
Schizolecis guntheri|[28]|Brazil.Sao Paulo|Loricariidae|
Schizolecis guntheri|[29]|Brazil.Sao Paulo|Loricariidae|
Schizolecis guntheri|[30]|Brazil.Sao Paulo|Loricariidae|
Schizolecis guntheri|[31]|Brazil.Sao Paulo|Loricariidae|
Schizolecis guntheri|[32]|Brazil.Sao Paulo|Loricariidae|
Schizolecis guntheri|[33]|Brazil.Sao Paulo|Loricariidae|
Schizolecis guntheri|[34]|Brazil.Sao Paulo|Loricariidae|
Schizolecis guntheri|[35]|Brazil.Sao Paulo|Loricariidae|
Schizolecis guntheri|[36]|Brazil.Sao Paulo|Loricariidae|
Schizolecis guntheri|[37]|Brazil.Sao Paulo|Loricariidae|
Schizolecis guntheri|[38]|Brazil.Sao Paulo|Loricariidae|
Schizolecis guntheri|[39]|Brazil.Sao Paulo|Loricariidae|
Schizolecis guntheri|[40]|Brazil.Sao Paulo|Loricariidae|
Schizolecis guntheri|[41]|Brazil.Sao Paulo|Loricariidae|
Schizolecis guntheri|[42]|Brazil.Sao Paulo|Loricariidae|
Schizolecis guntheri|[43]|Brazil.Sao Paulo|Loricariidae|
Schizolecis guntheri|[44]|Brazil.Sao Paulo|Loricariidae|
Schizolecis guntheri|[45]|Brazil.Sao Paulo|Loricariidae|
Schizolecis guntheri|[46]|Brazil.Sao Paulo|Loricariidae|
Schizolecis guntheri|[47]|Brazil.Sao Paulo|Loricariidae|
Schizolecis guntheri|[48]|Brazil.Sao Paulo|Loricariidae|
Schizolecis guntheri|[49]|Brazil.Rio de Janeiro|Loricariidae|
Schizolecis guntheri|[50]|Brazil.Sao Paulo|Loricariidae|
Schizolecis guntheri|[51]|Brazil.Sao Paulo|Loricariidae|
Schizolecis guntheri|[52]|Brazil.Sao Paulo|Loricariidae|
Schizolecis guntheri|[53]|Brazil.Sao Paulo|Loricariidae|
Schizolecis guntheri|[54]|Brazil.Sao Paulo|Loricariidae|
Schizolecis guntheri|[55]|Brazil.Sao Paulo|Loricariidae|
Schizolecis guntheri|[56]|Brazil.Sao Paulo|Loricariidae|
Schizolecis guntheri|[57]|Brazil.Sao Paulo|Loricariidae|
Schizolecis guntheri|[58]|Brazil.Sao Paulo|Loricariidae|
Schizolecis guntheri|[59]|Brazil.Sao Paulo|Loricariidae|
Schizolecis guntheri|[60]|Brazil.Sao Paulo|Loricariidae|
Schizolecis guntheri|[61]|Brazil.Sao Paulo|Loricariidae|
Schizolecis guntheri|[62]|Brazil.Sao Paulo|Loricariidae|
Schizolecis guntheri|[63]|Brazil.Sao Paulo|Loricariidae|
Schizolecis guntheri|[64]|Brazil.Sao Paulo|Loricariidae|
Schizolecis guntheri|[65]|Brazil.Sao Paulo|Loricariidae|
Schizolecis guntheri|[66]|Brazil.Sao Paulo|Loricariidae|
Schizolecis guntheri|[67]|Brazil.Sao Paulo|Loricariidae|
Schizolecis guntheri|[68]|Brazil.Sao Paulo|Loricariidae|
Schizolecis guntheri|[69]|Brazil.Sao Paulo|Loricariidae|
Schizolecis guntheri|[70]|Brazil.Sao Paulo|Loricariidae|
Schizolecis guntheri|[71]|Brazil.Sao Paulo|Loricariidae|
Schizolecis guntheri|[72]|Brazil.Sao Paulo|Loricariidae|
Schizolecis guntheri|[73]|Brazil.Sao Paulo|Loricariidae|
Schizolecis guntheri|[74]|Brazil.Sao Paulo|Loricariidae|
Schizolecis guntheri|[75]|Brazil.Sao Paulo|Loricariidae|
Schizolecis guntheri|[76]|Brazil.Sao Paulo|Loricariidae|
Schizolecis guntheri|[77]|Brazil.Sao Paulo|Loricariidae|
Schizolecis guntheri|[78]|Brazil.Sao Paulo|Loricariidae|
Schizolecis guntheri|[79]|Brazil.Sao Paulo|Loricariidae|
Schizolecis guntheri|[80]|Brazil.Sao Paulo|Loricariidae|
Schizolecis guntheri|[81]|Brazil.Sao Paulo|Loricariidae|
Schizolecis guntheri|[82]|Brazil.Sao Paulo|Loricariidae|
Schizolecis guntheri|[83]|Brazil.Sao Paulo|Loricariidae|
Schizolecis guntheri|[84]|Brazil.Rio de Janeiro|Loricariidae|
Schizolecis guntheri|[85]|Brazil.Parana|Loricariidae|
Schizolecis guntheri|[86]|Brazil.Parana|Loricariidae|
Schizolecis guntheri|[87]|Brazil.Parana|Loricariidae|
Schizolecis guntheri|[88]|Brazil.Parana|Loricariidae|
Schizolecis guntheri|[89]|Brazil.Parana|Loricariidae|
Schizolecis guntheri|[90]|Brazil.Rio de Janeiro|Loricariidae|
Schizolecis guntheri|[91]|Brazil.Rio de Janeiro|Loricariidae|
Hisonotus sp.|[92]|Brazil.Rio de Janeiro|Loricariidae|
Hisonotus sp.|[93]|Brazil.Rio de Janeiro|Loricariidae|
Hisonotus sp.|[94]|Brazil.Rio de Janeiro|Loricariidae|
Hisonotus sp.|[95]|Brazil.Rio de Janeiro|Loricariidae|
Hisonotus sp.|[96]|Brazil.Rio de Janeiro|Loricariidae|
Schizolecis guntheri|[97]|Brazil.Rio de Janeiro|Loricariidae|
Schizolecis guntheri|[98]|Brazil.Rio de Janeiro|Loricariidae|
Schizolecis guntheri|[99]|Brazil.Rio de Janeiro|Loricariidae|

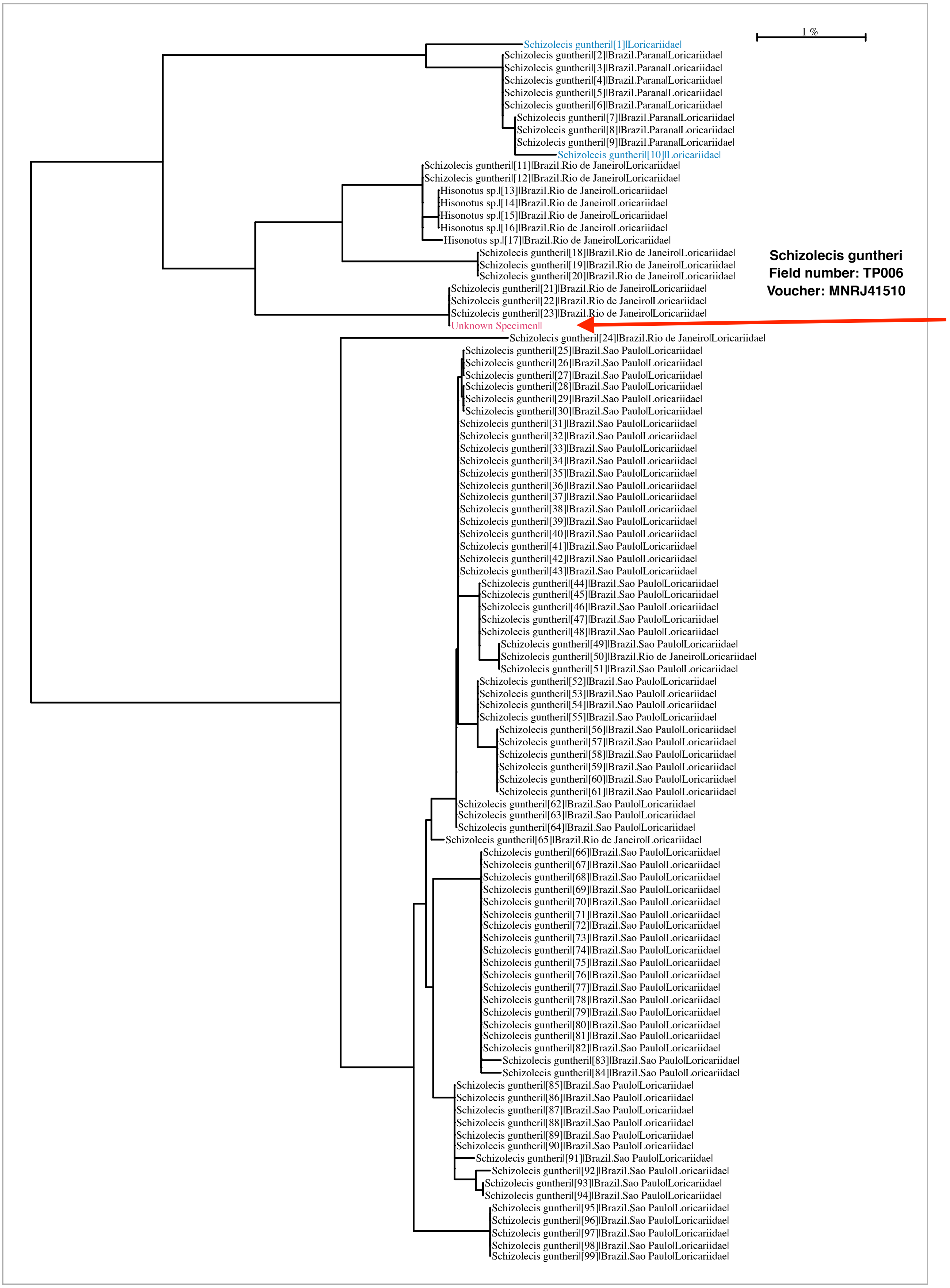
1 %
Schizolecis guntheri|[1]|Loricariidae|
Schizolecis guntheri|[2]|Brazil.Parana|Loricariidae|
Schizolecis guntheri|[3]|Brazil.Parana|Loricariidae|
Schizolecis guntheri|[4]|Brazil.Parana|Loricariidae|
Schizolecis guntheri|[5]|Brazil.Parana|Loricariidae|
Schizolecis guntheri|[6]|Brazil.Parana|Loricariidae|
Schizolecis guntheri|[7]|Brazil.Parana|Loricariidae|
Schizolecis guntheri|[8]|Brazil.Parana|Loricariidae|
Schizolecis guntheri|[9]|Brazil.Parana|Loricariidae|
Schizolecis guntheri|[10]|Loricariidae|
Schizolecis guntheri|[11]|Brazil.Rio de Janeiro|Loricariidae|
Schizolecis guntheri|[12]|Brazil.Rio de Janeiro|Loricariidae|
Hisonotus sp.|[13]|Brazil.Rio de Janeiro|Loricariidae|
Hisonotus sp.|[14]|Brazil.Rio de Janeiro|Loricariidae|
Hisonotus sp.|[15]|Brazil.Rio de Janeiro|Loricariidae|
Hisonotus sp.|[16]|Brazil.Rio de Janeiro|Loricariidae|
Hisonotus sp.|[17]|Brazil.Rio de Janeiro|Loricariidae|
Schizolecis guntheri|[18]|Brazil.Rio de Janeiro|Loricariidae|
Schizolecis guntheri|[19]|Brazil.Rio de Janeiro|Loricariidae|
Schizolecis guntheri|[20]|Brazil.Rio de Janeiro|Loricariidae|
Schizolecis guntheri|[21]|Brazil.Rio de Janeiro|Loricariidae|
Schizolecis guntheri|[22]|Brazil.Rio de Janeiro|Loricariidae|
Schizolecis guntheri|[23]|Brazil.Rio de Janeiro|Loricariidae|
Unknown Specimen||
Schizolecis guntheri
Field number: TP006
Voucher: MNRJ41510
Schizolecis guntheri|[24]|Brazil.Rio de Janeiro|Loricariidae|
Schizolecis guntheri|[25]|Brazil.Sao Paulo|Loricariidae|
Schizolecis guntheri|[26]|Brazil.Sao Paulo|Loricariidae|
Schizolecis guntheri|[27]|Brazil.Sao Paulo|Loricariidae|
Schizolecis guntheri|[28]|Brazil.Sao Paulo|Loricariidae|
Schizolecis guntheri|[29]|Brazil.Sao Paulo|Loricariidae|
Schizolecis guntheri|[30]|Brazil.Sao Paulo|Loricariidae|
Schizolecis guntheri|[31]|Brazil.Sao Paulo|Loricariidae|
Schizolecis guntheri|[32]|Brazil.Sao Paulo|Loricariidae|
Schizolecis guntheri|[33]|Brazil.Sao Paulo|Loricariidae|
Schizolecis guntheri|[34]|Brazil.Sao Paulo|Loricariidae|
Schizolecis guntheri|[35]|Brazil.Sao Paulo|Loricariidae|
Schizolecis guntheri|[36]|Brazil.Sao Paulo|Loricariidae|
Schizolecis guntheri|[37]|Brazil.Sao Paulo|Loricariidae|
Schizolecis guntheri|[38]|Brazil.Sao Paulo|Loricariidae|
Schizolecis guntheri|[39]|Brazil.Sao Paulo|Loricariidae|
Schizolecis guntheri|[40]|Brazil.Sao Paulo|Loricariidae|
Schizolecis guntheri|[41]|Brazil.Sao Paulo|Loricariidae|
Schizolecis guntheri|[42]|Brazil.Sao Paulo|Loricariidae|
Schizolecis guntheri|[43]|Brazil.Sao Paulo|Loricariidae|
Schizolecis guntheri|[44]|Brazil.Sao Paulo|Loricariidae|
Schizolecis guntheri|[45]|Brazil.Sao Paulo|Loricariidae|
Schizolecis guntheri|[46]|Brazil.Sao Paulo|Loricariidae|
Schizolecis guntheri|[47]|Brazil.Sao Paulo|Loricariidae|
Schizolecis guntheri|[48]|Brazil.Sao Paulo|Loricariidae|
Schizolecis guntheri|[49]|Brazil.Sao Paulo|Loricariidae|
Schizolecis guntheri|[50]|Brazil.Rio de Janeiro|Loricariidae|
Schizolecis guntheri|[51]|Brazil.Sao Paulo|Loricariidae|
Schizolecis guntheri|[52]|Brazil.Sao Paulo|Loricariidae|
Schizolecis guntheri|[53]|Brazil.Sao Paulo|Loricariidae|
Schizolecis guntheri|[54]|Brazil.Sao Paulo|Loricariidae|
Schizolecis guntheri|[55]|Brazil.Sao Paulo|Loricariidae|
Schizolecis guntheri|[56]|Brazil.Sao Paulo|Loricariidae|
Schizolecis guntheri|[57]|Brazil.Sao Paulo|Loricariidae|
Schizolecis guntheri|[58]|Brazil.Sao Paulo|Loricariidae|
Schizolecis guntheri|[59]|Brazil.Sao Paulo|Loricariidae|
Schizolecis guntheri|[60]|Brazil.Sao Paulo|Loricariidae|
Schizolecis guntheri|[61]|Brazil.Sao Paulo|Loricariidae|
Schizolecis guntheri|[62]|Brazil.Sao Paulo|Loricariidae|
Schizolecis guntheri|[63]|Brazil.Sao Paulo|Loricariidae|
Schizolecis guntheri|[64]|Brazil.Sao Paulo|Loricariidae|
Schizolecis guntheri|[65]|Brazil.Rio de Janeiro|Loricariidae|
Schizolecis guntheri|[66]|Brazil.Sao Paulo|Loricariidae|
Schizolecis guntheri|[67]|Brazil.Sao Paulo|Loricariidae|
Schizolecis guntheri|[68]|Brazil.Sao Paulo|Loricariidae|
Schizolecis guntheri|[69]|Brazil.Sao Paulo|Loricariidae|
Schizolecis guntheri|[70]|Brazil.Sao Paulo|Loricariidae|
Schizolecis guntheri|[71]|Brazil.Sao Paulo|Loricariidae|
Schizolecis guntheri|[72]|Brazil.Sao Paulo|Loricariidae|
Schizolecis guntheri|[73]|Brazil.Sao Paulo|Loricariidae|
Schizolecis guntheri|[74]|Brazil.Sao Paulo|Loricariidae|
Schizolecis guntheri|[75]|Brazil.Sao Paulo|Loricariidae|
Schizolecis guntheri|[76]|Brazil.Sao Paulo|Loricariidae|
Schizolecis guntheri|[77]|Brazil.Sao Paulo|Loricariidae|
Schizolecis guntheri|[78]|Brazil.Sao Paulo|Loricariidae|
Schizolecis guntheri|[79]|Brazil.Sao Paulo|Loricariidae|
Schizolecis guntheri|[80]|Brazil.Sao Paulo|Loricariidae|
Schizolecis guntheri|[81]|Brazil.Sao Paulo|Loricariidae|
Schizolecis guntheri|[82]|Brazil.Sao Paulo|Loricariidae|
Schizolecis guntheri|[83]|Brazil.Sao Paulo|Loricariidae|
Schizolecis guntheri|[84]|Brazil.Sao Paulo|Loricariidae|
Schizolecis guntheri|[85]|Brazil.Sao Paulo|Loricariidae|
Schizolecis guntheri|[86]|Brazil.Sao Paulo|Loricariidae|
Schizolecis guntheri|[87]|Brazil.Sao Paulo|Loricariidae|
Schizolecis guntheri|[88]|Brazil.Sao Paulo|Loricariidae|
Schizolecis guntheri|[89]|Brazil.Sao Paulo|Loricariidae|
Schizolecis guntheri|[90]|Brazil.Sao Paulo|Loricariidae|
Schizolecis guntheri|[91]|Brazil.Sao Paulo|Loricariidae|
Schizolecis guntheri|[92]|Brazil.Sao Paulo|Loricariidae|
Schizolecis guntheri|[93]|Brazil.Sao Paulo|Loricariidae|
Schizolecis guntheri|[94]|Brazil.Sao Paulo|Loricariidae|
Schizolecis guntheri|[95]|Brazil.Sao Paulo|Loricariidae|
Schizolecis guntheri|[96]|Brazil.Sao Paulo|Loricariidae|
Schizolecis guntheri|[97]|Brazil.Sao Paulo|Loricariidae|
Schizolecis guntheri|[98]|Brazil.Sao Paulo|Loricariidae|
Schizolecis guntheri|[99]|Brazil.Sao Paulo|Loricariidae|

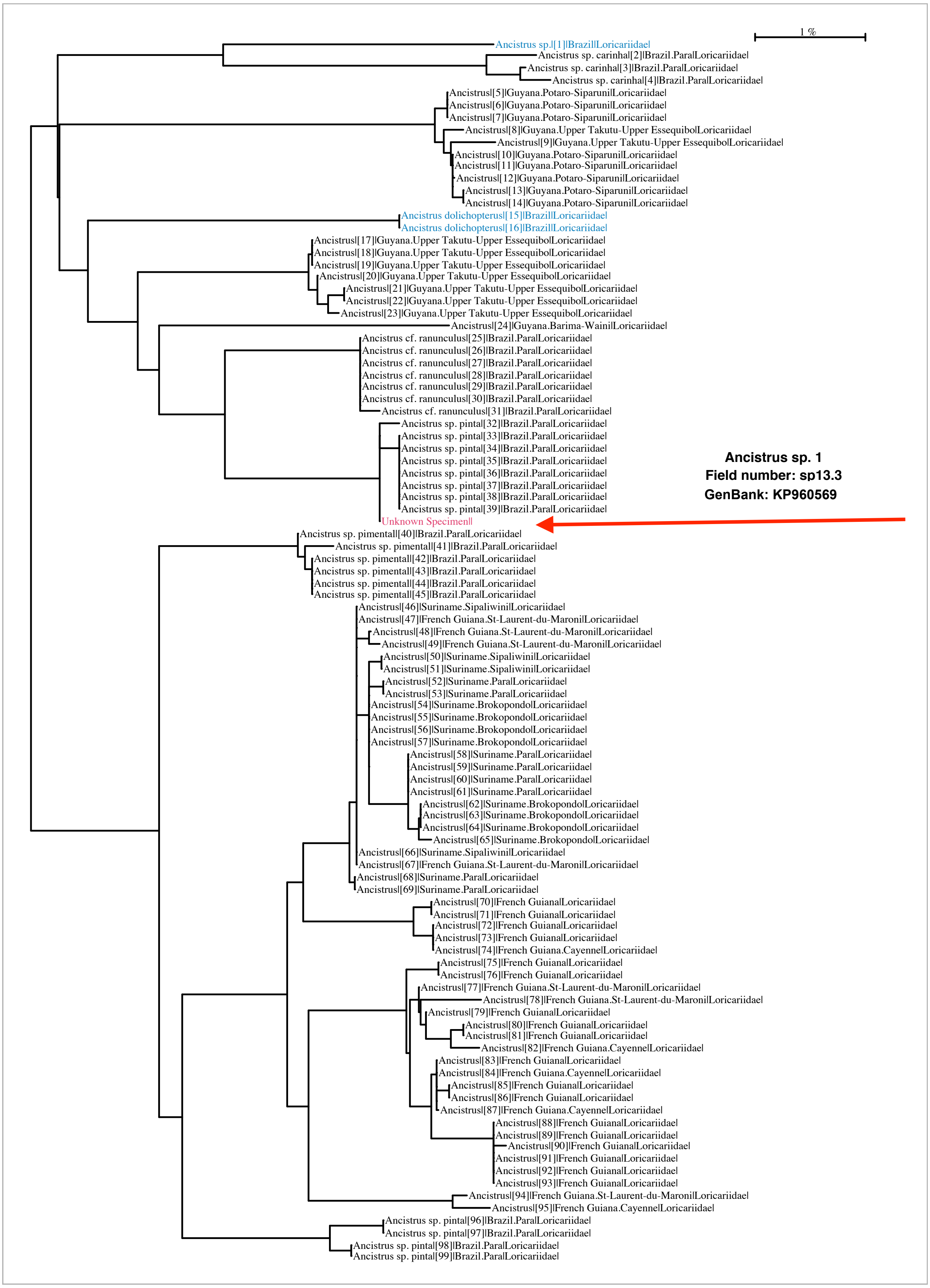
1 %
Ancistrus sp.|[1]|Brazil|Loricariidae|
Ancistrus sp. carinha|[2]|Brazil.Para|Loricariidae|
Ancistrus sp. carinha|[3]|Brazil.Para|Loricariidae|
Ancistrus sp. carinha|[4]|Brazil.Para|Loricariidae|
Ancistrus|[5]|Guyana.Potaro-Siparuni|Loricariidae|
Ancistrus|[6]|Guyana.Potaro-Siparuni|Loricariidae|
Ancistrus|[7]|Guyana.Potaro-Siparuni|Loricariidae|
Ancistrus|[8]|Guyana.Upper Takutu-Upper Essequibo|Loricariidae|
Ancistrus|[9]|Guyana.Upper Takutu-Upper Essequibo|Loricariidae|
Ancistrus|[10]|Guyana.Potaro-Siparuni|Loricariidae|
Ancistrus|[11]|Guyana.Potaro-Siparuni|Loricariidae|
Ancistrus|[12]|Guyana.Potaro-Siparuni|Loricariidae|
Ancistrus|[13]|Guyana.Potaro-Siparuni|Loricariidae|
Ancistrus|[14]|Guyana.Potaro-Siparuni|Loricariidae|
Ancistrus dolichopterus|[15]|Brazil|Loricariidae|
Ancistrus dolichopterus|[16]|Brazil|Loricariidae|
Ancistrus|[17]|Guyana.Upper Takutu-Upper Essequibo|Loricariidae|
Ancistrus|[18]|Guyana.Upper Takutu-Upper Essequibo|Loricariidae|
Ancistrus|[19]|Guyana.Upper Takutu-Upper Essequibo|Loricariidae|
Ancistrus|[20]|Guyana.Upper Takutu-Upper Essequibo|Loricariidae|
Ancistrus|[21]|Guyana.Upper Takutu-Upper Essequibo|Loricariidae|
Ancistrus|[22]|Guyana.Upper Takutu-Upper Essequibo|Loricariidae|
Ancistrus|[23]|Guyana.Upper Takutu-Upper Essequibo|Loricariidae|
Ancistrus|[24]|Guyana.Barima-Waini|Loricariidae|
Ancistrus cf. ranunculus|[25]|Brazil.Para|Loricariidae|
Ancistrus cf. ranunculus|[26]|Brazil.Para|Loricariidae|
Ancistrus cf. ranunculus|[27]|Brazil.Para|Loricariidae|
Ancistrus cf. ranunculus|[28]|Brazil.Para|Loricariidae|
Ancistrus cf. ranunculus|[29]|Brazil.Para|Loricariidae|
Ancistrus cf. ranunculus|[30]|Brazil.Para|Loricariidae|
Ancistrus cf. ranunculus|[31]|Brazil.Para|Loricariidae|
Ancistrus sp. pinta|[32]|Brazil.Para|Loricariidae|
Ancistrus sp. pinta|[33]|Brazil.Para|Loricariidae|
Ancistrus sp. pinta|[34]|Brazil.Para|Loricariidae|
Ancistrus sp. pinta|[35]|Brazil.Para|Loricariidae|
Ancistrus sp. pinta|[36]|Brazil.Para|Loricariidae|
Ancistrus sp. pinta|[37]|Brazil.Para|Loricariidae|
Ancistrus sp. pinta|[38]|Brazil.Para|Loricariidae|
Ancistrus sp. pinta|[39]|Brazil.Para|Loricariidae|
Unknown Specimen||
Ancistrus sp. 1
Field number: sp13.3
GenBank: KP960569
Ancistrus sp. pimenta|[40]|Brazil.Para|Loricariidae|
Ancistrus sp. pimenta|[41]|Brazil.Para|Loricariidae|
Ancistrus sp. pimenta|[42]|Brazil.Para|Loricariidae|
Ancistrus sp. pimenta|[43]|Brazil.Para|Loricariidae|
Ancistrus sp. pimenta|[44]|Brazil.Para|Loricariidae|
Ancistrus sp. pimenta|[45]|Brazil.Para|Loricariidae|
Ancistrus|[46]|Suriname.Sipaliwini|Loricariidae|
Ancistrus|[47]|French Guiana.St-Laurent-du-Maroni|Loricariidae|
Ancistrus|[48]|French Guiana.St-Laurent-du-Maroni|Loricariidae|
Ancistrus|[49]|French Guiana.St-Laurent-du-Maroni|Loricariidae|
Ancistrus|[50]|Suriname.Sipaliwini|Loricariidae|
Ancistrus|[51]|Suriname.Sipaliwini|Loricariidae|
Ancistrus|[52]|Suriname.Para|Loricariidae|
Ancistrus|[53]|Suriname.Para|Loricariidae|
Ancistrus|[54]|Suriname.Brokopondo|Loricariidae|
Ancistrus|[55]|Suriname.Brokopondo|Loricariidae|
Ancistrus|[56]|Suriname.Brokopondo|Loricariidae|
Ancistrus|[57]|Suriname.Brokopondo|Loricariidae|
Ancistrus|[58]|Suriname.Para|Loricariidae|
Ancistrus|[59]|Suriname.Para|Loricariidae|
Ancistrus|[60]|Suriname.Para|Loricariidae|
Ancistrus|[61]|Suriname.Para|Loricariidae|
Ancistrus|[62]|Suriname.Brokopondo|Loricariidae|
Ancistrus|[63]|Suriname.Brokopondo|Loricariidae|
Ancistrus|[64]|Suriname.Brokopondo|Loricariidae|
Ancistrus|[65]|Suriname.Brokopondo|Loricariidae|
Ancistrus|[66]|Suriname.Sipaliwini|Loricariidae|
Ancistrus|[67]|French Guiana.St-Laurent-du-Maroni|Loricariidae|
Ancistrus|[68]|Suriname.Para|Loricariidae|
Ancistrus|[69]|Suriname.Para|Loricariidae|
Ancistrus|[70]|French Guiana|Loricariidae|
Ancistrus|[71]|French Guiana|Loricariidae|
Ancistrus|[72]|French Guiana|Loricariidae|
Ancistrus|[73]|French Guiana|Loricariidae|
Ancistrus|[74]|French Guiana.Cayenne|Loricariidae|
Ancistrus|[75]|French Guiana|Loricariidae|
Ancistrus|[76]|French Guiana|Loricariidae|
Ancistrus|[77]|French Guiana.St-Laurent-du-Maroni|Loricariidae|
Ancistrus|[78]|French Guiana.St-Laurent-du-Maroni|Loricariidae|
Ancistrus|[79]|French Guiana|Loricariidae|
Ancistrus|[80]|French Guiana|Loricariidae|
Ancistrus|[81]|French Guiana|Loricariidae|
Ancistrus|[82]|French Guiana.Cayenne|Loricariidae|
Ancistrus|[83]|French Guiana|Loricariidae|
Ancistrus|[84]|French Guiana.Cayenne|Loricariidae|
Ancistrus|[85]|French Guiana|Loricariidae|
Ancistrus|[86]|French Guiana|Loricariidae|
Ancistrus|[87]|French Guiana.Cayenne|Loricariidae|
Ancistrus|[88]|French Guiana|Loricariidae|
Ancistrus|[89]|French Guiana|Loricariidae|
Ancistrus|[90]|French Guiana|Loricariidae|
Ancistrus|[91]|French Guiana|Loricariidae|
Ancistrus|[92]|French Guiana|Loricariidae|
Ancistrus|[93]|French Guiana|Loricariidae|
Ancistrus|[94]|French Guiana.St-Laurent-du-Maroni|Loricariidae|
Ancistrus|[95]|French Guiana.Cayenne|Loricariidae|
Ancistrus sp. pinta|[96]|Brazil.Para|Loricariidae|
Ancistrus sp. pinta|[97]|Brazil.Para|Loricariidae|
Ancistrus sp. pinta|[98]|Brazil.Para|Loricariidae|
Ancistrus sp. pinta|[99]|Brazil.Para|Loricariidae|

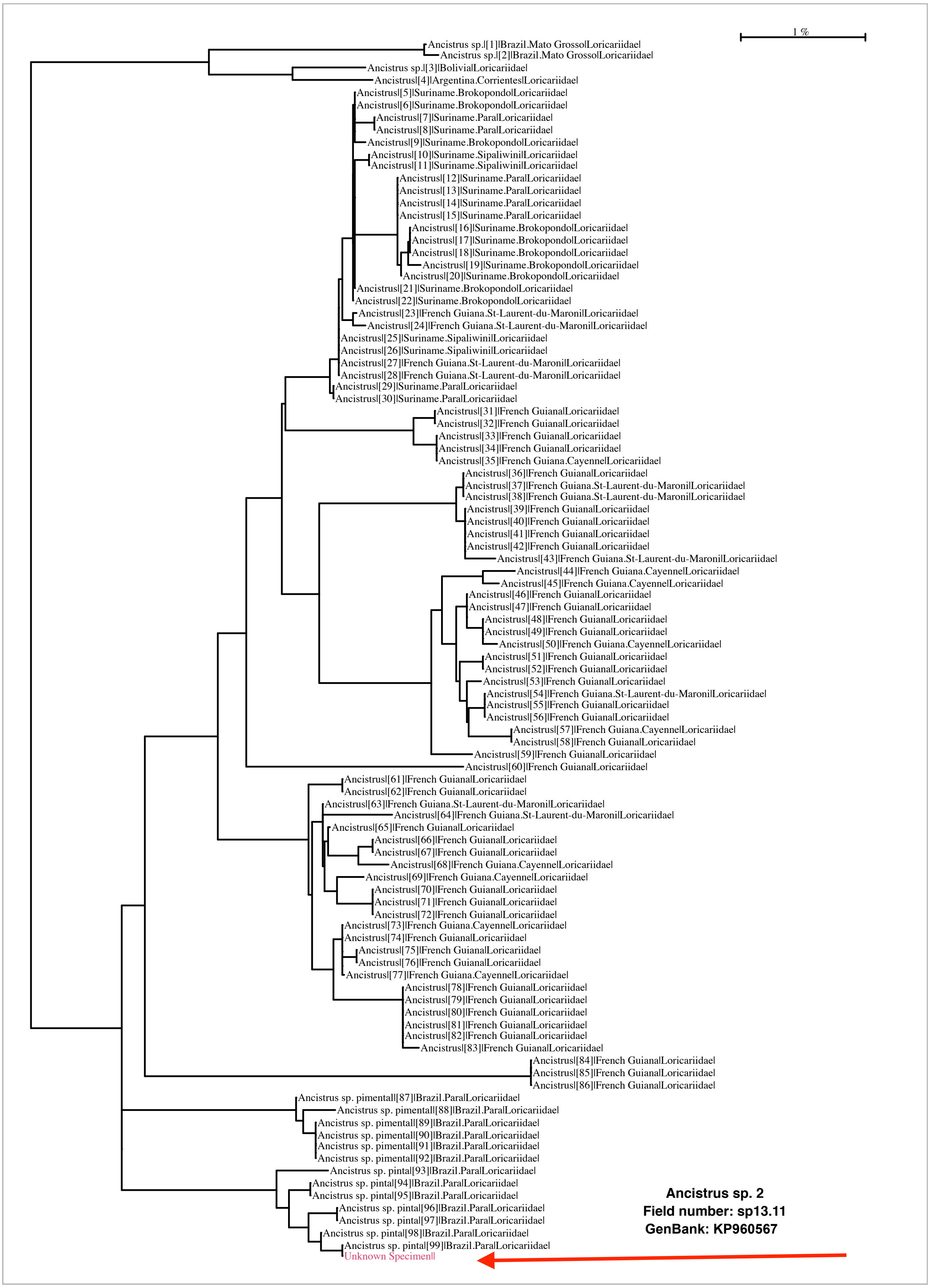
1 %
Ancistrus sp.|[1]|Brazil.Mato Grosso|Loricariidae|
Ancistrus sp.|[2]|Brazil.Mato Grosso|Loricariidae|
Ancistrus sp.|[3]|Bolivia|Loricariidae|
Ancistrus|[4]|Argentina.Corrientes|Loricariidae|
Ancistrus|[5]|Suriname.Brokopondo|Loricariidae|
Ancistrus|[6]|Suriname.Brokopondo|Loricariidae|
Ancistrus|[7]|Suriname.Para|Loricariidae|
Ancistrus|[8]|Suriname.Para|Loricariidae|
Ancistrus|[9]|Suriname.Brokopondo|Loricariidae|
Ancistrus|[10]|Suriname.Sipaliwini|Loricariidae|
Ancistrus|[11]|Suriname.Sipaliwini|Loricariidae|
Ancistrus|[12]|Suriname.Para|Loricariidae|
Ancistrus|[13]|Suriname.Para|Loricariidae|
Ancistrus|[14]|Suriname.Para|Loricariidae|
Ancistrus|[15]|Suriname.Para|Loricariidae|
Ancistrus|[16]|Suriname.Brokopondo|Loricariidae|
Ancistrus|[17]|Suriname.Brokopondo|Loricariidae|
Ancistrus|[18]|Suriname.Brokopondo|Loricariidae|
Ancistrus|[19]|Suriname.Brokopondo|Loricariidae|
Ancistrus|[20]|Suriname.Brokopondo|Loricariidae|
Ancistrus|[21]|Suriname.Brokopondo|Loricariidae|
Ancistrus|[22]|Suriname.Brokopondo|Loricariidae|
Ancistrus|[23]|French Guiana.St-Laurent-du-Maroni|Loricariidae|
Ancistrus|[24]|French Guiana.St-Laurent-du-Maroni|Loricariidae|
Ancistrus|[25]|Suriname.Sipaliwini|Loricariidae|
Ancistrus|[26]|Suriname.Sipaliwini|Loricariidae|
Ancistrus|[27]|French Guiana.St-Laurent-du-Maroni|Loricariidae|
Ancistrus|[28]|French Guiana.St-Laurent-du-Maroni|Loricariidae|
Ancistrus|[29]|Suriname.Para|Loricariidae|
Ancistrus|[30]|Suriname.Para|Loricariidae|
Ancistrus|[31]|French Guiana|Loricariidae|
Ancistrus|[32]|French Guiana|Loricariidae|
Ancistrus|[33]|French Guiana|Loricariidae|
Ancistrus|[34]|French Guiana|Loricariidae|
Ancistrus|[35]|French Guiana.Cayenne|Loricariidae|
Ancistrus|[36]|French Guiana|Loricariidae|
Ancistrus|[37]|French Guiana.St-Laurent-du-Maroni|Loricariidae|
Ancistrus|[38]|French Guiana.St-Laurent-du-Maroni|Loricariidae|
Ancistrus|[39]|French Guiana|Loricariidae|
Ancistrus|[40]|French Guiana|Loricariidae|
Ancistrus|[41]|French Guiana|Loricariidae|
Ancistrus|[42]|French Guiana|Loricariidae|
Ancistrus|[43]|French Guiana.St-Laurent-du-Maroni|Loricariidae|
Ancistrus|[44]|French Guiana.Cayenne|Loricariidae|
Ancistrus|[45]|French Guiana.Cayenne|Loricariidae|
Ancistrus|[46]|French Guiana|Loricariidae|
Ancistrus|[47]|French Guiana|Loricariidae|
Ancistrus|[48]|French Guiana|Loricariidae|
Ancistrus|[49]|French Guiana|Loricariidae|
Ancistrus|[50]|French Guiana.Cayenne|Loricariidae|
Ancistrus|[51]|French Guiana|Loricariidae|
Ancistrus|[52]|French Guiana|Loricariidae|
Ancistrus|[53]|French Guiana|Loricariidae|
Ancistrus|[54]|French Guiana.St-Laurent-du-Maroni|Loricariidae|
Ancistrus|[55]|French Guiana|Loricariidae|
Ancistrus|[56]|French Guiana|Loricariidae|
Ancistrus|[57]|French Guiana.Cayenne|Loricariidae|
Ancistrus|[58]|French Guiana|Loricariidae|
Ancistrus|[59]|French Guiana|Loricariidae|
Ancistrus|[60]|French Guiana|Loricariidae|
Ancistrus|[61]|French Guiana|Loricariidae|
Ancistrus|[62]|French Guiana|Loricariidae|
Ancistrus|[63]|French Guiana.St-Laurent-du-Maroni|Loricariidae|
Ancistrus|[64]|French Guiana.St-Laurent-du-Maroni|Loricariidae|
Ancistrus|[65]|French Guiana|Loricariidae|
Ancistrus|[66]|French Guiana|Loricariidae|
Ancistrus|[67]|French Guiana|Loricariidae|
Ancistrus|[68]|French Guiana.Cayenne|Loricariidae|
Ancistrus|[69]|French Guiana.Cayenne|Loricariidae|
Ancistrus|[70]|French Guiana|Loricariidae|
Ancistrus|[71]|French Guiana|Loricariidae|
Ancistrus|[72]|French Guiana|Loricariidae|
Ancistrus|[73]|French Guiana.Cayenne|Loricariidae|
Ancistrus|[74]|French Guiana|Loricariidae|
Ancistrus|[75]|French Guiana|Loricariidae|
Ancistrus|[76]|French Guiana|Loricariidae|
Ancistrus|[77]|French Guiana.Cayenne|Loricariidae|
Ancistrus|[78]|French Guiana|Loricariidae|
Ancistrus|[79]|French Guiana|Loricariidae|
Ancistrus|[80]|French Guiana|Loricariidae|
Ancistrus|[81]|French Guiana|Loricariidae|
Ancistrus|[82]|French Guiana|Loricariidae|
Ancistrus|[83]|French Guiana|Loricariidae|
Ancistrus|[84]|French Guiana|Loricariidae|
Ancistrus|[85]|French Guiana|Loricariidae|
Ancistrus|[86]|French Guiana|Loricariidae|
Ancistrus sp. pimentall[87]|Brazil.Para|Loricariidae|
Ancistrus sp. pimentall[88]|Brazil.Para|Loricariidae|
Ancistrus sp. pimentall[89]|Brazil.Para|Loricariidae|
Ancistrus sp. pimentall[90]|Brazil.Para|Loricariidae|
Ancistrus sp. pimentall[91]|Brazil.Para|Loricariidae|
Ancistrus sp. pimentall[92]|Brazil.Para|Loricariidae|
Ancistrus sp. pintal[93]|Brazil.Para|Loricariidae|
Ancistrus sp. pintal[94]|Brazil.Para|Loricariidae|
Ancistrus sp. pintal[95]|Brazil.Para|Loricariidae|
Ancistrus sp. pintal[96]|Brazil.Para|Loricariidae|
Ancistrus sp. pintal[97]|Brazil.Para|Loricariidae|
Ancistrus sp. pintal[98]|Brazil.Para|Loricariidae|
Ancistrus sp. pintal[99]|Brazil.Para|Loricariidae|
Unknown Specimen||
Ancistrus sp. 2
Field number: sp13.11
GenBank: KP960567

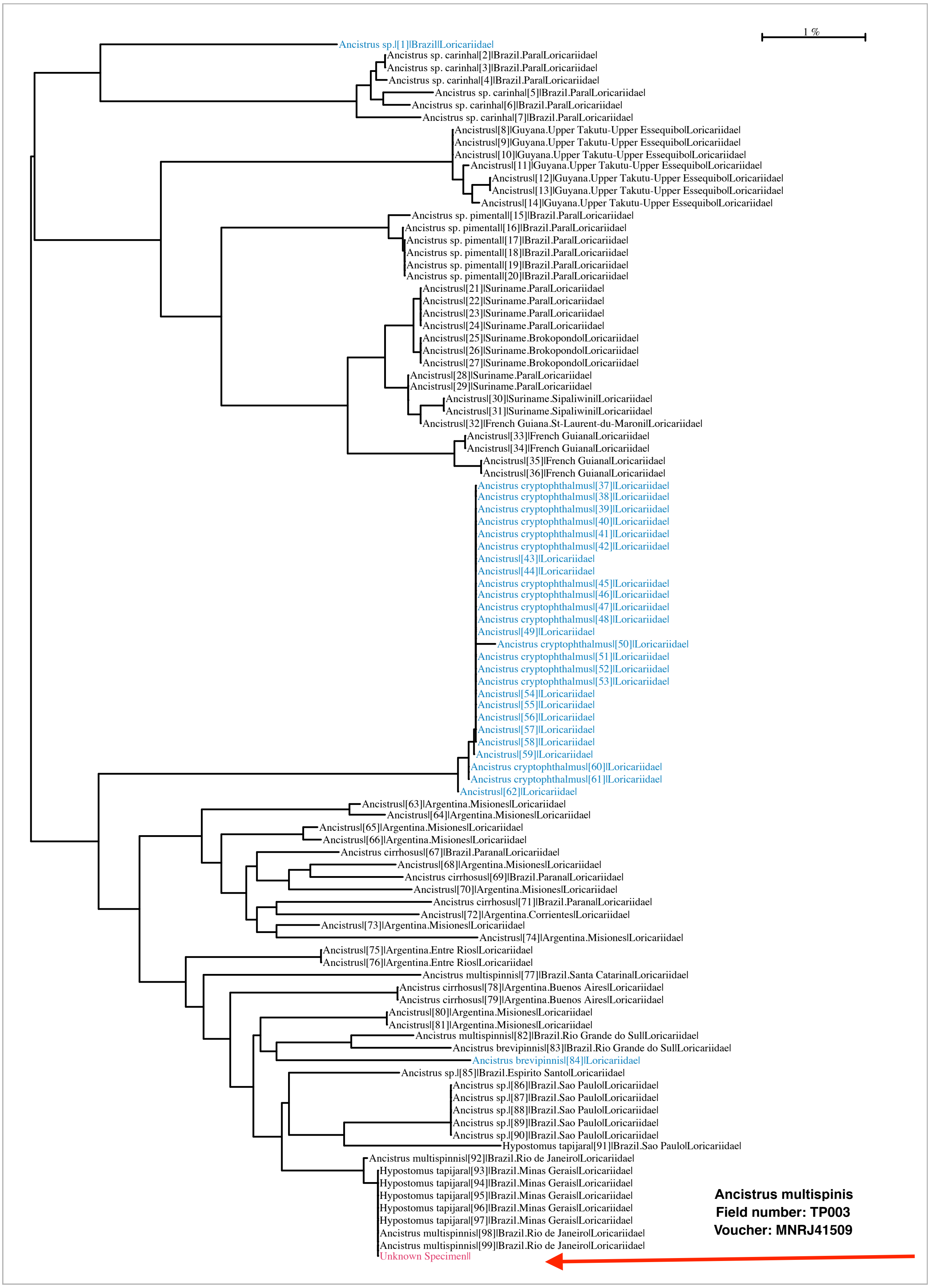
1 %
Ancistrus sp.|[1]|Brazil|Loricariidae|
Ancistrus sp. carinha|[2]|Brazil.Para|Loricariidae|
Ancistrus sp. carinha|[3]|Brazil.Para|Loricariidae|
Ancistrus sp. carinha|[4]|Brazil.Para|Loricariidae|
Ancistrus sp. carinha|[5]|Brazil.Para|Loricariidae|
Ancistrus sp. carinha|[6]|Brazil.Para|Loricariidae|
Ancistrus sp. carinha|[7]|Brazil.Para|Loricariidae|
Ancistrus|[8]|Guyana.Upper Takutu-Upper Essequibo|Loricariidae|
Ancistrus|[9]|Guyana.Upper Takutu-Upper Essequibo|Loricariidae|
Ancistrus|[10]|Guyana.Upper Takutu-Upper Essequibo|Loricariidae|
Ancistrus|[11]|Guyana.Upper Takutu-Upper Essequibo|Loricariidae|
Ancistrus|[12]|Guyana.Upper Takutu-Upper Essequibo|Loricariidae|
Ancistrus|[13]|Guyana.Upper Takutu-Upper Essequibo|Loricariidae|
Ancistrus|[14]|Guyana.Upper Takutu-Upper Essequibo|Loricariidae|
Ancistrus sp. pimenta|[15]|Brazil.Para|Loricariidae|
Ancistrus sp. pimenta|[16]|Brazil.Para|Loricariidae|
Ancistrus sp. pimenta|[17]|Brazil.Para|Loricariidae|
Ancistrus sp. pimenta|[18]|Brazil.Para|Loricariidae|
Ancistrus sp. pimenta|[19]|Brazil.Para|Loricariidae|
Ancistrus sp. pimenta|[20]|Brazil.Para|Loricariidae|
Ancistrus|[21]|Suriname.Para|Loricariidae|
Ancistrus|[22]|Suriname.Para|Loricariidae|
Ancistrus|[23]|Suriname.Para|Loricariidae|
Ancistrus|[24]|Suriname.Para|Loricariidae|
Ancistrus|[25]|Suriname.Brokopondo|Loricariidae|
Ancistrus|[26]|Suriname.Brokopondo|Loricariidae|
Ancistrus|[27]|Suriname.Brokopondo|Loricariidae|
Ancistrus|[28]|Suriname.Para|Loricariidae|
Ancistrus|[29]|Suriname.Para|Loricariidae|
Ancistrus|[30]|Suriname.Sipaliwini|Loricariidae|
Ancistrus|[31]|Suriname.Sipaliwini|Loricariidae|
Ancistrus|[32]|French Guiana.St-Laurent-du-Maroni|Loricariidae|
Ancistrus|[33]|French Guiana|Loricariidae|
Ancistrus|[34]|French Guiana|Loricariidae|
Ancistrus|[35]|French Guiana|Loricariidae|
Ancistrus|[36]|French Guiana|Loricariidae|
Ancistrus cryptophthalmus|[37]||Loricariidae|
Ancistrus cryptophthalmus|[38]||Loricariidae|
Ancistrus cryptophthalmus|[39]||Loricariidae|
Ancistrus cryptophthalmus|[40]||Loricariidae|
Ancistrus cryptophthalmus|[41]||Loricariidae|
Ancistrus cryptophthalmus|[42]||Loricariidae|
Ancistrus|[43]||Loricariidae|
Ancistrus|[44]||Loricariidae|
Ancistrus cryptophthalmus|[45]||Loricariidae|
Ancistrus cryptophthalmus|[46]||Loricariidae|
Ancistrus cryptophthalmus|[47]||Loricariidae|
Ancistrus cryptophthalmus|[48]||Loricariidae|
Ancistrus|[49]||Loricariidae|
Ancistrus cryptophthalmus|[50]||Loricariidae|
Ancistrus cryptophthalmus|[51]||Loricariidae|
Ancistrus cryptophthalmus|[52]||Loricariidae|
Ancistrus cryptophthalmus|[53]||Loricariidae|
Ancistrus|[54]||Loricariidae|
Ancistrus|[55]||Loricariidae|
Ancistrus|[56]||Loricariidae|
Ancistrus|[57]||Loricariidae|
Ancistrus|[58]||Loricariidae|
Ancistrus|[59]||Loricariidae|
Ancistrus cryptophthalmus|[60]||Loricariidae|
Ancistrus cryptophthalmus|[61]||Loricariidae|
Ancistrus|[62]||Loricariidae|
Ancistrus|[63]|Argentina.Misiones|Loricariidae|
Ancistrus|[64]|Argentina.Misiones|Loricariidae|
Ancistrus|[65]|Argentina.Misiones|Loricariidae|
Ancistrus|[66]|Argentina.Misiones|Loricariidae|
Ancistrus cirrhosus|[67]|Brazil.Parana|Loricariidae|
Ancistrus|[68]|Argentina.Misiones|Loricariidae|
Ancistrus cirrhosus|[69]|Brazil.Parana|Loricariidae|
Ancistrus|[70]|Argentina.Misiones|Loricariidae|
Ancistrus cirrhosus|[71]|Brazil.Parana|Loricariidae|
Ancistrus|[72]|Argentina.Corrientes|Loricariidae|
Ancistrus|[73]|Argentina.Misiones|Loricariidae|
Ancistrus|[74]|Argentina.Misiones|Loricariidae|
Ancistrus|[75]|Argentina.Entre Rios|Loricariidae|
Ancistrus|[76]|Argentina.Entre Rios|Loricariidae|
Ancistrus multispinnis|[77]|Brazil.Santa Catarina|Loricariidae|
Ancistrus cirrhosus|[78]|Argentina.Buenos Aires|Loricariidae|
Ancistrus cirrhosus|[79]|Argentina.Buenos Aires|Loricariidae|
Ancistrus|[80]|Argentina.Misiones|Loricariidae|
Ancistrus|[81]|Argentina.Misiones|Loricariidae|
Ancistrus multispinnis|[82]|Brazil.Rio Grande do Sul|Loricariidae|
Ancistrus brevipinnis|[83]|Brazil.Rio Grande do Sul|Loricariidae|
Ancistrus brevipinnis|[84]||Loricariidae|
Ancistrus sp.|[85]|Brazil.Espirito Santo|Loricariidae|
Ancistrus sp.|[86]|Brazil.Sao Paulo|Loricariidae|
Ancistrus sp.|[87]|Brazil.Sao Paulo|Loricariidae|
Ancistrus sp.|[88]|Brazil.Sao Paulo|Loricariidae|
Ancistrus sp.|[89]|Brazil.Sao Paulo|Loricariidae|
Ancistrus sp.|[90]|Brazil.Sao Paulo|Loricariidae|
Hypostomus tapijara|[91]|Brazil.Sao Paulo|Loricariidae|
Ancistrus multispinnis|[92]|Brazil.Rio de Janeiro|Loricariidae|
Hypostomus tapijara|[93]|Brazil.Minas Gerais|Loricariidae|
Hypostomus tapijara|[94]|Brazil.Minas Gerais|Loricariidae|
Hypostomus tapijara|[95]|Brazil.Minas Gerais|Loricariidae|
Hypostomus tapijara|[96]|Brazil.Minas Gerais|Loricariidae|
Hypostomus tapijara|[97]|Brazil.Minas Gerais|Loricariidae|
Ancistrus multispinnis|[98]|Brazil.Rio de Janeiro|Loricariidae|
Ancistrus multispinnis|[99]|Brazil.Rio de Janeiro|Loricariidae|
Unknown Specimen||
Ancistrus multispinis
Field number: TP003
Voucher: MNRJ41509

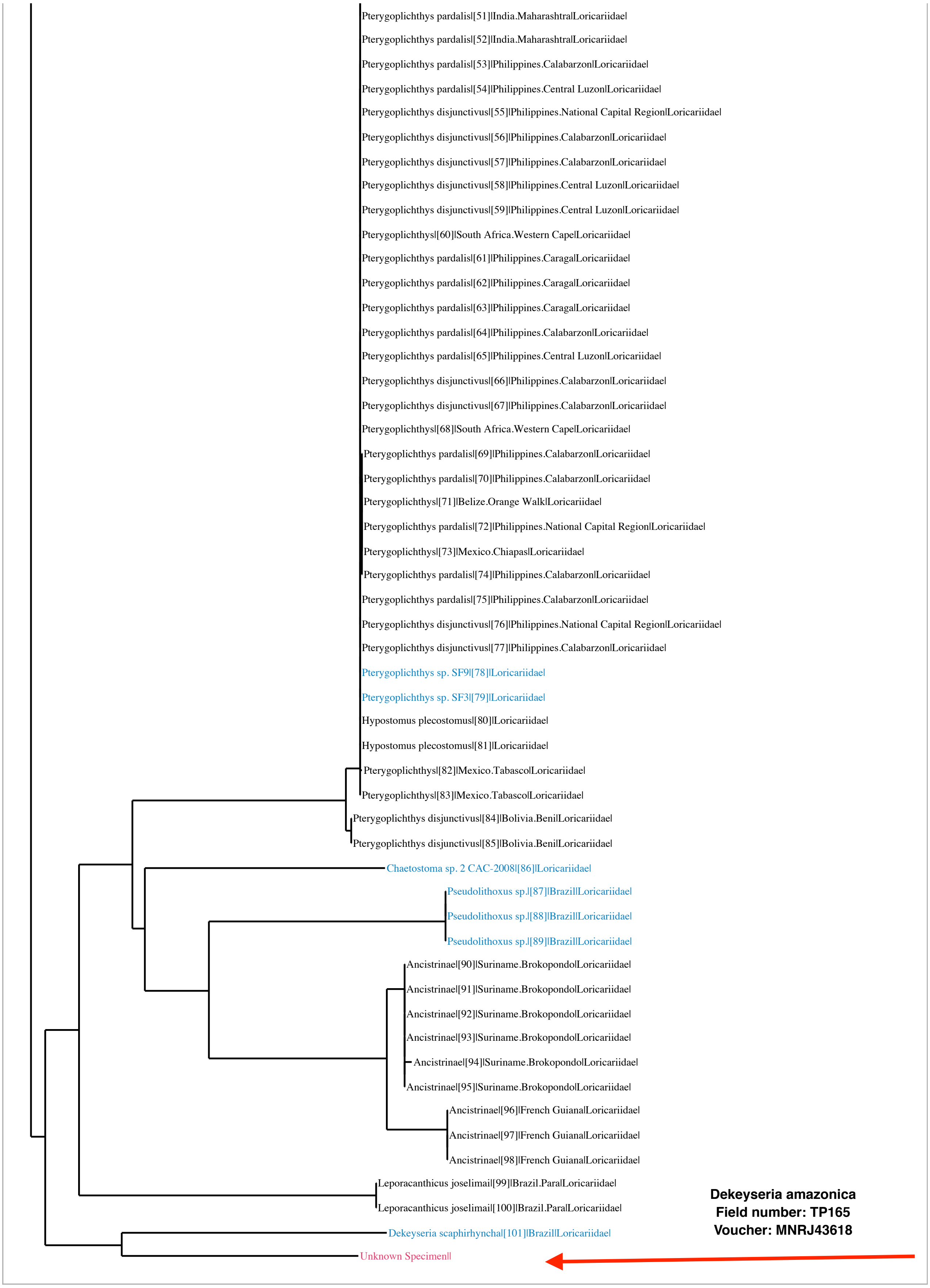
Pterygoplichthys pardalis|[51]|India.Maharashtra|Loricariidae|
Pterygoplichthys pardalis|[52]|India.Maharashtra|Loricariidae|
Pterygoplichthys pardalis|[53]|Philippines.Calabarzon|Loricariidae|
Pterygoplichthys pardalis|[54]|Philippines.Central Luzon|Loricariidae|
Pterygoplichthys disjunctivus|[55]|Philippines.National Capital Region|Loricariidae|
Pterygoplichthys disjunctivus|[56]|Philippines.Calabarzon|Loricariidae|
Pterygoplichthys disjunctivus|[57]|Philippines.Calabarzon|Loricariidae|
Pterygoplichthys disjunctivus|[58]|Philippines.Central Luzon|Loricariidae|
Pterygoplichthys disjunctivus|[59]|Philippines.Central Luzon|Loricariidae|
Pterygoplichthys|[60]|South Africa.Western Cape|Loricariidae|
Pterygoplichthys pardalis|[61]|Philippines.Caraga|Loricariidae|
Pterygoplichthys pardalis|[62]|Philippines.Caraga|Loricariidae|
Pterygoplichthys pardalis|[63]|Philippines.Caraga|Loricariidae|
Pterygoplichthys pardalis|[64]|Philippines.Calabarzon|Loricariidae|
Pterygoplichthys pardalis|[65]|Philippines.Central Luzon|Loricariidae|
Pterygoplichthys disjunctivus|[66]|Philippines.Calabarzon|Loricariidae|
Pterygoplichthys disjunctivus|[67]|Philippines.Calabarzon|Loricariidae|
Pterygoplichthys|[68]|South Africa.Western Cape|Loricariidae|
Pterygoplichthys pardalis|[69]|Philippines.Calabarzon|Loricariidae|
Pterygoplichthys pardalis|[70]|Philippines.Calabarzon|Loricariidae|
Pterygoplichthys|[71]|Belize.Orange Walk|Loricariidae|
Pterygoplichthys pardalis|[72]|Philippines.National Capital Region|Loricariidae|
Pterygoplichthys|[73]|Mexico.Chiapas|Loricariidae|
Pterygoplichthys pardalis|[74]|Philippines.Calabarzon|Loricariidae|
Pterygoplichthys pardalis|[75]|Philippines.Calabarzon|Loricariidae|
Pterygoplichthys disjunctivus|[76]|Philippines.National Capital Region|Loricariidae|
Pterygoplichthys disjunctivus|[77]|Philippines.Calabarzon|Loricariidae|
Pterygoplichthys sp. SF9|[78]|Loricariidae|
Pterygoplichthys sp. SF3|[79]|Loricariidae|
Hypostomus plecostomus|[80]|Loricariidae|
Hypostomus plecostomus|[81]|Loricariidae|
Pterygoplichthys|[82]|Mexico.Tabasco|Loricariidae|
Pterygoplichthys|[83]|Mexico.Tabasco|Loricariidae|
Pterygoplichthys disjunctivus|[84]|Bolivia.Beni|Loricariidae|
Pterygoplichthys disjunctivus|[85]|Bolivia.Beni|Loricariidae|
Chaetostoma sp. 2 CAC-2008|[86]|Loricariidae|
Pseudolithoxus sp.|[87]|Brazil|Loricariidae|
Pseudolithoxus sp.|[88]|Brazil|Loricariidae|
Pseudolithoxus sp.|[89]|Brazil|Loricariidae|
Ancistrinae|[90]|Suriname.Brokopondo|Loricariidae|
Ancistrinae|[91]|Suriname.Brokopondo|Loricariidae|
Ancistrinae|[92]|Suriname.Brokopondo|Loricariidae|
Ancistrinae|[93]|Suriname.Brokopondo|Loricariidae|
Ancistrinae|[94]|Suriname.Brokopondo|Loricariidae|
Ancistrinae|[95]|Suriname.Brokopondo|Loricariidae|
Ancistrinae|[96]|French Guiana|Loricariidae|
Ancistrinae|[97]|French Guiana|Loricariidae|
Ancistrinae|[98]|French Guiana|Loricariidae|
Leporacanthicus joselimai|[99]|Brazil.Para|Loricariidae|
Leporacanthicus joselimai|[100]|Brazil.Para|Loricariidae|
Dekeyseria scaphirhyncha|[101]|Brazil|Loricariidae|
Unknown Specimen||
Dekeyseria amazonica
Field number: TP165
Voucher: MNRJ43618

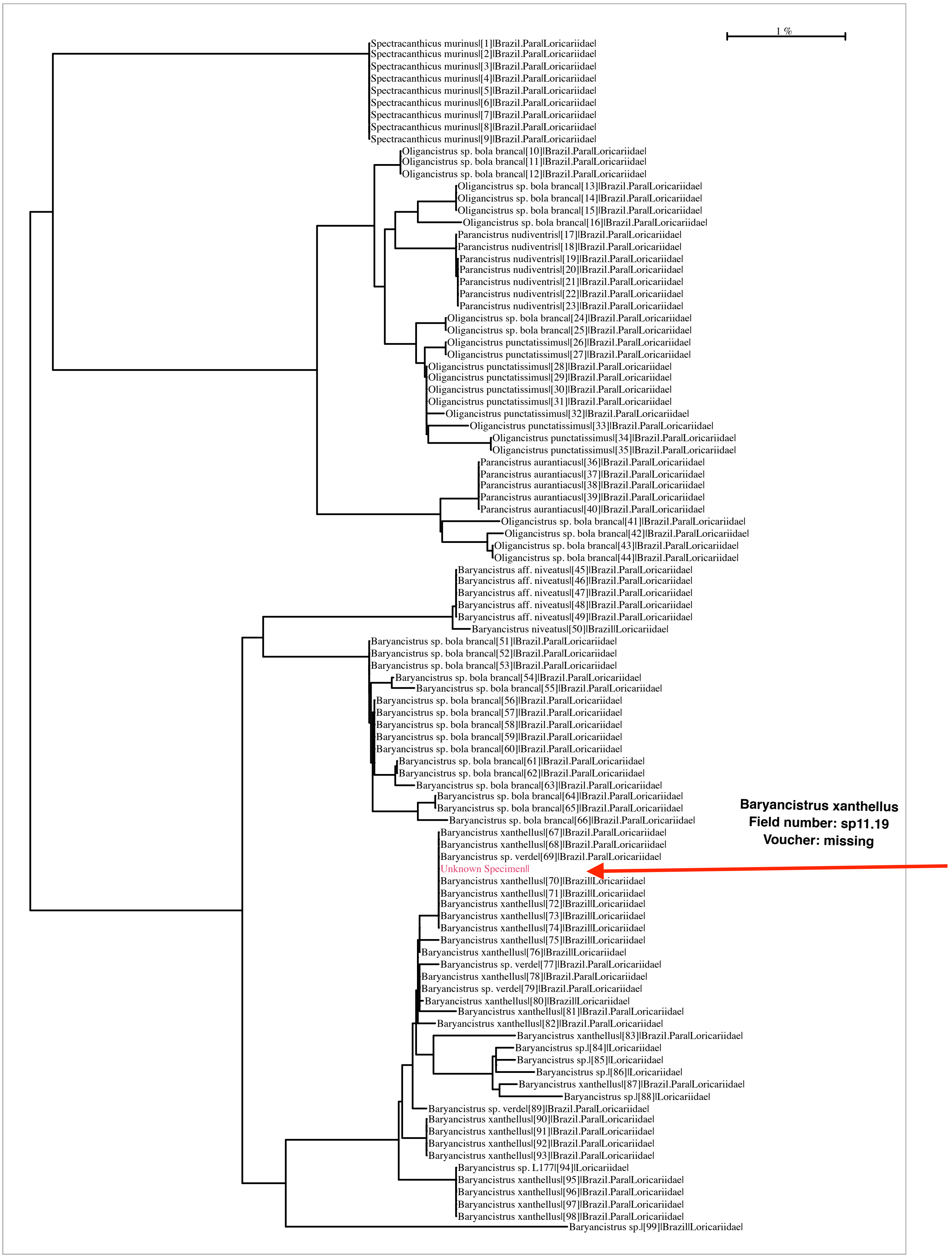
1 %
Spectracanthicus murinus|[1]|Brazil.Para|Loricariidae|
Spectracanthicus murinus|[2]|Brazil.Para|Loricariidae|
Spectracanthicus murinus|[3]|Brazil.Para|Loricariidae|
Spectracanthicus murinus|[4]|Brazil.Para|Loricariidae|
Spectracanthicus murinus|[5]|Brazil.Para|Loricariidae|
Spectracanthicus murinus|[6]|Brazil.Para|Loricariidae|
Spectracanthicus murinus|[7]|Brazil.Para|Loricariidae|
Spectracanthicus murinus|[8]|Brazil.Para|Loricariidae|
Spectracanthicus murinus|[9]|Brazil.Para|Loricariidae|
Oligancistrus sp. bola branca|[10]|Brazil.Para|Loricariidae|
Oligancistrus sp. bola branca|[11]|Brazil.Para|Loricariidae|
Oligancistrus sp. bola branca|[12]|Brazil.Para|Loricariidae|
Oligancistrus sp. bola branca|[13]|Brazil.Para|Loricariidae|
Oligancistrus sp. bola branca|[14]|Brazil.Para|Loricariidae|
Oligancistrus sp. bola branca|[15]|Brazil.Para|Loricariidae|
Oligancistrus sp. bola branca|[16]|Brazil.Para|Loricariidae|
Parancistrus nudiventris|[17]|Brazil.Para|Loricariidae|
Parancistrus nudiventris|[18]|Brazil.Para|Loricariidae|
Parancistrus nudiventris|[19]|Brazil.Para|Loricariidae|
Parancistrus nudiventris|[20]|Brazil.Para|Loricariidae|
Parancistrus nudiventris|[21]|Brazil.Para|Loricariidae|
Parancistrus nudiventris|[22]|Brazil.Para|Loricariidae|
Parancistrus nudiventris|[23]|Brazil.Para|Loricariidae|
Oligancistrus sp. bola branca|[24]|Brazil.Para|Loricariidae|
Oligancistrus sp. bola branca|[25]|Brazil.Para|Loricariidae|
Oligancistrus punctatissimus|[26]|Brazil.Para|Loricariidae|
Oligancistrus punctatissimus|[27]|Brazil.Para|Loricariidae|
Oligancistrus punctatissimus|[28]|Brazil.Para|Loricariidae|
Oligancistrus punctatissimus|[29]|Brazil.Para|Loricariidae|
Oligancistrus punctatissimus|[30]|Brazil.Para|Loricariidae|
Oligancistrus punctatissimus|[31]|Brazil.Para|Loricariidae|
Oligancistrus punctatissimus|[32]|Brazil.Para|Loricariidae|
Oligancistrus punctatissimus|[33]|Brazil.Para|Loricariidae|
Oligancistrus punctatissimus|[34]|Brazil.Para|Loricariidae|
Oligancistrus punctatissimus|[35]|Brazil.Para|Loricariidae|
Parancistrus aurantiacus|[36]|Brazil.Para|Loricariidae|
Parancistrus aurantiacus|[37]|Brazil.Para|Loricariidae|
Parancistrus aurantiacus|[38]|Brazil.Para|Loricariidae|
Parancistrus aurantiacus|[39]|Brazil.Para|Loricariidae|
Parancistrus aurantiacus|[40]|Brazil.Para|Loricariidae|
Oligancistrus sp. bola branca|[41]|Brazil.Para|Loricariidae|
Oligancistrus sp. bola branca|[42]|Brazil.Para|Loricariidae|
Oligancistrus sp. bola branca|[43]|Brazil.Para|Loricariidae|
Oligancistrus sp. bola branca|[44]|Brazil.Para|Loricariidae|
Baryancistrus aff. niveatus|[45]|Brazil.Para|Loricariidae|
Baryancistrus aff. niveatus|[46]|Brazil.Para|Loricariidae|
Baryancistrus aff. niveatus|[47]|Brazil.Para|Loricariidae|
Baryancistrus aff. niveatus|[48]|Brazil.Para|Loricariidae|
Baryancistrus aff. niveatus|[49]|Brazil.Para|Loricariidae|
Baryancistrus niveatus|[50]|Brazil||Loricariidae|
Baryancistrus sp. bola branca|[51]|Brazil.Para|Loricariidae|
Baryancistrus sp. bola branca|[52]|Brazil.Para|Loricariidae|
Baryancistrus sp. bola branca|[53]|Brazil.Para|Loricariidae|
Baryancistrus sp. bola branca|[54]|Brazil.Para|Loricariidae|
Baryancistrus sp. bola branca|[55]|Brazil.Para|Loricariidae|
Baryancistrus sp. bola branca|[56]|Brazil.Para|Loricariidae|
Baryancistrus sp. bola branca|[57]|Brazil.Para|Loricariidae|
Baryancistrus sp. bola branca|[58]|Brazil.Para|Loricariidae|
Baryancistrus sp. bola branca|[59]|Brazil.Para|Loricariidae|
Baryancistrus sp. bola branca|[60]|Brazil.Para|Loricariidae|
Baryancistrus sp. bola branca|[61]|Brazil.Para|Loricariidae|
Baryancistrus sp. bola branca|[62]|Brazil.Para|Loricariidae|
Baryancistrus sp. bola branca|[63]|Brazil.Para|Loricariidae|
Baryancistrus sp. bola branca|[64]|Brazil.Para|Loricariidae|
Baryancistrus sp. bola branca|[65]|Brazil.Para|Loricariidae|
Baryancistrus sp. bola branca|[66]|Brazil.Para|Loricariidae|
Baryancistrus xanthellus|[67]|Brazil.Para|Loricariidae|
Baryancistrus xanthellus|[68]|Brazil.Para|Loricariidae|
Baryancistrus sp. verde|[69]|Brazil.Para|Loricariidae|
Unknown Specimen||
Baryancistrus xanthellus|[70]|Brazil||Loricariidae|
Baryancistrus xanthellus|[71]|Brazil||Loricariidae|
Baryancistrus xanthellus|[72]|Brazil||Loricariidae|
Baryancistrus xanthellus|[73]|Brazil||Loricariidae|
Baryancistrus xanthellus|[74]|Brazil||Loricariidae|
Baryancistrus xanthellus|[75]|Brazil||Loricariidae|
Baryancistrus xanthellus|[76]|Brazil||Loricariidae|
Baryancistrus sp. verde|[77]|Brazil.Para|Loricariidae|
Baryancistrus xanthellus|[78]|Brazil.Para|Loricariidae|
Baryancistrus sp. verde|[79]|Brazil.Para|Loricariidae|
Baryancistrus xanthellus|[80]|Brazil||Loricariidae|
Baryancistrus xanthellus|[81]|Brazil.Para|Loricariidae|
Baryancistrus xanthellus|[82]|Brazil.Para|Loricariidae|
Baryancistrus xanthellus|[83]|Brazil.Para|Loricariidae|
Baryancistrus sp.|[84]||Loricariidae|
Baryancistrus sp.|[85]||Loricariidae|
Baryancistrus sp.|[86]||Loricariidae|
Baryancistrus xanthellus|[87]|Brazil.Para|Loricariidae|
Baryancistrus sp.|[88]||Loricariidae|
Baryancistrus sp. verde|[89]|Brazil.Para|Loricariidae|
Baryancistrus xanthellus|[90]|Brazil.Para|Loricariidae|
Baryancistrus xanthellus|[91]|Brazil.Para|Loricariidae|
Baryancistrus xanthellus|[92]|Brazil.Para|Loricariidae|
Baryancistrus xanthellus|[93]|Brazil.Para|Loricariidae|
Baryancistrus sp. L177|[94]||Loricariidae|
Baryancistrus xanthellus|[95]|Brazil.Para|Loricariidae|
Baryancistrus xanthellus|[96]|Brazil.Para|Loricariidae|
Baryancistrus xanthellus|[97]|Brazil.Para|Loricariidae|
Baryancistrus xanthellus|[98]|Brazil.Para|Loricariidae|
Baryancistrus sp.|[99]|Brazil||Loricariidae|
Baryancistrus xanthellus
Field number: sp11.19
Voucher: missing

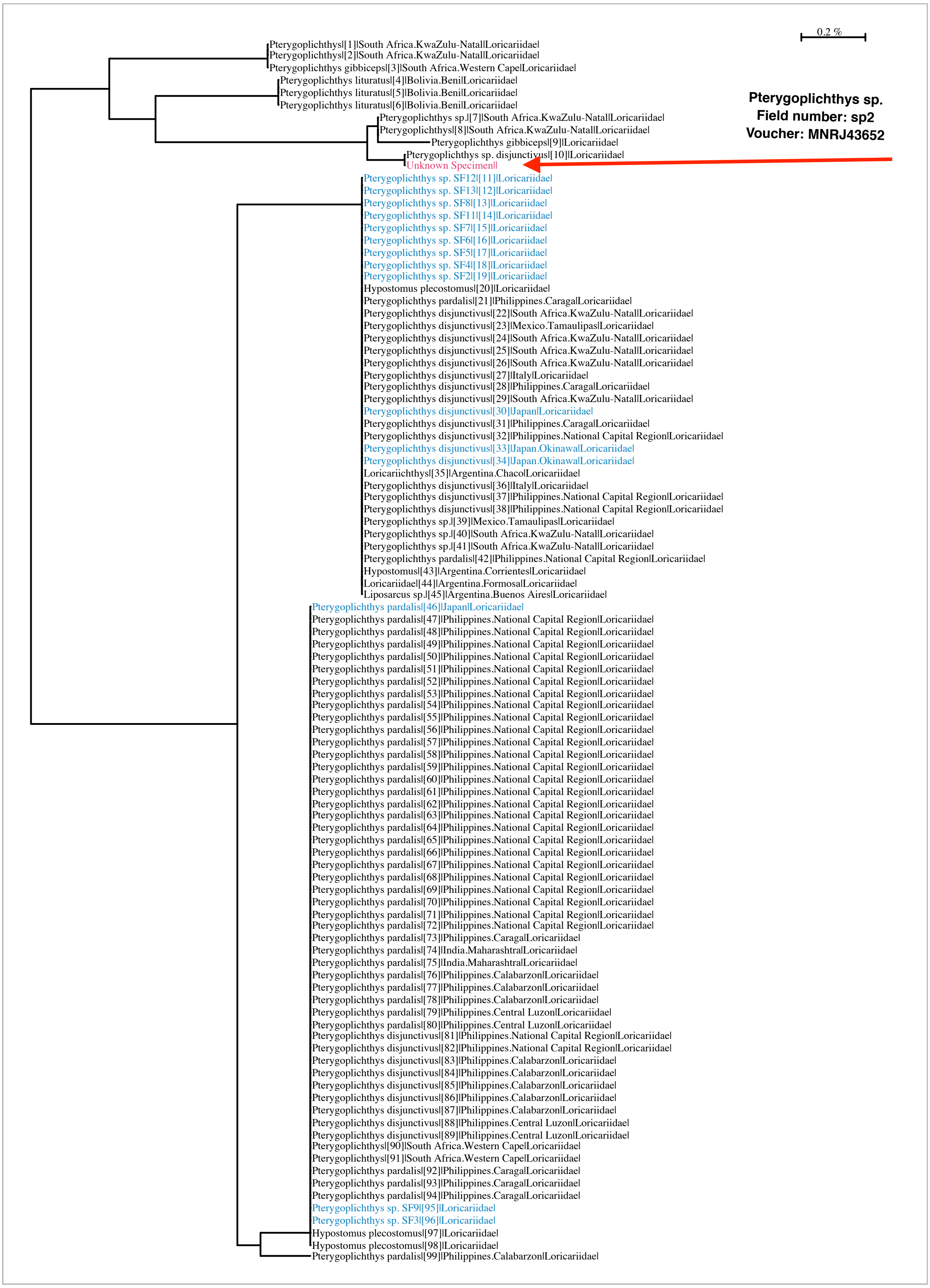
0.2 %
Pterygoplichthys sp.
Field number: sp2
Voucher: MNRJ43652
Pterygoplichthys|[1]|South Africa.KwaZulu-Natal|Loricariidae|
Pterygoplichthys|[2]|South Africa.KwaZulu-Natal|Loricariidae|
Pterygoplichthys gibbiceps|[3]|South Africa.Western Cape|Loricariidae|
Pterygoplichthys lituratus|[4]|Bolivia.Beni|Loricariidae|
Pterygoplichthys lituratus|[5]|Bolivia.Beni|Loricariidae|
Pterygoplichthys lituratus|[6]|Bolivia.Beni|Loricariidae|
Pterygoplichthys sp.|[7]|South Africa.KwaZulu-Natal|Loricariidae|
Pterygoplichthys|[8]|South Africa.KwaZulu-Natal|Loricariidae|
Pterygoplichthys gibbiceps|[9]||Loricariidae|
Pterygoplichthys sp. disjunctivus|[10]||Loricariidae|
Unknown Specimen||
Pterygoplichthys sp. SF12|[11]||Loricariidae|
Pterygoplichthys sp. SF13|[12]||Loricariidae|
Pterygoplichthys sp. SF8|[13]||Loricariidae|
Pterygoplichthys sp. SF11|[14]||Loricariidae|
Pterygoplichthys sp. SF7|[15]||Loricariidae|
Pterygoplichthys sp. SF6|[16]||Loricariidae|
Pterygoplichthys sp. SF5|[17]||Loricariidae|
Pterygoplichthys sp. SF4|[18]||Loricariidae|
Pterygoplichthys sp. SF2|[19]||Loricariidae|
Hypostomus plecostomus|[20]||Loricariidae|
Pterygoplichthys pardalis|[21]|Philippines.Caraga|Loricariidae|
Pterygoplichthys disjunctivus|[22]|South Africa.KwaZulu-Natal|Loricariidae|
Pterygoplichthys disjunctivus|[23]|Mexico.Tamaulipas|Loricariidae|
Pterygoplichthys disjunctivus|[24]|South Africa.KwaZulu-Natal|Loricariidae|
Pterygoplichthys disjunctivus|[25]|South Africa.KwaZulu-Natal|Loricariidae|
Pterygoplichthys disjunctivus|[26]|South Africa.KwaZulu-Natal|Loricariidae|
Pterygoplichthys disjunctivus|[27]|Italy|Loricariidae|
Pterygoplichthys disjunctivus|[28]|Philippines.Caraga|Loricariidae|
Pterygoplichthys disjunctivus|[29]|South Africa.KwaZulu-Natal|Loricariidae|
Pterygoplichthys disjunctivus|[30]|Japan|Loricariidae|
Pterygoplichthys disjunctivus|[31]|Philippines.Caraga|Loricariidae|
Pterygoplichthys disjunctivus|[32]|Philippines.National Capital Region|Loricariidae|
Pterygoplichthys disjunctivus|[33]|Japan.Okinawa|Loricariidae|
Pterygoplichthys disjunctivus|[34]|Japan.Okinawa|Loricariidae|
Loricariichthys|[35]|Argentina.Chaco|Loricariidae|
Pterygoplichthys disjunctivus|[36]|Italy|Loricariidae|
Pterygoplichthys disjunctivus|[37]|Philippines.National Capital Region|Loricariidae|
Pterygoplichthys disjunctivus|[38]|Philippines.National Capital Region|Loricariidae|
Pterygoplichthys sp.|[39]|Mexico.Tamaulipas|Loricariidae|
Pterygoplichthys sp.|[40]|South Africa.KwaZulu-Natal|Loricariidae|
Pterygoplichthys sp.|[41]|South Africa.KwaZulu-Natal|Loricariidae|
Pterygoplichthys pardalis|[42]|Philippines.National Capital Region|Loricariidae|
Hypostomus|[43]|Argentina.Corrientes|Loricariidae|
Loricariidae|[44]|Argentina.Formosa|Loricariidae|
Liposarcus sp.|[45]|Argentina.Buenos Aires|Loricariidae|
Pterygoplichthys pardalis|[46]|Japan|Loricariidae|
Pterygoplichthys pardalis|[47]|Philippines.National Capital Region|Loricariidae|
Pterygoplichthys pardalis|[48]|Philippines.National Capital Region|Loricariidae|
Pterygoplichthys pardalis|[49]|Philippines.National Capital Region|Loricariidae|
Pterygoplichthys pardalis|[50]|Philippines.National Capital Region|Loricariidae|
Pterygoplichthys pardalis|[51]|Philippines.National Capital Region|Loricariidae|
Pterygoplichthys pardalis|[52]|Philippines.National Capital Region|Loricariidae|
Pterygoplichthys pardalis|[53]|Philippines.National Capital Region|Loricariidae|
Pterygoplichthys pardalis|[54]|Philippines.National Capital Region|Loricariidae|
Pterygoplichthys pardalis|[55]|Philippines.National Capital Region|Loricariidae|
Pterygoplichthys pardalis|[56]|Philippines.National Capital Region|Loricariidae|
Pterygoplichthys pardalis|[57]|Philippines.National Capital Region|Loricariidae|
Pterygoplichthys pardalis|[58]|Philippines.National Capital Region|Loricariidae|
Pterygoplichthys pardalis|[59]|Philippines.National Capital Region|Loricariidae|
Pterygoplichthys pardalis|[60]|Philippines.National Capital Region|Loricariidae|
Pterygoplichthys pardalis|[61]|Philippines.National Capital Region|Loricariidae|
Pterygoplichthys pardalis|[62]|Philippines.National Capital Region|Loricariidae|
Pterygoplichthys pardalis|[63]|Philippines.National Capital Region|Loricariidae|
Pterygoplichthys pardalis|[64]|Philippines.National Capital Region|Loricariidae|
Pterygoplichthys pardalis|[65]|Philippines.National Capital Region|Loricariidae|
Pterygoplichthys pardalis|[66]|Philippines.National Capital Region|Loricariidae|
Pterygoplichthys pardalis|[67]|Philippines.National Capital Region|Loricariidae|
Pterygoplichthys pardalis|[68]|Philippines.National Capital Region|Loricariidae|
Pterygoplichthys pardalis|[69]|Philippines.National Capital Region|Loricariidae|
Pterygoplichthys pardalis|[70]|Philippines.National Capital Region|Loricariidae|
Pterygoplichthys pardalis|[71]|Philippines.National Capital Region|Loricariidae|
Pterygoplichthys pardalis|[72]|Philippines.National Capital Region|Loricariidae|
Pterygoplichthys pardalis|[73]|Philippines.Caraga|Loricariidae|
Pterygoplichthys pardalis|[74]|India.Maharashtra|Loricariidae|
Pterygoplichthys pardalis|[75]|India.Maharashtra|Loricariidae|
Pterygoplichthys pardalis|[76]|Philippines.Calabarzon|Loricariidae|
Pterygoplichthys pardalis|[77]|Philippines.Calabarzon|Loricariidae|
Pterygoplichthys pardalis|[78]|Philippines.Calabarzon|Loricariidae|
Pterygoplichthys pardalis|[79]|Philippines.Central Luzon|Loricariidae|
Pterygoplichthys pardalis|[80]|Philippines.Central Luzon|Loricariidae|
Pterygoplichthys disjunctivus|[81]|Philippines.National Capital Region|Loricariidae|
Pterygoplichthys disjunctivus|[82]|Philippines.National Capital Region|Loricariidae|
Pterygoplichthys disjunctivus|[83]|Philippines.Calabarzon|Loricariidae|
Pterygoplichthys disjunctivus|[84]|Philippines.Calabarzon|Loricariidae|
Pterygoplichthys disjunctivus|[85]|Philippines.Calabarzon|Loricariidae|
Pterygoplichthys disjunctivus|[86]|Philippines.Calabarzon|Loricariidae|
Pterygoplichthys disjunctivus|[87]|Philippines.Calabarzon|Loricariidae|
Pterygoplichthys disjunctivus|[88]|Philippines.Central Luzon|Loricariidae|
Pterygoplichthys disjunctivus|[89]|Philippines.Central Luzon|Loricariidae|
Pterygoplichthys|[90]|South Africa.Western Cape|Loricariidae|
Pterygoplichthys|[91]|South Africa.Western Cape|Loricariidae|
Pterygoplichthys pardalis|[92]|Philippines.Caraga|Loricariidae|
Pterygoplichthys pardalis|[93]|Philippines.Caraga|Loricariidae|
Pterygoplichthys pardalis|[94]|Philippines.Caraga|Loricariidae|
Pterygoplichthys sp. SF9|[95]||Loricariidae|
Pterygoplichthys sp. SF3|[96]||Loricariidae|
Hypostomus plecostomus|[97]||Loricariidae|
Hypostomus plecostomus|[98]||Loricariidae|
Pterygoplichthys pardalis|[99]|Philippines.Calabarzon|Loricariidae|

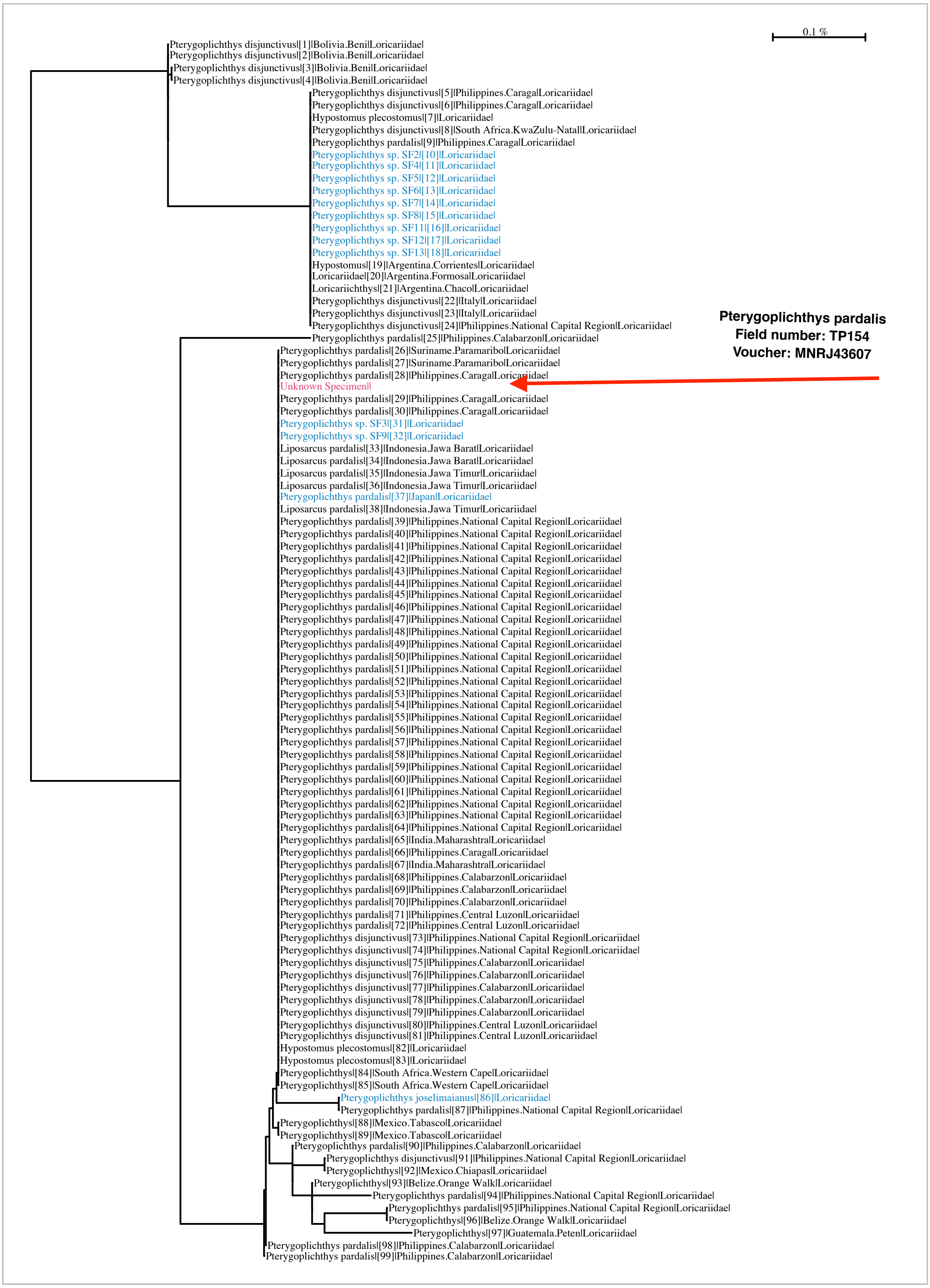
0.1 %
Pterygoplichthys disjunctivus|[1]|Bolivia.Beni|Loricariidae|
Pterygoplichthys disjunctivus|[2]|Bolivia.Beni|Loricariidae|
Pterygoplichthys disjunctivus|[3]|Bolivia.Beni|Loricariidae|
Pterygoplichthys disjunctivus|[4]|Bolivia.Beni|Loricariidae|
Pterygoplichthys disjunctivus|[5]|Philippines.Caraga|Loricariidae|
Pterygoplichthys disjunctivus|[6]|Philippines.Caraga|Loricariidae|
Hypostomus plecostomus|[7]||Loricariidae|
Pterygoplichthys disjunctivus|[8]|South Africa.KwaZulu-Natal|Loricariidae|
Pterygoplichthys pardalis|[9]|Philippines.Caraga|Loricariidae|
Pterygoplichthys sp. SF2|[10]||Loricariidae|
Pterygoplichthys sp. SF4|[11]||Loricariidae|
Pterygoplichthys sp. SF5|[12]||Loricariidae|
Pterygoplichthys sp. SF6|[13]||Loricariidae|
Pterygoplichthys sp. SF7|[14]||Loricariidae|
Pterygoplichthys sp. SF8|[15]||Loricariidae|
Pterygoplichthys sp. SF11|[16]||Loricariidae|
Pterygoplichthys sp. SF12|[17]||Loricariidae|
Pterygoplichthys sp. SF13|[18]||Loricariidae|
Hypostomus|[19]|Argentina.Corrientes|Loricariidae|
Loricariidae|[20]|Argentina.Formosa|Loricariidae|
Loricariichthys|[21]|Argentina.Chaco|Loricariidae|
Pterygoplichthys disjunctivus|[22]|Italy|Loricariidae|
Pterygoplichthys disjunctivus|[23]|Italy|Loricariidae|
Pterygoplichthys disjunctivus|[24]|Philippines.National Capital Region|Loricariidae|
Pterygoplichthys pardalis|[25]|Philippines.Calabarzon|Loricariidae|
Pterygoplichthys pardalis|[26]|Suriname.Paramaribo|Loricariidae|
Pterygoplichthys pardalis|[27]|Suriname.Paramaribo|Loricariidae|
Pterygoplichthys pardalis|[28]|Philippines.Caraga|Loricariidae|
Unknown Specimen||
Pterygoplichthys pardalis|[29]|Philippines.Caraga|Loricariidae|
Pterygoplichthys pardalis|[30]|Philippines.Caraga|Loricariidae|
Pterygoplichthys sp. SF3|[31]||Loricariidae|
Pterygoplichthys sp. SF9|[32]||Loricariidae|
Liposarcus pardalis|[33]|Indonesia.Jawa Barat|Loricariidae|
Liposarcus pardalis|[34]|Indonesia.Jawa Barat|Loricariidae|
Liposarcus pardalis|[35]|Indonesia.Jawa Timur|Loricariidae|
Liposarcus pardalis|[36]|Indonesia.Jawa Timur|Loricariidae|
Pterygoplichthys pardalis|[37]|Japan|Loricariidae|
Liposarcus pardalis|[38]|Indonesia.Jawa Timur|Loricariidae|
Pterygoplichthys pardalis|[39]|Philippines.National Capital Region|Loricariidae|
Pterygoplichthys pardalis|[40]|Philippines.National Capital Region|Loricariidae|
Pterygoplichthys pardalis|[41]|Philippines.National Capital Region|Loricariidae|
Pterygoplichthys pardalis|[42]|Philippines.National Capital Region|Loricariidae|
Pterygoplichthys pardalis|[43]|Philippines.National Capital Region|Loricariidae|
Pterygoplichthys pardalis|[44]|Philippines.National Capital Region|Loricariidae|
Pterygoplichthys pardalis|[45]|Philippines.National Capital Region|Loricariidae|
Pterygoplichthys pardalis|[46]|Philippines.National Capital Region|Loricariidae|
Pterygoplichthys pardalis|[47]|Philippines.National Capital Region|Loricariidae|
Pterygoplichthys pardalis|[48]|Philippines.National Capital Region|Loricariidae|
Pterygoplichthys pardalis|[49]|Philippines.National Capital Region|Loricariidae|
Pterygoplichthys pardalis|[50]|Philippines.National Capital Region|Loricariidae|
Pterygoplichthys pardalis|[51]|Philippines.National Capital Region|Loricariidae|
Pterygoplichthys pardalis|[52]|Philippines.National Capital Region|Loricariidae|
Pterygoplichthys pardalis|[53]|Philippines.National Capital Region|Loricariidae|
Pterygoplichthys pardalis|[54]|Philippines.National Capital Region|Loricariidae|
Pterygoplichthys pardalis|[55]|Philippines.National Capital Region|Loricariidae|
Pterygoplichthys pardalis|[56]|Philippines.National Capital Region|Loricariidae|
Pterygoplichthys pardalis|[57]|Philippines.National Capital Region|Loricariidae|
Pterygoplichthys pardalis|[58]|Philippines.National Capital Region|Loricariidae|
Pterygoplichthys pardalis|[59]|Philippines.National Capital Region|Loricariidae|
Pterygoplichthys pardalis|[60]|Philippines.National Capital Region|Loricariidae|
Pterygoplichthys pardalis|[61]|Philippines.National Capital Region|Loricariidae|
Pterygoplichthys pardalis|[62]|Philippines.National Capital Region|Loricariidae|
Pterygoplichthys pardalis|[63]|Philippines.National Capital Region|Loricariidae|
Pterygoplichthys pardalis|[64]|Philippines.National Capital Region|Loricariidae|
Pterygoplichthys pardalis|[65]|India.Maharashtra|Loricariidae|
Pterygoplichthys pardalis|[66]|Philippines.Caraga|Loricariidae|
Pterygoplichthys pardalis|[67]|India.Maharashtra|Loricariidae|
Pterygoplichthys pardalis|[68]|Philippines.Calabarzon|Loricariidae|
Pterygoplichthys pardalis|[69]|Philippines.Calabarzon|Loricariidae|
Pterygoplichthys pardalis|[70]|Philippines.Calabarzon|Loricariidae|
Pterygoplichthys pardalis|[71]|Philippines.Central Luzon|Loricariidae|
Pterygoplichthys pardalis|[72]|Philippines.Central Luzon|Loricariidae|
Pterygoplichthys disjunctivus|[73]|Philippines.National Capital Region|Loricariidae|
Pterygoplichthys disjunctivus|[74]|Philippines.National Capital Region|Loricariidae|
Pterygoplichthys disjunctivus|[75]|Philippines.Calabarzon|Loricariidae|
Pterygoplichthys disjunctivus|[76]|Philippines.Calabarzon|Loricariidae|
Pterygoplichthys disjunctivus|[77]|Philippines.Calabarzon|Loricariidae|
Pterygoplichthys disjunctivus|[78]|Philippines.Calabarzon|Loricariidae|
Pterygoplichthys disjunctivus|[79]|Philippines.Calabarzon|Loricariidae|
Pterygoplichthys disjunctivus|[80]|Philippines.Central Luzon|Loricariidae|
Pterygoplichthys disjunctivus|[81]|Philippines.Central Luzon|Loricariidae|
Hypostomus plecostomus|[82]||Loricariidae|
Hypostomus plecostomus|[83]||Loricariidae|
Pterygoplichthys|[84]|South Africa.Western Cape|Loricariidae|
Pterygoplichthys|[85]|South Africa.Western Cape|Loricariidae|
Pterygoplichthys joselimaianus|[86]||Loricariidae|
Pterygoplichthys pardalis|[87]|Philippines.National Capital Region|Loricariidae|
Pterygoplichthys|[88]|Mexico.Tabasco|Loricariidae|
Pterygoplichthys|[89]|Mexico.Tabasco|Loricariidae|
Pterygoplichthys pardalis|[90]|Philippines.Calabarzon|Loricariidae|
Pterygoplichthys disjunctivus|[91]|Philippines.National Capital Region|Loricariidae|
Pterygoplichthys|[92]|Mexico.Chiapas|Loricariidae|
Pterygoplichthys|[93]|Belize.Orange Walk|Loricariidae|
Pterygoplichthys pardalis|[94]|Philippines.National Capital Region|Loricariidae|
Pterygoplichthys pardalis|[95]|Philippines.National Capital Region|Loricariidae|
Pterygoplichthys|[96]|Belize.Orange Walk|Loricariidae|
Pterygoplichthys|[97]|Guatemala.Peten|Loricariidae|
Pterygoplichthys pardalis|[98]|Philippines.Calabarzon|Loricariidae|
Pterygoplichthys pardalis|[99]|Philippines.Calabarzon|Loricariidae|
Pterygoplichthys pardalis
Field number: TP154
Voucher: MNRJ43607

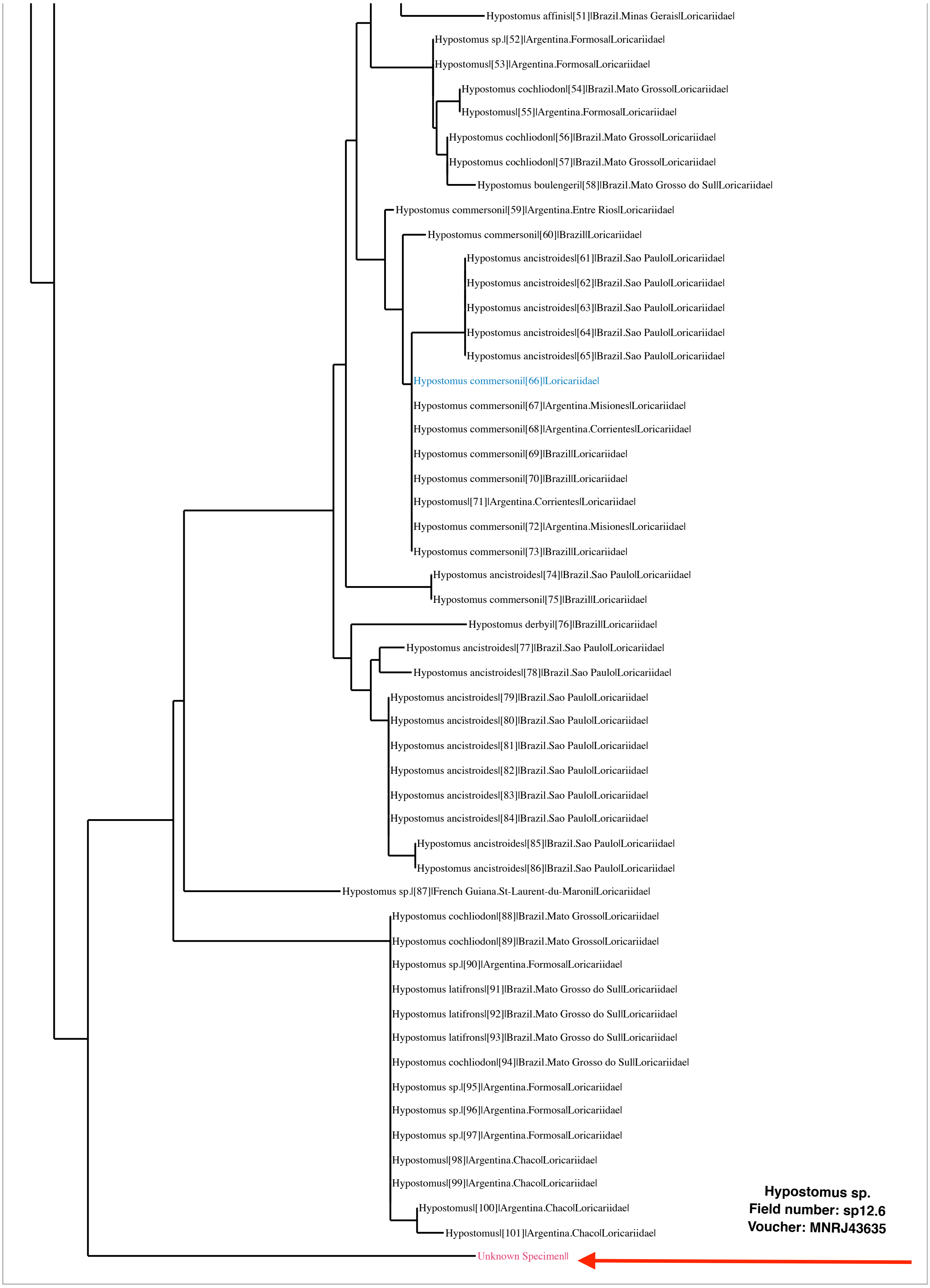
Hypostomus affinis|[51]|Brazil.Minas Gerais|Loricariidae|
Hypostomus sp.|[52]|Argentina.Formosa|Loricariidae|
Hypostomus|[53]|Argentina.Formosa|Loricariidae|
Hypostomus cochliodon|[54]|Brazil.Mato Grosso|Loricariidae|
Hypostomus|[55]|Argentina.Formosa|Loricariidae|
Hypostomus cochliodon|[56]|Brazil.Mato Grosso|Loricariidae|
Hypostomus cochliodon|[57]|Brazil.Mato Grosso|Loricariidae|
Hypostomus boulengeri|[58]|Brazil.Mato Grosso do Sul|Loricariidae|
Hypostomus commersoni|[59]|Argentina.Entre Rios|Loricariidae|
Hypostomus commersoni|[60]|Brazil|Loricariidae|
Hypostomus ancistroides|[61]|Brazil.Sao Paulo|Loricariidae|
Hypostomus ancistroides|[62]|Brazil.Sao Paulo|Loricariidae|
Hypostomus ancistroides|[63]|Brazil.Sao Paulo|Loricariidae|
Hypostomus ancistroides|[64]|Brazil.Sao Paulo|Loricariidae|
Hypostomus ancistroides|[65]|Brazil.Sao Paulo|Loricariidae|
Hypostomus commersoni|[66]|Loricariidae|
Hypostomus commersoni|[67]|Argentina.Misiones|Loricariidae|
Hypostomus commersoni|[68]|Argentina.Corrientes|Loricariidae|
Hypostomus commersoni|[69]|Brazil|Loricariidae|
Hypostomus commersoni|[70]|Brazil|Loricariidae|
Hypostomus|[71]|Argentina.Corrientes|Loricariidae|
Hypostomus commersoni|[72]|Argentina.Misiones|Loricariidae|
Hypostomus commersoni|[73]|Brazil|Loricariidae|
Hypostomus ancistroides|[74]|Brazil.Sao Paulo|Loricariidae|
Hypostomus commersoni|[75]|Brazil|Loricariidae|
Hypostomus derbyi|[76]|Brazil|Loricariidae|
Hypostomus ancistroides|[77]|Brazil.Sao Paulo|Loricariidae|
Hypostomus ancistroides|[78]|Brazil.Sao Paulo|Loricariidae|
Hypostomus ancistroides|[79]|Brazil.Sao Paulo|Loricariidae|
Hypostomus ancistroides|[80]|Brazil.Sao Paulo|Loricariidae|
Hypostomus ancistroides|[81]|Brazil.Sao Paulo|Loricariidae|
Hypostomus ancistroides|[82]|Brazil.Sao Paulo|Loricariidae|
Hypostomus ancistroides|[83]|Brazil.Sao Paulo|Loricariidae|
Hypostomus ancistroides|[84]|Brazil.Sao Paulo|Loricariidae|
Hypostomus ancistroides|[85]|Brazil.Sao Paulo|Loricariidae|
Hypostomus ancistroides|[86]|Brazil.Sao Paulo|Loricariidae|
Hypostomus sp.|[87]|French Guiana.St-Laurent-du-Maroni|Loricariidae|
Hypostomus cochliodon|[88]|Brazil.Mato Grosso|Loricariidae|
Hypostomus cochliodon|[89]|Brazil.Mato Grosso|Loricariidae|
Hypostomus sp.|[90]|Argentina.Formosa|Loricariidae|
Hypostomus latifrons|[91]|Brazil.Mato Grosso do Sul|Loricariidae|
Hypostomus latifrons|[92]|Brazil.Mato Grosso do Sul|Loricariidae|
Hypostomus latifrons|[93]|Brazil.Mato Grosso do Sul|Loricariidae|
Hypostomus cochliodon|[94]|Brazil.Mato Grosso do Sul|Loricariidae|
Hypostomus sp.|[95]|Argentina.Formosa|Loricariidae|
Hypostomus sp.|[96]|Argentina.Formosa|Loricariidae|
Hypostomus sp.|[97]|Argentina.Formosa|Loricariidae|
Hypostomus|[98]|Argentina.Chaco|Loricariidae|
Hypostomus|[99]|Argentina.Chaco|Loricariidae|
Hypostomus|[100]|Argentina.Chaco|Loricariidae|
Hypostomus|[101]|Argentina.Chaco|Loricariidae|
Unknown Specimen|
Hypostomus sp.
Field number: sp12.6
Voucher: MNRJ43635

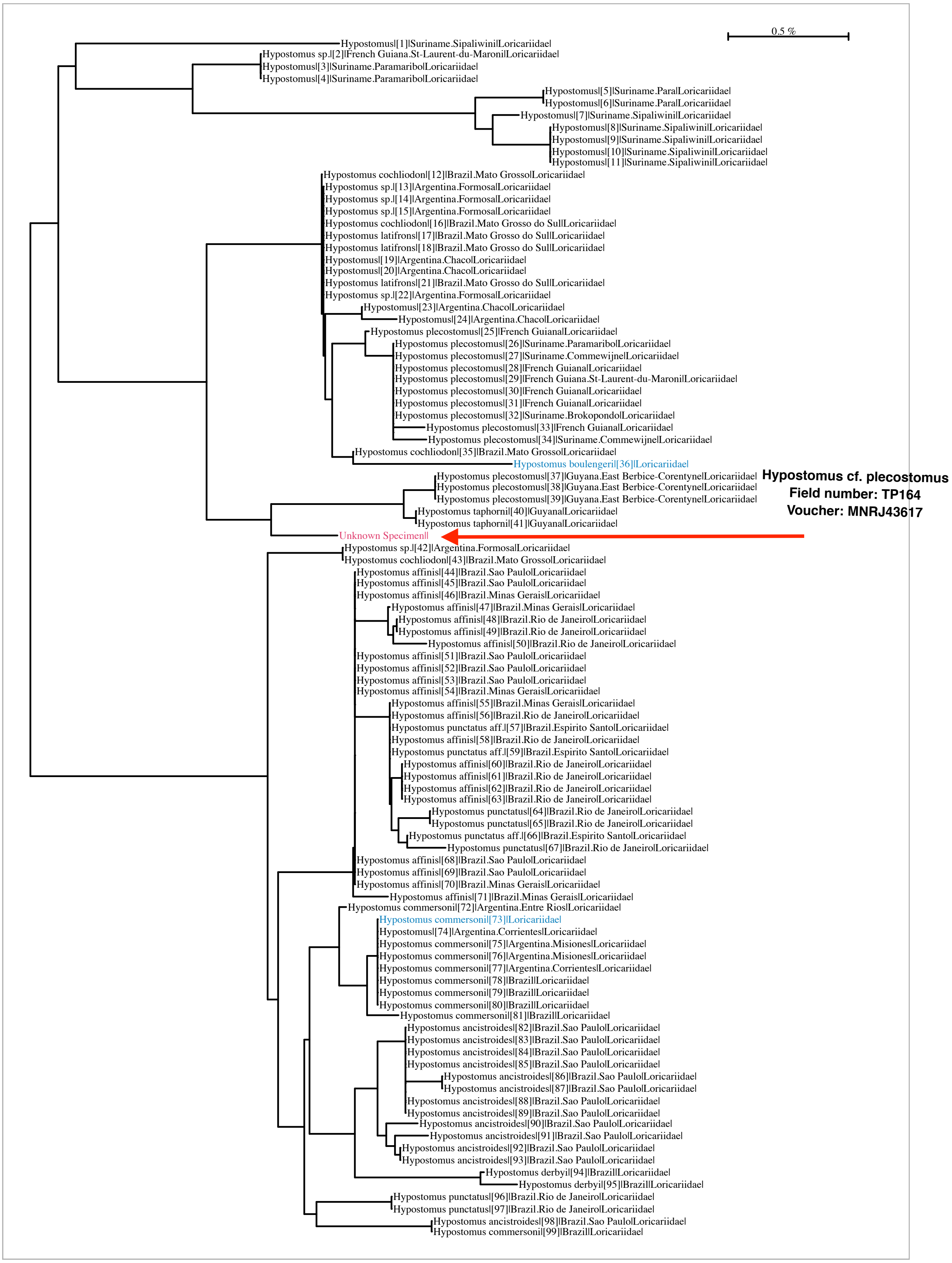
0.5 %
Hypostomus|[1]|Suriname.Sipaliwini|Loricariidae|
Hypostomus sp.|[2]|French Guiana.St-Laurent-du-Maroni|Loricariidae|
Hypostomus|[3]|Suriname.Paramaribo|Loricariidae|
Hypostomus|[4]|Suriname.Paramaribo|Loricariidae|
Hypostomus|[5]|Suriname.Para|Loricariidae|
Hypostomus|[6]|Suriname.Para|Loricariidae|
Hypostomus|[7]|Suriname.Sipaliwini|Loricariidae|
Hypostomus|[8]|Suriname.Sipaliwini|Loricariidae|
Hypostomus|[9]|Suriname.Sipaliwini|Loricariidae|
Hypostomus|[10]|Suriname.Sipaliwini|Loricariidae|
Hypostomus|[11]|Suriname.Sipaliwini|Loricariidae|
Hypostomus cochliodon|[12]|Brazil.Mato Grosso|Loricariidae|
Hypostomus sp.|[13]|Argentina.Formosa|Loricariidae|
Hypostomus sp.|[14]|Argentina.Formosa|Loricariidae|
Hypostomus sp.|[15]|Argentina.Formosa|Loricariidae|
Hypostomus cochliodon|[16]|Brazil.Mato Grosso do Sul|Loricariidae|
Hypostomus latifrons|[17]|Brazil.Mato Grosso do Sul|Loricariidae|
Hypostomus latifrons|[18]|Brazil.Mato Grosso do Sul|Loricariidae|
Hypostomus|[19]|Argentina.Chaco|Loricariidae|
Hypostomus|[20]|Argentina.Chaco|Loricariidae|
Hypostomus latifrons|[21]|Brazil.Mato Grosso do Sul|Loricariidae|
Hypostomus sp.|[22]|Argentina.Formosa|Loricariidae|
Hypostomus|[23]|Argentina.Chaco|Loricariidae|
Hypostomus|[24]|Argentina.Chaco|Loricariidae|
Hypostomus plecostomus|[25]|French Guiana|Loricariidae|
Hypostomus plecostomus|[26]|Suriname.Paramaribo|Loricariidae|
Hypostomus plecostomus|[27]|Suriname.Commewijne|Loricariidae|
Hypostomus plecostomus|[28]|French Guiana|Loricariidae|
Hypostomus plecostomus|[29]|French Guiana.St-Laurent-du-Maroni|Loricariidae|
Hypostomus plecostomus|[30]|French Guiana|Loricariidae|
Hypostomus plecostomus|[31]|French Guiana|Loricariidae|
Hypostomus plecostomus|[32]|Suriname.Brokopondo|Loricariidae|
Hypostomus plecostomus|[33]|French Guiana|Loricariidae|
Hypostomus plecostomus|[34]|Suriname.Commewijne|Loricariidae|
Hypostomus cochliodon|[35]|Brazil.Mato Grosso|Loricariidae|
Hypostomus boulengeri|[36]||Loricariidae|
Hypostomus plecostomus|[37]|Guyana.East Berbice-Corentyne|Loricariidae|
Hypostomus plecostomus|[38]|Guyana.East Berbice-Corentyne|Loricariidae|
Hypostomus plecostomus|[39]|Guyana.East Berbice-Corentyne|Loricariidae|
Hypostomus taphorni|[40]|Guyana|Loricariidae|
Hypostomus taphorni|[41]|Guyana|Loricariidae|
Unknown Specimen||
Hypostomus cf. plecostomus
Field number: TP164
Voucher: MNRJ43617
Hypostomus sp.|[42]|Argentina.Formosa|Loricariidae|
Hypostomus cochliodon|[43]|Brazil.Mato Grosso|Loricariidae|
Hypostomus affinis|[44]|Brazil.Sao Paulo|Loricariidae|
Hypostomus affinis|[45]|Brazil.Sao Paulo|Loricariidae|
Hypostomus affinis|[46]|Brazil.Minas Gerais|Loricariidae|
Hypostomus affinis|[47]|Brazil.Minas Gerais|Loricariidae|
Hypostomus affinis|[48]|Brazil.Rio de Janeiro|Loricariidae|
Hypostomus affinis|[49]|Brazil.Rio de Janeiro|Loricariidae|
Hypostomus affinis|[50]|Brazil.Rio de Janeiro|Loricariidae|
Hypostomus affinis|[51]|Brazil.Sao Paulo|Loricariidae|
Hypostomus affinis|[52]|Brazil.Sao Paulo|Loricariidae|
Hypostomus affinis|[53]|Brazil.Sao Paulo|Loricariidae|
Hypostomus affinis|[54]|Brazil.Minas Gerais|Loricariidae|
Hypostomus affinis|[55]|Brazil.Minas Gerais|Loricariidae|
Hypostomus affinis|[56]|Brazil.Rio de Janeiro|Loricariidae|
Hypostomus punctatus aff.|[57]|Brazil.Espirito Santo|Loricariidae|
Hypostomus affinis|[58]|Brazil.Rio de Janeiro|Loricariidae|
Hypostomus punctatus aff.|[59]|Brazil.Espirito Santo|Loricariidae|
Hypostomus affinis|[60]|Brazil.Rio de Janeiro|Loricariidae|
Hypostomus affinis|[61]|Brazil.Rio de Janeiro|Loricariidae|
Hypostomus affinis|[62]|Brazil.Rio de Janeiro|Loricariidae|
Hypostomus affinis|[63]|Brazil.Rio de Janeiro|Loricariidae|
Hypostomus punctatus|[64]|Brazil.Rio de Janeiro|Loricariidae|
Hypostomus punctatus|[65]|Brazil.Rio de Janeiro|Loricariidae|
Hypostomus punctatus aff.|[66]|Brazil.Espirito Santo|Loricariidae|
Hypostomus punctatus|[67]|Brazil.Rio de Janeiro|Loricariidae|
Hypostomus affinis|[68]|Brazil.Sao Paulo|Loricariidae|
Hypostomus affinis|[69]|Brazil.Sao Paulo|Loricariidae|
Hypostomus affinis|[70]|Brazil.Minas Gerais|Loricariidae|
Hypostomus affinis|[71]|Brazil.Minas Gerais|Loricariidae|
Hypostomus commersoni|[72]|Argentina.Entre Rios|Loricariidae|
Hypostomus commersoni|[73]||Loricariidae|
Hypostomus|[74]|Argentina.Corrientes|Loricariidae|
Hypostomus commersoni|[75]|Argentina.Misiones|Loricariidae|
Hypostomus commersoni|[76]|Argentina.Misiones|Loricariidae|
Hypostomus commersoni|[77]|Argentina.Corrientes|Loricariidae|
Hypostomus commersoni|[78]|Brazil|Loricariidae|
Hypostomus commersoni|[79]|Brazil|Loricariidae|
Hypostomus commersoni|[80]|Brazil|Loricariidae|
Hypostomus commersoni|[81]|Brazil|Loricariidae|
Hypostomus ancistroides|[82]|Brazil.Sao Paulo|Loricariidae|
Hypostomus ancistroides|[83]|Brazil.Sao Paulo|Loricariidae|
Hypostomus ancistroides|[84]|Brazil.Sao Paulo|Loricariidae|
Hypostomus ancistroides|[85]|Brazil.Sao Paulo|Loricariidae|
Hypostomus ancistroides|[86]|Brazil.Sao Paulo|Loricariidae|
Hypostomus ancistroides|[87]|Brazil.Sao Paulo|Loricariidae|
Hypostomus ancistroides|[88]|Brazil.Sao Paulo|Loricariidae|
Hypostomus ancistroides|[89]|Brazil.Sao Paulo|Loricariidae|
Hypostomus ancistroides|[90]|Brazil.Sao Paulo|Loricariidae|
Hypostomus ancistroides|[91]|Brazil.Sao Paulo|Loricariidae|
Hypostomus ancistroides|[92]|Brazil.Sao Paulo|Loricariidae|
Hypostomus ancistroides|[93]|Brazil.Sao Paulo|Loricariidae|
Hypostomus derbyi|[94]|Brazil|Loricariidae|
Hypostomus derbyi|[95]|Brazil|Loricariidae|
Hypostomus punctatus|[96]|Brazil.Rio de Janeiro|Loricariidae|
Hypostomus punctatus|[97]|Brazil.Rio de Janeiro|Loricariidae|
Hypostomus ancistroides|[98]|Brazil.Sao Paulo|Loricariidae|
Hypostomus commersoni|[99]|Brazil|Loricariidae|

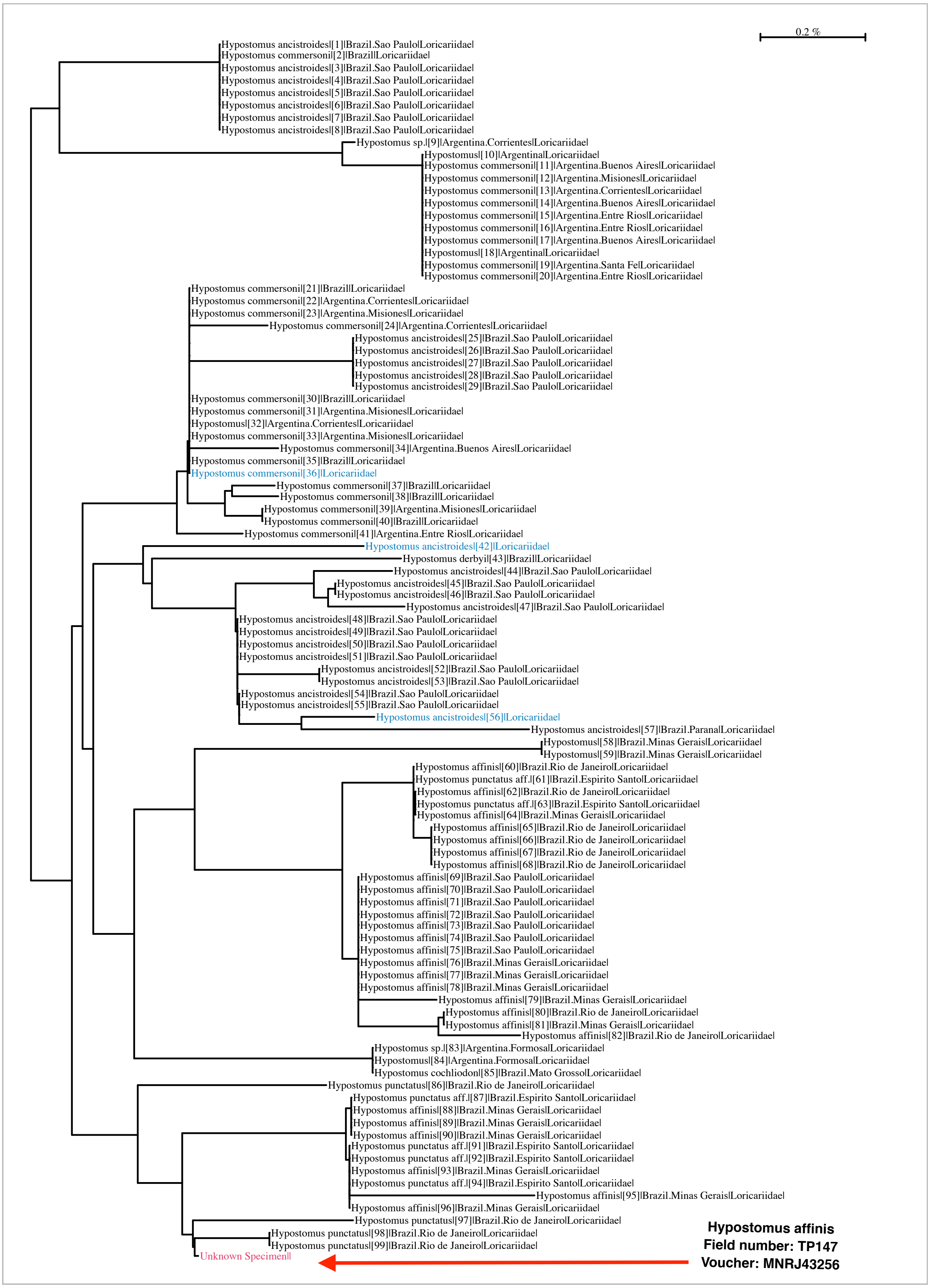
0.2 %
Hypostomus ancistroides|[1]|Brazil.Sao Paulo|Loricariidae|
Hypostomus commersoni|[2]|Brazil|Loricariidae|
Hypostomus ancistroides|[3]|Brazil.Sao Paulo|Loricariidae|
Hypostomus ancistroides|[4]|Brazil.Sao Paulo|Loricariidae|
Hypostomus ancistroides|[5]|Brazil.Sao Paulo|Loricariidae|
Hypostomus ancistroides|[6]|Brazil.Sao Paulo|Loricariidae|
Hypostomus ancistroides|[7]|Brazil.Sao Paulo|Loricariidae|
Hypostomus ancistroides|[8]|Brazil.Sao Paulo|Loricariidae|
Hypostomus sp.|[9]|Argentina.Corrientes|Loricariidae|
Hypostomus|[10]|Argentina|Loricariidae|
Hypostomus commersoni|[11]|Argentina.Buenos Aires|Loricariidae|
Hypostomus commersoni|[12]|Argentina.Misiones|Loricariidae|
Hypostomus commersoni|[13]|Argentina.Corrientes|Loricariidae|
Hypostomus commersoni|[14]|Argentina.Buenos Aires|Loricariidae|
Hypostomus commersoni|[15]|Argentina.Entre Rios|Loricariidae|
Hypostomus commersoni|[16]|Argentina.Entre Rios|Loricariidae|
Hypostomus commersoni|[17]|Argentina.Buenos Aires|Loricariidae|
Hypostomus|[18]|Argentina|Loricariidae|
Hypostomus commersoni|[19]|Argentina.Santa Fe|Loricariidae|
Hypostomus commersoni|[20]|Argentina.Entre Rios|Loricariidae|
Hypostomus commersoni|[21]|Brazil|Loricariidae|
Hypostomus commersoni|[22]|Argentina.Corrientes|Loricariidae|
Hypostomus commersoni|[23]|Argentina.Misiones|Loricariidae|
Hypostomus commersoni|[24]|Argentina.Corrientes|Loricariidae|
Hypostomus ancistroides|[25]|Brazil.Sao Paulo|Loricariidae|
Hypostomus ancistroides|[26]|Brazil.Sao Paulo|Loricariidae|
Hypostomus ancistroides|[27]|Brazil.Sao Paulo|Loricariidae|
Hypostomus ancistroides|[28]|Brazil.Sao Paulo|Loricariidae|
Hypostomus ancistroides|[29]|Brazil.Sao Paulo|Loricariidae|
Hypostomus commersoni|[30]|Brazil|Loricariidae|
Hypostomus commersoni|[31]|Argentina.Misiones|Loricariidae|
Hypostomus|[32]|Argentina.Corrientes|Loricariidae|
Hypostomus commersoni|[33]|Argentina.Misiones|Loricariidae|
Hypostomus commersoni|[34]|Argentina.Buenos Aires|Loricariidae|
Hypostomus commersoni|[35]|Brazil|Loricariidae|
Hypostomus commersoni|[36]||Loricariidae|
Hypostomus commersoni|[37]|Brazil|Loricariidae|
Hypostomus commersoni|[38]|Brazil|Loricariidae|
Hypostomus commersoni|[39]|Argentina.Misiones|Loricariidae|
Hypostomus commersoni|[40]|Brazil|Loricariidae|
Hypostomus commersoni|[41]|Argentina.Entre Rios|Loricariidae|
Hypostomus ancistroides|[42]||Loricariidae|
Hypostomus derbyi|[43]|Brazil|Loricariidae|
Hypostomus ancistroides|[44]|Brazil.Sao Paulo|Loricariidae|
Hypostomus ancistroides|[45]|Brazil.Sao Paulo|Loricariidae|
Hypostomus ancistroides|[46]|Brazil.Sao Paulo|Loricariidae|
Hypostomus ancistroides|[47]|Brazil.Sao Paulo|Loricariidae|
Hypostomus ancistroides|[48]|Brazil.Sao Paulo|Loricariidae|
Hypostomus ancistroides|[49]|Brazil.Sao Paulo|Loricariidae|
Hypostomus ancistroides|[50]|Brazil.Sao Paulo|Loricariidae|
Hypostomus ancistroides|[51]|Brazil.Sao Paulo|Loricariidae|
Hypostomus ancistroides|[52]|Brazil.Sao Paulo|Loricariidae|
Hypostomus ancistroides|[53]|Brazil.Sao Paulo|Loricariidae|
Hypostomus ancistroides|[54]|Brazil.Sao Paulo|Loricariidae|
Hypostomus ancistroides|[55]|Brazil.Sao Paulo|Loricariidae|
Hypostomus ancistroides|[56]||Loricariidae|
Hypostomus ancistroides|[57]|Brazil.Parana|Loricariidae|
Hypostomus|[58]|Brazil.Minas Gerais|Loricariidae|
Hypostomus|[59]|Brazil.Minas Gerais|Loricariidae|
Hypostomus affinis|[60]|Brazil.Rio de Janeiro|Loricariidae|
Hypostomus punctatus aff.|[61]|Brazil.Espirito Santo|Loricariidae|
Hypostomus affinis|[62]|Brazil.Rio de Janeiro|Loricariidae|
Hypostomus punctatus aff.|[63]|Brazil.Espirito Santo|Loricariidae|
Hypostomus affinis|[64]|Brazil.Minas Gerais|Loricariidae|
Hypostomus affinis|[65]|Brazil.Rio de Janeiro|Loricariidae|
Hypostomus affinis|[66]|Brazil.Rio de Janeiro|Loricariidae|
Hypostomus affinis|[67]|Brazil.Rio de Janeiro|Loricariidae|
Hypostomus affinis|[68]|Brazil.Rio de Janeiro|Loricariidae|
Hypostomus affinis|[69]|Brazil.Sao Paulo|Loricariidae|
Hypostomus affinis|[70]|Brazil.Sao Paulo|Loricariidae|
Hypostomus affinis|[71]|Brazil.Sao Paulo|Loricariidae|
Hypostomus affinis|[72]|Brazil.Sao Paulo|Loricariidae|
Hypostomus affinis|[73]|Brazil.Sao Paulo|Loricariidae|
Hypostomus affinis|[74]|Brazil.Sao Paulo|Loricariidae|
Hypostomus affinis|[75]|Brazil.Sao Paulo|Loricariidae|
Hypostomus affinis|[76]|Brazil.Minas Gerais|Loricariidae|
Hypostomus affinis|[77]|Brazil.Minas Gerais|Loricariidae|
Hypostomus affinis|[78]|Brazil.Minas Gerais|Loricariidae|
Hypostomus affinis|[79]|Brazil.Minas Gerais|Loricariidae|
Hypostomus affinis|[80]|Brazil.Rio de Janeiro|Loricariidae|
Hypostomus affinis|[81]|Brazil.Minas Gerais|Loricariidae|
Hypostomus affinis|[82]|Brazil.Rio de Janeiro|Loricariidae|
Hypostomus sp.|[83]|Argentina.Formosa|Loricariidae|
Hypostomus|[84]|Argentina.Formosa|Loricariidae|
Hypostomus cochliodon|[85]|Brazil.Mato Grosso|Loricariidae|
Hypostomus punctatus|[86]|Brazil.Rio de Janeiro|Loricariidae|
Hypostomus punctatus aff.|[87]|Brazil.Espirito Santo|Loricariidae|
Hypostomus affinis|[88]|Brazil.Minas Gerais|Loricariidae|
Hypostomus affinis|[89]|Brazil.Minas Gerais|Loricariidae|
Hypostomus affinis|[90]|Brazil.Minas Gerais|Loricariidae|
Hypostomus punctatus aff.|[91]|Brazil.Espirito Santo|Loricariidae|
Hypostomus punctatus aff.|[92]|Brazil.Espirito Santo|Loricariidae|
Hypostomus affinis|[93]|Brazil.Minas Gerais|Loricariidae|
Hypostomus punctatus aff.|[94]|Brazil.Espirito Santo|Loricariidae|
Hypostomus affinis|[95]|Brazil.Minas Gerais|Loricariidae|
Hypostomus affinis|[96]|Brazil.Minas Gerais|Loricariidae|
Hypostomus punctatus|[97]|Brazil.Rio de Janeiro|Loricariidae|
Hypostomus punctatus|[98]|Brazil.Rio de Janeiro|Loricariidae|
Hypostomus punctatus|[99]|Brazil.Rio de Janeiro|Loricariidae|
Unknown Specimen||
Hypostomus affinis
Field number: TP147
Voucher: MNRJ43256

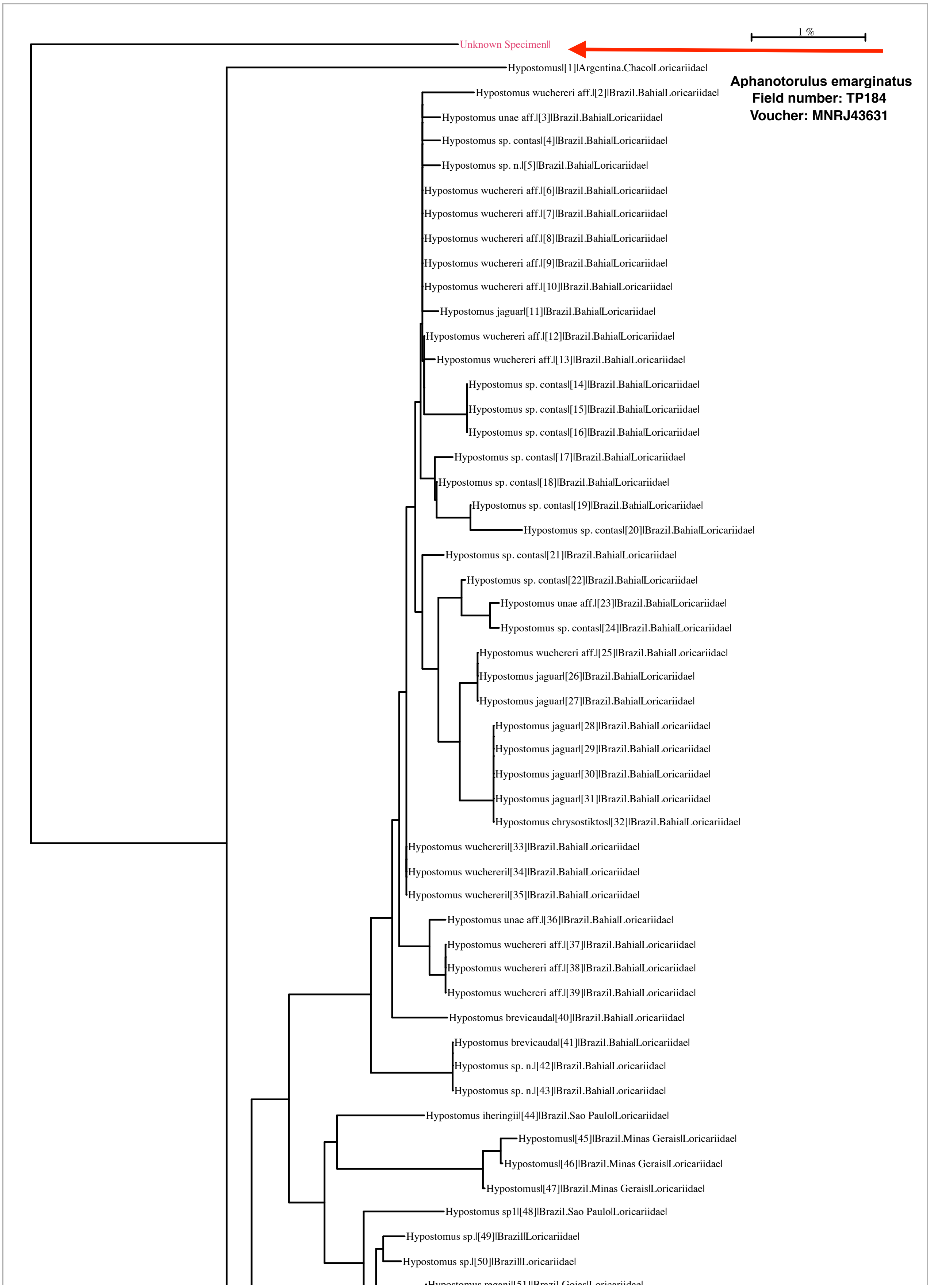
1 %
Unknown Specimen||
Aphanotorulus emarginatus
Field number: TP184
Voucher: MNRJ43631
Hypostomus|[1]|Argentina.Chaco|Loricariidae|
Hypostomus wuchereri aff.|[2]|Brazil.Bahia|Loricariidae|
Hypostomus unae aff.|[3]|Brazil.Bahia|Loricariidae|
Hypostomus sp. contas|[4]|Brazil.Bahia|Loricariidae|
Hypostomus sp. n.|[5]|Brazil.Bahia|Loricariidae|
Hypostomus wuchereri aff.|[6]|Brazil.Bahia|Loricariidae|
Hypostomus wuchereri aff.|[7]|Brazil.Bahia|Loricariidae|
Hypostomus wuchereri aff.|[8]|Brazil.Bahia|Loricariidae|
Hypostomus wuchereri aff.|[9]|Brazil.Bahia|Loricariidae|
Hypostomus wuchereri aff.|[10]|Brazil.Bahia|Loricariidae|
Hypostomus jaguar|[11]|Brazil.Bahia|Loricariidae|
Hypostomus wuchereri aff.|[12]|Brazil.Bahia|Loricariidae|
Hypostomus wuchereri aff.|[13]|Brazil.Bahia|Loricariidae|
Hypostomus sp. contas|[14]|Brazil.Bahia|Loricariidae|
Hypostomus sp. contas|[15]|Brazil.Bahia|Loricariidae|
Hypostomus sp. contas|[16]|Brazil.Bahia|Loricariidae|
Hypostomus sp. contas|[17]|Brazil.Bahia|Loricariidae|
Hypostomus sp. contas|[18]|Brazil.Bahia|Loricariidae|
Hypostomus sp. contas|[19]|Brazil.Bahia|Loricariidae|
Hypostomus sp. contas|[20]|Brazil.Bahia|Loricariidae|
Hypostomus sp. contas|[21]|Brazil.Bahia|Loricariidae|
Hypostomus sp. contas|[22]|Brazil.Bahia|Loricariidae|
Hypostomus unae aff.|[23]|Brazil.Bahia|Loricariidae|
Hypostomus sp. contas|[24]|Brazil.Bahia|Loricariidae|
Hypostomus wuchereri aff.|[25]|Brazil.Bahia|Loricariidae|
Hypostomus jaguar|[26]|Brazil.Bahia|Loricariidae|
Hypostomus jaguar|[27]|Brazil.Bahia|Loricariidae|
Hypostomus jaguar|[28]|Brazil.Bahia|Loricariidae|
Hypostomus jaguar|[29]|Brazil.Bahia|Loricariidae|
Hypostomus jaguar|[30]|Brazil.Bahia|Loricariidae|
Hypostomus jaguar|[31]|Brazil.Bahia|Loricariidae|
Hypostomus chrysostiktos|[32]|Brazil.Bahia|Loricariidae|
Hypostomus wuchereri|[33]|Brazil.Bahia|Loricariidae|
Hypostomus wuchereri|[34]|Brazil.Bahia|Loricariidae|
Hypostomus wuchereri|[35]|Brazil.Bahia|Loricariidae|
Hypostomus unae aff.|[36]|Brazil.Bahia|Loricariidae|
Hypostomus wuchereri aff.|[37]|Brazil.Bahia|Loricariidae|
Hypostomus wuchereri aff.|[38]|Brazil.Bahia|Loricariidae|
Hypostomus wuchereri aff.|[39]|Brazil.Bahia|Loricariidae|
Hypostomus brevicauda|[40]|Brazil.Bahia|Loricariidae|
Hypostomus brevicauda|[41]|Brazil.Bahia|Loricariidae|
Hypostomus sp. n.|[42]|Brazil.Bahia|Loricariidae|
Hypostomus sp. n.|[43]|Brazil.Bahia|Loricariidae|
Hypostomus iheringii|[44]|Brazil.Sao Paulo|Loricariidae|
Hypostomus|[45]|Brazil.Minas Gerais|Loricariidae|
Hypostomus|[46]|Brazil.Minas Gerais|Loricariidae|
Hypostomus|[47]|Brazil.Minas Gerais|Loricariidae|
Hypostomus sp1|[48]|Brazil.Sao Paulo|Loricariidae|
Hypostomus sp.|[49]|Brazil|Loricariidae|
Hypostomus sp.|[50]|Brazil|Loricariidae|

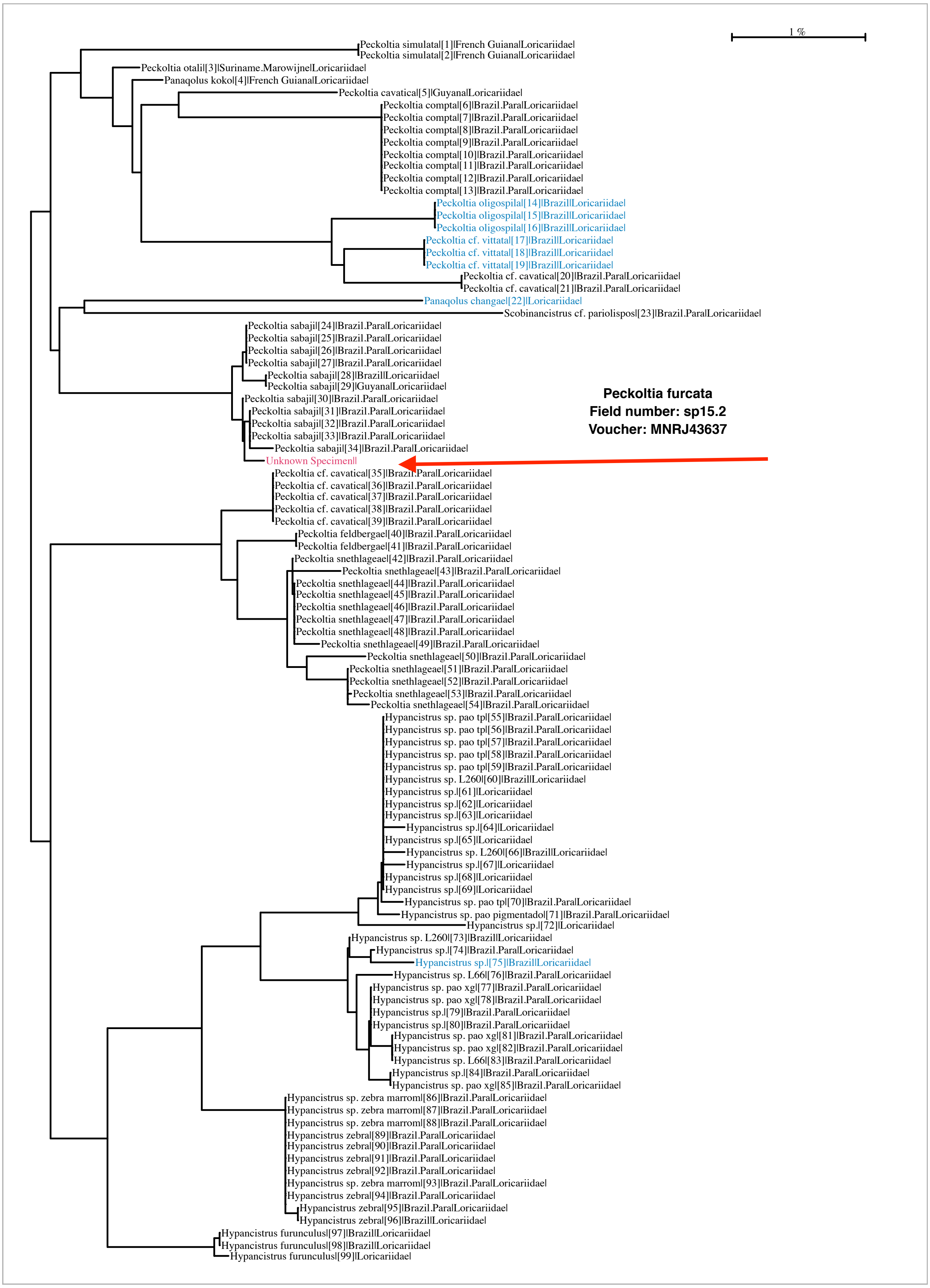
1 %
Peckoltia simulata|[1]|French Guiana|Loricariidae|
Peckoltia simulata|[2]|French Guiana|Loricariidae|
Peckoltia otali|[3]|Suriname.Marowijne|Loricariidae|
Panaqolus koko|[4]|French Guiana|Loricariidae|
Peckoltia cavatica|[5]|Guyana|Loricariidae|
Peckoltia compta|[6]|Brazil.Para|Loricariidae|
Peckoltia compta|[7]|Brazil.Para|Loricariidae|
Peckoltia compta|[8]|Brazil.Para|Loricariidae|
Peckoltia compta|[9]|Brazil.Para|Loricariidae|
Peckoltia compta|[10]|Brazil.Para|Loricariidae|
Peckoltia compta|[11]|Brazil.Para|Loricariidae|
Peckoltia compta|[12]|Brazil.Para|Loricariidae|
Peckoltia compta|[13]|Brazil.Para|Loricariidae|
Peckoltia oligospila|[14]|Brazil|Loricariidae|
Peckoltia oligospila|[15]|Brazil|Loricariidae|
Peckoltia oligospila|[16]|Brazil|Loricariidae|
Peckoltia cf. vittata|[17]|Brazil|Loricariidae|
Peckoltia cf. vittata|[18]|Brazil|Loricariidae|
Peckoltia cf. vittata|[19]|Brazil|Loricariidae|
Peckoltia cf. cavatica|[20]|Brazil.Para|Loricariidae|
Peckoltia cf. cavatica|[21]|Brazil.Para|Loricariidae|
Panaqolus changae|[22]||Loricariidae|
Scobinancistrus cf. pariolispos|[23]|Brazil.Para|Loricariidae|
Peckoltia sabaji|[24]|Brazil.Para|Loricariidae|
Peckoltia sabaji|[25]|Brazil.Para|Loricariidae|
Peckoltia sabaji|[26]|Brazil.Para|Loricariidae|
Peckoltia sabaji|[27]|Brazil.Para|Loricariidae|
Peckoltia sabaji|[28]|Brazil|Loricariidae|
Peckoltia sabaji|[29]|Guyana|Loricariidae|
Peckoltia sabaji|[30]|Brazil.Para|Loricariidae|
Peckoltia sabaji|[31]|Brazil.Para|Loricariidae|
Peckoltia sabaji|[32]|Brazil.Para|Loricariidae|
Peckoltia sabaji|[33]|Brazil.Para|Loricariidae|
Peckoltia sabaji|[34]|Brazil.Para|Loricariidae|
Unknown Specimen||
Peckoltia furcata
Field number: sp15.2
Voucher: MNRJ43637
Peckoltia cf. cavatica|[35]|Brazil.Para|Loricariidae|
Peckoltia cf. cavatica|[36]|Brazil.Para|Loricariidae|
Peckoltia cf. cavatica|[37]|Brazil.Para|Loricariidae|
Peckoltia cf. cavatica|[38]|Brazil.Para|Loricariidae|
Peckoltia cf. cavatica|[39]|Brazil.Para|Loricariidae|
Peckoltia feldbergae|[40]|Brazil.Para|Loricariidae|
Peckoltia feldbergae|[41]|Brazil.Para|Loricariidae|
Peckoltia snethlageae|[42]|Brazil.Para|Loricariidae|
Peckoltia snethlageae|[43]|Brazil.Para|Loricariidae|
Peckoltia snethlageae|[44]|Brazil.Para|Loricariidae|
Peckoltia snethlageae|[45]|Brazil.Para|Loricariidae|
Peckoltia snethlageae|[46]|Brazil.Para|Loricariidae|
Peckoltia snethlageae|[47]|Brazil.Para|Loricariidae|
Peckoltia snethlageae|[48]|Brazil.Para|Loricariidae|
Peckoltia snethlageae|[49]|Brazil.Para|Loricariidae|
Peckoltia snethlageae|[50]|Brazil.Para|Loricariidae|
Peckoltia snethlageae|[51]|Brazil.Para|Loricariidae|
Peckoltia snethlageae|[52]|Brazil.Para|Loricariidae|
Peckoltia snethlageae|[53]|Brazil.Para|Loricariidae|
Peckoltia snethlageae|[54]|Brazil.Para|Loricariidae|
Hypancistrus sp. pao tp|[55]|Brazil.Para|Loricariidae|
Hypancistrus sp. pao tp|[56]|Brazil.Para|Loricariidae|
Hypancistrus sp. pao tp|[57]|Brazil.Para|Loricariidae|
Hypancistrus sp. pao tp|[58]|Brazil.Para|Loricariidae|
Hypancistrus sp. pao tp|[59]|Brazil.Para|Loricariidae|
Hypancistrus sp. L260|[60]|Brazil|Loricariidae|
Hypancistrus sp.|[61]||Loricariidae|
Hypancistrus sp.|[62]||Loricariidae|
Hypancistrus sp.|[63]||Loricariidae|
Hypancistrus sp.|[64]||Loricariidae|
Hypancistrus sp.|[65]||Loricariidae|
Hypancistrus sp. L260|[66]|Brazil|Loricariidae|
Hypancistrus sp.|[67]||Loricariidae|
Hypancistrus sp.|[68]||Loricariidae|
Hypancistrus sp.|[69]||Loricariidae|
Hypancistrus sp. pao tp|[70]|Brazil.Para|Loricariidae|
Hypancistrus sp. pao pigmentado|[71]|Brazil.Para|Loricariidae|
Hypancistrus sp.|[72]||Loricariidae|
Hypancistrus sp. L260|[73]|Brazil|Loricariidae|
Hypancistrus sp.|[74]|Brazil.Para|Loricariidae|
Hypancistrus sp.|[75]|Brazil|Loricariidae|
Hypancistrus sp. L66|[76]|Brazil.Para|Loricariidae|
Hypancistrus sp. pao xg|[77]|Brazil.Para|Loricariidae|
Hypancistrus sp. pao xg|[78]|Brazil.Para|Loricariidae|
Hypancistrus sp.|[79]|Brazil.Para|Loricariidae|
Hypancistrus sp.|[80]|Brazil.Para|Loricariidae|
Hypancistrus sp. pao xg|[81]|Brazil.Para|Loricariidae|
Hypancistrus sp. pao xg|[82]|Brazil.Para|Loricariidae|
Hypancistrus sp. L66|[83]|Brazil.Para|Loricariidae|
Hypancistrus sp.|[84]|Brazil.Para|Loricariidae|
Hypancistrus sp. pao xg|[85]|Brazil.Para|Loricariidae|
Hypancistrus sp. zebra marrom|[86]|Brazil.Para|Loricariidae|
Hypancistrus sp. zebra marrom|[87]|Brazil.Para|Loricariidae|
Hypancistrus sp. zebra marrom|[88]|Brazil.Para|Loricariidae|
Hypancistrus zebra|[89]|Brazil.Para|Loricariidae|
Hypancistrus zebra|[90]|Brazil.Para|Loricariidae|
Hypancistrus zebra|[91]|Brazil.Para|Loricariidae|
Hypancistrus zebra|[92]|Brazil.Para|Loricariidae|
Hypancistrus sp. zebra marrom|[93]|Brazil.Para|Loricariidae|
Hypancistrus zebra|[94]|Brazil.Para|Loricariidae|
Hypancistrus zebra|[95]|Brazil.Para|Loricariidae|
Hypancistrus zebra|[96]|Brazil|Loricariidae|
Hypancistrus furunculus|[97]|Brazil|Loricariidae|
Hypancistrus furunculus|[98]|Brazil|Loricariidae|
Hypancistrus furunculus|[99]||Loricariidae|

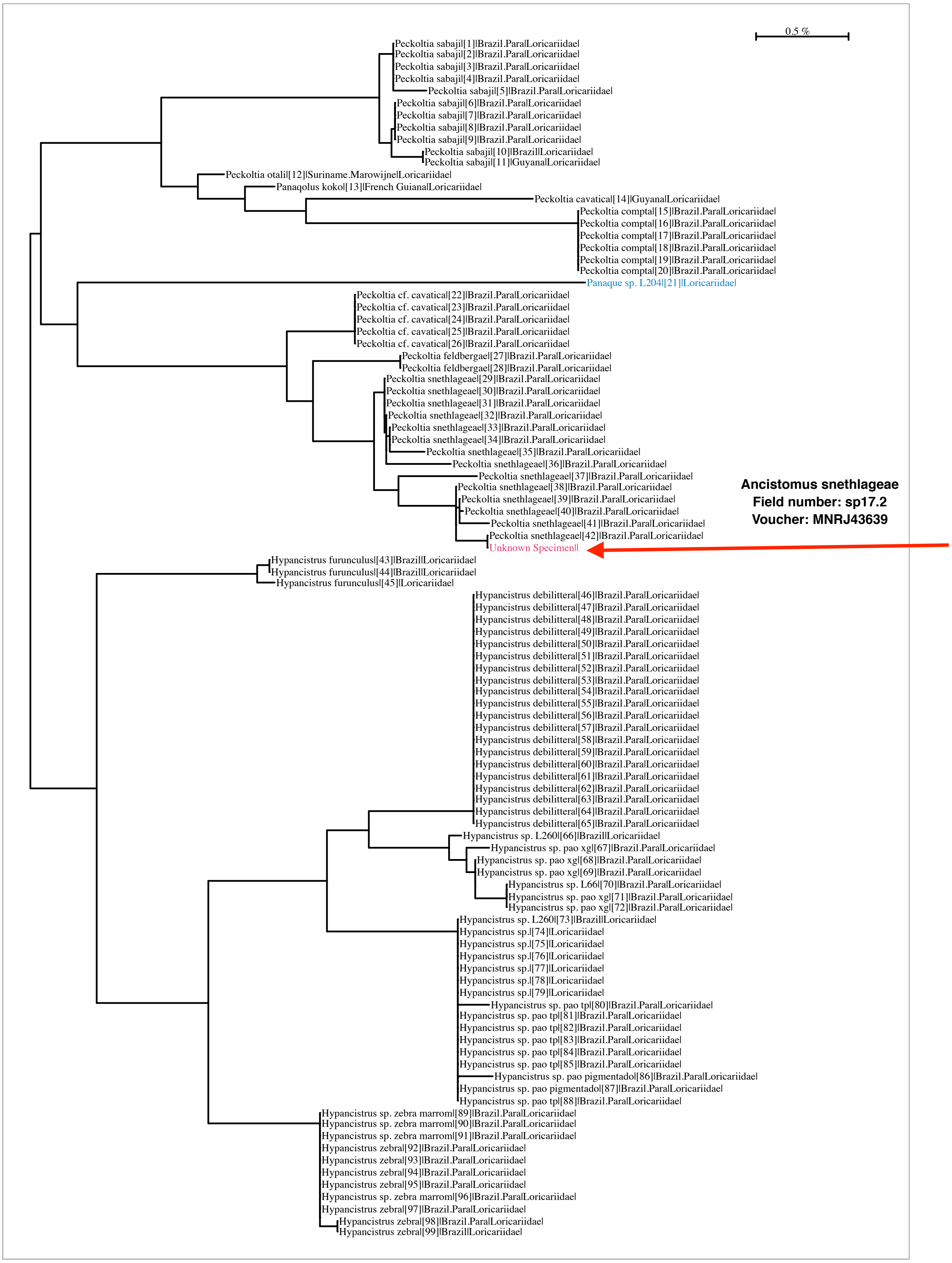
0.5 %
Peckoltia sabaji|[1]|Brazil.Para|Loricariidae|
Peckoltia sabaji|[2]|Brazil.Para|Loricariidae|
Peckoltia sabaji|[3]|Brazil.Para|Loricariidae|
Peckoltia sabaji|[4]|Brazil.Para|Loricariidae|
Peckoltia sabaji|[5]|Brazil.Para|Loricariidae|
Peckoltia sabaji|[6]|Brazil.Para|Loricariidae|
Peckoltia sabaji|[7]|Brazil.Para|Loricariidae|
Peckoltia sabaji|[8]|Brazil.Para|Loricariidae|
Peckoltia sabaji|[9]|Brazil.Para|Loricariidae|
Peckoltia sabaji|[10]|Brazil||Loricariidae|
Peckoltia sabaji|[11]|Guyana|Loricariidae|
Peckoltia otali|[12]|Suriname.Marowijne|Loricariidae|
Panaqolus koko|[13]|French Guiana|Loricariidae|
Peckoltia cavatica|[14]|Guyana|Loricariidae|
Peckoltia compta|[15]|Brazil.Para|Loricariidae|
Peckoltia compta|[16]|Brazil.Para|Loricariidae|
Peckoltia compta|[17]|Brazil.Para|Loricariidae|
Peckoltia compta|[18]|Brazil.Para|Loricariidae|
Peckoltia compta|[19]|Brazil.Para|Loricariidae|
Peckoltia compta|[20]|Brazil.Para|Loricariidae|
Panaque sp. L204|[21]||Loricariidae|
Peckoltia cf. cavatica|[22]|Brazil.Para|Loricariidae|
Peckoltia cf. cavatica|[23]|Brazil.Para|Loricariidae|
Peckoltia cf. cavatica|[24]|Brazil.Para|Loricariidae|
Peckoltia cf. cavatica|[25]|Brazil.Para|Loricariidae|
Peckoltia cf. cavatica|[26]|Brazil.Para|Loricariidae|
Peckoltia feldbergae|[27]|Brazil.Para|Loricariidae|
Peckoltia feldbergae|[28]|Brazil.Para|Loricariidae|
Peckoltia snethlageae|[29]|Brazil.Para|Loricariidae|
Peckoltia snethlageae|[30]|Brazil.Para|Loricariidae|
Peckoltia snethlageae|[31]|Brazil.Para|Loricariidae|
Peckoltia snethlageae|[32]|Brazil.Para|Loricariidae|
Peckoltia snethlageae|[33]|Brazil.Para|Loricariidae|
Peckoltia snethlageae|[34]|Brazil.Para|Loricariidae|
Peckoltia snethlageae|[35]|Brazil.Para|Loricariidae|
Peckoltia snethlageae|[36]|Brazil.Para|Loricariidae|
Peckoltia snethlageae|[37]|Brazil.Para|Loricariidae|
Peckoltia snethlageae|[38]|Brazil.Para|Loricariidae|
Peckoltia snethlageae|[39]|Brazil.Para|Loricariidae|
Peckoltia snethlageae|[40]|Brazil.Para|Loricariidae|
Peckoltia snethlageae|[41]|Brazil.Para|Loricariidae|
Peckoltia snethlageae|[42]|Brazil.Para|Loricariidae|
Unknown Specimen||
Ancistomus snethlageae
Field number: sp17.2
Voucher: MNRJ43639
Hypancistrus furunculus|[43]|Brazil||Loricariidae|
Hypancistrus furunculus|[44]|Brazil||Loricariidae|
Hypancistrus furunculus|[45]||Loricariidae|
Hypancistrus debilittera|[46]|Brazil.Para|Loricariidae|
Hypancistrus debilittera|[47]|Brazil.Para|Loricariidae|
Hypancistrus debilittera|[48]|Brazil.Para|Loricariidae|
Hypancistrus debilittera|[49]|Brazil.Para|Loricariidae|
Hypancistrus debilittera|[50]|Brazil.Para|Loricariidae|
Hypancistrus debilittera|[51]|Brazil.Para|Loricariidae|
Hypancistrus debilittera|[52]|Brazil.Para|Loricariidae|
Hypancistrus debilittera|[53]|Brazil.Para|Loricariidae|
Hypancistrus debilittera|[54]|Brazil.Para|Loricariidae|
Hypancistrus debilittera|[55]|Brazil.Para|Loricariidae|
Hypancistrus debilittera|[56]|Brazil.Para|Loricariidae|
Hypancistrus debilittera|[57]|Brazil.Para|Loricariidae|
Hypancistrus debilittera|[58]|Brazil.Para|Loricariidae|
Hypancistrus debilittera|[59]|Brazil.Para|Loricariidae|
Hypancistrus debilittera|[60]|Brazil.Para|Loricariidae|
Hypancistrus debilittera|[61]|Brazil.Para|Loricariidae|
Hypancistrus debilittera|[62]|Brazil.Para|Loricariidae|
Hypancistrus debilittera|[63]|Brazil.Para|Loricariidae|
Hypancistrus debilittera|[64]|Brazil.Para|Loricariidae|
Hypancistrus debilittera|[65]|Brazil.Para|Loricariidae|
Hypancistrus sp. L260|[66]|Brazil||Loricariidae|
Hypancistrus sp. pao xg|[67]|Brazil.Para|Loricariidae|
Hypancistrus sp. pao xg|[68]|Brazil.Para|Loricariidae|
Hypancistrus sp. pao xg|[69]|Brazil.Para|Loricariidae|
Hypancistrus sp. L66|[70]|Brazil.Para|Loricariidae|
Hypancistrus sp. pao xg|[71]|Brazil.Para|Loricariidae|
Hypancistrus sp. pao xg|[72]|Brazil.Para|Loricariidae|
Hypancistrus sp. L260|[73]|Brazil||Loricariidae|
Hypancistrus sp.|[74]||Loricariidae|
Hypancistrus sp.|[75]||Loricariidae|
Hypancistrus sp.|[76]||Loricariidae|
Hypancistrus sp.|[77]||Loricariidae|
Hypancistrus sp.|[78]||Loricariidae|
Hypancistrus sp.|[79]||Loricariidae|
Hypancistrus sp. pao tp|[80]|Brazil.Para|Loricariidae|
Hypancistrus sp. pao tp|[81]|Brazil.Para|Loricariidae|
Hypancistrus sp. pao tp|[82]|Brazil.Para|Loricariidae|
Hypancistrus sp. pao tp|[83]|Brazil.Para|Loricariidae|
Hypancistrus sp. pao tp|[84]|Brazil.Para|Loricariidae|
Hypancistrus sp. pao tp|[85]|Brazil.Para|Loricariidae|
Hypancistrus sp. pao pigmentado|[86]|Brazil.Para|Loricariidae|
Hypancistrus sp. pao pigmentado|[87]|Brazil.Para|Loricariidae|
Hypancistrus sp. pao tp|[88]|Brazil.Para|Loricariidae|
Hypancistrus sp. zebra marrom|[89]|Brazil.Para|Loricariidae|
Hypancistrus sp. zebra marrom|[90]|Brazil.Para|Loricariidae|
Hypancistrus sp. zebra marrom|[91]|Brazil.Para|Loricariidae|
Hypancistrus zebra|[92]|Brazil.Para|Loricariidae|
Hypancistrus zebra|[93]|Brazil.Para|Loricariidae|
Hypancistrus zebra|[94]|Brazil.Para|Loricariidae|
Hypancistrus zebra|[95]|Brazil.Para|Loricariidae|
Hypancistrus sp. zebra marrom|[96]|Brazil.Para|Loricariidae|
Hypancistrus zebra|[97]|Brazil.Para|Loricariidae|
Hypancistrus zebra|[98]|Brazil.Para|Loricariidae|
Hypancistrus zebra|[99]|Brazil||Loricariidae|

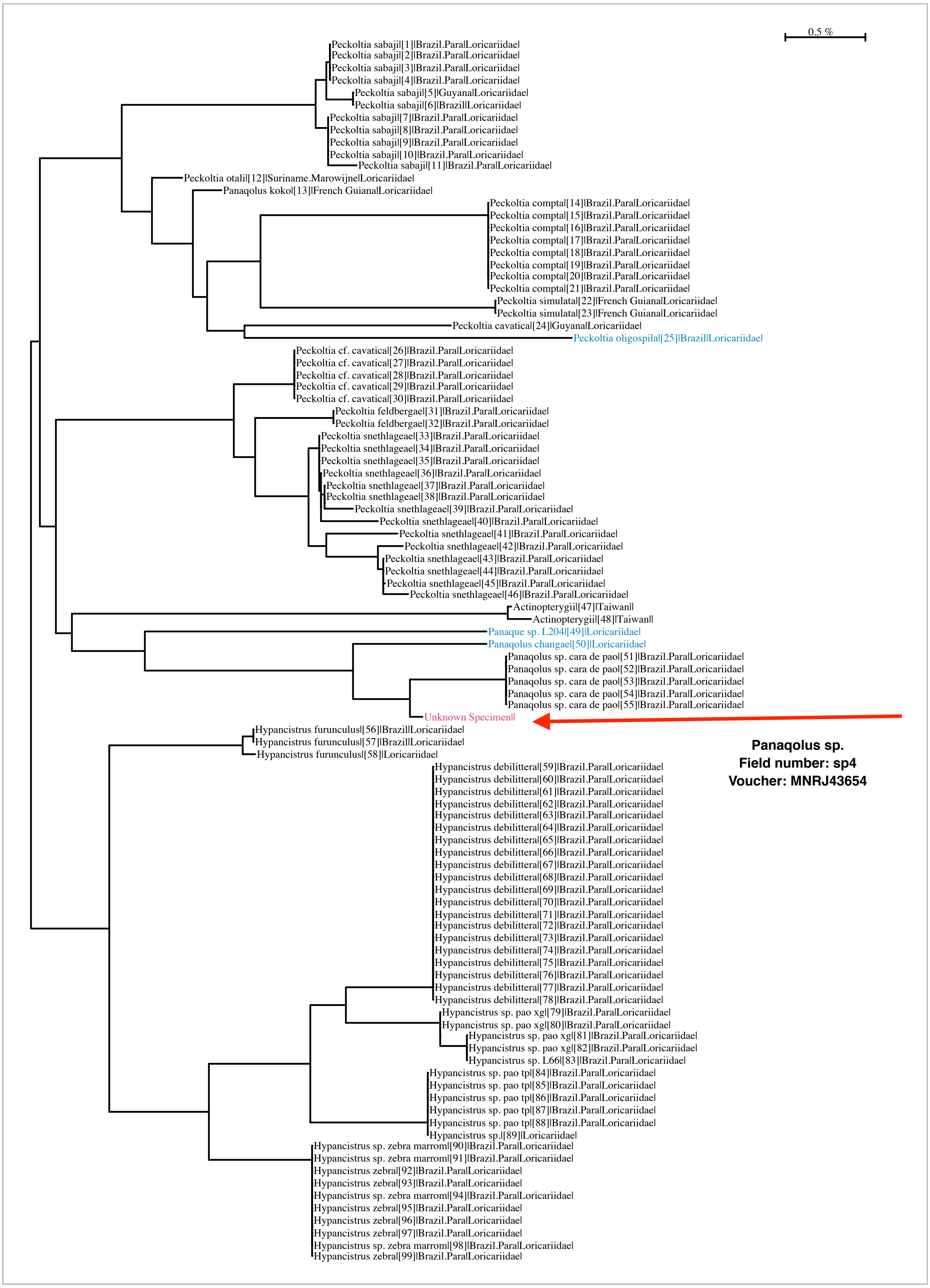
0.5 %
Peckoltia sabaji|[1]|Brazil.Para|Loricariidae|
Peckoltia sabaji|[2]|Brazil.Para|Loricariidae|
Peckoltia sabaji|[3]|Brazil.Para|Loricariidae|
Peckoltia sabaji|[4]|Brazil.Para|Loricariidae|
Peckoltia sabaji|[5]|Guyana|Loricariidae|
Peckoltia sabaji|[6]|Brazil|Loricariidae|
Peckoltia sabaji|[7]|Brazil.Para|Loricariidae|
Peckoltia sabaji|[8]|Brazil.Para|Loricariidae|
Peckoltia sabaji|[9]|Brazil.Para|Loricariidae|
Peckoltia sabaji|[10]|Brazil.Para|Loricariidae|
Peckoltia sabaji|[11]|Brazil.Para|Loricariidae|
Peckoltia otali|[12]|Suriname.Marowijne|Loricariidae|
Panaqolus koko|[13]|French Guiana|Loricariidae|
Peckoltia compta|[14]|Brazil.Para|Loricariidae|
Peckoltia compta|[15]|Brazil.Para|Loricariidae|
Peckoltia compta|[16]|Brazil.Para|Loricariidae|
Peckoltia compta|[17]|Brazil.Para|Loricariidae|
Peckoltia compta|[18]|Brazil.Para|Loricariidae|
Peckoltia compta|[19]|Brazil.Para|Loricariidae|
Peckoltia compta|[20]|Brazil.Para|Loricariidae|
Peckoltia compta|[21]|Brazil.Para|Loricariidae|
Peckoltia simulata|[22]|French Guiana|Loricariidae|
Peckoltia simulata|[23]|French Guiana|Loricariidae|
Peckoltia cavatica|[24]|Guyana|Loricariidae|
Peckoltia oligospila|[25]|Brazil|Loricariidae|
Peckoltia cf. cavatica|[26]|Brazil.Para|Loricariidae|
Peckoltia cf. cavatica|[27]|Brazil.Para|Loricariidae|
Peckoltia cf. cavatica|[28]|Brazil.Para|Loricariidae|
Peckoltia cf. cavatica|[29]|Brazil.Para|Loricariidae|
Peckoltia cf. cavatica|[30]|Brazil.Para|Loricariidae|
Peckoltia feldbergae|[31]|Brazil.Para|Loricariidae|
Peckoltia feldbergae|[32]|Brazil.Para|Loricariidae|
Peckoltia snethlageae|[33]|Brazil.Para|Loricariidae|
Peckoltia snethlageae|[34]|Brazil.Para|Loricariidae|
Peckoltia snethlageae|[35]|Brazil.Para|Loricariidae|
Peckoltia snethlageae|[36]|Brazil.Para|Loricariidae|
Peckoltia snethlageae|[37]|Brazil.Para|Loricariidae|
Peckoltia snethlageae|[38]|Brazil.Para|Loricariidae|
Peckoltia snethlageae|[39]|Brazil.Para|Loricariidae|
Peckoltia snethlageae|[40]|Brazil.Para|Loricariidae|
Peckoltia snethlageae|[41]|Brazil.Para|Loricariidae|
Peckoltia snethlageae|[42]|Brazil.Para|Loricariidae|
Peckoltia snethlageae|[43]|Brazil.Para|Loricariidae|
Peckoltia snethlageae|[44]|Brazil.Para|Loricariidae|
Peckoltia snethlageae|[45]|Brazil.Para|Loricariidae|
Peckoltia snethlageae|[46]|Brazil.Para|Loricariidae|
Actinopterygii|[47]|Taiwan||
Actinopterygii|[48]|Taiwan||
Panaque sp. L204|[49]||Loricariidae|
Panaqolus changae|[50]||Loricariidae|
Panaqolus sp. cara de pao|[51]|Brazil.Para|Loricariidae|
Panaqolus sp. cara de pao|[52]|Brazil.Para|Loricariidae|
Panaqolus sp. cara de pao|[53]|Brazil.Para|Loricariidae|
Panaqolus sp. cara de pao|[54]|Brazil.Para|Loricariidae|
Panaqolus sp. cara de pao|[55]|Brazil.Para|Loricariidae|
Unknown Specimen||
Hypancistrus furunculus|[56]|Brazil|Loricariidae|
Hypancistrus furunculus|[57]|Brazil|Loricariidae|
Hypancistrus furunculus|[58]||Loricariidae|
Hypancistrus debilittera|[59]|Brazil.Para|Loricariidae|
Hypancistrus debilittera|[60]|Brazil.Para|Loricariidae|
Hypancistrus debilittera|[61]|Brazil.Para|Loricariidae|
Hypancistrus debilittera|[62]|Brazil.Para|Loricariidae|
Hypancistrus debilittera|[63]|Brazil.Para|Loricariidae|
Hypancistrus debilittera|[64]|Brazil.Para|Loricariidae|
Hypancistrus debilittera|[65]|Brazil.Para|Loricariidae|
Hypancistrus debilittera|[66]|Brazil.Para|Loricariidae|
Hypancistrus debilittera|[67]|Brazil.Para|Loricariidae|
Hypancistrus debilittera|[68]|Brazil.Para|Loricariidae|
Hypancistrus debilittera|[69]|Brazil.Para|Loricariidae|
Hypancistrus debilittera|[70]|Brazil.Para|Loricariidae|
Hypancistrus debilittera|[71]|Brazil.Para|Loricariidae|
Hypancistrus debilittera|[72]|Brazil.Para|Loricariidae|
Hypancistrus debilittera|[73]|Brazil.Para|Loricariidae|
Hypancistrus debilittera|[74]|Brazil.Para|Loricariidae|
Hypancistrus debilittera|[75]|Brazil.Para|Loricariidae|
Hypancistrus debilittera|[76]|Brazil.Para|Loricariidae|
Hypancistrus debilittera|[77]|Brazil.Para|Loricariidae|
Hypancistrus debilittera|[78]|Brazil.Para|Loricariidae|
Hypancistrus sp. pao xg|[79]|Brazil.Para|Loricariidae|
Hypancistrus sp. pao xg|[80]|Brazil.Para|Loricariidae|
Hypancistrus sp. pao xg|[81]|Brazil.Para|Loricariidae|
Hypancistrus sp. pao xg|[82]|Brazil.Para|Loricariidae|
Hypancistrus sp. L66|[83]|Brazil.Para|Loricariidae|
Hypancistrus sp. pao tp|[84]|Brazil.Para|Loricariidae|
Hypancistrus sp. pao tp|[85]|Brazil.Para|Loricariidae|
Hypancistrus sp. pao tp|[86]|Brazil.Para|Loricariidae|
Hypancistrus sp. pao tp|[87]|Brazil.Para|Loricariidae|
Hypancistrus sp. pao tp|[88]|Brazil.Para|Loricariidae|
Hypancistrus sp.|[89]||Loricariidae|
Hypancistrus sp. zebra marrom|[90]|Brazil.Para|Loricariidae|
Hypancistrus sp. zebra marrom|[91]|Brazil.Para|Loricariidae|
Hypancistrus zebra|[92]|Brazil.Para|Loricariidae|
Hypancistrus zebra|[93]|Brazil.Para|Loricariidae|
Hypancistrus sp. zebra marrom|[94]|Brazil.Para|Loricariidae|
Hypancistrus zebra|[95]|Brazil.Para|Loricariidae|
Hypancistrus zebra|[96]|Brazil.Para|Loricariidae|
Hypancistrus zebra|[97]|Brazil.Para|Loricariidae|
Hypancistrus sp. zebra marrom|[98]|Brazil.Para|Loricariidae|
Hypancistrus zebra|[99]|Brazil.Para|Loricariidae|
Panaqolus sp.
Field number: sp4
Voucher: MNRJ43654

COI_FULL_DATABASE_includes_records_without_species_designati... Mon Apr 11 14:20:28 2016 Page 1 of 1

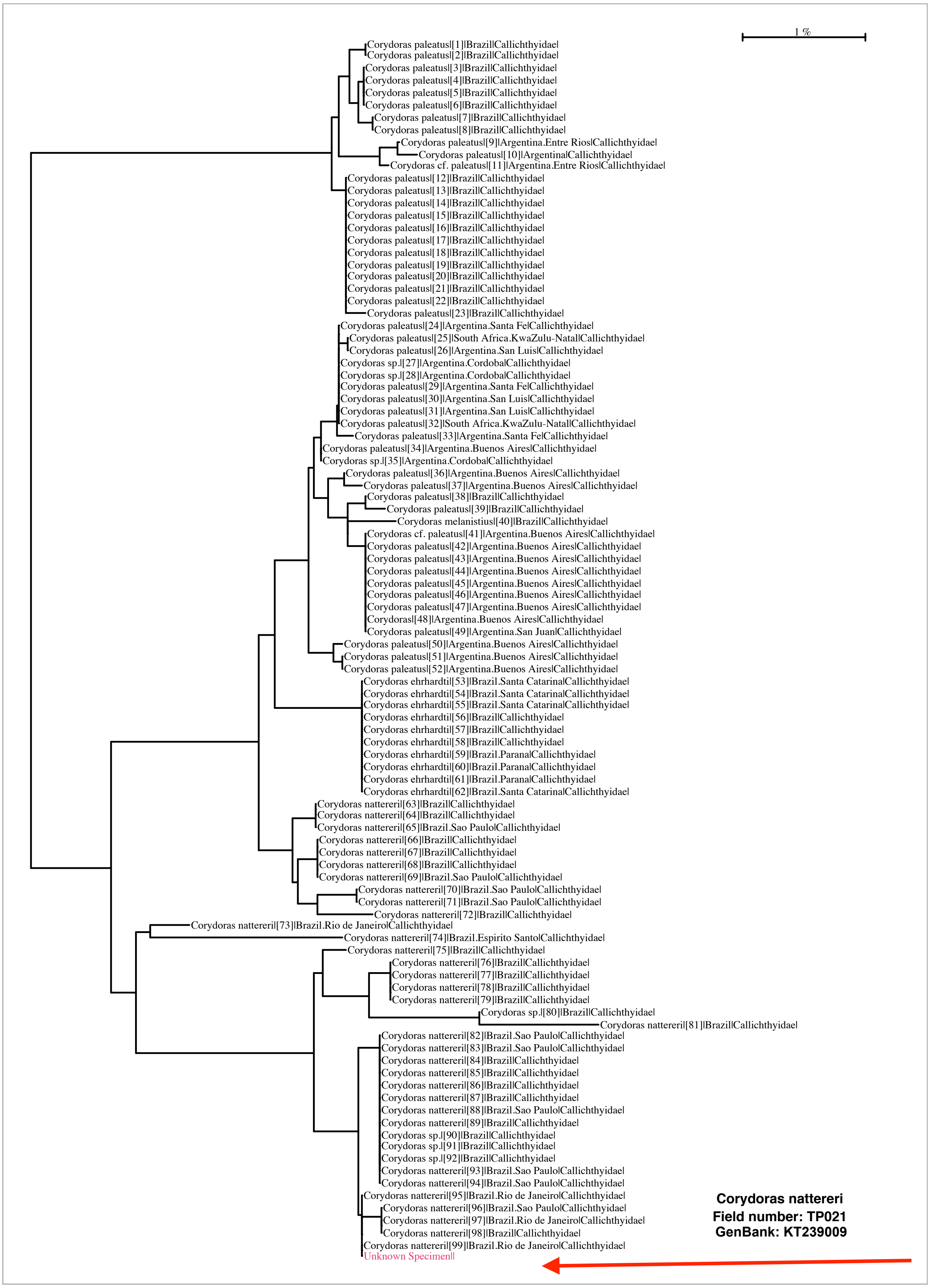

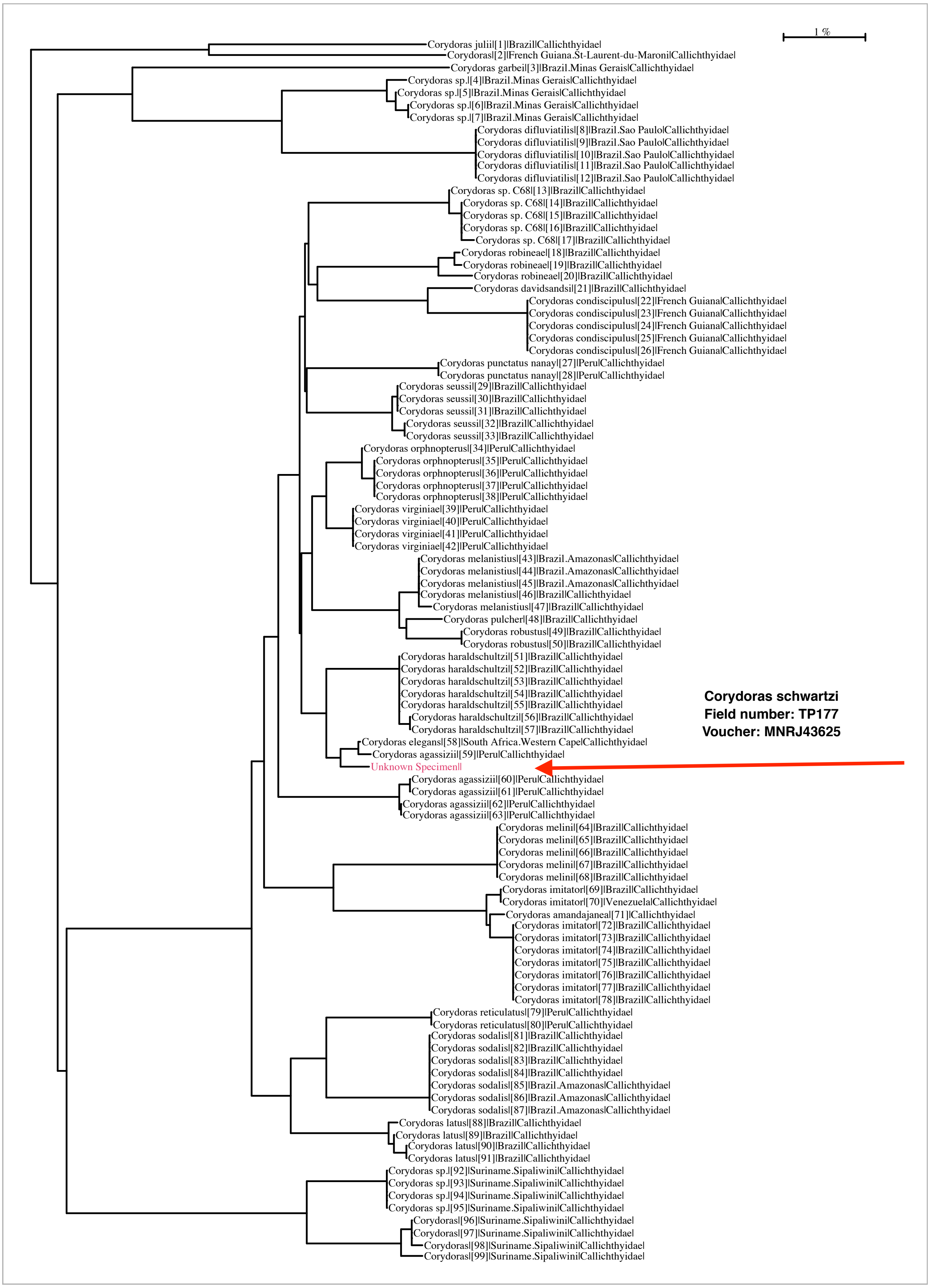
1 %
Corydoras julii|[1]|Brazil|Callichthyidae|
Corydoras|[2]|French Guiana.St-Laurent-du-Maroni|Callichthyidae|
Corydoras garbei|[3]|Brazil.Minas Gerais|Callichthyidae|
Corydoras sp.|[4]|Brazil.Minas Gerais|Callichthyidae|
Corydoras sp.|[5]|Brazil.Minas Gerais|Callichthyidae|
Corydoras sp.|[6]|Brazil.Minas Gerais|Callichthyidae|
Corydoras sp.|[7]|Brazil.Minas Gerais|Callichthyidae|
Corydoras difluviatilis|[8]|Brazil.Sao Paulo|Callichthyidae|
Corydoras difluviatilis|[9]|Brazil.Sao Paulo|Callichthyidae|
Corydoras difluviatilis|[10]|Brazil.Sao Paulo|Callichthyidae|
Corydoras difluviatilis|[11]|Brazil.Sao Paulo|Callichthyidae|
Corydoras difluviatilis|[12]|Brazil.Sao Paulo|Callichthyidae|
Corydoras sp. C68|[13]|Brazil|Callichthyidae|
Corydoras sp. C68|[14]|Brazil|Callichthyidae|
Corydoras sp. C68|[15]|Brazil|Callichthyidae|
Corydoras sp. C68|[16]|Brazil|Callichthyidae|
Corydoras sp. C68|[17]|Brazil|Callichthyidae|
Corydoras robineae|[18]|Brazil|Callichthyidae|
Corydoras robineae|[19]|Brazil|Callichthyidae|
Corydoras robineae|[20]|Brazil|Callichthyidae|
Corydoras davidsandsi|[21]|Brazil|Callichthyidae|
Corydoras condiscipulus|[22]|French Guiana|Callichthyidae|
Corydoras condiscipulus|[23]|French Guiana|Callichthyidae|
Corydoras condiscipulus|[24]|French Guiana|Callichthyidae|
Corydoras condiscipulus|[25]|French Guiana|Callichthyidae|
Corydoras condiscipulus|[26]|French Guiana|Callichthyidae|
Corydoras punctatus nanay|[27]|Peru|Callichthyidae|
Corydoras punctatus nanay|[28]|Peru|Callichthyidae|
Corydoras seussi|[29]|Brazil|Callichthyidae|
Corydoras seussi|[30]|Brazil|Callichthyidae|
Corydoras seussi|[31]|Brazil|Callichthyidae|
Corydoras seussi|[32]|Brazil|Callichthyidae|
Corydoras seussi|[33]|Brazil|Callichthyidae|
Corydoras orphnopterus|[34]|Peru|Callichthyidae|
Corydoras orphnopterus|[35]|Peru|Callichthyidae|
Corydoras orphnopterus|[36]|Peru|Callichthyidae|
Corydoras orphnopterus|[37]|Peru|Callichthyidae|
Corydoras orphnopterus|[38]|Peru|Callichthyidae|
Corydoras virginiae|[39]|Peru|Callichthyidae|
Corydoras virginiae|[40]|Peru|Callichthyidae|
Corydoras virginiae|[41]|Peru|Callichthyidae|
Corydoras virginiae|[42]|Peru|Callichthyidae|
Corydoras melanistius|[43]|Brazil.Amazonas|Callichthyidae|
Corydoras melanistius|[44]|Brazil.Amazonas|Callichthyidae|
Corydoras melanistius|[45]|Brazil.Amazonas|Callichthyidae|
Corydoras melanistius|[46]|Brazil|Callichthyidae|
Corydoras melanistius|[47]|Brazil|Callichthyidae|
Corydoras pulcher|[48]|Brazil|Callichthyidae|
Corydoras robustus|[49]|Brazil|Callichthyidae|
Corydoras robustus|[50]|Brazil|Callichthyidae|
Corydoras haraldschultzi|[51]|Brazil|Callichthyidae|
Corydoras haraldschultzi|[52]|Brazil|Callichthyidae|
Corydoras haraldschultzi|[53]|Brazil|Callichthyidae|
Corydoras haraldschultzi|[54]|Brazil|Callichthyidae|
Corydoras haraldschultzi|[55]|Brazil|Callichthyidae|
Corydoras haraldschultzi|[56]|Brazil|Callichthyidae|
Corydoras haraldschultzi|[57]|Brazil|Callichthyidae|
Corydoras elegans|[58]|South Africa.Western Cape|Callichthyidae|
Corydoras agassizii|[59]|Peru|Callichthyidae|
Unknown Specimen||
Corydoras agassizii|[60]|Peru|Callichthyidae|
Corydoras agassizii|[61]|Peru|Callichthyidae|
Corydoras agassizii|[62]|Peru|Callichthyidae|
Corydoras agassizii|[63]|Peru|Callichthyidae|
Corydoras melini|[64]|Brazil|Callichthyidae|
Corydoras melini|[65]|Brazil|Callichthyidae|
Corydoras melini|[66]|Brazil|Callichthyidae|
Corydoras melini|[67]|Brazil|Callichthyidae|
Corydoras melini|[68]|Brazil|Callichthyidae|
Corydoras imitator|[69]|Brazil|Callichthyidae|
Corydoras imitator|[70]|Venezuela|Callichthyidae|
Corydoras amandajanea|[71]||Callichthyidae|
Corydoras imitator|[72]|Brazil|Callichthyidae|
Corydoras imitator|[73]|Brazil|Callichthyidae|
Corydoras imitator|[74]|Brazil|Callichthyidae|
Corydoras imitator|[75]|Brazil|Callichthyidae|
Corydoras imitator|[76]|Brazil|Callichthyidae|
Corydoras imitator|[77]|Brazil|Callichthyidae|
Corydoras imitator|[78]|Brazil|Callichthyidae|
Corydoras reticulatus|[79]|Peru|Callichthyidae|
Corydoras reticulatus|[80]|Peru|Callichthyidae|
Corydoras sodalis|[81]|Brazil|Callichthyidae|
Corydoras sodalis|[82]|Brazil|Callichthyidae|
Corydoras sodalis|[83]|Brazil|Callichthyidae|
Corydoras sodalis|[84]|Brazil|Callichthyidae|
Corydoras sodalis|[85]|Brazil.Amazonas|Callichthyidae|
Corydoras sodalis|[86]|Brazil.Amazonas|Callichthyidae|
Corydoras sodalis|[87]|Brazil.Amazonas|Callichthyidae|
Corydoras latus|[88]|Brazil|Callichthyidae|
Corydoras latus|[89]|Brazil|Callichthyidae|
Corydoras latus|[90]|Brazil|Callichthyidae|
Corydoras latus|[91]|Brazil|Callichthyidae|
Corydoras sp.|[92]|Suriname.Sipaliwini|Callichthyidae|
Corydoras sp.|[93]|Suriname.Sipaliwini|Callichthyidae|
Corydoras sp.|[94]|Suriname.Sipaliwini|Callichthyidae|
Corydoras sp.|[95]|Suriname.Sipaliwini|Callichthyidae|
Corydoras|[96]|Suriname.Sipaliwini|Callichthyidae|
Corydoras|[97]|Suriname.Sipaliwini|Callichthyidae|
Corydoras|[98]|Suriname.Sipaliwini|Callichthyidae|
Corydoras|[99]|Suriname.Sipaliwini|Callichthyidae|
Corydoras schwartzi
Field number: TP177
Voucher: MNRJ43625
